# The efficacy of Pembrolizumab, Ipilimumab, and Nivolumab monotherapy and combination for colorectal cancer: A systematic review and meta-analysis

**DOI:** 10.1371/journal.pone.0307128

**Published:** 2025-11-14

**Authors:** Albertus Ari Adrianto, Ignatius Riwanto, Udadi Sadhana, Dewi Kartikawati Paramita, Henry Setyawan, Kevin Christian Tjandra, Danendra Rakha Putra Respati, Derren David Christian Homenta Rampengan, Roy Novri Ramadhan, Gastin Gabriel Jangkang, Endang Mahati, Patricia Winona

**Affiliations:** 1 Department of Digestive Surgery, Faculty of Medicine, Universitas Diponegoro, Semarang, Indonesia; 2 Department of Anatomical Pathology, Faculty of Medicine, Universitas Diopnegoro, Semarang, Indonesia; 3 Department of Histology and Cell Biology, Faculty of Medicine, Public Health and Nursing, Universitas Gadjah Mada, Yogyakarta, Indonesia; 4 Integrated Research Laboratory, Faculty of Medicine, Public Health and Nursing, Universitas Gadjah Mada, Yogyakarta, Indonesia; 5 Study Center for Biotechnology, Universitas Gadjah Mada, Yogyakarta, Indonesia; 6 Department of Public Health, Faculty of Public Health, Universitas Diponegoro, Semarang, Indonesia; 7 Department of Medicine, Faculty of Medicine, Universitas Diponegoro, Semarang, Indonesia; 8 Medical Program, Faculty of Medicine, Universitas Sam Ratulangi, Manado, Indonesia; 9 Medical Program, Faculty of Medicine, Universitas Airlangga, Surabaya, Indonesia; 10 Medical Program, Faculty of Medicine, Universitas Lambung Mangkurat, Banjarmasin, Indonesia; 11 Department of Pharmacology and Therapeutic, Faculty of Medicine, Diponegoro University, Semarang, Indonesia; 12 School of Medicine and Health Sciences, Atma Jaya Catholic University of Indonesia, Jakarta, Indonesia; Roswell Park Cancer Institute, UNITED STATES OF AMERICA

## Abstract

**Background:**

*Colorectal cancer* (*CRC*) is the third leading cause of cancer-related deaths worldwide, with cases expected to rise 60% by 2030, especially in Asia. Metastatic CRC (mCRC) has a poor 5-year survival rate of 14%, posing a major treatment challenge. Tumors with DNA mismatch repair deficiency (dMMR) and a high level of microsatellite instability (MSI-H) respond well to *immune checkpoint inhibitors* (*ICIs*), shifting treatment strategies. This systematic review and meta-analysis evaluate Pembrolizumab (PEM), Nivolumab (NIV), and Nivolumab plus Ipilimumab (NIV *+* IPI) for their promising antitumor efficacy in MSI-H/dMMR mCRC.

**Methods:**

This systematic review followed PRISMA guidelines and Cochrane Handbook standards, covering studies from 2014 to 2024 on advanced CRC patients treated with ICIs. A comprehensive search across eight databases was conducted by 12 independent reviewers. Extracted outcomes included *overall survival* (*OS*), *progression-free survival* (*PFS*), *disease control rate* (*DCR*), and *objective response rate* (*ORR*). To facilitate pooled analysis, data reported as median and *interquartile range* (*IQR*), or median, minimum, and maximum were converted to mean and *standard deviation* (*SD*) using combined formulas by Luo D et al. and Wan X et al. Risk of bias was assessed using the Cochrane RoB 2 tool. Meta-analyses were performed using random-effects models, with subgroup analyses by dosage. Publication bias and sensitivity analyses were conducted. All statistical analyses used RevMan version 5.4.

**Results:**

A total of 13 eligible studies were analyzed, with sample sizes ranging from 11 to 307 and follow-up durations between 5.3 and 44.5 months. NIV *+* IPI showed the highest efficacy across all endpoints: ORR 0.54 [95% CI: 0.45–0.65, I² *=* 75%], OS 0.84 [95% CI: 0.81–0.88, I² *=* 0%], PFS 0.73 [95% CI: 0.68–0.78, I² *=* 0%], and DCR 0.82 [95% CI: 0.77–0.86, I² *=* 0%]. This combination outperformed NIV alone, which demonstrated ORR 0.36 [95% CI: 0.21–0.60, I² *=* 81%], OS 0.73 [95% CI: 0.62–0.86, I² *=* 54%], PFS 0.54 [95% CI: 0.43–0.68, I² *=* 34%], and DCR 0.70 [95% CI: 0.64–0.77, I² *=* 0%]. PEM showed lower efficacy with ORR 0.33 [95% CI: 0.23–0.49, I² *=* 94.6%], OS 0.59 [95% CI: 0.31–0.66, I² *=* 94%], PFS 0.45 [95% CI: 0.31–0.66, I² *=* 84%], and DCR 0.73 [95% CI: 0.47–1.12, I² *=* 94%]. PEM’s 200 mg dosage subgroup exhibited the best performance in its group with an ORR of 0.45 [95% CI: 0.38–0.52, I² *=* 0%]. Despite these findings, heterogeneity was notably high in PEM-related studies, highlighting variability in populations and study designs. Overall, NIV *+* IPI demonstrated superior and more consistent clinical outcomes.

**Conclusions:**

This study highlights NIV *+* IPI as a promising combination for advanced CRC, showing superior efficacy, while PEM also demonstrated potential. However, high heterogeneity suggests the need for further research. Acknowledging its limitations, this study marks a pioneering effort in comparing short- and long-term effects of anti-CTLA-4 and anti-PD-1 therapies, paving the way for future advancements in CRC treatment.

## Introduction

Colorectal cancer ranks as the third most common cause of cancer-related mortality globally [1]. Over the last thirty years, the incidence of CRC has increased significantly in many countries, particularly in Asia, where a substantial rise has been observed. Some regions in Asia, specifically East Asia, have reported a rise of 85.2% in incidence rates, while Southeast Asia has reported a 40.9% rise in these rates [2]. Projections indicate a significant rise in its worldwide incidence, with an expected increase of 60% by the year 2030 [1,3]. The prognosis remains poor for individuals diagnosed with CRC, as evidenced by a 5-year survival rate of only 14% [4]. The incidence of CRC and its associated mortality rate rise with advancing age, with a median age of diagnosis typically reported as 68 years old. A striking 93% of CRC-related deaths are observed in individuals aged 50 years and older. The relative survival rates at 5 and 10 years post-diagnosis for CRC stand at 65% and 58%, respectively [5].

Until recently, the treatment choices available for individuals with inoperable CRC worldwide have been predominantly confined to chemotherapy and targeted therapies [6]. Findings from several clinical trials indicate that CRC tumors identified by dMMR and MSI-H exhibit susceptibility to treatment with ICIs [7–9]. Tumors characterized as MSI-H/dMMR possess an elevated tumor mutational burden, leading to the generation of neoantigens, thereby rendering MSI-H/dMMR tumors more immunogenic compared to those with proficient DNA mismatch repair mechanisms. Consequently, MSI-H/dMMR tumors tend to harbor increased numbers of tumor-infiltrating lymphocytes, whose function can be potentiated by ICI therapy. Patients with MSI-H/dMMR mCRC typically derive less benefit from standard chemotherapy compared to those with microsatellite stable/mismatch repair-proficient mCRC [10,11]. However, MSI-H/dMMR status serves as a predictive indicator of favorable response to anti-programmed death (PD)-1 checkpoint inhibitor therapy [8,12,13].

The interaction involving the PD-1 receptor and its ligands, PD-L1 and PD-L2, typically serves as an immune checkpoint mechanism responsible for regulating the equilibrium between T-cell activation, immune tolerance, and preventing immune-related tissue damage. This pathway is often exploited by tumors to evade immune surveillance [14]. PD-1 is present on T, B, and natural killer T cells, as well as activated monocytes and a significant portion of tumor-infiltrating lymphocytes in various malignancies [14]. When PD-L1 (found on cells of diverse lineages) or PD-L2 (present on macrophages and dendritic cells) binds to the PD-1 receptor, it ultimately leads to the inhibition of T-cell function. Both PD-1 ligands have the potential to be expressed either constitutively or induced in various cell types, which also includes tumor cells [14]. PEM is a selective humanized monoclonal antibody of the immunoglobulin G4/κ type, designed to specifically hinder the interaction between PD-1 and PD-L1/PD-L2 by directly binding to PD-1 [15]. Previous studies have shown that checkpoint inhibitors targeting PD-1 and cytotoxic T-lymphocyte-associated protein 4 (CTLA-4), including NIV and IPI, respectively, synergize and stimulate an immune response against tumors through separate but complementary mechanisms. NIV, a human monoclonal antibody blocking PD-1, has received accelerated approval from the U.S. Food and Drug Administration (FDA) for use either alone or in combination with low-dose ipilimumab in patients diagnosed with MSI-H or dMMR mCRC. Another findings also reported that both PEM and NIV, inhibitors targeting PD-1, have demonstrated significant antitumor efficacy in patients with previously treated MSI-H/dMMR mCRC [7–9].

Through PD-1 pathway inhibition, PD-1 blockers reactivate T-cells, allowing them to recognize and attack tumor cells more effectively. This reactivation can significantly improve clinical outcomes, particularly in terms of PFS and OS. In clinical studies, patients with MSI-H/dMMR mCRC treated with PD-1 inhibitors have shown prolonged PFS, with delayed disease progression compared to traditional therapies. Additionally, these therapies often lead to durable responses, meaning that the benefits can last much longer, even when the initial response may appear slow. This durability is particularly important in extending OS, offering some patients the chance of long-term remission or significantly improved life expectancy. Ultimately, targeting the PD-1 pathway represents a major advancement in cancer therapy, providing a more personalized and potentially long-lasting treatment option for patients with certain genetic profiles, such as MSI-H/dMMR mCRC [7–9].

Due to their distinct mechanism of action, ICIs are linked to adverse events (AEs) that contrast with those typically associated with chemotherapy. These immune-related AEs commonly impact various bodily systems including the skin, gastrointestinal (GI) tract, lungs, kidneys, endocrine glands, and liver. It is also often associated with immune-related adverse events (irAEs) such as pneumonitis, colitis, hepatitis, and thyroid dysfunction. These irAEs occur in approximately 15−20% of patients receiving PD-1 inhibitors, with severe (Grade 3−4) events occurring in about 3−5%. In contrast, traditional chemotherapy tends to produce a different set of toxicities, including myelosuppression (low blood cell counts), neuropathy (nerve damage), and gastrointestinal issues such as severe nausea and vomiting (Brahmer et al., 2018). For instance, chemotherapy-induced neutropenia, a common and potentially life-threatening side effect, can occur in 25−40% of patients, often requiring hospitalization or treatment delays. Chemotherapy-induced neuropathy, which can be permanent in some cases, affects approximately 30% of patients. In contrast, while PD-1 inhibitors carry the risk of autoimmune complications, these events are typically less frequent, more manageable, and reversible with corticosteroid treatment or other immunosuppressants. Additionally, PD-1 inhibitors generally result in lower rates of fatigue, nausea, and cytopenias compared to chemotherapy [16,17].

Timely detection and appropriate management of these AEs, which may involve the use of systemic corticosteroids as necessary, could enhance outcomes for patients undergoing checkpoint inhibitor therapy. Understanding the predictors of response and resistance to immunotherapy is crucial for identifying patients who are most likely to benefit from these agents. PEM, NIV, and IPI represent novel therapeutic options for patients with advanced CRC, offering the potential for durable responses and improved survival outcomes. Therefore, this systematic review and meta-analysis aims to provide a comprehensive overview of the efficacy of PEM, NIV, and IPI in advanced CRC, with insights into future directions and challenges in the immunotherapy landscape.

## Materials and methods

### Registration

The systematic review and meta-analysis titled “The Efficacy of PEM, NIV, and IPI Monotherapy and Combination for Advanced Colorectal Cancer: A Systematic Review and Meta-Analysis” was officially registered on the PROSPERO on 24 February 2024 with registration number: CRD42024512511

### Eligibility criteria

This review adhered to the guidelines stipulated by the Preferred Reporting Items for Systematic Reviews and Meta-Analyses (PRISMA) statement and the Cochrane Handbook [18,19]. The inclusion criteria encompassed studies involving patients of any age diagnosed with advanced or mCRC, regardless of microsatellite instability-high and/or mismatch repair-deficient (MSI-H/ dMMR) status, treated by PEM, NIV, and IPI as the monotherapy or combination therapy. The primary focus was assessing the efficacy of this treatment. The criteria for inclusion necessitated the availability of data on the mentioned primary outcomes published from 2014 until 2024. Furthermore, the eligible studies were restricted to observational designs (cohort/case-control) or randomized clinical trials (RCTs) or clinical trial.

The exclusion criteria were technical reports, editor responses, narrative reviews, systematic reviews, meta-analyses, non-comparative research, in silico studies, in vitro studies, in vivo studies, scientific posters, study protocols, and conference abstracts. For data synthesis, the study will be grouped based on relevant comparators that include the use of NIV + IPI, NIV, and PEM monotherapy.

### Literature search and study selection

A comprehensive search of English literature across eight international databases— Scopus, PubMed, Cochrane Library, Sage pub, ProQuest, Science Direct, Research Gate, Epistemonikos—was conducted by a team of 12 independent authors from inception until February 1st, 2024. To capture all potentially relevant literature, a combination of keywords was utilized, employed the following keywords: “Pembrolizumab” or “Nivolumab” or “Ipilimumab” and “colorectal carcinoma” and “therapeutic”. The studies were organized and managed using the Mendeley Group Reference Manager in the authors’ library. Initially, duplicate articles were removed, followed by screening based on titles and abstracts. Each researcher independently reviewed the titles and abstracts, resolving any disparities through discussion until reaching an agreement. Subsequently, they collaborated in 6 pairs of group to review the titles and abstracts of all retrieved articles. If necessary, the corresponding author was involved to make the final decision. Articles that passed this initial screening underwent full-text evaluation against predetermined inclusion/exclusion criteria. The full-text screening was conducted by the same 12 independent researchers with any discrepancies resolved through discussion with the corresponding author as the final decision maker (Table 1).

**Table 1:** Keyword Used in Literature Searching.

Database	Keywords
Scopus	“Pembrolizumab” or “Nivolumab” or “Ipilimumab” and “colorectal carcinoma” and “therapeutic”
PubMed	
Cochrane Library	
Sage pub	
ProQuest	
Science Direct	
Research Gate	
Epistemonikos	

### Data extraction

The following data were extracted for analysis: study ID, publication year, country, study design, sample size, participant characteristics (such as average age and sex distribution), follow-up duration, the number of participants in the PEM, NIV, and NIV + IPI utilization, and key outcomes. Twelve independent authors carried out the data extraction, organizing everything in Microsoft Excel 2019.

For the primary outcomes, we focused on survival and therapy response. Survival was measured using OS and PFS, capturing both the treatment period and the time afterward, where the disease either remained stable or showed no signs of progression. Therapy response was evaluated using ORR and DCR to get a well-rounded picture of how effective the intervention was in individuals with CRC.

When it came to missing data, we did our best to fill in the gaps. If key numbers were missing, we reached out to the original study authors for clarification. When that wasn’t possible, we estimated missing continuous variables based on reported medians and interquartile ranges or, in some cases, reconstructed them from graphs. For categorical data, we ran sensitivity analyses to assess the impact of missing values, and if a study had too much missing data, we left it out of the final analysis. The full data extraction results can be found in Table 2.

**Table 2:** Study Characteristic and Data Extraction.

Study	Data Extractor	Data Extraction Date	Design	N	Treatment	Control	Age [Median (Min-Max)]	Male/ Female	Follow-up	Survival Analysis (6–12 months)			
										OS	PFS	ORR	DCR
**PEM**													
O’Neil et al; 2017	AAD	15-Feb-24	Cohort	23	PEM IV (10 mg/kg)	N/A	57.0 (40.0–78.0)	13/10	6 months	0.435 (95% CI, 0.232–0.815)	0.174 (95% CI, 0.092–0.328)	4% [95% CI, 0.1–22}	NR
Ott et al; 2017	IR	15-Feb-24	Cohort	25	PEM IV (10 mg/kg)	N/A	63.0 (46.0–82.0)	2/23	10.6 months	0.476 (95% CI, 0.280–0.809)	0.197 (95% CI, 0.112–0.352)	17%[95% CI, 22 5% − 37%]	58.% (48%−70%)
Diaz et al; 2022	US	15-Feb-24	RCT	307	PEM IV (300 mg)	Chemotherapy	62.5 (24.0–93.0)	30/27	44.5 months	0.780 (95% CI, 0.650–0.935)	0.770 (95% CI, 0.725–0.817)	45.1% [95% CI, 37.1–53.3]	NR
André et al; 2021	DKP	15-Feb-24	RCT	307	PEM IV (200 mg)	Chemotherapy	63.0 (24.0–93.0)	153/154	NR	0.438 (95% CI, 0.358–0.52)	0.6 (95% CI, 0.45–0.80)	43.8% [95% CI, 35.8–52.0]	NR
Yoshino et al;2023	HS	15-Feb-24	RCT	48	PEM IV (200 mg)	Chemotherapy	64.0 (24.0–83.0)	23/25	45.3 months	0.770 (95% CI, 0.950–0.912)	0.62 (95% CI, 0.507–0.757)	50.0% [95% CI, 28.2–71.8]	NR
Le et al; 2015	KCT	15-Feb-24	Nonrandomized	41	PEM IV (10 mg/kg)	N/A	46.0 (24.0–65.0)	24/17	31.3 months	NR	NR	40% [95% CI, 12–74]	90% [95% CI, 55–100]
**NIV**													
Okuma et al; 2023	KCT	15-Feb-24	Nonrandomized	11	NIV (240 mg)	N/A	70.0 (54.0–78.0)	3/8	24.7 months	0.80 (95% CI, 70.9–94.6)	0.4 (95% CI, 0.232–0.689)	60% [95% CI, 26.2–87.8]	70% [95% CI, 34.8–93.3]
Overman et al; 2017	DRPR	15-Feb-24	Nonrandomized	74	NIV (3 mg/kg)	N/A	52·5 (44·0–64·0)	44/30	12.0 months	0.734 (95%, 0.615 - 0.821)	0.504 (95% CI, 0.381–0.614)	31·1% (95% CI 20·8% –42·9%	68·9% (95% CI 57·1%–79·2%)
Morris et al; 2017	DDCHR	15-Feb-24	Nonrandomized	37	NIV (3 mg/kg)	N/A	56.0 (51.0–64.0)	10/27	10.1 months	0.52 (95% CI, 0.350–0.772)	0.63 (95% CI, 0.505–0.785)	0.24 (0.15-0.377)	72% [95% CI, 53–84]
**NIV + IPI**													
Lenz et al; 2021	RNR	15-Feb-24	Cohort	45	NIV (3 mg/kg) + IPI (1 mg/kg)	N/A	66.0 (21.0-85.0)	23/22	24.2 months	0.841 (69.5 to 92.1)	0.764 (60.5 to 86.6)	62% [95% CI, 46.5–76.2]	78% [95% CI, 63–89]
Overman et al; 2018	GGJ	15-Feb-24	Cohort	119	NIV (3 mg/kg) + IPI (1 mg/kg)	N/A	58.0 (21.0-88.0)	70/49	13.4 months	0.85 (0.78-0.93)	0.71 (95% CI, 0.638–0.791)	55% (95% CI, 45.2 to 63.8)	80% (95% CI, 71.5 to 86.6)
Morse et al; 2019	EM	15-Feb-24	Cohort	119	NIV (3 mg/kg) + IPI (1 mg/kg)	N/A	NR	NR	NR	0.84 (0.718-0.907)	0.70 (55.75-80.45)	61% [95% CI, 52–70}	82% [95% CI, 74–89}
Cohen et al; 2020	PW	15-Feb-24	Nonrandomized	57	NIV (3 mg/kg) + IPI (1 mg/kg)	N/A	56.5 (45.8–63.8)	30/27	18.1 months	0.84 (0.75-0.95)	0.729 (95% CI, 0.629–0.844)	35% (0.26-0.47)	87% [95% CI, 0.77-- 1.0]

NR = Not Reported; NA = Not Applicable; PEM = Pembrolizumab NIV = Nivolimumab; IPI = Ipilimumab.

### Risk of bias assessment

The evaluation of bias risk was carried out by five independent authors utilizing validated instruments. To assess the quality of the included Randomized Controlled Trials (RCTs), the Cochrane Collaboration’s Risk of Bias version 2 (RoB v2) tool was employed, comprising methodological assessments across five domains: (a) randomization process; (b) deviations from intended interventions; (c) missing outcome data; (d) measurement of the outcome; and (e) selection of the reported results. The outcomes are depicted in Fig 2. The authors’ appraisals were categorized as “low risk,” “high risk,” or “some concerns” of bias. Two review authors (KCT, DR) independently utilized the tool for each included study and documented supporting information for their assessments of risk of bias in each domain. In evaluating the quality of included cohort/case-control studies or non-randomized clinical trial, the Risk Of Bias In Non-randomized Studies – of Interventions (ROBINS-I) was utilized, covering seven domains: (1) confounding; (2) selection of participants; (3) classification of intervention; (4) deviation from intended intervention; (5) missing data; (6) measurement of outcomes; (7) selection of the reported result. The authors’ appraisals were categorized as “low risk,” “high risk,” or “some concerns” of bias. Two review authors (KCT, DR) independently utilized the tool for each included study and documented supporting information for their assessments of risk of bias in each domain. Any disparities in risk of bias assessments or justifications were addressed through discussion to achieve consensus between the two review authors (KCT, DR) If needed, corresponding author (EM) served as an arbiter to facilitate resolution

**Fig 1:**
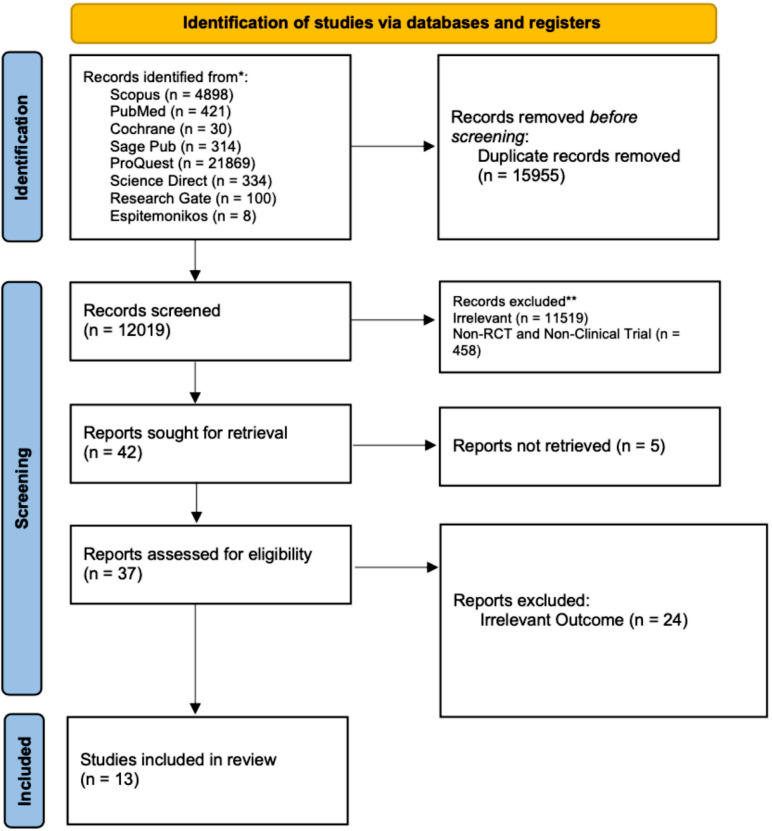
PRISMA 2020 flow diagram.

### Statistical analysis

For the analytical synthesis of continuous variable outcomes, we employed the mean difference (MD) with 95% confidence intervals (95% CI), utilizing the Inverse-Variance formula. Dichotomous variable outcomes were pooled into risk ratios (RR) with 95% CI using the Mantel-Haenszel formula. The PICOS criteria necessitated a significant follow-up period in each trial to mitigate potential bias arising from heavily censored data in the tails of treatment arms during the observation period. Additionally, the funnel plots also indicated evidence of heterogeneity for these outcomes. Heterogeneity between studies was assessed using the I-squared (I^2^) statistic, where values exceeding 50% were categorized as significant heterogeneity. To facilitate pooled analysis, we employed a combined formula from Luo D et al. [15] and Wan X et al. [16] for transforming data expressed as the median and IQR, or median, minimum, and maximum into MD and standard deviations (SD). All statistical analysis was performed utilizing the RevMan 5.4 application

### Reporting bias and certainity assessment

Publication bias analysis was conducted when there were over 10 studies for each interest outcome. In cases of funnel plot asymmetry, a thorough examination of characteristics aimed to discern whether observed asymmetry could be attributed to publication bias or other factors like methodological heterogeneity among studies. All statistical analyses were executed using RevMan 5.4 application. The robustness of outcomes was scrutinized through sensitivity meta-analysis, exclusively considering studies with an overall low risk of bias. To further assess the robustness of outcomes, a sensitivity meta-analysis was conducted, focusing solely on studies with an overall low risk of bias. This approach helps to confirm the stability of the findings by excluding studies with a higher risk of bias, thus refining the interpretation of the pooled results.

## Result

### Study selection and characteristics

We conducted a thorough literature search across eight international databases, identifying a total of 27,974 studies. To refine our selection, we applied filters based on the release date and article type, which automatically removed 15,955 duplicate records before moving on to the screening stage.

Next, we carefully reviewed the titles and abstracts, eliminating 11,519 studies that were not relevant to our research focus. Additionally, 458 studies were excluded for not being randomized controlled trials (RCTs) or clinical trials, leaving us with 42 studies for further evaluation.

A deeper, full-text assessment of these 42 studies led to the exclusion of 24 studies that did not align with our desired outcomes, and unfortunately, 5 studies could not be retrieved. In the end, we included 13 studies that met all eligibility criteria, as shown in Fig 1.

The sample sizes of these studies ranged from 11 to 307 participants, with follow-up periods spanning from 5.3 months to 44.5 months. A detailed breakdown of the baseline characteristics of the included studies is available in Table 2.

For full transparency, the detailed study selection process, including reasons for inclusion and exclusion, can be found in S1 File.

### Risk of bias in studies

The risk of bias in the included studies was evaluated using The Revised Tool for Risk of Bias in Randomized Trials (RoB 2.0) for randomized studies and the Risk Of Bias In Non-randomised Studies – of Interventions (ROBINS-I) for non-randomized studies. The assessment results indicated that 80% of the randomized studies had a low risk of bias, while 75% of the non-randomized studies exhibited a low risk of bias.

The risk of bias assessment using the RoB 2.0 tool showed that one randomized controlled trial (RCT) had “some concerns” regarding the randomization process and deviations from intended interventions. The remaining four RCTs were judged to have a “low” risk of bias across all five assessment domains.

**Fig 2:**
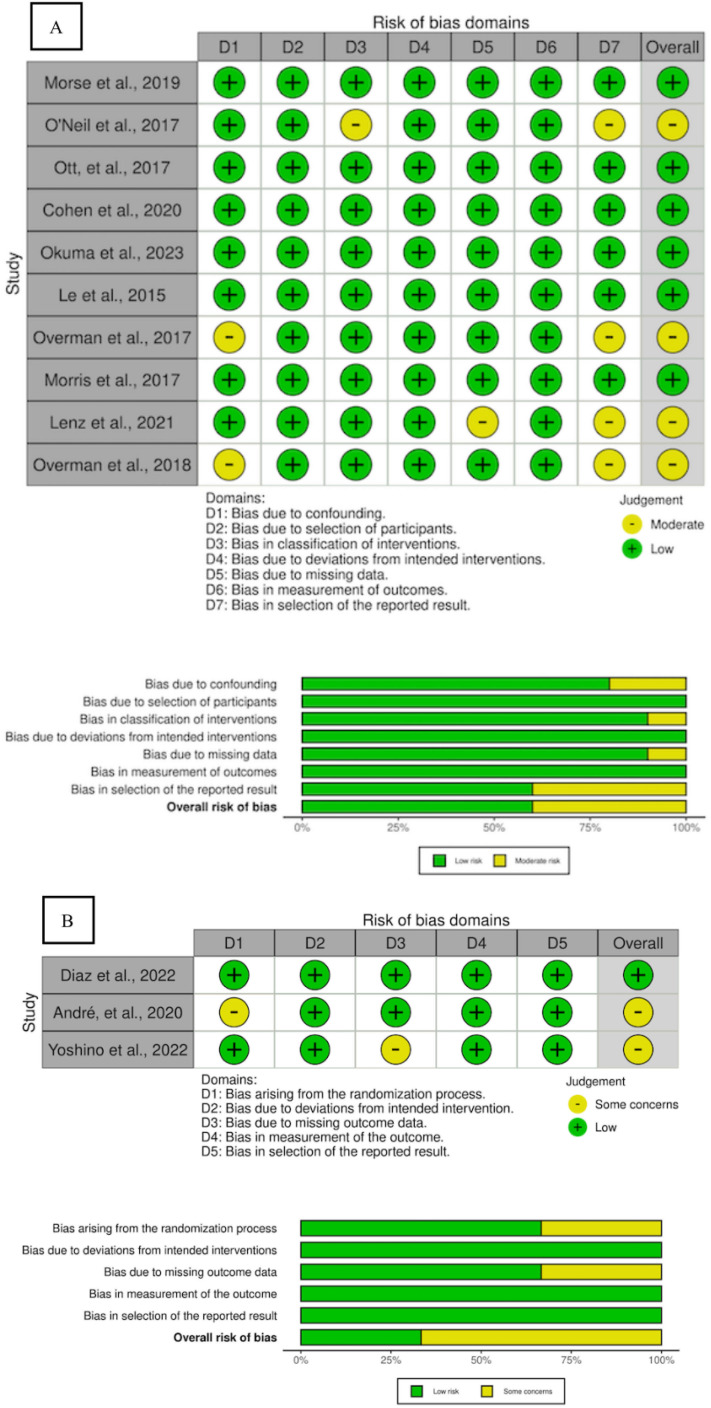
(A) ROBINS-I Risk of Bias Assessment; (B) ROB-2 Risk of Bias Assessment.

For non-randomized studies, the ROBINS-I tool revealed that three studies had a “moderate” risk of bias due to confounding and selection of participants, while the remaining nine studies demonstrated a “low” risk of bias. These findings are summarized in Fig 2: (A) ROBINS-I Risk of Bias Assessment; (B) ROB-2 Risk of Bias Assessment. The assessment bias process is available in S2 File.

### Objective response rate

**Fig 3:**
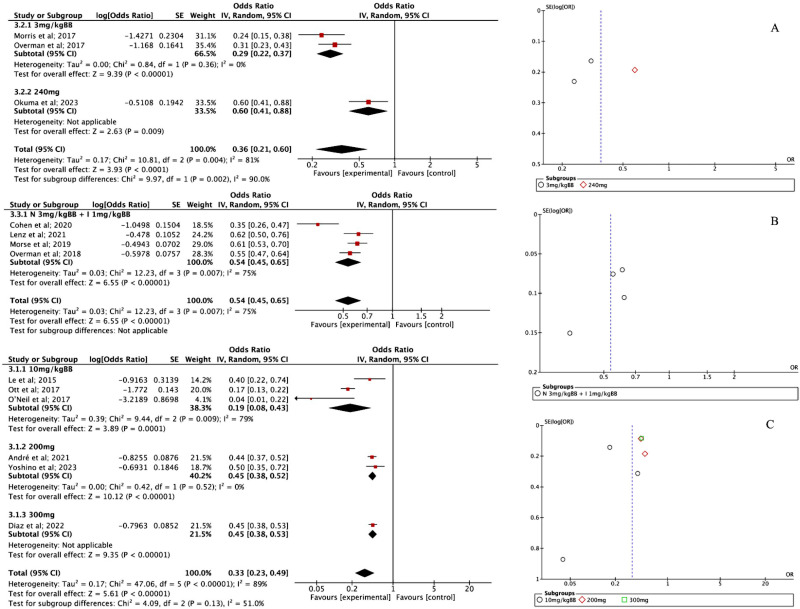
(A) Forest plot of ORR NIV intervention and Funnel plot of ORR NIV intervention; (B) Forest plot of ORR NIV + IPI intervention and Funnel plot of ORR NIV + IPI intervention; (C) Forest plot of ORR PEM intervention and Funnel plot of ORR PEM intervention.

Three studies that used NIV reported an ORR of 0.36 [95% CI 0.21; 0.60, P < 0.001]. The heterogeneity was quite high (I² = 81%), but interestingly, the funnel plot (Fig 3A) showed no signs of publication bias. Meanwhile, four studies evaluating NIV combined with Ipilimumab found a higher ORR of 0.54 [95% CI 0.45; 0.65, P < 0.001], with moderate heterogeneity (I² = 75%). The funnel plot for this combination (Fig 3B) did suggest some true heterogeneity, indicating variations in study outcomes. PEM was assessed in six studies, showing an ORR of 0.33 [95% CI 0.23; 0.49, P < 0.001]. However, heterogeneity was very high (I² = 94.6%), and the corresponding funnel plot (Fig 3C) confirmed evidence of variability among the studies.

A closer look at PEM’s effects across different dosages revealed some interesting insights. For the 10 mg/kg dosage, three studies reported an ORR of 0.19 [95% CI 0.15; 0.24], with a strong overall effect (Z = 12.90, P < 0.00001). However, heterogeneity remained high (I² = 79%), meaning there was substantial variation among the studies. In contrast, the 200 mg dosage group, based on two studies, showed a much higher ORR of 0.45 [95% CI 0.38; 0.52, P < 0.001], with almost no heterogeneity (I² = 0%), suggesting more consistent findings. The effect was also statistically significant (Z = 10.12, P < 0.00001), supporting the effectiveness of PEM at this dose. Finally, for the 300 mg dosage, only one study was available, which reported an ORR of 0.45 [95% CI 0.38; 0.53]. Since it was a single study, heterogeneity couldn’t be assessed, but the effect remained significant (Z = 9.35, P < 0.00001).

### Overall survival

**Fig 4:**
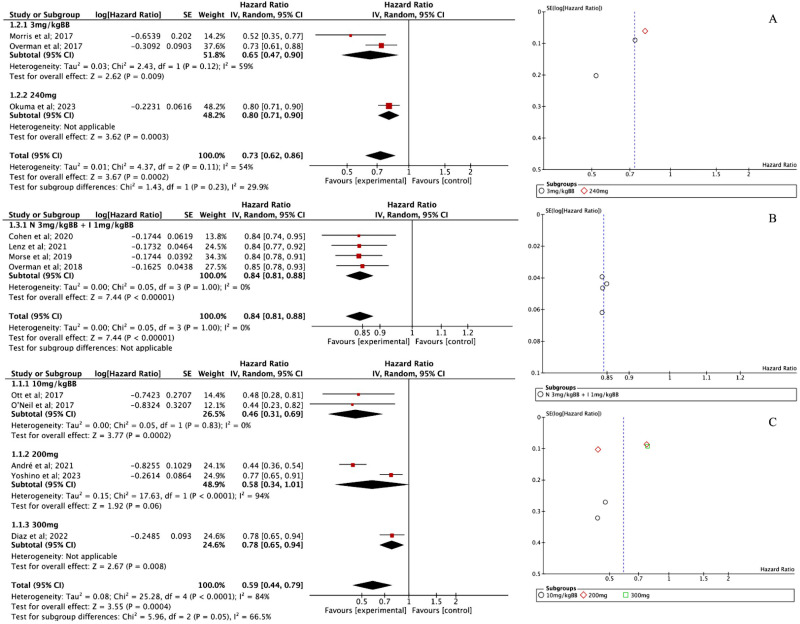
(A) Forest plot of OS NIV intervention and Funnel plot of OS NIV intervention; (B) Forest plot of OS NIV + IPI intervention and Funnel plot of OS NIV plus Ipilimumab intervention; (C) Forest plot of OS PEM intervention and Funnel plot of OS PEM intervention.

Three studies evaluating NIV reported an OS effect size of 0.73 [95% CI 0.62; 0.86, P < 0.001]. The heterogeneity was moderate (I² = 54%), and the funnel plot (Fig 4A) showed no signs of publication bias, suggesting consistency in the results. Looking at the dosage breakdown, the 3 mg/kg group had a lower OS of 0.65 [95% CI 0.47; 0.90], while the 240 mg group showed a higher OS of 0.80 [95% CI 0.71; 0.90, P < 0.001], indicating better survival outcomes with the higher dose.

For NIV + IPI, four studies reported an OS effect size of 0.84 [95% CI 0.81; 0.88, P < 0.001]. Interestingly, there was no heterogeneity (I² = 0%), meaning the results were highly consistent across studies. The funnel plot (Fig 4B) also confirmed that there was no evidence of true heterogeneity.

PEM, on the other hand, showed a more varied impact on OS. Five studies reported an OS effect size of 0.45 [95% CI 0.31; 0.66, P < 0.001], but heterogeneity was high. The funnel plot (Fig 4C) further confirmed evidence of variability between studies. When breaking it down by dosage, the 10 mg/kg subgroup, based on two studies, had an OS of 0.46 [95% CI 0.31; 0.69] with no heterogeneity (I² = 0%), and the combined effect was significant (Z = 3.77, P = 0.0002), highlighting a clear benefit of the treatment. In contrast, the 200 mg group (also two studies) showed an OS of 0.58 [95% CI 0.34; 1.01], but heterogeneity was substantial (I² = 94%). Although the effect wasn’t statistically significant at the 5% level (Z = 1.92, P = 0.06), the trend still suggested a potential survival benefit. Meanwhile, for the 300 mg dosage, only one study was available, reporting an OS of 0.78 [95% CI 0.65; 0.94]. The effect was significant (Z = 2.67, P = 0.008), but heterogeneity could not be assessed since it was based on a single study.

### Progression-free survival

**Fig 5:**
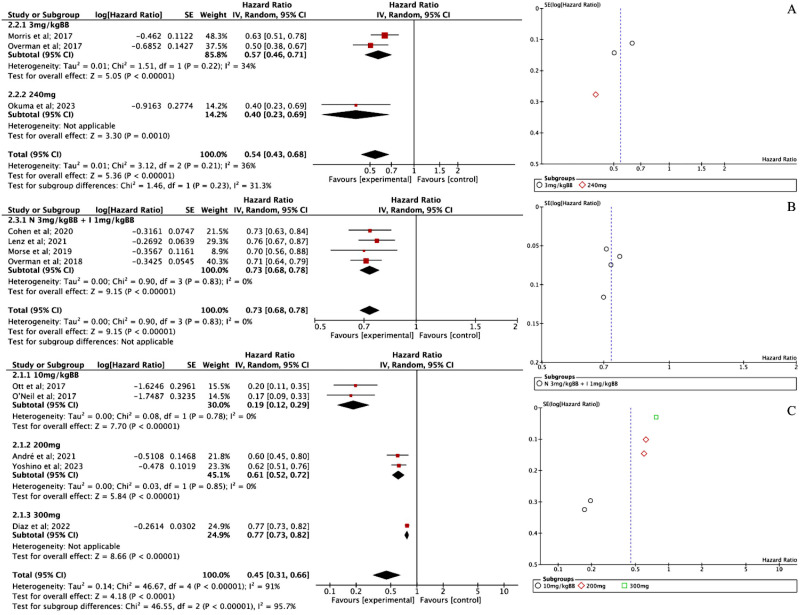
(A) Forest plot of PFS NIV intervention and Funnel plot of PFS NIV intervention; (B) Forest plot of OS NIV + IPI intervention and (C) Funnel plot of OS NIV + IPI intervention; (C) Forest plot of PFS PEM intervention and Funnel plot of PFS PEM intervention.

Three studies evaluating NIV reported a PFS effect size of 0.54 [95% CI 0.43; 0.68, P < 0.001]. Heterogeneity was low, and the funnel plot (Fig 5A) showed no signs of bias, suggesting that the results are reliable. When looking at different dosages, the 3 mg/kg subgroup—based on two studies—reported a PFS of 0.57 [95% CI 0.46–0.71] with low heterogeneity (I² = 34%), indicating relatively consistent findings. In contrast, the 240 mg subgroup, represented by only one study, reported a lower PFS of 0.40 [95% CI 0.23–0.69], suggesting a significant reduction in risk. However, since this result is based on a single study, its generalizability is limited and should be interpreted with caution.

For the combination of NIV + IPI, four studies reported a PFS effect size of 0.73 [95% CI 0.68; 0.78, P < 0.001]. There was no heterogeneity (I² = 0%), and the funnel plot (Fig 5B) confirmed consistent results across all studies, reinforcing the effectiveness of this combination therapy.

PEM, however, showed more variation across studies. Five studies reported a PFS effect size of 0.45 [95% CI 0.31; 0.66, P < 0.001], but heterogeneity was high, suggesting differences in study outcomes. The funnel plot (Fig 5C) indicated true heterogeneity, meaning factors such as study design or patient characteristics may have influenced the results. Looking at dosage-based analysis, the 10 mg/kg subgroup (based on two studies) had a PFS of 0.19 [95% CI 0.12–0.29], showing a significant risk reduction with no observed heterogeneity (I² = 0%). The overall effect was highly significant (Z = 7.70, p < 0.00001), further supporting the effectiveness of this dosage. The 200 mg subgroup, also based on two studies, showed a pooled PFS of 0.61 [95% CI 0.52–0.72], with no significant heterogeneity (I² = 0%) and a strong overall effect (Z = 5.84, p < 0.00001), reinforcing its efficacy. Lastly, the 300 mg subgroup, represented by a single study (Diaz et al., 2022), reported a PFS of 0.77 [95% CI 0.73–0.82], showing a significant risk reduction (Z = 8.66, p < 0.00001). However, since it was based on only one study, heterogeneity couldn’t be assessed, meaning these results should be interpreted cautiously.

### Disease control rate

**Fig 6:**
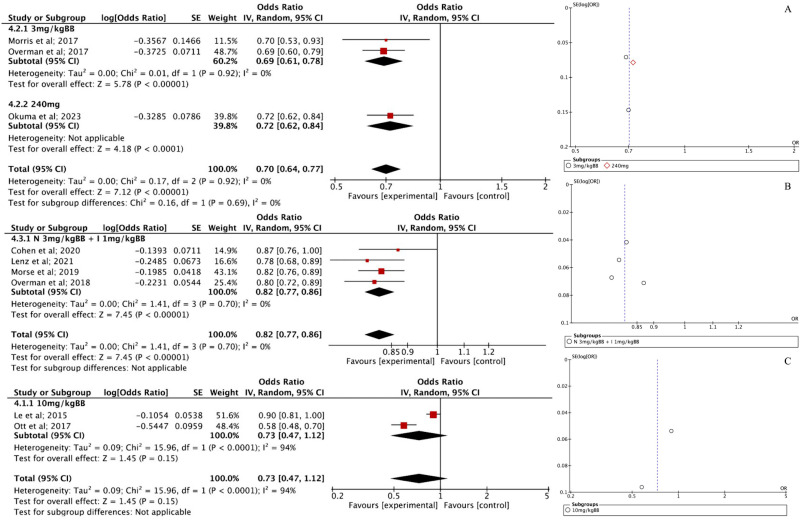
(A) Forest plot of DCR NIV intervention and Funnel plot of DCR NIV intervention; (B) Forest plot of DCR PEM intervention and Funnel plot of DCR PEM intervention; (C) Forest plot of DCR NIV and PEM intervention; (B) Funnel plot of DCR NIV and PEM intervention.

Three studies evaluating NIV reported a DCR with an effect size of 0.70 [95% CI: 0.64–0.77, p < 0.00001]. There was no heterogeneity among the studies (I² = 0%), and the funnel plot (Fig 6A) showed no evidence of bias, suggesting that the results are consistent and reliable. Looking at specific dosages, the 3 mg/kg subgroup—based on two studies—reported a combined odds ratio (OR) of 0.69 [95% CI: 0.61–0.78, p < 0.00001], showing a favorable outcome with no heterogeneity, meaning the findings were highly consistent. For the 240 mg dosage, one study reported an OR of 0.72 [95% CI: 0.62–0.84, p < 0.0001], showing a similar benefit. The absence of heterogeneity across all these studies suggests that the observed effects were consistent, regardless of dosage or study differences.

For PEM at a 10 mg/kg dosage, the meta-analysis included two studies, with a combined OR of 0.73 [95% CI: 0.47–1.12, p = 0.15]. Since the confidence interval crosses 1, this result was not statistically significant, indicating that PEM may not have a meaningful impact on DCR at this dosage. Additionally, substantial heterogeneity (I² = 94%, p < 0.0001) suggests a high degree of variability between studies, possibly due to differences in study design, patient populations, or other influencing factors. The corresponding funnel plot (Fig 6B) confirms this variability.

In contrast, when NIV (3 mg/kg) and PEM (1 mg/kg) were used together, four studies were included in the meta-analysis, yielding a pooled OR of 0.82 [95% CI: 0.77–0.86, p < 0.00001]. This indicates a statistically significant reduction in the odds of the outcome in favor of the combination therapy compared to the control. The confidence interval does not cross 1, reinforcing the reliability of this result. Heterogeneity was low (I² = 0%, p = 0.70), meaning the findings were highly consistent across studies. The effect size of each group is summarized in Table 3 below.

**Table 3:** Size effects comparison of each group.

Intervention	Effect Size (95% CI)			
	ORR	OS	PFS	DCR
PEM	0.33 (0.23; 0.49)	0.59 (0.44; 0.79)	0.45 (0.31; 0.66)	0.73 (0.47; 1.12)
NIV	0.36 (0.21; 0.60)	0.73 (0.62; 0.86)	0.54 (0.43; 0.68)	0.70 (0.64; 0.77)
NIV + IPI	0.54 (0.45; 0.65)	0.84 (0.81; 0.88)	0.73 (0.68; 0.78)	0.82 (0.77; 0.86)

## Discussion

In this study, we aimed to evaluate the effectiveness of immunotherapy drugs in CRC, considering the increasing incidence of the disease and the growing interest in immunotherapy as a treatment option. Our primary focus was to assess the overall impact of these drugs on patient outcomes. This study conducts a comprehensive analysis, incorporating data from 13 studies, encompassing a sample size of 1337 patients. The assessment of Efficacy (ORR, OS, PFS, and DCR) for each group with the investigation of AEs and survival can validate the safety of this immunotherapy. However, AEs will only be covered descriptively in this study due to the different ways in which they have been presented in previous studies. The combination of NIV + IPI demonstrates the highest efficacy. PEM and NIV monotherapy also shows promising results, with their respective advantages and disadvantages based on the analysis of this study. However, the heterogeneity observed in some of the outcomes highlights the need for further studies to confirm these findings.

The combination of NIV + IPI has been shown to be effective in various cancers, including colorectal carcinoma. The combination of these two medicines may enhance the immune response against cancer cells by targeting different checkpoints in the immune system. The high efficacy observed in our meta-analysis supports the use of this combination in mCRC patients [20]. PEM, a PD-1 inhibitor, has been approved for the treatment of mCRC patients with dMMR or MSI-H tumors. The promising results observed in our meta-analysis suggest that PEM may be a viable treatment option for a broader range of mCRC patients. Further research is needed to confirm these findings and to explore the optimal treatment strategies for mCRC patients [15].

### Treatment outcome

Immunotherapy stands as a pivotal cornerstone in tumor control, and the exploration of combination immunotherapy involving multiple agents has emerged as a promising strategy. This comprehensive approach strategically intervenes in diverse immune response processes, encompassing chemoradiotherapy, targeted therapy, and interactions with immune pathways. By orchestrating a multifaceted anti-tumor immune response, combination immunotherapy seeks to mitigate the risk of drug resistance, marking it as a highly promising avenue for investigation [21].

Among the various combinations being studied, the concurrent administration of anti-PD-1 (NIV) anti-CTLA-4 (IPI) has exhibited notable efficacy in tumor treatment [22,23]. This combination has demonstrated positive outcomes and substantial improvements in patients with metastatic melanoma, advanced renal cell carcinoma (RCC), and mCRC [23]. Hellmann et al. reported NIV + IPI efficacy in small cell lung cancer (SCLC) treatment. In those with a high tumor mutation burden, the response rate was 46.2% with the combination of NIV + IPI versus 21.3% with NIV monotherapy. Similarly, for patients with medium and low tumor mutation burdens, the combination outperformed monotherapy (16.0% versus 6.8% and 22.2% versus 4.8%, respectively), highlighting the added benefit of dual checkpoint inhibition [24]. Ready et al. provided evidence supporting the effectiveness and tolerability of NIV combined with low-dose Ipilimumab as a first-line treatment for advanced or metastatic non-small cell lung cancer (NSCLC). The ORR was higher with the combination therapy (21.9%) compared to NIV monotherapy (11.6%), indicating its superior efficacy. However, toxicities were more frequent with the combination, and treatment-related deaths were slightly higher (3 cases versus 1 case with monotherapy). These findings highlight the need for further investigation into the safety and toxicity profile of combination therapy to optimize its clinical application [25].

NIV + IPI has also shown promising outcomes, particularly in advanced RCC. Shao’s et al. conditional survival analysis shows that NIV + IPI treatment resulted in a notable increase in one-year PFS, compared to sunitinib. Specifically, PFS improved by 13% at 0.5 years (rising from 70.0% to 83.0%) and by 12% at 0.75 years (from 71.0% to 83.0%), which are both considerably higher than the 8% improvement observed in one-year OS (from 72.3% to 80.3%) [26]. This accumulating evidence underscores the potential synergistic effects of combining immunotherapeutic agents, notably anti-PD-1 and anti-CTLA-4, in conjunction with PEM, opening new avenues for the advancement of cancer treatment modalities.

NIV + IPI and NIV monotherapy have been evaluated in earlier meta-analyses conducted across multiple publications with varying cancer types. It has been reported that the combination outperforms NIV monotherapy in terms of ORR and DCR, where the treatment resulted in a significant improvement in the ORR of 1.40 (95% CI: 1.27–1.54) and PFS of 0.83 (95% CI: 0.77–0.90), but it did not significantly enhance OS (0.93 [95% CI, 0.84–1.03]) [27]. NIV + IPI also has been consistently shown to enhance ORR in advance cancer across multiple meta-analyses [28–30]. NIV + IPI use, however, still requires cautious consideration because of the possibility of excessive adverse effects. Therefore, more thorough research is required to determine how each type of cancer is affected by the combination of NIV + IPI [31].

In the realm of combination immunotherapy, the inclusion of PEM has further broadened the horizon of treatment modalities. PEM, an anti-PD-1 agent, has demonstrated its efficacy and safety profile and sustained antitumor effects across a variety of solid tumors, with minimal effective dose that achieved its full antitumor potential was 2 mg/kg administered every three weeks [32,33]. PEM combination with Ipilimumab has also demonstrated antitumor activity, achieving a median PFS of 4.1 months (95% CI: 1.4–5.8] and a median OS of 10.9 months (95% CI: 6.1–23.7), though it was also linked to significant toxicity [34].

### Adverse events

The AEs found according to previous meta-analyses indicate a high incidence of AEs in the gastrointestinal system. However, in contrast, with the endocrine system, the use of anti-CTLA-4 or anti-PD-1 immunotherapy is reported to have a greater potential to cause endocrine issues (such as hypothyroidism, hyperthyroidism, thyroiditis, and adrenal insufficiency) in other cancers [35,36]. These findings are also in line with the study conducted by Almutairi AR, et al., 2020, who stated that Healthcare providers must remain vigilant in monitoring for irAEs after combined therapeutic interventions [37].

Additionally, particular attention should be directed towards gastrointestinal and cutaneous irAEs in the context of ipilimumab therapy. Furthermore, clinicians should be cognizant of the potential occurrence of irAEs associated with hyperglycemia, thyroid dysfunction, hepatic abnormalities, and musculoskeletal disorders following administration of NIV and PEM [37]. However, this treatment strategy is still preferable compared to conventional chemotherapy since it has better efficacy with the same AEs as stated by O’bryne K, et al., 2023, There was no statistically significant difference in the occurrence of AEs necessitating discontinuation between the combination of NIV + IPI and PEM combined with chemotherapy, as well as between NIV + IPI and PEM monotherapy. However, it is noteworthy that the incidence of treatment-related grade ≥3 AEs was higher with NIV + IPI compared to PEM monotherapy (OR = 2.21 [95%CI: 1.30–3.75]) [30].

### Strength and limitation

To our knowledge, this study represents the first systematic review and meta-analysis that compares both short-term and long-term effects of presently available anti-CTLA-4 and anti-PD-1 combination or monotherapies for treating patients with CRC. However, this study does have some limitations. Due to variations in the presentation of AEs across studies, with one individual potentially experiencing two or more AEs simultaneously, extracting data posed challenges for us. Additionally, it can be quite challenging to classify an AE as a potential irAE, as this determination may vary among trials or observers. This variability could introduce detection bias, particularly because our analysis included both open-label and double-blind trials. Such variations might result in an overestimation of the incidence of potential irAEs. Nevertheless, we made efforts to mitigate this by only including patients treated with either anti-CTLA-4 or anti-PD-1 monotherapies or combination therapies, thereby excluding AEs that could be attributed to other treatments or immunotherapies. Moreover, significant statistical heterogeneity observed in most analyses is a crucial consideration. Variations in study designs, tumor stage, prior treatment history, or patient demographics among the included studies could have contributed to this heterogeneity. In addition, the significance of MSI-H/dMMR is emphasized as a key determinant influencing the effectiveness of immunotherapy in patients with mCRC.

However, by including patients regardless of their MSI-H/dMMR status, the study may dilute the significance of its findings related to immunotherapy response. Since MSI-H/dMMR status is a critical factor affecting treatment efficacy, not limiting the study to those patients may lead to conclusions that do not accurately represent the dynamics of immunotherapy response within the relevant population. Furthermore, including a broader patient population introduces variability that could confound the results. Differences in treatment response among patients with different biomarker statuses could obscure the effects of immunotherapy, making it difficult to draw meaningful conclusions.

## Conclusion

In conclusion, the exploration of combination immunotherapy involving anti-PD-1 and anti-CTLA-4 agents presents a promising avenue in cancer treatment, demonstrating notable efficacy across various cancers, including CRC. The concurrent use of NIV + IPI, particularly, showcased superior efficacy in terms of ORR, OS, and PFS. PEM monotherapy also exhibited promising results, highlighting its efficacy in treating CRC.

While the study, encompassing 13 studies with 1337 patients, provides valuable insights into the effectiveness and safety of immunotherapy in CRC, the identified heterogeneity in some outcomes emphasizes the necessity for additional research and exploration. This study represents a pioneering systematic review comparing short-term and long-term effects of anti-CTLA-4 and anti-PD-1 therapies in CRC, acknowledging certain limitations and the need for ongoing investigation to refine and validate these findings.

## Supporting information

S1 File S1 Study Selection. (DOCX)

S2 File S2 Risk of Bias Assessment. (XLSX)

S1 Checklist Prisma Checklist. (DOCX)
