# Supplementary material for: The efficacy of Pembrolizumab, Ipilimumab, and Nivolumab monotherapy and combination for colorectal cancer: A systematic review and meta-analysis
Source: PLoS One. 2025 Nov 14;20(11):e0307128. doi: 10.1371/journal.pone.0307128 (PMC12617863; doi:10.1371/journal.pone.0307128)
Supplement: S1 File — (DOCX) [file pone.0307128.s001.docx]

**The Efficacy and Safety of Pembrolizumab, Ipilimumab, and Nivolumab Monoteraphy and Combination for Colorectal Cancer: A Systematic Review and Meta-Analysis**

| 1 | Le et al., 2015 | | PD-1 Blockade in Tumors with Mismatch-Repair Deficiency. | Yes | - | | |
| --- | --- | --- | --- | --- | --- | --- | --- |
| 2 | Lenz et al., 2021 | | First-Line Nivolumab Plus Low-Dose Ipilimumab for Microsatellite Instability-High/ Mismatch Repair-Deficient Metastatic Colorectal Cancer: The Phase II CheckMate 142 Study. | Yes | - | | |
| 3 | Diaz et al., 2022 | | Pembrolizumab versus chemotherapy for microsatellite instability-high or mismatch repair-deficient metastatic colorectal cancer (KEYNOTE-177): final analysis of a randomised, open-label, phase 3 study. | Yes | - | | |
| 4 | Morse et al., 2019 | | Safety of Nivolumab plus Low-Dose Ipilimumab in Previously Treated Microsatellite Instability-High/Mismatch Repair-Deficient Metastatic Colorectal Cancer. | Yes | - | | |
| 5 | Cohen et al., 2020 | | RECIST and iRECIST criteria for the evaluation of nivolumab plus ipilimumab in patients with microsatellite instability-high/mismatch repair-deficient metastatic colorectal cancer: | Yes | - | | |
| 6 | O’Neil et al., 2017 | | Safety and antitumor activity of the anti–PD-1 antibody pembrolizumab in patients with advanced colorectal carcinoma. | Yes | - | | |
| 7 | Okuma et al., 2023 | | Phase II Trial of Nivolumab in Metastatic Rare Cancer with dMMR or MSI-H and Relation with Immune Phenotypic Analysis (the ROCK Trial). | Yes | - | | |
| 8 | Yoshino et al., 2023 | | Pembrolizumab in Asian patients with microsatellite-instability-high/mismatch-repair-deficient colorectal cancer. | Yes |  | | |
| 9 | Overman et al., 2017 | | Nivolumab in patients with metastatic DNA mismatch repair-deficient or microsatellite instability-high colorectal cancer (CheckMate 142): an open-label, multicentre, phase 2 study. | Yes | - | | |
| 10 | Ott et al., 2017 | | Safety and antitumor activity of the anti-PD-1 antibody pembrolizumab in patients with recurrent carcinoma of the anal canal. | Yes | - | | |
| 11 | Morris et al., 2017 | | Nivolumab for previously treated unresectable metastatic anal cancer (NCI9673): a multicentre, single-arm, phase 2 study. | Yes | - | | |
| 12 | Overman et al., 2018 | | Durable Clinical Benefit With Nivolumab Plus Ipilimumab in DNA Mismatch Repair-Deficient/Microsatellite Instability-High Metastatic Colorectal Cancer. | Yes | - | | |
| 13 | Andre et al., 2020 | |  | Yes | - | | |
| 14 | Ready et al., 2020 | | Nivolumab Monotherapy and Nivolumab Plus Ipilimumab in Recurrent Small Cell Lung Cancer: Results From the CheckMate 032 Randomized Cohort. | No | Different outcomes | | |
| 15 | Patnaik et al., 2015 | | Phase I study of pembrolizumab (MK-3475; Anti-PD-1 monoclonal antibody) in patients with advanced solid tumors. | No | Different outcomes | | |
| 16 | Venderbosch et al., 2024 | | Mismatch repair status and BRAF mutation status in metastatic colorectal cancer patients: A pooled analysis of the CAIRO, CAIRO2, COIN, and FOCUS studies. | No | Different outcomes | | |
| 17 | Xu et al., 2021 | | The efficacy and safety of Nivolumab combined with Ipilimumab in the immunotherapy of cancer: a meta-analysis. | No | Different outcomes | | |
| 18 | O’Byrne et al., 2023 | | Long-term comparative efficacy and safety of nivolumab plus ipilimumab relative to other first-line therapies for advanced non-small-cell lung cancer: A systematic literature review and network meta-analysis. | No | Different outcomes | | |
| 19 | Cruz et al., 2022 | | Predictive and therapeutic implications of a novel PLCγ1/SHP2-driven mechanism of cetuximab resistance in metastatic colorectal cancer. | No | Different outcomes | | |
| 20 | Shao et al., 2019 | | Conditional survival in patients with advanced renal cell carcinoma treated with nivolumab. | No | Different outcomes | | |
| 21 | Serritella et al., 2023 | | Nivolumab Plus Ipilimumab vs Nivolumab Alone in Advanced Cancers Other Than Melanoma: A Meta-Analysis. | No | Different outcomes | | |
| 22 | Jiang et al., 2021 | | Nivolumab monotherapy or combination therapy with ipilimumab for lung cancer: a systemic review and meta-analysis. | No | Different outcomes | | |
| 23 | Ros et al., 2023 | | Advances in immune checkpoint inhibitor combination strategies for microsatellite stable colorectal cancer. | No | Different outcomes | | |
| 24 | Boutros et al., 2016 | | Safety profiles of anti-CTLA-4 and anti-PD-1 antibodies alone and in combination. | No | Different outcomes | | |
| 25 | Gomar et al., 2021 | | Durable complete response to pembrolizumab in microsatellite stable colorectal cancer. | No | Different outcomes | | |
| 26 | Ghiringhelli et al., 2019 | | Is There a Place for Immunotherapy for Metastatic Microsatellite Stable Colorectal Cancer? | No | Different outcomes | | |
| 27 | Mettu et al., 2022 | | Assessment of capecitabine and bevacizumab with or without 28atezolizumab for the treatment of refractory metastatic co29lorectal cancer: A randomized clinical trial. | No | Different outcomes | | |
| 28 | Hellmann et al., 2018 | | Tum30or Mutational Burden and Efficacy of Nivolumab Monotherapy and in Combination with Ipilimumab in Small-Cell Lung Cancer. | No | Different outcomes | | |
| 29 | Chen et al., 2020 | | Effect of combined immune checkpoint inhibition vs best supportive care alone in patients with advanced colorectal cancer: the canadian cancer trials group CO.26 study. | No | Different outcomes | | |
| 30 | Almutairi et al., 2020 | | Potential Immune-Related Adverse Events Associated With Monotherapy and Combination Therapy of Ipilimumab, Nivolumab, and Pembrolizumab for Advanced Melanoma: A Systematic Review and Meta-Analysis | No | Different outcomes | | |
| 31 | Fan et al., 2021 | | Immunotherapy in colorectal cancer: Current achievements and future perspective. | No | Different outcomes | | |
| 32 | Heinemann et al., 2018 | | Somatic DNA mutations, tumor mutational burden (TMB), and MSI status: Association with efficacy in patients (pts) with metastatic colorectal cancer (mCRC) of FIRE-3 (AIO KRK-0306) | No | Different outcomes | | |
| 33 | Ree et al., 2024 | | First-line oxaliplatin-based chemotherapy and nivolumab for metastatic microsatellite-stable colorectal cancer-the randomised METIMMOX trial. | No | Different outcomes | | |
| 34 | Le et al., 2017 | | Mismatch repair deficiency predicts response of solid tumors to PD-1 blockade. | No | Different outcomes | | |
| 35 | Ando et al., 2020 | | Nivolumab plus ipilimumab versus existing immunotherapies in patients with PD-L1-positive advanced non-small cell lung cancer: A systematic review and network meta-analysis. | No | Different outcomes | | |
| 36 | Marginean et al., 2018 | | Is there a role for programmed death ligand-1 testing and immunotherapy in colorectal cancer with microsatellite instability?: Part II-the challenge of programmed death ligand-1 testing and its role in microsatellite instability-high colorectal cancer. | No | Different outcomes | | |
| 37 | Hargadon et al., 2018 | | Immune checkpoint blockade therapy for cancer: An overview of FDA-approved immune checkpoint inhibitors. | No | Different outcomes | | |
| 38 | Antoniotti et al., 2022 | | Upfront FOLFOXIRI plus bevacizumab with or without atezolizumab in the treatment of patients with metastatic colorectal cancer (AtezoTRIBE): a multicentre, open-label, randomised, controlled, phase 2 trial. | No | Reports not retrieved | | |
| 39 | Barzi et al., 2022 | | Phase I/II study of regorafenib (rego) and pembrolizumab (pembro) in refractory microsatellite stable colorectal cancer (MSSCRC). | No | Reports not retrieved | | |
| 40 | Boukouris et al., 2022 | | Latest evidence on immune checkpoint inhibitors in metastatic colorectal cancer: A 2022 update. | No | Reports not retrieved | | |
| 41 | Di Nicolantonio et al., 2008 | | Wild-yype BRAF is required for response to panitumumab or cetuximab in metastatic colorectal cancer. | No | Reports not retrieved | | |
| 42 | De Roock et al., 2010 | | Effects of *KRAS, BRAF, NR*AS, and *PIK3CA* mutations on the efficacy of cetuximab plus chemotherapy in chemotherapy-refractory metastatic colorectal cancer: a retrospective consortium analysis. | No | Reports not retrieved | | |
| 43 | Ozawa et al., 2024 | | Pembrolizumab Effectiveness in MSI-H Endometrial Cancers | No | Irrelevant | | |
| 44 | Ree et al., 2024 | | Oxaliplatin-Based Chemotherapy + Nivolumab for MSS CRC | No | Irrelevant | | |
| 45 | Sharma et al., 2024 | | Molecular Insights into Clinical Trials for ICIs in CRC | No | Irrelevant | | |
| 46 | Silva et al., 2024 | | Recent Updates on Pembrolizumab: A Narrative Review | No | Irrelevant | | |
| 47 | Chen et al., 2024 | | Pembrolizumab Plus Binimetinib With or Without Chemotherapy for MSS/pMMR Metastatic Colorectal Cancer: Outcomes From KEYNOTE-651 Cohorts A, C, and E | No | Irrelevant | | |
| 48 | Elez et al., 2024 | | Binimetinib in combination with nivolumab or nivolumab and ipilimumab in patients with previously treated microsatellite-stable metastatic colorectal cancer with RAS mutations in an open-label phase 1b/2 study | No | Irrelevant | | |
| 49 | Miao et al., 2024 | | Literature review Analysis of the Current State of Targeted Therapy for Gastrointestinal Cancers | No | Irrelevant | | |
| 50 | Chen et al., 2024 | | Tackling Challenges in Assessing the Economic Value of Tumor-Agnostic Therapies: A Cost-Effectiveness Analysis of Pembrolizumab as a Case Study | No | Irrelevant | | |
| 51 | Kim et al., 2024 | | Atezolizumab plus modified docetaxel, cisplatin, and fluorouracil as first-line treatment for advanced anal cancer (SCARCE C17-02 PRODIGE 60) | No | Irrelevant | | |
| 52 | Voisin et al., 2024 | | Xevinapant Combined with Pembrolizumab in Patients with Advanced, Pretreated, Colorectal and Pancreatic Cancer | No | Irrelevant | | |
| 53 | Shiraishi et al., 2024 | | History and Perspective of Chemotherapy in Advanced Esophageal Cancer | No | Irrelevant | | |
| 54 | Huang et al., 2024 | | Selective activation of IFNgamma-ipilimumab enhances the therapeutic effect and safety of ipilimumab | No | Irrelevant | | |
| 55 | Lenz et al., 2024 | | Modified FOLFOX6 plus bevacizumab with and without nivolumab for first-line treatment of metastatic colorectal cancer | No | Irrelevant | | |
| 56 | Kim et al., 2024 | | Phase II study of nivolumab in patients with genetic alterations in DNA damage repair | No | Irrelevant | | |
| 57 | Khushalani et al., 2024 | | Final results of urelumab in combination with cetuximab or nivolumab in advanced solid tumors | No | Irrelevant | | |
| 58 | Boustani et al., 2023 | | Practice-changing clinical trials in radiation oncology for gastrointestinal malignancies | No | Irrelevant | | |
| 59 | Xiang et al., 2024 | | A case of dMMR/MSI-H/TMB-H colon cancer with brain metastasis treated with PD-1 monoclonal antibody | No | - | | |
| 60 | Matsumoto et al., 2024 | | Conversion therapy with pembrolizumab for a peritoneal metastasis of rectal cancer causing hydronephrosis in a patient with Lynch syndrome | No | - | | |
| 61 | Boiarsky et al., 2024 | | A Panel-Based Mutational Signature of Mismatch Repair Deficiency and Pembrolizumab Response in Prostate Cancer | No | Irrelevant | | |
| 62 | Pan et al., 2024 | | Neoadjuvant Immunotherapy With Ipilimumab Plus Nivolumab in MMR-deficient Colorectal Cancer | No | - | | |
| 63 | Takei et al., 2024 | | Multiomic molecular characterization of the response to combination immunotherapy in MSS/pMMR metastatic colorectal cancer | No | Irrelevant | | |
| 64 | Armstrong et al., 2024 | | CXCR2 antagonist navarixin in combination with pembrolizumab in select advanced solid tumors | No | Irrelevant | | |
| 65 | Nishizawa et al., 2023 | | A Case of Pathological Complete Response for MSI-High Unresectable Transverse Colon Cancer Treated with Nivolumab + Ipilimumab | No | - | | |
| 66 | Tsumuraya et al., 2023 | | A Case of MSI-High Sigmoid Colon Cancer with Long-Term Survival Achieved by Pembrolizumab | No | - | | |
| 67 | Pastorino et al., 2024 | | Cross-trial comparisons for the adjuvant treatment of MSI colorectal cancer | No | Irrelevant | | |
| 68 | Yu et al., 2024 | | Progress in the treatment of anal cancer: latest investigational drugs | No | Irrelevant | | |
| 69 | Huffman et al., 2024 | | Biomarkers of pembrolizumab efficacy in advanced anal squamous cell carcinoma | No | Irrelevant | | |
| 70 | Chiba et al., 2024 | | Lenvatinib + Pembrolizumab for Advanced Endometrial Cancer: Insights from ProMisE Classification | No | Irrelevant | | |
| 71 | Coffman-D'Annibale et al., 2024 | | VB-111 (ofranergene obadenovec) in combination with nivolumab in MSS colorectal liver metastases | No | Irrelevant | | |
| 72 | Hong et al., 2024 | | Use of pembrolizumab among older adults in the US before and after tumor-agnostic approval | No | Irrelevant | | |
| 73 | Miao et al., 2024 | | RNA Expression-Based Analysis for Predicting Response to Regorafenib + Nivolumab in pMMR Colorectal Cancer | No | Irrelevant | | |
| 74 | Kageyama et al., 2023 | | A Case of dMMR Transverse Colon Cancer with Complete Response to ICI in Stage IV Lung Adenocarcinoma | No | Irrelevant | | |
| 75 | Fujii et al., 2023 | | Recurrent Colon Cancer Successfully Treated by Nivolumab + Ipilimumab to Prevent Skin Disorders | No | Irrelevant | | |
| 76 | Wilbur et al., 2024 | | Immunotherapy of MSI Cancer: Facts and Hopes | No | Irrelevant | | |
| 77 | Kassab et al., 2023 | | Immune checkpoint inhibitors in BRAF-mutated advanced colorectal cancer | No | Irrelevant | | |
| 78 | Kleef et al., 2023 | | Comparison of immunomodulatory treatments and conventional therapies in gastrointestinal cancer | No | Irrelevant | | |
| 79 | Fakih et al., 2024 | | Immunotherapy response in microsatellite stable colorectal cancer is influenced by site of metastases | No | Irrelevant | | |
| 80 | Saberzadeh-Ardestani et al., 2024 | | Metastatic site and clinical outcome of dMMR colorectal cancer treated with ICI | No | Irrelevant | | |
| 81 | Valentí et al., 2023 | | Variation of Plasma Damage-Associated Molecular Patterns in Advanced Solid Tumors | No | Irrelevant | | |
| 82 | Preti et al., 2023 | | Complete Pathologic Response of Two Colonic Primaries with MLH1 Mutation to Neoadjuvant Pembrolizumab | No | Irrelevant | | |
| 83 | Guo et al., 2023 | | Durable Response to Neoadjuvant and Adjuvant Immunotherapy in Muir-Torre Syndrome | No | Irrelevant | | |
| 84 | Kato et al., 2024 | | Complete Pathologic Response After Hepatectomy Following Nivolumab + Ipilimumab in MSI-H Colon Cancer | No | Irrelevant | | |
| 85 | Brooksbank et al., 2023 | | Emerging Biomarkers of Resistance to Immune Checkpoint Inhibition in dMMR Cancer | No | Irrelevant | | |
| 86 | Akce et al., 2023 | | Phase II Trial of Nivolumab + Metformin in Treatment-Refractory MSS Colorectal Cancer | No | Irrelevant | | |
| 87 | Zhu et al., 2023 | | Cost-effectiveness Analysis of Pembrolizumab vs Chemotherapy for dMMR/MSI-H CRC in China | No | Irrelevant | | |
| 88 | Okuma et al., 2023 | | Phase II Trial of Nivolumab in Metastatic Rare Cancer with dMMR/MSI-H | No | Irrelevant | | |
| 89 | Elez et al., 2024 | | SEAMARK: First-line Encorafenib + Cetuximab + Pembrolizumab for MSI-H/dMMR BRAFV600E-mutant CRC | No | Irrelevant | | |
| 90 | Oliveres et al., 2023 | | Metabolic Interventions to Enhance Immunotherapy in Advanced Colorectal Cancer | No | Irrelevant | | |
| 91 | Arima et al., 2023 | | Microsatellite Instability-High Signet Ring Cell Carcinoma of the Colon Treated With Immunotherapy | No | Irrelevant | | |
| 92 | André et al., 2023 | | KEYSTEP-008: Pembrolizumab-Based Combination in MSI-H/dMMR Metastatic CRC | No | Irrelevant | | |
| 93 | Oshima et al., 2023 | | ICI Therapy in Neoadjuvant and Adjuvant Treatment for Resectable Gastrointestinal Cancer | No | Irrelevant | | |
| 94 | Margalit et al., 2023 | | Combination Treatment of Vidutolimod, Radiosurgery, Nivolumab, and Ipilimumab for MSS CRC | No | Irrelevant | | |
| 95 | Coston et al., 2023 | | Efficacy of Immune Checkpoint Inhibitors in MSI-H Pancreatic Cancer: Mayo Clinic Experience | No | Irrelevant | | |
| 96 | Bennion et al., 2023 | | FcγRIIB on CD8+ T Cells Limits Response to PD-1 Checkpoint Inhibition | No | Irrelevant | | |
| 97 | Chakrabarti et al., 2023 | | Systematic Review on Neoadjuvant ICI for Early-Stage dMMR CRC | No | - | | |
| 98 | Krekeler et al., 2023 | | Complete Metabolic Response to Combined ICI After Pembrolizumab Failure in mCRC | No | - | | |
| 99 | Duvivier et al., 2023 | | Pembrolizumab in High Tumor Mutational Burden Tumors: TAPUR Study Results | No | Irrelevant | | |
| 100 | Veen et al., 2023 | | Clinical Trials of Neoadjuvant ICIs for Early-Stage Colon and Rectal Cancer | No | - | | |
| 101 | Sagawa et al., 2023 | | Sarcoidosis-Like Reaction with ICIs in mCRC | No | Irrelevant | | |
| 102 | Mutlu et al., 2023 | | Chemoimmunotherapy in MSI-H Goblet Cell Carcinoma | No | Irrelevant | | |
| 103 | Sokop et al., 2023 | | Effect of Pembrolizumab in MMR-D Rectal Cancer | No | - | | |
| 104 | Justesen et al., 2023 | | Neoadjuvant Pembrolizumab in MMR-D Colon Cancer | No | - | | |
| 105 | Vegivinti et al., 2023 | | Role of ICIs in MSS CRC with High TMB | No | Irrelevant | | |
| 106 | Sugiura et al., 2023 | | Cost-Effectiveness of Pembrolizumab in Japan | No | Irrelevant | | |
| 107 | Hayashi et al., 2023 | | Irinotecan and FTD/TPI After Nivolumab in Gastric Cancer | No | Irrelevant | | |
| 108 | Saúde-Conde et al., 2023 | | Immunotherapies in Non-Metastatic GI Cancers | No | Irrelevant | | |
| 109 | Hong et al., 2023 | | Metronomic Chemotherapy as Partner for ICIs in CRC | No | Irrelevant | | |
| 110 | Liu et al., 2023 | | Medullary Carcinoma of the Duodenum Treated with Pembrolizumab | No | Irrelevant | | |
| 111 | Filippi et al., 2023 | | Y-90 Radioembolization and ICIs in Hepatic Tumors | No | Irrelevant | | |
| 112 | Guo et al., 2023 | | Molecular Profiling for Targeted & Immunotherapy in CRC | No | Irrelevant | | |
| 113 | Le et al., 2023 | | Pembrolizumab in MSI-H/dMMR CRC: KEYNOTE-164 Final Analysis | No | - | | |
| 114 | Liang et al., 2023 | | KRT17 Expression and T-Cell Infiltration in CRC | No | Irrelevant | | |
| 115 | Das et al., 2023 | | Nivolumab in Pediatric High TMB & MMR-D Cancers | No | Irrelevant | | |
| 116 | Martini et al., 2023 | | Patient-Derived Organoids to Guide CRC Treatment | No | Irrelevant | | |
| 117 | Fakih et al., 2023 | | Adenovirus Therapy with Nivolumab in Epithelial Cancer | No | Irrelevant | | |
| 118 | Li et al., 2023 | | Inflammatory Signature for CRC Prognosis | No | Irrelevant | | |
| 119 | Cole et al., 2023 | | Phase 1 Trial of NEO-201 in Solid Tumors | No | Irrelevant | | |
| 120 | Peehl et al., 2023 | | Animal Models in Imaging-Assisted Co-Clinical Trials | No | Irrelevant | | |
| 121 | Zhu et al., 2023 | | Neoadjuvant ICIs in CRC: Right Patients, Right Regimens? | No | - | | |
| 122 | Karpel et al., 2023 | | Endometrial Cancer Treatments in 2023 | No | Irrelevant | | |
| 123 | Ziranu et al., 2023 | | CDX-2 Expression in MSI-H mCRC Treated with ICIs | No | Irrelevant | | |
| 124 | Kodama et al., 2023 | | Second-Line Ipi + Nivo in MSI-H Ascending Colon Cancer | No | - | | |
| 125 | Marmarelis et al., 2023 | | Pembrolizumab in Malignant Peritoneal Mesothelioma | No | Irrelevant | | |
| 126 | Fakih et al., 2023 | | Regorafenib + Ipi + Nivo in MSS CRC | No | - | | |
| 127 | Nagahisa et al., 2023 | | Pembrolizumab + Pelvic Radiotherapy for Bladder Cancer with Rectal Mets | No | Irrelevant | | |
| 128 | Karpel et al., 2023 | | Biomarker-Driven Therapy in Endometrial Cancer | No | Irrelevant | | |
| 129 | Krimmel et al., 2023 | | Immunotherapy in Hepatogastroenterology | No | Irrelevant | | |
| 130 | Sahin et al., 2023 | | Neoadjuvant ICI in MSI-H CRC | No | - | | |
| 131 | Giuliani et al., 2023 | | Cost-Effectiveness of Pembrolizumab in First-Line for MSI-H/dMMR mCRC | No | Irrelevant | | |
| 132 | Hill et al., 2023 | | MSI, TMB, and MMR as Predictive Biomarkers in Endometrial Cancer | No | Irrelevant | | |
| 133 | Kobayashi et al., 2023 | | Pembrolizumab for MSI-H Rhabdoid Carcinoma of the Colon | No | Irrelevant | | |
| 134 | Djerroudi et al., 2023 | | MSI-High Perivascular Epithelioid Cell Tumors with Lynch Syndrome Treated with Pembrolizumab | No | Irrelevant | | |
| 135 | Arora et al., 2023 | | Need for Dual Immunotherapy in First-Line MSI-H mCRC | No | Irrelevant | | |
| 136 | Bando et al., 2023 | | Future Directions in Metastatic CRC | No | Irrelevant | | |
| 137 | Sidaway et al., 2023 | | Neoadjuvant Pembrolizumab in MSI-H/dMMR Tumors | No | - | | |
| 138 | | Lemech et al., 2023 | | | Pixatimod + Nivolumab in MSS mCRC and Pancreatic Cancer | No | Irrelevant |
| 139 | | Ludford et al., 2023 | | | Neoadjuvant Pembrolizumab in MSI-H/dMMR Solid Tumors | No | - |
| 140 | | Nasca et al., 2023 | | | Association of irAEs with Outcomes of ICIs in MSI-H mCRC | No | - |
| 141 | | Liu et al., 2023 | | | Cost-Effectiveness of Pembrolizumab vs Chemotherapy in MSI-H mCRC | No | Irrelevant |
| 142 | | Saoudi Gonzalez et al., 2022 | | | PK/PD of Monoclonal Antibodies in CRC | No | Irrelevant |
| 143 | | Garralda et al., 2022 | | | Favezelimab + Pembrolizumab in MSS CRC | No | Irrelevant |
| 144 | | Di Dio et al., 2023 | | | Immunotherapy in MMR-D and MMR-Proficient Endometrial Cancer | No | Irrelevant |
| 145 | | Sui et al., 2022 | | | Inflammation Promotes ICI Resistance in MSI-H CRC | No | Irrelevant |
| 146 | | Bureš et al., 2022 | | | GI Toxicity of Systemic Oncology Immunotherapy | No | Irrelevant |
| 147 | | He et al., 2023 | | | Regorafenib ± PD-1 Inhibitor in MSS mCRC | No | Irrelevant |
| 148 | | Yoshino et al., 2023 | | | Pembrolizumab in Asian Patients with MSI-H/dMMR mCRC | No | - |
| 149 | | El-Ghazzi et al., 2023 | | | Pembrolizumab as Second-Line Treatment in MSI-H GI Cancers | No | - |
| 150 | | Kelkar et al., 2023 | | | Real-World Outcomes of Immunotherapy in MSI-H/dMMR Endometrial Cancer | No | Irrelevant |
| 151 | | Grothey et al., 2022 | | | Pembrolizumab in Metastatic dMMR CRC | No | - |
| 152 | | Saunders et al., 2022 | | | CXD101 + Nivolumab in MSS CRC | No | Irrelevant |
| 153 | | Narayanan et al., 2022 | | | Role of CTLA-4 in Colon Cancer Diagnosis | No | Irrelevant |
| 154 | | Shimura et al., 2022 | | | Pembrolizumab Outcomes in MSI-H Patients | No | - |
| 155 | | Morris et al., 2023 | | | ASCO Guidelines for Metastatic CRC | No | Irrelevant |
| 156 | | He et al., 2022 | | | Immune Subtypes in Locally Advanced Rectal Cancer | No | Irrelevant |
| 157 | | Gerger et al., 2022 | | | BRAF V600E Mutant mCRC Treatment Pathways | No | Irrelevant |
| 158 | | Rakké et al., 2023 | | | GITR Ligation and PD-1 Blockade in MMR-Proficient CRC | No | Irrelevant |
| 159 | | Demes et al., 2022 | | | Nivolumab in HIV/HPV-Related Metastatic Anal Cancer | No | Irrelevant |
| 160 | | Rizzolo et al., 2022 | | | Ipilimumab + Nivolumab in Malignant Peritoneal Mesothelioma | No | Irrelevant |
| 161 | | Palmer et al., 2022 | | | Chimpanzee Adenovirus & mRNA Neoantigen Vaccine + Nivolumab & Ipilimumab | No | Irrelevant |
| 162 | | Serpas Higbie et al., 2022 | | | Antibiotic Exposure and ICI Response in MSI-H/dMMR mCRC | No | Irrelevant |
| 163 | | Bertaglia et al., 2022 | | | ICI + Locoregional Treatments in Solid Tumors | No | Irrelevant |
| 164 | | Sanborn et al., 2022 | | | Anti-CD27 (Varlilumab) + Nivolumab in Solid Tumors | No | Irrelevant |
| 165 | | Coutzac et al., 2022 | | | Perioperative Immunotherapy in MSI/dMMR Tumors (IMHOTEP) | No | Irrelevant |
| 166 | | Barrington et al., 2022 | | | NLR and Immunotherapy in Recurrent Endometrial Cancer | No | Irrelevant |
| 167 | | Yamazaki et al., 2022 | | | ICI Strategy & Genetic Testing for MSI-H CRC | No | Irrelevant |
| 168 | | Weng et al., 2022 | | | Exploring Immunotherapy in CRC | No | Irrelevant |
| 169 | | Corrò et al., 2022 | | | Phase II Study: Neoadjuvant Pembrolizumab + Radiotherapy in Rectal Cancer | No | Irrelevant |
| 170 | | Hsieh-Wong et al., 2022 | | | MSI-H Rectal Adenocarcinoma & Urothelial Carcinoma Treated with ICI | No | Irrelevant |
| 171 | | Zhao et al., 2022 | | | Immunotherapy in CRC: Progress & Future Directions | No | Irrelevant |
| 172 | | André et al., 2022 | | | 4-Year Follow-Up: Nivolumab + Ipilimumab in MSI-H/mCRC (CheckMate 142) | No | - |
| 173 | | Lanuza et al., 2022 | | | Adoptive NK Cell Transfer in CRC | No | Irrelevant |
| 174 | | Kubo et al., 2022 | | | MSI-H in Hepato-Biliary-Pancreatic Malignancies | No | Irrelevant |
| 175 | | Sartore-Bianchi et al., 2022 | | | Histology-Agnostic Treatments in mCRC | No | Irrelevant |
| 176 | | Hiroi et al., 2022 | | | Complete Pathologic Response to Pembrolizumab in MSI-H Colon Cancer | No | - |
| 177 | | Gatto et al., 2022 | | | Hypermutation as a Biomarker for Immunotherapy in Gliomas | No | Irrelevant |
| 178 | | Jácome et al., 2022 | | | Role of Immunotherapy in Anal Cancer | No | Irrelevant |
| 179 | | Mori et al., 2022 | | | Nivolumab in Locally Advanced Rectal Cancer | No | Irrelevant |
| 180 | | Maio et al., 2022 | | | QoL in MSI-H/dMMR Solid Tumors Treated with Pembrolizumab | No | Irrelevant |
| 181 | | Kim et al., 2022 | | | Regorafenib + Nivolumab in MMR-Proficient CRC | No | Irrelevant |
| 182 | | Crisafulli et al., 2022 | | | Temozolomide Alters MMR & Boosts Mutational Burden in CRC | No | Irrelevant |
| 183 | | Mullally et al., 2022 | | | TTP Following Ipilimumab + Nivolumab in Melanoma | No | Irrelevant |
| 184 | | Zhou et al., 2022 | | | Complete Response in Locally Advanced CRC with Nivolumab | No | - |
| 185 | | Haag et al., 2022 | | | Pembrolizumab + Maraviroc in MSS CRC (PICCASSO Trial) | No | Irrelevant |
| 186 | | Diaz et al., 2022 | | | Pembrolizumab vs. Chemo in MSI-H/mCRC (KEYNOTE-177) | No | - |
| 187 | | Zhang et al., 2022 | | | Neoadjuvant Immunotherapy in MSI-H/dMMR CRC | No | - |
| 188 | | Graham et al., 2022 | | | MMR Deficiency in Prostate Cancer | No | Irrelevant |
| 189 | | Boukouris et al., 2022 | | | Update on ICIs in mCRC | No | Irrelevant |
| 190 | | Sakata et al., 2022 | | | Targeted Therapy for CRC | No | Irrelevant |
| 191 | | Tan-Garcia et al., 2022 | | | Complete pCR Following Neoadjuvant Pembrolizumab in dMMR Colon Cancer | No | - |
| 192 | | Gong & Zhao, 2022 | | | ICI in CRC: Review of Clinical Trials | No | Irrelevant |
| 193 | | Zhang et al., 2022 | | | PD-1/PD-L1 Inhibitors in mCRC: Systematic Review & Meta-Analysis | No | Irrelevant |
| 194 | | Rahma et al., 2022 | | | Phase IB Study: Ziv-Aflibercept + Pembrolizumab in Advanced Tumors | No | Irrelevant |
| 195 | | Morano et al., 2022 | | | Temozolomide + Ipilimumab + Nivolumab in MSS CRC (MAYA Trial) | No | Irrelevant |
| 196 | | Ieranò et al., 2022 | | | Nivolumab & PD-1+ Colon Cancer Cells | No | Irrelevant |
| 197 | | Silva et al., 2022 | | | Current & Evolving Role of Immunotherapy in mCRC | No | Irrelevant |
| 198 | | Copur et al., 2022 | | | Complete pCR to Neoadjuvant Chemoimmunotherapy in Colon Cancer | No | - |
| 199 | | Liu et al., 2022 | | | ML-Based Immune-Derived lncRNA Signature in CRC | No | Irrelevant |
| 200 | | Arole et al., 2022 | | | Evaluating MMR Status for Immunotherapy in Breast Cancer | No | Irrelevant |
| 201 | | Parikh et al., 2021 | | | RT Enhances Immunotherapy in MSS CRC & PDAC | No | Irrelevant |
| 202 | | Marabelle et al., 2022 | | | Pembrolizumab in Advanced Anal SCC (KEYNOTE-158) | No | Different cancer type |
| 203 | | Kasi et al., 2022 | | | ctDNA Analysis During PD-1 Blockade in dMMR mCRC | No | Irrelevant |
| 204 | | Bai et al., 2022 | | | Tumor-Infiltrating Lymphocytes in CRC & Immunotherapy | No | Review article |
| 205 | | Jordan et al., 2022 | | | Ipilimumab Re-exposition in Heavily Pretreated MSI-H mCRC | No | Case report |
| 206 | | Bando et al., 2022 | | | Preop CRT + Nivolumab in MSS/MSI-H LARC | No | Irrelevant |
| 207 | | Fanale et al., 2022 | | | Tumor-Agnostic MSI/MMR Status in Immunotherapy | No | Review article |
| 208 | | Tonello et al., 2022 | | | pCR in Lynch Syndrome & Peritoneal Metastases | No | Case report |
| 209 | | Kuang et al., 2022 | | | Pembrolizumab + Azacitidine in Refractory mCRC | No | Irrelevant |
| 210 | | O'Malley et al., 2022 | | | Pembrolizumab in MSI-H Advanced Endometrial Cancer | No | Different cancer type |
| 211 | | Vlachostergios et al., 2021 | | | Integrin Signaling & ICI Outcomes | No | Irrelevant |
| 212 | | Dhamani et al., 2021 | | | Paradigm Shift in MSI-H mCRC with ICIs | No | Review article |
| 213 | | Ganesh, 2022 | | | Optimizing Immunotherapy for CRC | No | Review article |
| 214 | | Panhaleux et al., 2022 | | | PD-L1 ICIs in Systemic Sclerosis Patients | No | Irrelevant |
| 215 | | Martin-Romano et al., 2021 | | | Case Report on RT & Anti-PD1 in Anal SCC | No | Case report, different cancer type |
| 216 | | Pan et al., 2021 | | | Future Directions in CRC Immunotherapy | No | Review article |
| 217 | | Gallois et al., 2022 | | | Encorafenib + Cetuximab in BRAF V600E & MSI-H mCRC | No | Different treatment focus |
| 218 | | Kadono et al., 2021 | | | Pembrolizumab in Small Bowel Mucinous AdenoCa | No | Case report, different cancer type |
| 219 | | Touati & Cohen, 2022 | | | Nivolumab + Ipilimumab in MSI-H mCRC | No | Drug approval summary |
| 220 | | Srikrishna, 2022 | | | Role of Physical Activity in RegoNivo for MSS-mCRC | No | Opinion article |
| 221 | | Borozan et al., 2022 | | | Fusobacterium & CRC Outcomes | No | Different research focus |
| 222 | | Tominaga et al., 2022 | | | pCR to Pembrolizumab in MSI-H Ascending Colon Cancer | No | Case report |
| 223 | | Kanani et al., 2021 | | | Neoadjuvant Immunotherapy in CRC | No | Review article |
| 224 | | Fan et al., 2021 | | | Current & Future Perspectives in CRC Immunotherapy | No | Review article |
| 225 | | Zhou et al., 2021 | | | Hyperprogression in dMMR GI Cancer with Anti-PD1 | No | Case report, different focus |
| 226 | | Lee et al., 2021 | | | Sanguisorbae Radix + Pembrolizumab in CRC Model | No | Preclinical study |
| 227 | | Fountzilas et al., 2021 | | | Cetuximab + Pembrolizumab in RASwt mCRC | No | Different treatment focus |
| 228 | | Lenz et al., 2022 | | | Nivolumab + Ipilimumab in MSI-H mCRC (CheckMate-142) | Yes | - |
| 229 | | Tabatabai et al., 2022 | | | RAS Inhibitors in CRC Treatment | No | Different treatment focus |
| 230 | | Suarez-Carmona et al., 2021 | | | CXCL12 & PD-1 Inhibition in MSS CRC & PDAC | No | Different treatment focus |
| 231 | | Kawazoe et al., 2021 | | | TAS-116 + Nivolumab in CRC & Solid Tumors | No | Different treatment focus |
| 232 | | Graham et al., 2021 | | | Hypermutation, MMR Deficiency & Checkpoint Blockade | No | Review article |
| 233 | | Bordonaro et al., 2021 | | | Trifluridine/Tipiracil + Oxaliplatin + Nivolumab in mCRC | No | Different treatment focus |
| 234 | | Saleh et al., 2021 | | | Pembrolizumab: A New SoC in mCRC | No | Commentary article |
| 235 | | Rüschoff et al., 2021 | | | MSI Testing & ICIs in CRC | No | Review article |
| 236 | | Trojan et al., 2021 | | | pCR After Neoadjuvant Ipilimumab + Nivolumab in MSI-H RC | Yes | - |
| 237 | | Wang et al., 2021 | | | Pancreatic Cancer Progression in Lynch Syndrome | No | Different cancer type |
| 238 | | Ciner et al., 2021 | | | Delayed CRS After Neoadjuvant Nivolumab in CRC | No | Case report |
| 239 | | Gomar et al., 2021 | | | Durable Response to Pembrolizumab in MSS CRC | No | Case report |
| 240 | | Kim et al., 2021 | | | Single-Cell RNA Seq in CRC Response to CD73 + PD-1 ICI | No | Preclinical study |
| 241 | | Chen et al., 2021 | | | Hyperprogression from PD-1 Inhibitors in Pan-Cancer | No | Different cancer types |
| 242 | | Gallois et al., 2021 | | | Pembrolizumab + CAPOX + Bevacizumab in MSS CRC | No | Different treatment focus |
| 243 | | Bortolomeazzi et al., 2021 | | | Immunogenomics & Checkpoint Blockade in CRC (KEYNOTE-177) | Yes | - |
| 244 | | Rahma et al., 2021 | | | TNT with Pembrolizumab in LARC | No | Different setting |
| 245 | | Herting et al., 2021 | | | Pembrolizumab + Chemo in mCRC (Phase Ib) | No | Different treatment focus |
| 246 | | Takushima et al., 2021 | | | Cost-effectiveness of Trifluridine/Tipiracil vs Nivolumab | No | Economic analysis |
| 247 | | Kato et al., 2021 | | | Tumor Growth Rate & Response to Nivolumab in Gastric Cancer | No | Different cancer type |
| 248 | | Pecci et al., 2021 | | | Beyond MSI: Immunotherapy in MSS CRC | No | Review article |
| 249 | | Alphones et al., 2021 | | | MMR Deficiency in Pediatric HGG | No | Different cancer type |
| 250 | | Wilson et al., 2021 | | | ICI in Lower GI Cancers: Systematic Review | No | Review article |
| 251 | | Danley et al., 2021 | | | Durable Pembrolizumab Response in Uterine Serous Carcinoma | No | Different cancer type |
| 252 | | Rossi et al., 2021 | | | Pepinemab + Nivolumab/Ipilimumab in CRC & Pancreatic Cancer | No | Different treatment focus |
| 253 | | Manz et al., 2021 | | | Efficacy & Side Effects of ICI in CRC | No | Review article |
| 254 | | Jagieła et al., 2021 | | | Nephrotoxicity in CRC & Melanoma Immunotherapy | No | Review article |
| 255 | | Sherman et al., 2021 | | | Nivolumab + RT in Lynch Syndrome & Glioblastoma | No | Different cancer type |
| 256 | | Mazlom et al., 2021 | | | Management of Small Bowel Adenocarcinoma | No | Different cancer type |
| 257 | | Casak et al., 2021 | | | FDA Approval of Pembrolizumab for MSI-H mCRC | Yes | - |
| 258 | | Long et al., 2021 | | | STK11 Mutation & ICI Resistance in Lynch Syndrome | No | Different cancer type |
| 259 | | Kim et al., 2021 | | | Ibrutinib + Pembrolizumab in pMMR mCRC | No | Different treatment focus |
| 260 | | Andre et al., 2021 | | | QoL in MSI-H mCRC: Pembrolizumab vs Chemo (KEYNOTE-177) | Yes | - |
| 261 | | Rousseau et al., 2021 | | | Checkpoint Blockade in Hypermutated Tumors | No | Commentary article |
| 262 | | Pureur et al., 2021 | | | Pembrolizumab as First-line in MSI-H mCRC | No | No abstract available |
| 263 | | Park et al., 2021 | | | CU06-1004 & Immunotherapy in TME | No | Preclinical study |
| 264 | | Glode & May, 2021 | | | ICIs in NSCLC | No | Different cancer type |
| 265 | | Childers et al., 2021 | | | Checkpoint Blockade in Lynch Syndrome Pancreatic Cancer | No | Different cancer type |
| 266 | | Ghidini et al., 2021 | | | ICIs in CRC Therapy | No | Review article |
| 267 | | Patel et al., 2021 | | | Trifluridine/Tipiracil + Nivolumab in MSS mCRC | No | Different treatment focus |
| 268 | | Tan & Sahin, 2021 | | | ICIs in dMMR/MSI-H CRC | No | Review article |
| 269 | | Ishiguro et al., 2020 | | | CR to Pembrolizumab in Recurrent CRC | No | Case report |
| 270 | | Moriuchi et al., 2020 | | | Pembrolizumab in MSI-H Small Bowel Cancer | No | Different cancer type |
| 271 | | Arteaga Pérez, 2022 | | | Biomarker Discovery & Patient Work | No | Social science focus |
| 272 | | Bregni et al., 2021 | | | REGINA Trial: Regorafenib + Nivolumab in Rectal Cancer | No | Different treatment focus |
| 273 | | Akagi et al., 2021 | | | Real-world MSI Status in Solid Tumors | No | Epidemiological study |
| 274 | | Padovan et al., 2021 | | | Pembrolizumab in MMRd Oligodendroglioma | No | Different cancer type |
| 275 | | Jacobi et al., 2021 | | | MSI/MMR Testing in Cytology Specimens | No | Diagnostic study |
| 276 | | Mudd et al., 2021 | | | MS4A1 in CRC Tumor Microenvironment | No | Preclinical study |
| 277 | | Kishore & Bhadra, 2021 | | | Future of ICIs in CRC | No | Review article |
| 278 | | André et al., 2020 | | | Pembrolizumab in MSI-H CRC (KEYNOTE-177) | Yes | - |
| 279 | | Perrotta et al., 2020 | | | Polymyalgia Rheumatica After Pembrolizumab | No | Case report |
| 280 | | Hirano et al., 2021 | | | ICIs in CRC | No | Review article |
| 281 | | Cohen et al., 2020 | | | NIPICOL Study: Nivolumab + Ipilimumab in MSI-H CRC | Yes | - |
| 282 | | Anghileri et al., 2021 | | | High TMB & T-cell Activation in LS Glioblastoma | No | Different cancer type |
| 283 | | Nguyen et al., 2021 | | | Update on ICIs in CRC | No | Review article |
| 284 | | Fernandes et al., 2020 | | | New ICI Mechanism: RIPR-PD1 | No | Preclinical study |
| 285 | | Suzuki et al., 2021 | | | CR to Ipilimumab in Colon Cancer | No | Case report |
| 286 | | Plitnick et al., 2020 | | | Murine Model for Anti-PD-1 Response | No | Preclinical study |
| 287 | | Deligiorgi & Trafalis, 2020 | | | Adrenal Insufficiency from PD-1 Inhibitors | No | Review article |
| 288 | | Lu et al., 2020 | | | p50 & T-cell Regulation in Immunotherapy | No | Preclinical study |
| 289 | | van Dijk et al., 2020 | | | NABUCCO: Nivolumab + Ipilimumab in Urothelial Cancer | No | Different cancer type |
| 290 | | Razak et al., 2020 | | | AMG 820 + Pembrolizumab in Solid Tumors | No | Different treatment focus |
| 291 | | Park et al., 2020 | | | Pembrolizumab in MSI-driven Parathyroid Carcinoma | No | Different cancer type |
| 292 | | Schardt, 2020 | | | Use of ICIs in Oncology | No | Review article |
| 293 | | Ding et al., 2021 | | | Cost-Effectiveness of Pembrolizumab + Axitinib vs. Sunitinib | No | Economic analysis |
| 294 | | Eso & Seno, 2020 | | | ICIs in GI, Hepatobiliary, and Pancreatic Cancers | No | Review article |
| 295 | | Sun, 2021 | | | MSI Testing in CRC Management | No | Review article |
| 296 | | Damato et al., 2020 | | | NIVACOR: Nivolumab + FOLFOXIRI in mCRC | No | Different treatment focus |
| 297 | | Kichloo et al., 2020 | | | Pembrolizumab-Induced Diabetes Mellitus in CRC | No | Case report |
| 298 | | Wang et al., 2020 | | | IgG4 & Immune Evasion in CRC | No | Preclinical study |
| 299 | | Geva et al., 2020 | | | MK-1248 + Pembrolizumab in Advanced Solid Tumors | No | Different treatment focus |
| 300 | | Ricci et al., 2020 | | | Immunotherapy in Biliary Tract Cancer | No | Different cancer type |
| 301 | | Li et al., 2024 | | | Targeting metabolic pathway enhances CAR-T potency for solid tumor | No | Different treatment modality (CAR-T) |
| 302 | | Saha et al., 2024 | | | Unraveling complexities of colorectal cancer and its therapies | No | Broad review, lacks specific focus on immunotherapy |
| 303 | | Guan et al., 2024 | | | Immunogenic peptides from intratumor microbes in CRC | No | Preclinical study |
| 304 | | Xiao et al., 2024 | | | Circulating immune function in solid tumors | No | Focus on diagnostics rather than treatment |
| 305 | | Nishimura et al., 2024 | | | STING pathway regulation in HCC | No | Different cancer type (HCC) |
| 306 | | Cai et al., 2024 | | | Anticancer potential of marine alkaloids | No | Focus on novel drug sources, not immunotherapy |
| 307 | | Rajkumari et al., 2024 | | | Myeloid-derived suppressor cells in cancer | No | Focus on tumor microenvironment rather than treatment |
| 308 | | Seufferlein et al., 2024 | | | Palliative treatment of metastatic rectal cancer | No | Focus on palliative care, lacks immunotherapy discussion |
| 309 | | Park et al., 2024 | | | Tri-specific T-cell engager for GBM | No | Different cancer type (GBM) |
| 310 | | Liu et al., 2024 | | | Mutation-treatment effects in multiple cancers | No | Broad cancer dataset, lacks specific focus on colorectal cancer |
| 311 | | Guan et al., 2024 | | | PEA3 subfamily genes as tumor markers | No | Focus on biomarkers, not treatment |
| 312 | | Ros et al., 2024 | | | BRAF V600E-Mutated mCRC treated with Trifluridine/Tipiracil | No | Focus on chemotherapy, not immunotherapy |
| 313 | | Sun et al., 2024 | | | Second-line systemic treatment for mCRC | No | General treatment review, lacks immunotherapy focus |
| 314 | | Porro et al., 2024 | | | Cholangiocarcinoma Biology and Characterization | No | Different cancer type (cholangiocarcinoma) |
| 315 | | Aspesi et al., 2024 | | | Microbiota and early tumors in colon carcinogenesis | No | Focus on microbiome, not treatment |
| 316 | | Garrido et al., 2024 | | | LOH in HLA genes in breast cancer | No | Different cancer type (breast cancer) |
| 317 | | Zhao et al., 2024 | | | Gut microbiome and cancer immunotherapy | No | Focus on microbiome, not direct treatment |
| 318 | | Wei et al., 2024 | | | Sensitizing immune checkpoint inhibitors | No | General cancer immunotherapy, lacks colorectal focus |
| 319 | | Ohsawa et al., 2024 | | | Fusobacterium nucleatum in cancer treatment | No | Focus on microbiome, not direct treatment |
| 320 | | Chen et al., 2024 | | | SPP1+ macrophages and tumor immune evasion | No | Focus on immune mechanisms, not treatment |
| 321 | | Peng et al., 2024 | | | Blood lipid biomarkers in cancer survival | No | Focus on biomarkers, not treatment |
| 322 | | Raaijmakers et al., 2024 | | | Cancer-associated fibroblasts and radiotherapy | No | Focus on tumor microenvironment, not immunotherapy |
| 323 | | Liang et al., 2024 | | | HDACi + IDO1i in MSS colorectal cancer | No | Preclinical study, lacks clinical relevance |
| 324 | | Lv et al., 2024 | | | Cancer-associated fibroblasts in tumorigenicity | No | Focus on tumor biology, not treatment |
| 325 | | Cotan et al., 2024 | | | Prognostic and predictive markers in CRC | No | Focus on markers, not treatment |
| 326 | | Duta-Ion et al., 2024 | | | Molecular pathways in colorectal cancer | No | Broad review, lacks immunotherapy focus |
| 327 | | Spirito et al., 2024 | | | Oncolytic virotherapy in head & neck cancer | No | Different cancer type (head & neck) |
| 328 | | Albertí-Valls et al., 2024 | | | Immunotherapy in endometrial cancer | No | Different cancer type (endometrial) |
| 329 | | Sowter et al., 2024 | | | MMR detection in endometrial cancer | No | Different cancer type (endometrial) |
| 330 | | Henmi et al., 2024 | | | Suppression of colon cancer metastasis in mice | No | Preclinical study |
| 331 | | Ruiz-Lorente et al., 2024 | | | Immunoresponse in bladder cancer | No | Different cancer type (bladder) |
| 332 | | Sun et al., 2024 | | | Nucleic acid drugs in cancer treatment | No | Broad review, lacks CRC focus |
| 333 | | Delahousse et al., 2024 | | | Sex differences in pharmacokinetics of anticancer drugs | No | Focus on pharmacokinetics, not treatment |
| 334 | | Berriel Diaz et al., 2024 | | | Cancer cachexia and metabolic dysfunction | No | Focus on cachexia, not treatment |
| 335 | | Duan et al., 2024 | | | Multi-omic analysis in lung cancer & brain metastasis | No | Different cancer type (lung, brain) |
| 336 | | Ciernikova et al., 2024 | | | Gut & tumor microbiome in cancer resistance | No | Focus on microbiome, not treatment |
| 337 | | Lin et al., 2024 | | | Photochemotherapy nanodrug in HNSCC | No | Different cancer type (head & neck) |
| 338 | | Mei et al., 2024 | | | Nanoparticles & immunogenic death in cancer | No | Preclinical study |
| 339 | | Su et al., 2024 | | | Tumor microenvironment remodeling in rectal cancer | No | Focus on microenvironment, not treatment |
| 340 | | Xie et al., 2024 | | | Innate immunity + PD-1/PD-L1 blockade in CRC | No | Preclinical study |
| 341 | | Yang et al., 2024 | | | VEGF family in colon adenocarcinoma | No | Focus on VEGF signaling, not immunotherapy |
| 342 | | Yu et al., 2024 | | | Tertiary lymphoid structures in cancer | No | Focus on immune microenvironment, not direct treatment |
| 343 | | Zhu et al., 2024 | | | Immune dynamics in liver metastases | No | Focus on metastases, not primary CRC treatment |
| 344 | | Wu et al., 2024 | | | DDR1 as an immunotherapy target in MSS colon cancer | No | Preclinical study, lacks clinical relevance |
| 345 | | Xiao et al., 2024 | | | Regorafenib, ipilimumab, nivolumab in MSS non-liver mCRC | No | Phase I trial, lacks broader clinical validation |
| 346 | | Cao et al., 2024 | | | ENC1 as a tumor microenvironment biomarker | No | Focus on biomarker, not treatment |
| 347 | | Yang et al., 2024 | | | Chronic stress and intestinal immunity in CRC | No | Focus on stress-induced immune changes, not treatment |
| 348 | | Mushtaq et al., 2024 | | | X-PDT as a new cancer therapy | No | Not related to immunotherapy |
| 349 | | Götz et al., 2024 | | | CEACAM1 in cancer | No | Focus on tumor markers, not treatment |
| 350 | | Cortés-Ballinas et al., 2024 | | | STAT3/survivin as targets in CRC | No | Preclinical study, lacks clinical relevance |
| 351 | | Svec et al., 2024 | | | CRC development & therapy | No | General overview, lacks immunotherapy focus |
| 352 | | Luo et al., 2024 | | | Ubiquitin genes in ovarian carcinoma | No | Different cancer type (ovarian) |
| 353 | | Jubashi et al., 2024 | | | Targeted therapy in esophageal SCC | No | Different cancer type (esophageal) |
| 354 | | Ielpo et al., 2024 | | | CAF-NK/DC communication in immunotherapy | No | Focus on immune mechanisms, not direct treatment |
| 355 | | Wu et al., 2024 | | | Cold & hot tumors in immunotherapy | No | General immunotherapy, lacks CRC focus |
| 356 | | Wang et al., 2024 | | | Biomarkers for immunotherapy in mesothelioma | No | Different cancer type (mesothelioma) |
| 357 | | Yang et al., 2024 | | | Nano-PROTACs in CRC therapy | No | Preclinical study, lacks clinical validation |
| 358 | | Feng et al., 2024 | | | Macrophage-driven CRC transcriptional insights | No | Focus on immune profiling, not treatment |
| 359 | | Huang et al., 2024 | | | Branched N-glycan PD-L1 in HNSCC | No | Different cancer type (HNSCC) |
| 360 | | Li et al., 2024 | | | Signaling pathways in CRC | No | General molecular review, lacks immunotherapy focus |
| 361 | | Luo et al., 2024 | | | TRIM37 and sunitinib resistance in RCC | No | Different cancer type (renal) |
| 362 | | Pan et al., 2024 | | | γδ T cells in CRC | No | Focus on immune mechanisms, not treatment |
| 363 | | Pu et al., 2024 | | | T-cell function regulators in CRC | No | Preclinical study, lacks clinical validation |
| 364 | | Vangilbergen et al., 2024 | | | IL-17/IL-23 inhibitors in cancer | No | General immune modulation, lacks CRC focus |
| 365 | | Markelc et al., 2024 | | | pmIL12 gene therapy preclinical study | No | Preclinical study, lacks clinical data |
| 366 | | Roth et al., 2024 | | | CD8+ T cells & HIPEC in peritoneal metastasis | No | Focus on surgery-based therapy, not immunotherapy |
| 367 | | Mo et al., 2024 | | | New therapies for mCRC | No | General treatment review, lacks immunotherapy focus |
| 368 | | Zhang et al., 2024 | | | SMARCA4 mutations in lung adenocarcinoma | No | Different cancer type (lung) |
| 369 | | Williams et al., 2024 | | | Neoadjuvant immunotherapy in CRC | No | Broad review, lacks novel insights |
| 370 | | Khalili-Tanha et al., 2024 | | | Biomarkers in gastric cancer | No | Different cancer type (gastric) |
| 371 | | Venturini et al., 2024 | | | HER2 in CRC | No | Focuses on HER2, not general CRC treatment |
| 372 | | Shi et al., 2024 | | | Intratumor microbiota in cancer | No | Focuses on microbiota, not CRC therapy |
| 373 | | Benboubker et al., 2024 | | | CRC organoid treatment validation | No | Preclinical study, not clinical therapy |
| 374 | | Incorvaia et al., 2024 | | | HR-MMR intersection in cancer | No | Broad genetic focus, not treatment-based |
| 375 | | Kanoda et al., 2024 | | | STING signaling in MSI-H GC | No | Focuses on gastric cancer, not CRC |
| 376 | | Suto et al., 2024 | | | Tregs in endometrial cancer | No | Focuses on endometrial cancer, not CRC |
| 377 | | Zhang et al., 2024 | | | CAFs and tumor metabolism | No | Not specific to CRC therapy |
| 378 | | Zhang et al., 2024 | | | Locoregional therapy + ICI in liver metastases | No | Broad liver metastasis study, not CRC-specific |
| 379 | | Nobin et al., 2024 | | | PD-L1 expression in CRC | No | Biomarker-focused, not treatment-based |
| 380 | | Fan et al., 2024 | | | MMR-deficient CRC characteristics | No | Descriptive study, not focused on new therapies |
| 381 | | Detchou et al., 2024 | | | IL-4 in glioblastoma resistance | No | Focuses on glioblastoma, not CRC |
| 382 | | Yang et al., 2024 | | | Prognostic model for breast cancer | No | Focuses on breast cancer, not CRC |
| 383 | | Chong et al., 2024 | | | Immunotherapy for GI cancers | No | Broad GI focus, not CRC-specific |
| 384 | | Zheng et al., 2024 | | | Tumor immune phenotypes | No | Focuses on immune mechanisms, not therapy |
| 385 | | Nie et al., 2024 | | | Urea cycle regulation in CRC | No | Preclinical study, lacks clinical data |
| 386 | | Schwarz et al., 2024 | | | Peptide-stimulated T cells in MSI CRC | No | Preclinical study, not clinical treatment |
| 387 | | Jasim et al., 2024 | | | siRNA therapy in liver cancer | No | Focuses on liver cancer, not CRC |
| 388 | | Liu et al., 2024 | | | Ubiquitination in cancer therapy | No | Broad cancer focus, not CRC-specific |
| 389 | | Xiang et al., 2024 | | | Proteogenomics in melanoma | No | Focuses on melanoma, not CRC |
| 390 | | Qiao et al., 2024 | | | MSS/MSI classifier in CRC | No | Biomarker study, not treatment-focused |
| 391 | | Dai et al., 2024 | | | Negative lymph node count in MSI CRC | No | Prognostic study, not treatment-related |
| 392 | | Liu et al., 2024 | | | Tumor treatment strategies | No | Broad cancer focus, not CRC-specific |
| 393 | | Monti et al., 2024 | | | Plasmacytoid DCs in cancer immunity | No | Focuses on immune modulation, not therapy |
| 394 | | Cantero et al., 2024 | | | MSC-based therapy in HCC | No | Focuses on liver cancer, not CRC |
| 395 | | Hu et al., 2024 | | | Tumor pyroptosis in immunotherapy | No | Focuses on general cancer, not CRC |
| 396 | | Olivera et al., 2024 | | | Regional T-cell therapy | No | Not CRC-specific, lacks clinical application |
| 397 | | Li et al., 2024 | | | Loureirin analogs for CRC | No | Preclinical study, lacks clinical data |
| 398 | | Choi et al., 2024 | | | PIKfyve in tumor immunity | No | Preclinical study, not focused on CRC treatment |
| 399 | | Heidari Horestani et al., 2024 | | | BHLHE40 in prostate cancer | No | Focuses on prostate cancer, not CRC |
| 400 | | Luo et al., 2024 | | | Gasdermin E in CRC | No | Mechanistic study, not focused on clinical treatment |
| 401 | | Song et al., 2024 | | | Signaling pathways in CRC | No | Focuses on signaling pathways, not direct therapies |
| 402 | | Pitts et al., 2024 | | | Soluble immune checkpoints | No | Focuses on biomarkers, not CRC treatment |
| 403 | | Wu et al., 2024 | | | PD-1/PD-L1 inhibitors in early/mid CRC | No | Broad review, not specific to novel therapies |
| 404 | | Liu et al., 2024 | | | Cancer biotherapy review | No | Not specific to CRC |
| 405 | | Gustav et al., 2024 | | | Deep learning for MSI/POLE in CRC | No | Focuses on diagnostics, not treatment |
| 406 | | Zhang et al., 2024 | | | Dual-target rebalance in tumors | No | Preclinical study, no clinical data yet |
| 407 | | Yin et al., 2024 | | | Immunoregulatory anti-cancer drugs | No | Broad focus, not CRC-specific |
| 408 | | Hwang et al., 2024 | | | Therapy in cutaneous SCC | No | Focuses on SCC, not CRC |
| 409 | | Zhou et al., 2024 | | | Tumor biomarkers for therapy | No | Focuses on biomarkers, not direct treatment |
| 410 | | Huang et al., 2024 | | | Neutrophils in cancer therapy | No | Not specific to CRC |
| 411 | | Li et al., 2024 | | | Gut microbiota in cancer therapy | No | Focuses on microbiota, not direct treatment |
| 412 | | Chen et al., 2024 | | | CD8+ T cells in immunotherapy | No | Not specific to CRC |
| 413 | | Guan et al., 2024 | | | Mitochondrial genome in tumor progression | No | Focuses on metabolism, not direct treatment |
| 414 | | Chauhan et al., 2024 | | | Immunosuppression in metastatic BC | No | Focuses on breast cancer, not CRC |
| 415 | | Cheng et al., 2024 | | | Bispecific Abs vs ICI in cancer | No | Not specific to CRC |
| 416 | | Zhang et al., 2024 | | | Plasma proteomics for lung cancer therapy | No | Focuses on lung cancer, not CRC |
| 417 | | Zhou et al., 2024 | | | Prognosis modeling in CRC liver metastases | No | Focuses on prognosis models, not treatment |
| 418 | | Hu et al., 2024 | | | Innate immune pathways in cancer | No | Not specific to CRC |
| 419 | | Kasikova et al., 2024 | | | TLS and B cells in ovarian cancer | No | Focuses on ovarian cancer, not CRC |
| 420 | | Deng et al., 2024 | | | TGF-β signaling in cancer | No | Not specific to CRC |
| 421 | | Deycmar et al., 2024 | | | MLH1 silencing in rhesus macaques | No | Animal model study, not human treatment |
| 422 | | Wang et al., 2024 | | | Lymphocytes in PD-1 therapy for GC | No | Focuses on gastric cancer, not CRC |
| 423 | | Xiao et al., 2024 | | | Dietary intervention in cancer | No | Not specific to CRC |
| 424 | | Guo et al., 2024 | | | NF-κB in cancer therapy | No | Not specific to CRC |
| 425 | | Wang et al., 2024 | | | TLS in rectal cancer | No | Focuses on rectal cancer, not general CRC |
| 426 | | Hu et al., 2024 | | | IL6-STAT3 in lung cancer | No | Focuses on lung cancer, not CRC |
| 427 | | Ebrahimi et al., 2024 | | | NF-κB and immune checkpoints | No | Not specific to CRC |
| 428 | | Thng et al., 2024 | | | Personalized therapy for mCRC | No | Organoid-based study, not direct treatment |
| 429 | | Saeed et al., 2024 | | | Cabozantinib + durvalumab in CRC | No | Phase II data, insufficient evidence |
| 430 | | Toulmonde et al., 2024 | | | Oncolytic virus + avelumab in STS | No | Focuses on STS, not CRC |
| 431 | | Li J, Yu T, Sun J, et al. 2024 | | | Comprehensive integration of single-cell RNA and transcriptome RNA sequencing to establish a pyroptosis-related signature for improving prognostic prediction of gastric cancer | No | Primarily focuses on prognostic prediction rather than immunotherapy application. |
| 432 | | Li T, Huang M, Sun N, et al. 2024 | | | Tumorigenesis of basal muscle invasive bladder cancer was mediated by PTEN protein degradation resulting from SNHG1 upregulation | No | Discusses tumorigenesis but lacks direct immunotherapy relevance. |
| 433 | | Zhao L, Wang Y, Mu P, et al. 2024 | | | IGFBP3 induces PD-L1 expression to promote glioblastoma immune evasion | No | Investigates immune evasion but lacks clinical immunotherapy intervention. |
| 434 | | Jiang Y, Zhao M, Tang W, et al. 2024 | | | Impacts of systemic treatments on health-related quality of life for patients with metastatic colorectal cancer: a systematic review and network meta-analysis | No | Focuses on quality of life rather than treatment effectiveness. |
| 435 | | Zhu K, Yang X, Tai H, et al. 2024 | | | HER2-targeted therapies in cancer: a systematic review | No | Primarily focused on targeted therapy, not immunotherapy. |
| 436 | | Yang Q, Zhuo Z, Qiu X, et al. 2024 | | | Adverse clinical outcomes and immunosuppressive microenvironment of RHO-GTPase activation pattern in hepatocellular carcinoma | No | Lacks direct implications for immunotherapy development. |
| 437 | | Han X, Guo Y, Ye H, et al. 2024 | | | Development of a machine learning-based radiomics signature for estimating breast cancer TME phenotypes and predicting anti-PD-1/PD-L1 immunotherapy response | No | Uses AI for prediction but lacks clinical application of immunotherapy. |
| 438 | | Sini MC, Doro MG, Frogheri L, et al. 2024 | | | Combination of mutations in genes controlling DNA repair and high mutational load plays a prognostic role in pancreatic ductal adenocarcinoma (PDAC): a retrospective real-life study in Sardinian population | No | Focuses on mutational analysis without immunotherapy application. |
| 439 | | Zhao Q, Zong H, Zhu P, et al. 2024 | | | Crosstalk between colorectal CSCs and immune cells in tumorigenesis, and strategies for targeting colorectal CSCs | No | Lacks direct clinical immunotherapy intervention. |
| 440 | | Meraz-Torres F, Niessner H, Plöger S, et al. 2024 | | | Augmenting MEK inhibitor efficacy in BRAF wild-type melanoma: synergistic effects of disulfiram combination therapy | No | Targets BRAF pathway without emphasis on immunotherapy. |
| 441 | | Gurbatri CR, Radford GA, Vrbanac L, et al. 2024 | | | Engineering tumor-colonizing E. coli Nissle 1917 for detection and treatment of colorectal neoplasia | No | Investigates bacterial therapies, not immune-based treatments. |
| 442 | | Wang J, Peng J, Chen Y, et al. 2024 | | | The role of stromal cells in epithelial–mesenchymal plasticity and its therapeutic potential | No | Focuses on tumor microenvironment rather than immunotherapy. |
| 443 | | Qin G, Bai F, Hu H, et al. 2024 | | | Targeting the NAT10/NPM1 axis abrogates PD-L1 expression and improves the response to immune checkpoint blockade therapy | No | Lacks sufficient clinical validation for immunotherapy application. |
| 444 | | Ye F, Chen M, Zheng X, et al. 2024 | | | Clinicopathological and molecular characteristics of colorectal adenosquamous carcinoma in an Asian population | No | Primarily descriptive without immunotherapy relevance. |
| 445 | | Hou S, Zhao Y, Chen J, et al. 2024 | | | Tumor-associated macrophages in colorectal cancer metastasis: molecular insights and translational perspectives | No | Discusses immune cells but lacks direct immunotherapy application. |
| 446 | | Cao Y, Xia H, Tan X, et al. 2024 | | | Intratumoural microbiota: a new frontier in cancer development and therapy | No | Explores microbiota influence on cancer but lacks immunotherapy focus. |
| 447 | | Wang Y, Suarez ER, Kastrunes G, et al. 2024 | | | Evolution of cell therapy for renal cell carcinoma | No | Concentrates on cell therapy rather than immune-based treatments. |
| 448 | | Yang G, Cai S, Hu M, et al. 2024 | | | Spatial features of specific CD103+CD8+ tissue-resident memory T cell subsets define the prognosis in patients with non-small cell lung cancer | No | Primarily focused on prognosis, not immunotherapy intervention. |
| 449 | | Ascierto PA, Casula M, Bulgarelli J, et al. 2024 | | | Sequential immunotherapy and targeted therapy for metastatic BRAF V600 mutated melanoma: 4-year survival and biomarkers evaluation from the phase II SECOMBIT trial | No | Provides survival data but lacks new immunotherapy strategies. |
| 450 | | Xing T, Li L, Rao X, et al. 2024 | | | ARID1A deficiency promotes progression and potentiates therapeutic antitumour immunity in hepatitis B virus-related hepatocellular carcinoma | No | Links ARID1A mutation to immune response but lacks actionable therapy. |
| 451 | | Amodio et al., 2024 | | | DNA repair-dependent immunogenic liabilities in colorectal cancer: opportunities from errors | No | Irrelevant |
| 452 | | Field et al., 2024 | | | SMARCA4 and SMARCA2 co-deficiency: A molecular signature in aggressive malignancies | No | Irrelevant |
| 453 | | Istomina et al., 2024 | | | Phage display for discovery of anticancer antibodies | No | Irrelevant |
| 454 | | Gandhi et al., 2024 | | | Systemic chemokine-modulatory regimen with neoadjuvant chemotherapy in breast cancer | No | Irrelevant |
| 455 | | Li et al., 2024 | | | Small-Molecule Therapeutics Sensitizing CRC to Immune Checkpoint Blockade | No | Irrelevant |
| 456 | | Schwarz et al., 2024 | | | Inhibition of Bruton’s tyrosine kinase with PD-1 blockade in solid tumors | No | Irrelevant |
| 457 | | Ma et al., 2024 | | | Mechanisms of B vitamins in colorectal cancer risk | No | Irrelevant |
| 458 | | Boland et al., 2024 | | | TAS-102, Irinotecan, and bevacizumab in metastatic CRC (TABAsCO trial) | No | Irrelevant |
| 459 | | Underwood et al., 2024 | | | Precision Medicine for Metastatic Colorectal Cancer | No | Irrelevant |
| 460 | | Holla et al., 2024 | | | Genomic Alterations in DNA Mismatch Repair Genes Across Cancer Types | No | Irrelevant |
| 461 | | Contrera et al., 2024 | | | CD8+ Tumor-Infiltrating Lymphocytes in Head and Neck Cancer | No | Irrelevant |
| 462 | | Pan et al., 2024 | | | Multifaceted roles of neutrophils in tumor microenvironment | No | Irrelevant |
| 463 | | Li et al., 2024 | | | Targeting extracellular matrix interaction in gastrointestinal cancer | No | Irrelevant |
| 464 | | Rocca et al., 2024 | | | Germline Variant Spectrum in High-Risk Hereditary Breast Cancer Patients | No | Irrelevant |
| 465 | | Mitiushkina et al., 2024 | | | 3′ RACE-Based Targeted RNA Sequencing for Urothelial Carcinomas | No | Irrelevant |
| 466 | | Derakhshandeh et al., 2024 | | | Identification of Functional Immune Biomarkers in Breast Cancer | No | Irrelevant |
| 467 | | Peng et al., 2024 | | | Advancements in p53-Based Anti-Tumor Gene Therapy | No | Irrelevant |
| 468 | | Nkosi et al., 2024 | | | Hypocrellin as a Photosensitizer for PDT of Melanoma | No | Irrelevant |
| 469 | | Li et al., 2024 | | | RNF43 and ZNRF3: Regulators in Cancer | No | Irrelevant |
| 470 | | Tosato et al., 2024 | | | 1945 JNCI Pioneering Contribution to Antiangiogenic Therapy | No | Irrelevant |
| 471 | | Matsuoka et al., 2024 | | | Malignant Progression of Gastric Cancer Within the Tumor Microenvironment | No | Irrelevant |
| 472 | | Federica et al., 2024 | | | Targeting the DNA Damage Response in Cancer | No | Irrelevant |
| 473 | | Fan et al., 2024 | | | Neuroscience in Peripheral Cancers: Tumor-Nerve Interactions | No | Irrelevant |
| 474 | | Zhang et al., 2024 | | | VISTA-mediated immune evasion in cancer | No | Irrelevant |
| 475 | | Wang et al., 2024 | | | Molecular Mechanisms of Targeting Esophageal Carcinoma | No | Irrelevant |
| 476 | | Qi et al., 2024 | | | Targeted Modulation of Myeloid-Derived Suppressor Cells in Cancer | No | Irrelevant |
| 477 | | Chen et al., 2024 | | | Matrix Stiffness in CRC Immunotherapy | No | Irrelevant |
| 478 | | Goyal et al., 2024 | | | Wnt/β-catenin Signaling in Future Cancer Therapies | No | Irrelevant |
| 479 | | Wu et al., 2024 | | | Tumor Budding in Intrahepatic Cholangiocarcinoma | No | Irrelevant |
| 480 | | Kalyanaraman et al., 2024 | | | Short-Chain Fatty Acids in Cancer Prevention & Treatment | No | Irrelevant |
| 481 | | Jiang et al., 2024 | | | Unraveling the mysteries of MGMT: Implications for neuroendocrine tumors | No | Irrelevant |
| 482 | | Liu et al., 2024 | | | Involvement of SIRT1-mediated cellular immune response in cancer | No | Irrelevant |
| 483 | | Ge et al., 2024 | | | Liquid biopsy: Comprehensive overview of circulating tumor DNA | No | Irrelevant |
| 484 | | Li et al., 2024 | | | Neoadjuvant Immunotherapy Alone for Patients with dMMR/MSI-H Colorectal Cancer | No | - |
| 485 | | Zeng et al., 2024 | | | Local TSH/TSHR signaling promotes CD8+ T cell exhaustion in CRC | No | Irrelevant |
| 486 | | Oaknin et al., 2024 | | | Efficacy of Trastuzumab Deruxtecan in HER2-Expressing Solid Tumors | No | Irrelevant |
| 487 | | Gao et al., 2024 | | | Hepatocyte growth factor promotes melanoma metastasis | No | Irrelevant |
| 488 | | Yu et al., 2024 | | | cGAS/STING signalling pathway in senescence and oncogenesis | No | Irrelevant |
| 489 | | Koedijk et al., 2024 | | | Immune phenotypes in pediatric acute myeloid leukemia | No | Irrelevant |
| 490 | | Pan et al., 2024 | | | FN1 as a prognostic biomarker for thyroid cancer | No | Irrelevant |
| 491 | | Xue et al., 2024 | | | Role of GDF15 in gastrointestinal cancer | No | Irrelevant |
| 492 | | Matboli et al., 2024 | | | Machine-Learning-Based RNA-Signature for Hepatocellular Carcinoma | No | Irrelevant |
| 493 | | Suzuki et al., 2024 | | | Current status of vaccine immunotherapy for gastrointestinal cancers | No | Irrelevant |
| 494 | | Mneimneh et al., 2024 | | | Drug-repurposed-loaded nanocarriers for colorectal cancer treatment | No | Irrelevant |
| 495 | | Fabian et al., 2024 | | | Alum-anchored IL-12 combined with chemotherapy in head and neck cancer | No | Irrelevant |
| 496 | | Poschel et al., 2024 | | | PD-L1 restrains PD-1+Nrp1lo Treg cells in colorectal cancer | No | Irrelevant |
| 497 | | Zhang et al., 2024 | | | Immunogenic cell death-based oncolytic virus therapy | No | Irrelevant |
| 498 | | Fan et al., 2024 | | | Lipopolyplex-formulated mRNA cancer vaccine elicits neoantigen-specific T cell responses | No | Irrelevant |
| 499 | | Williams et al., 2024 | | | Evaluation of CD3/CD8 T-Cell IHC for Prognostication in CRC | No | Irrelevant |
| 500 | | Segal et al., 2024 | | | COLUMBIA-1: Durvalumab plus oleclumab in MSS CRC | No | Irrelevant |
| 501 | | Hu et al., 2024 | | | Key Genes Associated with Temozolomide Resistance in Glioblastoma | No | Irrelevant |
| 502 | | Fujii et al., 2024 | | | Comprehensive Genomic Assessment of Advanced-Stage GI Stromal Tumors | No | Irrelevant |
| 503 | | Ricci et al., 2024 | | | Tumor Immune Microenvironment in Intrahepatic Cholangiocarcinoma | No | Irrelevant |
| 504 | | Glauß et al., 2024 | | | Chimeric Oncolytic Virus Mediates a Multifaceted Cellular Immune Response | No | Irrelevant |
| 505 | | González-Montero et al., 2024 | | | Predictors of Response to Immunotherapy in Colorectal Cancer | No | - |
| 506 | | Kehmann et al., 2024 | | | Therapeutic Landscape of Advanced Biliary Tract Cancer | No | Irrelevant |
| 507 | | Negrón-Figueroa et al., 2024 | | | Intratumoral Microbiome and Immunotherapy Response | No | Irrelevant |
| 508 | | Montauti et al., 2024 | | | CD4+ T Cells in Antitumor Immunity | No | Irrelevant |
| 509 | | Mastrogeorgiou et al., 2024 | | | Immune Microenvironment of Cervical Cancer | No | Irrelevant |
| 510 | | Zhang et al., 2024 | | | HER2-Targeted Therapy and Resistance in Colorectal Cancer | No | Irrelevant |
| 511 | | Almasoud et al., 2024 | | | Ramucirumab and 5-Azacitidine in Hepatoma Cells | No | Irrelevant |
| 512 | | Willenbockel et al., 2024 | | | Metabolic Role of Infections in the Tumor Microenvironment | No | Irrelevant |
| 513 | | Budczies et al., 2024 | | | Tumor Mutational Burden: Clinical Utility and Challenges | No | Irrelevant |
| 514 | | Kamal et al., 2024 | | | Novel Drug Delivery Systems in Colorectal Cancer | No | Irrelevant |
| 515 | | Yang et al., 2024 | | | Spatial Immune Landscape of CD103+CD8+ T Cells in Lung Cancer | No | Irrelevant |
| 516 | | Rauth et al., 2024 | | | Trends in Gastrointestinal Cancer Targeted Therapies | No | Irrelevant |
| 517 | | Bhagyalalitha et al., 2024 | | | Advances in HER2-Targeted Therapies | No | Irrelevant |
| 518 | | Ayala-de Miguel et al., 2024 | | | Third-Line Treatment and Beyond in Metastatic Colorectal Cancer | No | Irrelevant |
| 519 | | Mendiola et al., 2024 | | | Idylla Microsatellite Instability Test in Endometrial Cancer | No | Irrelevant |
| 520 | | Horikawa et al., 2024 | | | IL-10-Producing T-Cell Response in Head and Neck Cancer | No | Irrelevant |
| 521 | | Carlomagno et al., 2024 | | | Pancreatic Ductal Adenocarcinoma Microenvironment and NK Cells | No | Irrelevant |
| 522 | | Shawer et al., 2024 | | | Adverse Effects of Anti-Cancer Biologics on the Ocular Surface | No | Irrelevant |
| 523 | | Mikó et al., 2024 | | | Guidelines for Microbiome Studies in Neoplastic Diseases | No | Irrelevant |
| 524 | | Liu et al., 2024 | | | Cryoablation and PD-1 Inhibitor Synergy in Lung Adenocarcinoma | No | Irrelevant |
| 525 | | Ni et al., 2024 | | | Exploring Liver Metastasis in Prostate Cancer | No | Irrelevant |
| 526 | | Cai et al., 2024 | | | Multi-Pathway Combination Therapy for MSS Colorectal Cancer | No | - |
| 527 | | Chen et al., 2024 | | | Histogram Analysis for Mismatch Repair Status in Rectal Adenocarcinoma | No | Irrelevant |
| 528 | | Hu et al., 2024 | | | TIM-3/CD68 Expression in Glioma: Prognosis and Therapeutic Approaches | No | Irrelevant |
| 529 | | Tran et al., 2024 | | | TCF-1 and T Cell Differentiation in Colorectal Cancer | No | Irrelevant |
| 530 | | Zhang et al., 2024 | | | Disulfidptosis and Ferroptosis-Related Genes in Bladder Cancer | No | Irrelevant |
| 531 | | Zhang et al., 2024 | | | Modified Banxiaxiexin Decoction Benefits Chemotherapy in Gastric Cancer | No | Irrelevant |
| 532 | | Özbay Kurt et al., 2024 | | | S100A9 and HMGB1 in MDSC-Mediated Immunosuppression in Melanoma | No | Irrelevant |
| 533 | | Yang et al., 2024 | | | Development of CXCR4 Antagonists for Disease Therapy | No | Irrelevant |
| 534 | | Ajadee et al., 2024 | | | Gene Expression in Clear Cell Renal Cell Carcinoma | No | Irrelevant |
| 535 | | Ghadrdoost Nakhchi et al., 2024 | | | Targeting Endothelial Rap1B to Overcome Vascular Immunosuppression | No | Irrelevant |
| 536 | | Liu et al., 2024 | | | Emerging Targets in Non-Small Cell Lung Cancer | No | Irrelevant |
| 537 | | Shah et al., 2024 | | | Cancer-Associated Fibroblast Proteins as Targets in CRC | No | Irrelevant |
| 538 | | Quaquarini et al., 2024 | | | HER2 Status in Non-Breast and Non-Gastroesophageal Carcinomas | No | Irrelevant |
| 539 | | Ochman et al., 2024 | | | Associations of SEMA7A, SEMA4D, ADAMTS10, and ADAM8 in CRC | No | Irrelevant |
| 540 | | Dinić et al., 2024 | | | Cancer Patient-Derived Cell-Based Models | No | Irrelevant |
| 541 | | Rapanotti et al., 2024 | | | Circulating Tumor Cells: Current Applications and Future Perspectives | No | Irrelevant |
| 542 | | Mihaila et al., 2024 | | | Predictive Biomarkers and Immunotherapy Response in Solid Tumors | No | Irrelevant |
| 543 | | Gharib et al., 2024 | | | Holistic Perspective on Colorectal Carcinogenesis | No | Irrelevant |
| 544 | | Çakan et al., 2024 | | | Therapeutic Antisense Oligonucleotides in Oncology | No | Irrelevant |
| 545 | | Pavelescu et al., 2024 | | | Resistance Mechanisms of Checkpoint Inhibitors in Solid Tumors | No | Irrelevant |
| 546 | | Liu et al., 2024 | | | Tumor-Associated Exosomes in Cancer Progression | No | Irrelevant |
| 547 | | Reitsam et al., 2024 | | | AI in Colorectal Cancer: Screening and Treatment Decisions | No | Irrelevant |
| 548 | | Voutsadakis et al., 2024 | | | PIK3CA Mutated CRC Without KRAS, NRAS, and BRAF Mutations | No | Irrelevant |
| 549 | | Takaoka et al., 2024 | | | Prevalence of Cachexia in Cancer Patients | No | Irrelevant |
| 550 | | Cheraghpour et al., 2024 | | | Gut Microbiome-Related Strategies in CRC | No | Irrelevant |
| 551 | | Teillaud et al., 2024 | | | Tertiary Lymphoid Structures in Anticancer Immunity | No | Irrelevant |
| 552 | | Jia et al., 2024 | | | Targeting Metabolic Pathways in Tumor Microenvironment | No | Irrelevant |
| 553 | | Silva-Hurtado et al., 2024 | | | Hypomethylating Agents in Glioblastoma | No | Irrelevant |
| 554 | | Cern et al., 2024 | | | Nano-Mupirocin as Tumor-Targeted Antibiotic | No | Irrelevant |
| 555 | | Zeng et al., 2024 | | | Myeloid-Derived Suppressor Cells in CRC | No | Irrelevant |
| 556 | | Tian et al., 2024 | | | Role of AMIGO2 in Cancer Progression | No | Irrelevant |
| 557 | | Ius et al., 2024 | | | Local Therapy in Glioma: From History to Horizons | No | Irrelevant |
| 558 | | Cao et al., 2024 | | | Host-Gut Microbiota Metabolic Interactions in GI Cancers | No | Irrelevant |
| 559 | | Kennedy et al., 2024 | | | Safety of TARE with Yttrium-90 and Systemic Therapy in Liver Cancer | No | Irrelevant |
| 560 | | Zhou et al., 2024 | | | Hot and Cold Tumor Prognostic Signature in Stage II CRC | No | Irrelevant |
| 561 | | Hernando-Calvo et al., 2024 | | | Durvalumab With Olaparib or Cediranib in MMR-Proficient CRC/PC | No | Irrelevant |
| 562 | | Jones et al., 2024 | | | HER-2 Directed Therapies Across GI Cancers | No | Irrelevant |
| 563 | | Bessudo et al., 2024 | | | Vicriviroc + Pembrolizumab in MSS CRC | No | - |
| 564 | | Niu et al., 2024 | | | Macrophages and TLS as Prognostic Indicators in Cancer | No | Irrelevant |
| 565 | | Cañellas-Socias et al., 2024 | | | Mechanisms of Metastatic CRC | No | Irrelevant |
| 566 | | Du et al., 2024 | | | JAK Inhibitor Overcomes ICI Resistance in GC | No | Irrelevant |
| 567 | | Nishida et al., 2024 | | | HER2-Negative Advanced GC Treatment Strategies | No | Irrelevant |
| 568 | | Shi et al., 2024 | | | Hepatic Arterial Infusion Chemo + Camrelizumab in PHNEC | No | Irrelevant |
| 569 | | Men et al., 2024 | | | PD-1 Blockade + Chemo + Bevacizumab in MSS CRC Liver Metastases | No | - |
| 570 | | Zou et al., 2024 | | | Targeting Immunosuppressive Cells in CRC TME | No | Irrelevant |
| 571 | | Kong et al., 2024 | | | Immunomodulatory Molecules in CRC Liver Metastasis | No | Irrelevant |
| 572 | | Allevato et al., 2024 | | | Glutamine Metabolism and Ferroptosis in HNSCC | No | Irrelevant |
| 573 | | Zhang et al., 2024 | | | Immune Combination Therapy in MSS/pMMR CRC | No | - |
| 574 | | Fan et al., 2024 | | | Intratumoral Microbiota in CRC Immunotherapy | No | Irrelevant |
| 575 | | Aung et al., 2024 | | | Spatially Informed Gene Signatures in Melanoma | No | Irrelevant |
| 576 | | Liu et al., 2024 | | | Serum LDH as a Prognostic Marker in NPC | No | Irrelevant |
| 577 | | Li et al., 2024 | | | Seamless Phase II/III Design for Oncology Drugs | No | Irrelevant |
| 578 | | Di Nitto et al., 2024 | | | IL-7 Fusion Protein Targeting EDA FN in Cancer | No | Irrelevant |
| 579 | | Swierz et al., 2024 | | | TACE vs Systemic Chemo in CRC Liver Metastases | No | Irrelevant |
| 580 | | Cortese et al., 2024 | | | HER2 SynNotch/CEA-CAR in CRC | No | Irrelevant |
| 581 | | Wang et al., 2024 | | | Epigenetic Inhibitor Nanoprodrug + Oncolytic Virus | No | Irrelevant |
| 582 | | Gonzalez-Gutierrez et al., 2024 | | | Obesity-Associated CRC | No | Irrelevant |
| 583 | | Herrera-Quintana et al., 2024 | | | Cancer and the Microbiome | No | Irrelevant |
| 584 | | Ashouri et al., 2024 | | | Predictive and Prognostic Biomarkers in CRC | No | Irrelevant |
| 585 | | Mo et al., 2024 | | | Resistance to Anti-HER2 Therapies in GI Malignancies | No | Irrelevant |
| 586 | | Mechahougui et al., 2024 | | | Advances in Personalized Oncology | No | Irrelevant |
| 587 | | Huo et al., 2024 | | | RNF43 in Cancer and Immunotherapy | No | Irrelevant |
| 588 | | Dravillas et al., 2024 | | | Tumor Microbiome in Melanoma and ICI Response | No | Irrelevant |
| 589 | | Ito et al., 2024 | | | WT1 Epitope Immunogenicity in Mesothelioma | No | Irrelevant |
| 590 | | Zhao et al., 2024 | | | N6-Methyladenosine in Tumor Neovascularization | No | Irrelevant |
| 591 | | Mei et al., 2024 | | | Hydrogel-Based Drug Delivery in Cancer Therapy | No | Irrelevant |
| 592 | | Yang et al., 2024 | | | Improving ICI Efficiency in pMMR/MSS CRC | No | - |
| 593 | | Zhou et al., 2024 | | | Gut Microbiota Metabolite TMAO in Cancer | No | Irrelevant |
| 594 | | Meng et al., 2024 | | | Fecal Microbiota Transplantation for Anti-PD-1 Resistance | No | Irrelevant |
| 595 | | Ji et al., 2024 | | | Targeting Bacterial Metabolites in Tumors for Therapy | No | Irrelevant |
| 596 | | Zhou et al., 2024 | | | Microbes in Tumor Microenvironment & Immunotherapy | No | Irrelevant |
| 597 | | Ali et al., 2024 | | | Liposomic Nanoparticles in CRC and Ovarian Cancer | No | Irrelevant |
| 598 | | Ye et al., 2024 | | | Multi-Dimensional Apoptosis in Melanoma TME | No | Irrelevant |
| 599 | | Lee et al., 2024 | | | Hypoxia Biomarkers in Head & Neck Cancer Models | No | Irrelevant |
| 600 | | Liu et al., 2024 | | | Gene Signature for PTM in CRC and Therapeutic Response | No | Irrelevant |
| 601 | | Nielsen et al., 2024 | | | Abdominal Aortic Aneurysm Repair in Patients with Concomitant Cancer: A Literature Review | No | Irrelevant |
| 602 | | Xia et al., 2024 | | | Comprehensive analysis to identify the relationship between CALD1 and immune infiltration in glioma | No | Irrelevant |
| 603 | | Pan et al., 2024 | | | Identification of a cancer driver gene-associated lncRNA signature for prognostic prediction and immune response evaluation in clear cell renal cell carcinoma | No | Irrelevant |
| 604 | | Qin et al., 2024 | | | Prognosis of immune checkpoint inhibitor-related myocarditis: Retrospective experience of a single institution | No | Irrelevant |
| 605 | | Jin et al., 2024 | | | VISTA deficiency exerts anti-tumor effects in breast cancer through regulating macrophage polarization | No | Irrelevant |
| 606 | | Li et al., 2024 | | | Research Progress on Anticancer Components in Citrus and Their Action Mechanisms | No | Irrelevant |
| 607 | | Kang et al., 2024 | | | Immune-inflammatory modulation by natural products derived from edible and medicinal herbs used in Chinese classical prescriptions | No | Irrelevant |
| 608 | | Guasp et al., 2024 | | | RNA vaccines for cancer: Principles to practice | No | Irrelevant |
| 609 | | Colombo et al., 2024 | | | Immune checkpoint inhibitors in BRAF mutated metastatic colorectal cancer: A review | No | - |
| 610 | | Esmail et al., 2024 | | | The Recent Trends of Systemic Treatments and Locoregional Therapies for Cholangiocarcinoma | No | Irrelevant |
| 611 | | Dang et al., 2024 | | | Molecular subtypes of colorectal cancer in the era of precision oncotherapy: Current inspirations and future challenges | No | - |
| 612 | | Rigopoulos et al., 2024 | | | A Multi-Omics Analysis of an Exhausted T Cells’ Molecular Signature in Pan-Cancer | No | Irrelevant |
| 613 | | Muthukumaran et al., 2024 | | | Paraneoplastic Glomerular Diseases | No | Irrelevant |
| 614 | | Shilo et al., 2024 | | | Performance Analysis of Leica Biosystems Monoclonal Antibody Programmed Cell Death Ligand 1 Clone 73-10 on Breast, Colorectal, and Hepatocellular Carcinomas | No | Irrelevant |
| 615 | | Salva de Torres et al., 2024 | | | Current and Emerging Treatment Paradigms in Colorectal Cancer: Integrating Hallmarks of Cancer | No | - |
| 616 | | Kotrulev et al., 2024 | | | Soluble CD26: From Suggested Biomarker for Cancer Diagnosis to Plausible Marker for Dynamic Monitoring of Immunotherapy | No | Irrelevant |
| 617 | | Hossain et al., 2024 | | | Genomic and Epigenomic Biomarkers of Immune Checkpoint Immunotherapy Response in Melanoma: Current and Future Perspectives | No | Irrelevant |
| 618 | | Papachristos et al., 2024 | | | Medullary Thyroid Cancer: Molecular Drivers and Immune Cellular Milieu of the Tumour Microenvironment | No | Irrelevant |
| 619 | | O’Connell et al., 2024 | | | Challenges and Opportunities for Precision Surgery for Colorectal Liver Metastases | No | Irrelevant |
| 620 | | Rac et al., 2024 | | | Synthesis and Regulation of miRNA, Its Role in Oncogenesis, and Its Association with Colorectal Cancer Progression, Diagnosis, and Prognosis | No | - |
| 621 | | Nagasaki et al., 2024 | | | Spatial intratumor heterogeneity of programmed death-ligand 1 expression predicts poor prognosis in resected non–small cell lung cancer | No | Irrelevant |
| 622 | | Yaniz-Galende et al., 2024 | | | Spatial Profiling of Ovarian Carcinoma and Tumor Microenvironment Evolution under Neoadjuvant Chemotherapy | No | Irrelevant |
| 623 | | Frenette et al., 2024 | | | ACG Clinical Guideline: Focal Liver Lesions | No | Irrelevant |
| 624 | | Groen-Van Schooten et al., 2024 | | | Mapping the complexity and diversity of tertiary lymphoid structures in primary and peritoneal metastatic gastric cancer | No | Irrelevant |
| 625 | | Alteber et al., 2024 | | | PVRIG is Expressed on Stem-Like T Cells in Dendritic Cell–Rich Niches in Tumors and Its Blockade May Induce Immune Infiltration in Non-Inflamed Tumors | No | Irrelevant |
| 626 | | Wang et al., 2024 | | | Organoids in gastrointestinal diseases: from bench to clinic | No | Irrelevant |
| 627 | | Mehdikhani et al., 2024 | | | From immunomodulation to therapeutic prospects: Unveiling the biology of butyrophilins in cancer | No | Irrelevant |
| 628 | | Marin et al., 2024 | | | Strategies to enhance the response of liver cancer to pharmacological treatments | No | Irrelevant |
| 629 | | Nakatsu et al., 2024 | | | Interactions between diet and gut microbiota in cancer | No | Irrelevant |
| 630 | | Lin et al., 2024 | | | Evolving treatment paradigms for platinum-resistant ovarian cancer: An update narrative review | No | Irrelevant |
| 631 | | Gschnell et al., 2024 | | | Cutaneous metastases: From epidemiology to therapy | No | Irrelevant |
| 632 | | Agarwala et al., 2024 | | | Targeting metabolic pathways to counter cancer immunotherapy resistance | No | Irrelevant |
| 633 | | Liu et al., 2024 | | | Type X collagen knockdown inactivates ITGB1/PI3K/AKT to suppress TNBC progression | No | Irrelevant |
| 634 | | Jia et al., 2024 | | | Rapamycin circumvents anti PD-1 therapy resistance in colorectal cancer | No | - |
| 635 | | Zhu et al., 2024 | | | N6-methyladenosine RNA modification in gastrointestinal tract cancers | No | Irrelevant |
| 636 | | Su et al., 2024 | | | Epigenetic regulation as a strategy to overcome drug resistance in liver cancer | No | Irrelevant |
| 637 | | Ambrosini et al., 2024 | | | Immune checkpoint inhibitors for POLE/POLD1 proofreading-deficient colorectal cancer | No | - |
| 638 | | Cabrero-de las Heras et al., 2024 | | | Serum CXCL13 levels and outcomes in colorectal cancer patients | No | Irrelevant |
| 639 | | Cocco et al., 2024 | | | Patient-Derived Xenografts as a preclinical platform for cancer research | No | Irrelevant |
| 640 | | Gao et al., 2024 | | | VISTA: A novel checkpoint for cancer immunotherapy | No | Irrelevant |
| 641 | | Yu et al., 2024 | | | A humanized Anti-YKL-40 antibody inhibits tumor development | No | Irrelevant |
| 642 | | Singh et al., 2024 | | | Advancements in combining targeted therapy and immunotherapy for colorectal cancer | No | - |
| 643 | | Goebeler et al., 2024 | | | Bispecific and multispecific antibodies in oncology: opportunities and challenges | No | Irrelevant |
| 644 | | Fortin et al., 2024 | | | Circadian control of tumor immunosuppression and ICB efficacy | No | Irrelevant |
| 645 | | Volinsky-Fremond et al., 2024 | | | Prediction of recurrence risk in endometrial cancer with deep learning | No | Irrelevant |
| 646 | | Sangani et al., 2024 | | | The therapeutic impact of PD-1 in colorectal cancer | No | - |
| 647 | | Mencel et al., 2024 | | | Targeting microsatellite instability in gastrointestinal cancers | No | - |
| 648 | | Demir et al., 2024 | | | Emerging targeted therapies and resistance in biliary tract cancers | No | Irrelevant |
| 649 | | Geurts et al., 2024 | | | Mismatch repair deficiency vs microsatellite instability in immunotherapy | No | - |
| 650 | | Assal et al., 2024 | | | Blocking PD-1/PD-L1 and CD155/TIGIT in hepatocellular carcinoma | No | Irrelevant |
| 651 | | Chai et al., 2024 | | | DNA-delivered monoclonal antibodies targeting p53 R175H mutant | No | Irrelevant |
| 652 | | Eslinger et al., 2024 | | | ctDNA monitoring in Lynch syndrome with colorectal cancer | No | - |
| 653 | | He et al., 2024 | | | The role of CD8+ T-cells in colorectal cancer immunotherapy | No | - |
| 654 | | McKenzie et al., 2024 | | | NFκB signaling in colorectal cancer | No | Irrelevant |
| 655 | | Luo et al., 2024 | | | Necroptosis subtypes and tumor antigens for mRNA vaccines in colorectal cancer | No | Irrelevant |
| 656 | | Gagto et al., 2024 | | | Hybrid approaches to pulmonary metastasectomy: a review | No | Irrelevant |
| 657 | | Tran et al., 2024 | | | Oncolytic virus V937 with PD-1 blockade in liver and colorectal cancer | No | Irrelevant |
| 658 | | Li et al., 2024 | | | TMErisk score: A tumor microenvironment-based prognosis model | No | Irrelevant |
| 659 | | Nakao et al., 2024 | | | Tumor regression grade impact after preoperative CRT in colorectal cancer | No | - |
| 660 | | Heydari et al., 2024 | | | Alterations in DNA methylation patterns in gastrointestinal cancers | No | Irrelevant |
| 661 | | Ferretti et al., 2024 | | | Pt-resistance in gastrointestinal cancer cells and lncRNA expression | No | Irrelevant |
| 662 | | Anderson et al., 2024 | | | Treatment of Microsatellite-Unstable Rectal Cancer in Sporadic and Hereditary Settings | No | - |
| 663 | | Healy et al., 2024 | | | Advances in Therapies Targeting Inhibitory Checkpoint Receptors | No | - |
| 664 | | Gulubova et al., 2024 | | | Immunosuppression and Immunotherapy Strategies in MSS Colorectal Cancer | No | - |
| 665 | | Elbialy et al., 2024 | | | Patient-Derived Conditionally Reprogrammed Cells in Prostate Cancer | No | Irrelevant |
| 666 | | Zhou et al., 2024 | | | cGAS-STING in antitumor immunity: prospects and challenges | No | Irrelevant |
| 667 | | De Palma et al., 2024 | | | Tumor vascularization and its therapeutic targeting | No | Irrelevant |
| 668 | | Martínez-Pérez et al., 2024 | | | Targeted Treatment against Cancer Stem Cells in Colorectal Cancer | No | - |
| 669 | | Wu et al., 2024 | | | Immune Regulation and CD155 Signaling in Melanoma | No | Irrelevant |
| 670 | | Angelico et al., 2024 | | | ARID1A Mutations in Gastric Cancer | No | Irrelevant |
| 671 | | Emelyanova et al., 2024 | | | Mismatch Repair Genes and MSI Status in Pancreatic Cancer | No | Irrelevant |
| 672 | | Nikolouzakis et al., 2024 | | | Current and Future Trends of Colorectal Cancer Treatment | No | - |
| 673 | | Mouillet-Richard et al., 2024 | | | CMS4 Colon Cancer in Precision Medicine | No | - |
| 674 | | Mokhtarpour et al., 2024 | | | Oncolytic virus therapy in brain tumors | No | Irrelevant |
| 675 | | Beech et al., 2024 | | | Molecular Approach to Colorectal Carcinoma | No | - |
| 676 | | Li et al., 2024 | | | Single Organ Metastatic Sites in NSCLC: Patient Characteristics & Treatment | No | Irrelevant |
| 677 | | Yao et al., 2024 | | | Proteogenomic Analysis Identifies Neoantigens in Colorectal Cancer | No | - |
| 678 | | Yang et al., 2024 | | | Multi-modal Omics for Gastric Cancer Subtypes | No | Irrelevant |
| 679 | | Zhou et al., 2024 | | | Mouse Models for Head and Neck Squamous Cell Carcinoma | No | Irrelevant |
| 680 | | Zhou et al., 2024 | | | Epigenetic Modulations in Triple-Negative Breast Cancer | No | Irrelevant |
| 681 | | Xu et al., 2024 | | | Precision Medicine in Colorectal Cancer with Multi-Omics & AI | No | - |
| 682 | | Chen et al., 2024 | | | Fusobacterium nucleatum in Carcinogenesis & Drug Delivery | No | Irrelevant |
| 683 | | Wang et al., 2024 | | | Lymph Node Targeting for Cancer Vaccination | No | Irrelevant |
| 684 | | Kuwata et al., 2024 | | | Molecular Classification and Heterogeneity in Gastric Cancer | No | Irrelevant |
| 685 | | Chen et al., 2024 | | | Pembrolizumab Plus Binimetinib With or Without Chemotherapy for MSS CRC | No | - |
| 686 | | Voutsadakis et al., 2024 | | | Mediator Kinase Module and Super-Enhancer Genes in Colorectal Cancer | No | Irrelevant |
| 687 | | Han et al., 2024 | | | Immunotherapy for MSS Colorectal Cancer: Resistance and Strategies | No | - |
| 688 | | Mager et al., 2024 | | | Microbiota, Mucosal Malignancies, and Immunotherapy | No | Irrelevant |
| 689 | | Lee et al., 2024 | | | DNA Methylation Alterations in Cancer: Therapeutic Targeting | No | Irrelevant |
| 690 | | Yamamoto et al., 2024 | | | Microsatellite Instability: A 2024 Update | No | - |
| 691 | | Mohammadnezhad et al., 2024 | | | Atezolizumab and Bevacizumab in Advanced Hepatocellular Carcinoma: Cost-effectiveness | No | Irrelevant |
| 692 | | Peng et al., 2024 | | | Targeting MEK/COX-2 Axis to Improve Immunotherapy in dMMR CRC with PIK3CA Overexpression | No | - |
| 693 | | Huang et al., 2024 | | | NK Cells as a Therapeutic Tool in Cancer Immunotherapy | No | Irrelevant |
| 694 | | Thiery et al., 2024 | | | Integration of Proteomics in the Molecular Tumor Board | No | Irrelevant |
| 695 | | Abedizadeh et al., 2024 | | | Colorectal Cancer: Carcinogenesis, Diagnosis, and Novel Strategies for Treatment | No | - |
| 696 | | Suydam et al., 2024 | | | Management of Oligometastatic Colorectal Cancer | No | - |
| 697 | | Ma et al., 2024 | | | Downregulated CDH3 Correlated with Better Prognosis in LUAD | No | Irrelevant |
| 698 | | Li et al., 2024 | | | Prognostic Risk Model for Stomach Adenocarcinoma Based on ER Stress Genes | No | Irrelevant |
| 699 | | Luo et al., 2024 | | | Prognostic Role of MUCIN Family in Diffuse-Type Gastric Cancer | No | Irrelevant |
| 700 | | Peng et al., 2024 | | | Treatment Progress of BRAF V600E-Mutated Metastatic Colorectal Cancer | No | - |
| 701 | | Zhu et al., 2024 | | | PD-L1 Targeting Peptide-Based Radionuclide Molecular Probes | No | Irrelevant |
| 702 | | Li et al., 2024 | | | Stimulating STING for Cancer Therapy via Extracellular Route | No | Irrelevant |
| 703 | | Wu et al., 2024 | | | FERMT2 in Tumor Microenvironment & Immunotherapy in Pan-Cancer | No | Irrelevant |
| 704 | | Wang et al., 2024 | | | Prognostic & Treatment Markers in Hepatocellular Carcinoma via GPCR Analysis | No | Irrelevant |
| 705 | | Chhetri et al., 2024 | | | Inhibition of Metastasis in Colorectal Cancer via Anticancer Compounds | No | - |
| 706 | | Song et al., 2024 | | | mRNA Vaccines in Colorectal Cancer Immunotherapy | No | - |
| 707 | | Naing et al., 2024 | | | Phase 1 Study of Arginase Inhibitor INCB001158 with Pembrolizumab | No | - |
| 708 | | Zhang et al., 2024 | | | Cuproptosis-Related Gene ATP7B in IDH1 Wild-Type Glioma | No | Irrelevant |
| 709 | | Patel et al., 2024 | | | Immunomodulatory Role of Tinospora Cordifolia & Solanum Nigrum in CRC | No | Irrelevant |
| 710 | | Miller et al., 2024 | | | B7-H3 (CD276) as a Therapeutic Target in Human Malignancies | No | Irrelevant |
| 711 | | Fellhofer-Hofer et al., 2024 | | | Chemokines as Prognostic Factor in Colorectal Cancer | No | - |
| 712 | | Ryan et al., 2024 | | | Immune Cell Migration to Cancer | No | Irrelevant |
| 713 | | Mitra et al., 2024 | | | Overcoming Immune Evasion in Cancer Immunotherapy | No | - |
| 714 | | Larson et al., 2024 | | | Immunomodulatory Functions of Gemcitabine in Cancer | No | Irrelevant |
| 715 | | Yin et al., 2024 | | | RNA Methylation Genes INHBB & SOWAHA in MSI Colorectal Cancer | No | - |
| 716 | | Wendlinger et al., 2024 | | | Melanoma Susceptibility to Targeted Therapy & Neutrophil Protection | No | Irrelevant |
| 717 | | Dakal et al., 2024 | | | Tumor-Infiltrating Immune Cells & Personalized Cancer Treatments | No | - |
| 718 | | Guven et al., 2024 | | | Efficacy of Immune Checkpoint Inhibitors in MSS Colorectal Cancer | No | - |
| 719 | | Wen et al., 2024 | | | Immune Cell Prognostic Model & Tumor Immune Modulation in Esophageal Cancer | No | Irrelevant |
| 720 | | Zhang et al., 2024 | | | Intratumoral Microbiota in CRC: Diagnosis & Therapy | No | - |
| 721 | | Hosseinzadeh et al., 2024 | | | IFN-Gamma, STING Agonist & PD-1 Blockade in Gastric Cancer | No | Irrelevant |
| 722 | | Khosravi et al., 2024 | | | Immunologic Tumor Microenvironment Modulators for Cold Tumors | No | - |
| 723 | | Robinson et al., 2024 | | | HER2-Positive Metastatic Colorectal Cancer | No | - |
| 724 | | Fu et al., 2024 | | | Immune-Stimulating Antibody Conjugates (ISACs) from Bench to Bedside | No | Irrelevant |
| 725 | | Zhang et al., 2024 | | | Cancer Immunity by Tissue-Resident Innate Lymphoid & Killer T Cells | No | Irrelevant |
| 726 | | Kikuchi et al., 2024 | | | Molecular Tumor Marker Clinical Guidelines (2nd Edition) | No | Irrelevant |
| 727 | | Masheghati et al., 2024 | | | Role of Gut Microbiota & Probiotics in CRC Prevention & Treatment | No | Irrelevant |
| 728 | | Li et al., 2024 | | | Macrophage Lineage Transition & Immune Evasion in Gastric Cancer | No | Irrelevant |
| 729 | | Wang et al., 2024 | | | Modulation of Gut & Intra-Tumor Microbiota for ICI Response Enhancement | No | Irrelevant |
| 730 | | Lecomte et al., 2024 | | | French Guidelines for Non-Metastatic Colon Cancer | No | Irrelevant |
| 731 | | Horvath et al., 2024 | | | Bridging Neutrophil Diversity to New Therapeutic Approaches in NSCLC | No | Irrelevant |
| 732 | | Zhang et al., 2024 | | | DNA Damage Repair Signature in Esophageal Squamous Cell Carcinoma | No | Irrelevant |
| 733 | | Molimard et al., 2024 | | | Immune Infiltrate by HER2 Status in CRC | No | Irrelevant |
| 734 | | Cao et al., 2024 | | | Ubiquitin-Associated Genes in CRC Immunotherapy | No | - |
| 735 | | Chauhan et al., 2024 | | | p53 Deregulation & Immune Response Modulation in Cancer | No | Irrelevant |
| 736 | | Yarahmadi et al., 2024 | | | Therapeutic Bacteria & Viruses in Cancer Therapy | No | Irrelevant |
| 737 | | Zhang et al., 2024 | | | RNA-Binding Proteins in Tumor Microenvironment of CRC | No | - |
| 738 | | Frentzas et al., 2024 | | | Phase 1 Study of Ivonescimab (AK112/SMT112) | No | - |
| 739 | | Fan et al., 2024 | | | Role of Gut Microbiota in CRC Metastasis | No | - |
| 740 | | Pennel et al., 2024 | | | Radiation & Targeted Therapy Combinations for Rectal Cancer | No | - |
| 741 | | Wilbur et al., 2024 | | | Immunotherapy of MSI Cancer | No | - |
| 742 | | Kiran et al., 2024 | | | Precision Medicine in CRC: Molecular Profiling to Targeted Therapy | No | - |
| 743 | | de Boer et al., 2024 | | | ER Stress & Unfolded Protein Response in GI Carcinogenesis | No | Irrelevant |
| 744 | | Bowen et al., 2024 | | | Vaccine Development for Hereditary CRC Syndromes | No | - |
| 745 | | Lin et al., 2024 | | | Genes Limiting T Cell Fitness in Cancer | No | Irrelevant |
| 746 | | Wasson et al., 2024 | | | LncRNA Expression & Cancer-Promoting Effects in Breast Cancer | No | Irrelevant |
| 747 | | Shadbash et al., 2024 | | | Polyomaviruses & GI Complications | No | Irrelevant |
| 748 | | Ryu et al., 2024 | | | 2023 Korean Guidelines for CRC Diagnosis & Treatment | No | Irrelevant |
| 749 | | Pieniądz et al., 2024 | | | CRC Microenvironment & Therapeutic Possibilities | No | - |
| 750 | | Ayabe et al., 2024 | | | Microbiome's Role in CRC Metastasis | No | - |
| 751 | | Sanguedolce et al., 2024 | | | PD-L1 Prognostic Biomarker in Bladder Cancer | No | Irrelevant |
| 752 | | Ibrahim et al., 2024 | | | Modulating Tumor Microenvironment for BCG Immunotherapy | No | Irrelevant |
| 753 | | Patel et al., 2024 | | | Molecular Landscape & Therapeutic Strategies in CRC | No | - |
| 754 | | Radu et al., 2024 | | | Cancer Stem Cells in CRC | No | - |
| 755 | | An et al., 2024 | | | Immune Checkpoint Expression by MSI Status in CRC | No | - |
| 756 | | Ulanja et al., 2024 | | | 45-Year SEER Analysis of Left vs Right-Sided Colon Cancer | No | Irrelevant |
| 757 | | Sullivan et al., 2024 | | | Macrophage Gene Signature in Gastric Cancer | No | Irrelevant |
| 758 | | Reshkin et al., 2024 | | | Genetic Signature of Pancreatic Cancer | No | Irrelevant |
| 759 | | MacLean et al., 2024 | | | Tumor-Promoting Factors & Pathways in Solid Tumors | No | Irrelevant |
| 760 | | Petrella et al., 2024 | | | CRC Pulmonary Metastasectomy | No | Irrelevant |
| 761 | | Haugh et al., 2024 | | | Targeted DNA Sequencing of Cutaneous Melanoma | No | Irrelevant |
| 762 | | Wong et al., 2024 | | | Surgical Excision & Radiotherapy for Brain Metastasis from CRC | No | Irrelevant |
| 763 | | Fountzilas et al., 2024 | | | Tumor-Agnostic Basket Trials & Real-World Data in Oncology | No | Irrelevant |
| 764 | | Abdullaev et al., 2024 | | | Dual Role of LncRNA Metastasis Suppressor-1 in Cancer | No | Irrelevant |
| 765 | | Jani et al., 2024 | | | Immunotherapy Biomarkers in Renal Cell Carcinoma | No | Irrelevant |
| 766 | | Johnson et al., 2024 | | | Advances in Targeted Therapy for Metastatic CRC | No | - |
| 767 | | Klein et al., 2024 | | | Bispecific Antibodies for Cancer Therapy | No | Irrelevant |
| 768 | | Zhang et al., 2024 | | | Molecular Classification & Sensitivity to Therapy in Lung Adenocarcinoma | No | Irrelevant |
| 769 | | Li et al., 2024 | | | ICI & TKI Combination Therapy in CRC | No | - |
| 770 | | Dai et al., 2024 | | | Microbiome in Cancer: Biomarkers & Therapeutic Targets | No | - |
| 771 | | Coffey et al., 2024 | | | Metabolic Alterations in Renal Cell Carcinoma | No | Irrelevant |
| 772 | | Urganci et al., 2024 | | | DNA Mismatch Repair Gene in Pancreatic Adenocarcinoma | No | Irrelevant |
| 773 | | Pan et al., 2024 | | | Zinc-Mediated SMAD4 Upregulation in CRC | No | Irrelevant |
| 774 | | Cordani et al., 2024 | | | EMT, Autophagy & Immune Checkpoints in Cancer | No | Irrelevant |
| 775 | | Swanton et al., 2024 | | | Cancer Complexity & Systemic Disease Hallmarks | No | Irrelevant |
| 776 | | Geng et al., 2024 | | | NK Cell Therapy with XELOX in Stage III CRC | No | - |
| 777 | | Naik et al., 2024 | | | Cancer Testis Antigens as Therapeutic Targets | No | Irrelevant |
| 778 | | Van Den Heuvel et al., 2024 | | | Serum Tumor Markers for Lung Cancer Immunotherapy | No | Irrelevant |
| 779 | | Gao et al., 2024 | | | Traditional Chinese Medicine in Tumor Microenvironment | No | Irrelevant |
| 780 | | Depietro et al., 2024 | | | Chemoembolization Beyond Hepatocellular Carcinoma | No | Irrelevant |
| 781 | | Gutman et al., 2024 | | | SBRT for Liver Tumors | No | Irrelevant |
| 782 | | Sarrett et al., 2024 | | | CD133 as a Radiotheranostic Target in SCLC | No | Irrelevant |
| 783 | | Lee et al., 2024 | | | TIMP3's Role in Oncology | No | Irrelevant |
| 784 | | Leiphrakpam et al., 2024 | | | PI3K/Akt/mTOR Pathway in CRC Treatment | No | - |
| 785 | | Cao et al., 2024 | | | Epigenetic Alteration in CRC | No | - |
| 786 | | Longo et al., 2024 | | | SMARCA4-Deficient Thoracic Tumors | No | Irrelevant |
| 787 | | Bryushkova et al., 2024 | | | B Cell Clonality in Cancer | No | Irrelevant |
| 788 | | Sun et al., 2024 | | | Bimetallic Coordination Polymers in Biomedicine | No | Irrelevant |
| 789 | | Roa et al., 2024 | | | BRAF Mutations in Solid Tumors | No | Irrelevant |
| 790 | | Kuo et al., 2024 | | | Cimetidine Modulation of Tumor Microenvironment in CRC | No | Irrelevant |
| 791 | | Jiang et al., 2024 | | | Decreased interleukin-17RA expression is associated with good prognosis in patients with colorectal cancer and inhibits tumor growth and vascularity in mice | No | Irrelevant |
| 792 | | Zhong et al., 2024 | | | The Therapeutic Potential of Cytokine-Induced Killer in Patients with Cancer | No | Irrelevant |
| 793 | | Philchenkov et al., 2024 | | | Cancer Stem Cells as a Therapeutic Target: Current Clinical Development and Future Prospective | No | Irrelevant |
| 794 | | Awasthi et al., 2024 | | | Neutrophils at the Crossroads: Unraveling the Multifaceted Role in the Tumor Microenvironment | No | Irrelevant |
| 795 | | Dal Buono et al., 2024 | | | Lynch Syndrome: From Multidisciplinary Management to Precision Prevention | No | Irrelevant |
| 796 | | Ren et al., 2024 | | | PIK3CA mutation-driven immune signature as a prognostic marker for evaluating the tumor immune microenvironment and therapeutic response in breast cancer | No | Irrelevant |
| 797 | | Albuquerque et al., 2024 | | | Loss of RAS Mutations in Liquid Biopsies of Patients With Multi-Treated Metastatic Colorectal Cancer | No | Irrelevant |
| 798 | | Savardekar et al., 2024 | | | Single-Cell RNA-Seq Analysis of Patient Myeloid-Derived Suppressor Cells and the Response to Inhibition of Bruton's Tyrosine Kinase | No | Irrelevant |
| 799 | | Yadav et al., 2024 | | | Innovative Nanoparticulate Strategies in Colon Cancer Treatment: A Paradigm Shift | No | Irrelevant |
| 800 | | Ho et al., 2024 | | | Colorectal neuroendocrine carcinoma and mixed neuroendocrine-non-neuroendocrine neoplasm: Prognostic factors and PD-L1 expression | No | Irrelevant |
| 801 | | Le et al., 2024 | | | Immunohistochemical detection of cancer genetic abnormalities | No | Irrelevant |
| 802 | | Hakami et al., 2024 | | | Biomarker discovery and validation for gastrointestinal tumors: A comprehensive review of colorectal, gastric, and liver cancers | No | Irrelevant |
| 803 | | Tan et al., 2024 | | | STING signalling compensates for low tumour mutation burden to drive anti-tumour immunity | No | Irrelevant |
| 804 | | Karjula et al., 2024 | | | Multiplexed analysis of macrophage polarisation in pulmonary metastases of microsatellite stable colorectal cancer | No | Irrelevant |
| 805 | | Ciardiello et al., 2024 | | | The role of anti-EGFR rechallenge in metastatic colorectal cancer, from available data to future developments: A systematic review | No | Irrelevant |
| 806 | | De Silva et al., 2024 | | | Immunogenic cell death in colorectal cancer: a review of mechanisms and clinical utility | No | Irrelevant |
| 807 | | Di Modugno et al., 2024 | | | Tumoral and stromal hMENA isoforms impact tertiary lymphoid structure localization in lung cancer and predict immune checkpoint blockade response in patients with cancer | No | Irrelevant |
| 808 | | Saadh et al., 2024 | | | Deciphering the functional landscape and therapeutic implications of noncoding RNAs in the TGF-β signaling pathway in colorectal cancer: A comprehensive review | No | Irrelevant |
| 809 | | Song et al., 2024 | | | Cell fate regulation governed by p53: Friends or reversible foes in cancer therapy | No | Irrelevant |
| 810 | | Gouda et al., 2024 | | | Liquid Biopsy Response Evaluation Criteria in Solid Tumors (LB-RECIST) | No | Irrelevant |
| 811 | | Shaw et al., 2024 | | | VEGF signaling: Role in angiogenesis and beyond | No | Irrelevant |
| 812 | | Liu et al., 2024 | | | Advances and prospects of mRNA vaccines in cancer immunotherapy | No | Irrelevant |
| 813 | | Nowak et al., 2024 | | | Predictive and prognostic biomarkers in gastrointestinal tract tumours | No | Irrelevant |
| 814 | | Patton et al., 2024 | | | Current updates in sarcoma biomarker discovery: emphasis on next-generation sequencing-based methods | No | Irrelevant |
| 815 | | Attia et al., 2024 | | | HLA class II polymorphisms as prognostic biomarkers for right and left-sided colon cancer | No | Irrelevant |
| 816 | | Chen et al., 2024 | | | Nanotherapeutic derived from multiple bioactive ingredients of natural products: A robust tool for the development of traditional medicine | No | Irrelevant |
| 817 | | Arantes et al., 2024 | | | Unveiling the role of MGMT and DAPK hypermethylation in response to anti-EGFR agents: Molecular insights for advancing HNSCC treatment | No | Irrelevant |
| 818 | | Som et al., 2024 | | | Percutaneous Intratumoral Immunoadjuvant Gel Increases the Abscopal Effect of Cryoablation for Checkpoint Inhibitor Resistant Cancer | No | Irrelevant |
| 819 | | Du et al., 2024 | | | Transcriptome analysis of tertiary lymphoid structures (TLSs)-related genes reveals prognostic value and immunotherapeutic potential in cancer | No | Irrelevant |
| 820 | | Pooyan et al., 2024 | | | Imaging of abdominopelvic oncologic emergencies | No | Irrelevant |
| 821 | | Mahanti et al., 2024 | | | Rough neighborhood: Intricacies of cancer stem cells and infiltrating immune cell interaction in tumor microenvironment and potential in therapeutic targeting | No | Irrelevant |
| 822 | | Nofi et al., 2024 | | | NCCN Guideline Concordance Improves Survival in Pediatric and Young Adult Rectal Cancer | No | Irrelevant |
| 823 | | Miyashita et al., 2024 | | | Immune checkpoint status and oncogenic mutation profiling of rectal cancer after neoadjuvant chemotherapy (KSCC1301-A2) | No | Irrelevant |
| 824 | | Tabasi et al., 2024 | | | Transitional Insight into the RNA-Based Oligonucleotides in Cancer Treatment | No | Irrelevant |
| 825 | | Nieto-Gómez et al., 2024 | | | Analysis of oncological drugs authorised in Spain in the last decade: association between clinical benefit and reimbursement | No | Irrelevant |
| 826 | | Zhao et al., 2024 | | | The efficacy-associated biomarkers for immune checkpoint inhibitors in gastrointestinal cancer: a literature review | No | Irrelevant |
| 827 | | Zhou et al., 2024 | | | Regional delivery of mesothelin-targeted chimeric antigen receptor T-cell effectively and safely targets colorectal cancer liver metastases in mice | No | Irrelevant |
| 828 | | Hsieh et al., 2024 | | | Enhanced antitumour response of gold nanostar-mediated photothermal therapy in combination with immunotherapy in a mouse model of colon carcinoma | No | Irrelevant |
| 829 | | Banerjee et al., 2024 | | | Recent Contributions of Mass Spectrometry-Based “Omics” in the Studies of Breast Cancer | No | Irrelevant |
| 830 | | Deng et al., 2024 | | | Nitrated T cell epitope linked vaccine targeting CD47 elicits antitumor immune responses and acts synergistically with vaccine targeting PDL1 | No | Irrelevant |
| 831 | | Eberly et al., 2024 | | | Current and Emerging Diagnostic, Prognostic, and Predictive Biomarkers in Head and Neck Cancer | No | Irrelevant |
| 832 | | Avgoustakis et al., 2024 | | | Biomaterial-Based Responsive Nanomedicines for Targeting Solid Tumor Microenvironments | No | Irrelevant |
| 833 | | Huang et al., 2024 | | | Clinical features and mutation analysis of class 1/2/3 BRAF mutation colorectal cancer | No | Irrelevant |
| 834 | | Shan et al., 2024 | | | Molecular Targeting of the Phosphoinositide-3-Protein Kinase (PI3K) Pathway across Various Cancers | No | Irrelevant |
| 835 | | Dong et al., 2024 | | | The Role of SPEN Mutations as Predictive Biomarkers for Immunotherapy Response in Colorectal Cancer: Insights from a Retrospective Cohort Analysis | No | Irrelevant |
| 836 | | Papavassiliou et al., 2024 | | | Novel Therapeutic Approaches for Colorectal Cancer Treatment | No | Irrelevant |
| 837 | | Radhakrishnan et al., 2024 | | | Circulating Tumor Cells: How Far Have We Come with Mining These Seeds of Metastasis? | No | Irrelevant |
| 838 | | Cornice et al., 2024 | | | NF-κB: Governing Macrophages in Cancer | No | Irrelevant |
| 839 | | Abdelrahman et al., 2024 | | | Immunohistochemical Expression of Immune Checkpoints; CTLA-4, LAG3, and TIM-3 in Cancer Cells and Tumor-infiltrating Lymphocytes (TILs) in Colorectal Carcinoma | No | Irrelevant |
| 840 | | Feustel et al., 2024 | | | B7-H3 Inhibitors in Oncology Clinical Trials: A Review | No | Irrelevant |
| 841 | | Underwood et al., 2024 | | | Update on Targeted Therapy and Immunotherapy for Metastatic Colorectal Cancer | No | Irrelevant |
| 842 | | Das et al., 2024 | | | Combined Immunotherapy Improves Outcome for Replication-Repair-Deficient (RRD) High-Grade Glioma Failing Anti-PD-1 Monotherapy: A Report from the International RRD Consortium | No | Irrelevant |
| 843 | | Vivaldi et al., 2024 | | | Mismatch Repair Deficiency in Biliary Tract Cancer: Prognostic Implications and Correlation with Histology | No | Irrelevant |
| 844 | | Houdek et al., 2024 | | | Whole Blood Metal Levels in the Setting of an Oncologic Endoprosthesis: Is There Cause for Concern? | No | Irrelevant |
| 845 | | Huang et al., 2024 | | | Targeting ENPP1 for cancer immunotherapy: Killing two birds with one stone | No | Irrelevant |
| 846 | | Guo et al., 2024 | | | Microbiomes in pancreatic cancer can be an accomplice or a weapon | No | Irrelevant |
| 847 | | Torresan et al., 2024 | | | Liquid biopsy in colorectal cancer: Onward and upward | No | Irrelevant |
| 848 | | Leibold et al., 2024 | | | Somatic mouse models of gastric cancer reveal genotype-specific features of metastatic disease | No | Irrelevant |
| 849 | | Chai et al., 2024 | | | ETV7 promotes colorectal cancer progression through upregulation of IFIT3 | No | Irrelevant |
| 850 | | Xiong et al., 2024 | | | Glioblastoma vaccines: past, present, and opportunities | No | Irrelevant |
| 851 | | Lasser et al., 2024 | | | Myeloid-derived suppressor cells in cancer and cancer therapy | No | Irrelevant |
| 852 | | Wang et al., 2024 | | | A comprehensive analysis of immunotherapy in advanced endometrial cancer | No | Irrelevant |
| 853 | | Doi et al., 2024 | | | Correlation of Vein-Rich Tumor Microenvironment of Intrahepatic Cholangiocarcinoma With Tertiary Lymphoid Structures and Patient Outcome | No | Irrelevant |
| 854 | | Shi et al., 2024 | | | Luteolin, a flavone ingredient: Anticancer mechanisms, combined medication strategy, pharmacokinetics, clinical trials, and pharmaceutical researches | No | Irrelevant |
| 855 | | Chen et al., 2024 | | | Anti-PD-1/PD-L1 therapy for colorectal cancer: Clinical implications and future considerations | No | Irrelevant |
| 856 | | García-Díaz et al., 2024 | | | Small molecule inhibitors targeting regulatory T cells for cancer treatment | No | Irrelevant |
| 857 | | Romagnoli et al., 2024 | | | CD8+CD103+PD1+TIM3+ T cells in glioblastoma microenvironment correlate with prognosis | No | Irrelevant |
| 858 | | Mathavan et al., 2024 | | | Clinical Presentation and Targeted Interventions in Urachal Adenocarcinoma: A Single-Institution Case Series and Review of Emerging Therapies | No | Irrelevant |
| 859 | | Riedinger et al., 2024 | | | Characterization of mismatch-repair/microsatellite instability-discordant endometrial cancers | No | Irrelevant |
| 860 | | Liu et al., 2024 | | | Regulator of G protein signaling-1 regulates immune infiltration and macrophage polarization in clear cell renal cell carcinoma | No | Irrelevant |
| 861 | | Eberly et al., 2024 | | | Current and Emerging Diagnostic, Prognostic, and Predictive Biomarkers in Head and Neck Cancer | No | Irrelevant |
| 862 | | Avgoustakis et al., 2024 | | | Biomaterial-Based Responsive Nanomedicines for Targeting Solid Tumor Microenvironments | No | Irrelevant |
| 863 | | Huang et al., 2024 | | | Clinical features and mutation analysis of class 1/2/3 BRAF mutation colorectal cancer | No | Irrelevant |
| 864 | | Shan et al., 2024 | | | Molecular Targeting of the Phosphoinositide-3-Protein Kinase (PI3K) Pathway across Various Cancers | No | Irrelevant |
| 865 | | Dong et al., 2024 | | | The Role of SPEN Mutations as Predictive Biomarkers for Immunotherapy Response in Colorectal Cancer: Insights from a Retrospective Cohort Analysis | No | Irrelevant |
| 866 | | Papavassiliou et al., 2024 | | | Novel Therapeutic Approaches for Colorectal Cancer Treatment | No | Irrelevant |
| 867 | | Radhakrishnan et al., 2024 | | | Circulating Tumor Cells: How Far Have We Come with Mining These Seeds of Metastasis? | No | Irrelevant |
| 868 | | Cornice et al., 2024 | | | NF-κB: Governing Macrophages in Cancer | No | Irrelevant |
| 869 | | Abdelrahman et al., 2024 | | | Immunohistochemical Expression of Immune Checkpoints; CTLA-4, LAG3, and TIM-3 in Cancer Cells and Tumor-infiltrating Lymphocytes (TILs) in Colorectal Carcinoma | No | Irrelevant |
| 870 | | Feustel et al., 2024 | | | B7-H3 Inhibitors in Oncology Clinical Trials: A Review | No | Irrelevant |
| 871 | | Underwood et al., 2024 | | | Update on Targeted Therapy and Immunotherapy for Metastatic Colorectal Cancer | No | Irrelevant |
| 872 | | Das et al., 2024 | | | Combined Immunotherapy Improves Outcome for Replication-Repair-Deficient (RRD) High-Grade Glioma Failing Anti-PD-1 Monotherapy | No | Irrelevant |
| 873 | | Vivaldi et al., 2024 | | | Mismatch Repair Deficiency in Biliary Tract Cancer: Prognostic Implications and Correlation with Histology | No | Irrelevant |
| 874 | | Houdek et al., 2024 | | | Whole Blood Metal Levels in the Setting of an Oncologic Endoprosthesis: Is There Cause for Concern? | No | Irrelevant |
| 875 | | Huang et al., 2024 | | | Targeting ENPP1 for cancer immunotherapy: Killing two birds with one stone | No | Irrelevant |
| 876 | | Guo et al., 2024 | | | Microbiomes in pancreatic cancer can be an accomplice or a weapon | No | Irrelevant |
| 877 | | Torresan et al., 2024 | | | Liquid biopsy in colorectal cancer: Onward and upward | No | Irrelevant |
| 878 | | Leibold et al., 2024 | | | Somatic mouse models of gastric cancer reveal genotype-specific features of metastatic disease | No | Irrelevant |
| 879 | | Chai et al., 2024 | | | ETV7 promotes colorectal cancer progression through upregulation of IFIT3 | No | Irrelevant |
| 880 | | Xiong et al., 2024 | | | Glioblastoma vaccines: past, present, and opportunities | No | Irrelevant |
| 881 | | Lasser et al., 2024 | | | Myeloid-derived suppressor cells in cancer and cancer therapy | No | Irrelevant |
| 882 | | Wang et al., 2024 | | | A comprehensive analysis of immunotherapy in advanced endometrial cancer | No | Irrelevant |
| 883 | | Doi et al., 2024 | | | Correlation of Vein-Rich Tumor Microenvironment of Intrahepatic Cholangiocarcinoma With Tertiary Lymphoid Structures and Patient Outcome | No | Irrelevant |
| 884 | | Shi et al., 2024 | | | Luteolin, a flavone ingredient: Anticancer mechanisms, combined medication strategy, pharmacokinetics, clinical trials, and pharmaceutical researches | No | Irrelevant |
| 885 | | Chen et al., 2024 | | | Anti-PD-1/PD-L1 therapy for colorectal cancer: Clinical implications and future considerations | No | Irrelevant |
| 886 | | García-Díaz et al., 2024 | | | Small molecule inhibitors targeting regulatory T cells for cancer treatment | No | Irrelevant |
| 887 | | Romagnoli et al., 2024 | | | CD8+CD103+PD1+TIM3+ T cells in glioblastoma microenvironment correlate with prognosis | No | Irrelevant |
| 888 | | Mathavan et al., 2024 | | | Clinical Presentation and Targeted Interventions in Urachal Adenocarcinoma: A Single-Institution Case Series and Review of Emerging Therapies | No | Irrelevant |
| 889 | | Riedinger et al., 2024 | | | Characterization of mismatch-repair/microsatellite instability-discordant endometrial cancers | No | Irrelevant |
| 890 | | Liu et al., 2024 | | | Regulator of G protein signaling-1 regulates immune infiltration and macrophage polarization in clear cell renal cell carcinoma | No | Irrelevant |
| 891 | | Damane et al., 2024 | | | Immunotherapy and immunomodulation in colorectal cancer treatment resistance | No | Irrelevant |
| 892 | | Duan et al., 2024 | | | VISTA in hematological malignancies: a review of the literature | No | Irrelevant |
| 893 | | Patny et al., 2024 | | | The RAS competitive landscape | No | Irrelevant |
| 894 | | Liao et al., 2024 | | | Mevalonate kinase inhibits anti-tumor immunity by impairing the tumor cell-intrinsic interferon response in microsatellite instability colorectal cancer | No | Irrelevant |
| 895 | | Deza, 2024 | | | Improving Clinical Communication: A Clinician’s Guide to Building Better Skills and Patient Outcomes | No | Irrelevant |
| 896 | | Bai et al., 2024 | | | TGF-β in tumor microenvironment, metabolism, and immunotherapy | No | Irrelevant |
| 897 | | Wang et al., 2024 | | | FTO activates PD-L1 promotes immunosuppression in breast cancer via the m6A/YTHDF3/PDK1 axis under hypoxic conditions | No | Irrelevant |
| 898 | | Yao et al., 2024 | | | Construction and validation of a regulatory T cells-based classification of renal cell carcinoma: an integrated bioinformatic analysis and clinical cohort study | No | Irrelevant |
| 899 | | Karami et al., 2024 | | | The emerging role of TIM-3 in colorectal cancer: a promising target for immunotherapy | No | Irrelevant |
| 900 | | Zou et al., 2024 | | | Microbiome in urologic neoplasms: focusing on tumor immunity | No | Irrelevant |
| 901 | | Fan et al., 2024 | | | Biomarkers and potential therapeutic targets driving progression of non-alcoholic steatohepatitis to hepatocellular carcinoma predicted through transcriptomic analysis | No | Irrelevant |
| 902 | | Wu et al., 2024 | | | A real-world study: third-line treatment options for metastatic colorectal cancer | No | - |
| 903 | | Xu et al., 2024 | | | Leveraging the synergy between anti-angiogenic therapy and immune checkpoint inhibitors to treat digestive system cancers | No | Irrelevant |
| 904 | | Zhang et al., 2024 | | | Therapeutic potential of Xihuang Pill in colorectal cancer: Metabolomic and microbiome-driven approaches | No | Irrelevant |
| 905 | | Babič et al., 2024 | | | B7-H3 in glioblastoma and beyond: significance and therapeutic strategies | No | Irrelevant |
| 906 | | Yan et al., 2024 | | | Gut microbiota as a biomarker and modulator of anti-tumor immunotherapy outcomes | No | Irrelevant |
| 907 | | Clabeaux et al., 2024 | | | Oculofacial plastic surgery in the cancer patient: A narrative review | No | Irrelevant |
| 908 | | Ma et al., 2024 | | | Microbiome engineering and cancer therapy | No | Irrelevant |
| 909 | | Pourali et al., 2024 | | | Cancer precision medicine: Focus on gastrointestinal cancer | No | Irrelevant |
| 910 | | Zhang et al., 2024 | | | Current Status and Research Progress of Immunotherapy for Colorectal Cancer | No | - |
| 911 | | Shamim, 2024 | | | Overview of the current standards in rectal carcinoma treatment | No | Irrelevant |
| 912 | | Cullum et al., 2024 | | | Case report: Dramatic impact of DNA next generation sequencing results using specific targeted therapies—ALK and PIK3CA | No | Irrelevant |
| 913 | | Xu et al., 2024 | | | Progress on angiogenic and antiangiogenic agents in the tumor microenvironment | No | Irrelevant |
| 914 | | Huang et al., 2024 | | | The oral-gut microbiome axis in breast cancer: from basic research to therapeutic applications | No | Irrelevant |
| 915 | | Saxena et al., 2024 | | | Targeting regulated cell death pathways in cancers for effective treatment: a comprehensive review | No | Irrelevant |
| 916 | | Elewaut et al., 2024 | | | Cancer cells impair monocyte-mediated T cell stimulation to evade immunity | No | Irrelevant |
| 917 | | Akshatha et al., 2024 | | | New Insights on Anti-Colorectal Cancer Effects of Plant-Based Bioactive Compounds | No | Irrelevant |
| 918 | | Zhou et al., 2024 | | | Nuclear Molecular Imaging for Evaluating T Cell Exhaustion | No | Irrelevant |
| 919 | | AlDoughaim et al., 2024 | | | Cancer Biomarkers and Precision Oncology: A Review of Recent Trends and Innovations | No | Irrelevant |
| 920 | | Jiang et al., 2024 | | | CES1 is associated with cisplatin resistance and poor prognosis of head and neck squamous cell carcinoma | No | Irrelevant |
| 921 | | Wu et al., 2024 | | | Comparison of differences in immune cells and immune microenvironment among different kinds of oncolytic virus treatments | No | Irrelevant |
| 922 | | Xu et al., 2024 | | | Immunotherapy for Colorectal Cancer | No | - |
| 923 | | Li et al., 2024 | | | Radiomics in precision medicine for colorectal cancer: a bibliometric analysis (2013–2023) | No | Irrelevant |
| 924 | | Wang et al., 2024 | | | The role of PIK3CA gene mutations in colorectal cancer and the selection of treatment strategies | No | Irrelevant |
| 925 | | Nandi et al., 2024 | | | Integrating immunotherapy with conventional treatment regime for breast cancer patients- an amalgamation of armamentarium | No | Irrelevant |
| 926 | | Liu et al., 2024 | | | Unveiling the Power of Gut Microbiome in Predicting Neoadjuvant Immunochemotherapy Responses in Esophageal Squamous Cell Carcinoma | No | Irrelevant |
| 927 | | Gao et al., 2024 | | | Organ Crosstalk: The Role of Spleen | No | Irrelevant |
| 928 | | Chang et al., 2024 | | | Insights into the historical trajectory and research trends of immune checkpoint blockade in colorectal cancer: visualization and bibliometric analysis | No | Irrelevant |
| 929 | | Chou, 2024 | | | Mass-Action Law Dynamics Theory and Algorithm for Translational and Precision Medicine Informatics | No | Irrelevant |
| 930 | | Shimizu et al., 2024 | | | First-in-human phase 1 dose-escalation results with livmoniplimab, an antibody targeting the GARP:TGF-ß1 complex, as monotherapy and in combination with the anti–PD-1 antibody budigalimab in patients with advanced solid tumors | No | Irrelevant |
| 931 | | Li et al., 2024 | | | Oncolytic immunotherapy with nivolumab in muscle-invasive bladder cancer: a phase 1b trial | No | Irrelevant |
| 932 | | Li et al., 2024 | | | Lung enteric-type adenocarcinoma with gastric metastasis: a rare case report and literature review | No | Irrelevant |
| 933 | | Requesens et al., 2024 | | | Genomic instability as a driver and suppressor of anti-tumor immunity | No | Irrelevant |
| 934 | | Almeida et al., 2024 | | | Peripheral immune profiling of soft tissue sarcoma: perspectives for disease monitoring | No | Irrelevant |
| 935 | | Yu et al., 2024 | | | Identification and validation of a prognostic model based on immune-related genes in ovarian carcinoma | No | Irrelevant |
| 936 | | Rompen et al., 2024 | | | Definition and Predictors of Early Recurrence in Neoadjuvantly Treated Esophageal and Gastroesophageal Adenocarcinoma: a Dual-Center Retrospective Cohort Study | No | Irrelevant |
| 937 | | Feakins, 2024 | | | Inflammatory disorders of the large intestine | No | Irrelevant |
| 938 | | Reste et al., 2024 | | | The role of dendritic cells in tertiary lymphoid structures: implications in cancer and autoimmune diseases | No | Irrelevant |
| 939 | | Yu et al., 2024 | | | Prognostic and therapeutic potential of gene profiles related to tertiary lymphoid structures in colorectal cancer | No | Irrelevant |
| 940 | | Yang et al., 2024 | | | A Long-term Survival Risk Prediction Model for Patients with Superficial Esophageal Squamous Cell Carcinoma | No | Irrelevant |
| 941 | | Yang et al., 2024 | | | Establishment and bioinformatics analysis of a four-miRNA prognostic signature for pleural mesothelioma | No | Irrelevant |
| 942 | | Peng et al., 2024 | | | T2WI and ADC radiomics combined with a nomogram based on clinicopathologic features to quantitatively predict microsatellite instability in colorectal cancer | No | Irrelevant |
| 943 | | Gupta et al., 2024 | | | Ophthalmic Signs in Practice of Medicine | No | Irrelevant |
| 944 | | Edwards et al., 2024 | | | Lynch Syndrome | No | - |
| 945 | | Haque et al., 2024 | | | Targeted therapy approaches for epithelial-mesenchymal transition in triple negative breast cancer | No | Irrelevant |
| 946 | | Xu et al., 2024 | | | Targeting STING signaling for the optimal cancer immunotherapy | No | Irrelevant |
| 947 | | Lin et al., 2024 | | | Targeting ACSLs to modulate ferroptosis and cancer immunity | No | Irrelevant |
| 948 | | Mansur et al., 2024 | | | New frontiers in radioembolization | No | Irrelevant |
| 949 | | Zheng et al., 2024 | | | Effects of intratumoral microbiota on tumorigenesis, anti-tumor immunity, and microbe-based cancer therapy | No | Irrelevant |
| 950 | | Tang et al., 2024 | | | The role of SIRT1 in autophagy and drug resistance: unveiling new targets and potential biomarkers in cancer therapy | No | Irrelevant |
| 951 | | Kraus et al., 2024 | | | Pulmonary Adenocarcinoma with Enteric Differentiation without TTF-1 Expression Is a Very Rare Subtype with Limited Treatment Options and Poor Prognosis | No | Irrelevant |
| 952 | | Colloca et al., 2024 | | | The Prognosis of Metastatic Gastrointestinal Cancer | No | Irrelevant |
| 953 | | Ansari et al., 2024 | | | Tumor Mutational Burden as a Biomarker of Immunotherapy Response: An Immunogram Approach in Onco-immunology | No | Irrelevant |
| 954 | | Kim et al., 2024 | | | Gut microbiome therapy: fecal microbiota transplantation vs live biotherapeutic products | No | Irrelevant |
| 955 | | Hourani et al., 2024 | | | Transforming growth factor-β in tumor microenvironment: Understanding its impact on monocytes and macrophages for its targeting | No | Irrelevant |
| 956 | | Wei et al., 2024 | | | Natural plant-derived polysaccharides targeting macrophage polarization: a promising strategy for cancer immunotherapy | No | Irrelevant |
| 957 | | Lu et al., 2024 | | | Metabolic mediators: microbial-derived metabolites as key regulators of anti-tumor immunity, immunotherapy, and chemotherapy | No | Irrelevant |
| 958 | | Komine et al., 2024 | | | Impact of Tumoral β2-Adrenergic Receptor Expression on Chemotherapeutic Response and Prognosis in Patients with Advanced Colorectal Cancer | No | Irrelevant |
| 959 | | González et al., 2024 | | | Personalised medicine based on host genetics and microbiota applied to colorectal cancer | No | Irrelevant |
| 960 | | Hara et al., 2024 | | | Pyroptosis and chemical classification of pyroptotic agents | No | Irrelevant |
| 961 | | Szupryczyński et al., 2024 | | | What is the Reason That the Pharmacological Future of Chemotherapeutics in the Treatment of Lung Cancer Could Be Most Closely Related to Nanostructures? | No | Irrelevant |
| 962 | | Consoli et al., 2024 | | | Navigating heme pathways: the breach of heme oxygenase and hemin in breast cancer | No | Irrelevant |
| 963 | | Kögl et al., 2024 | | | The game-changing impact of POLE mutations in oncology—a review from a gynecologic oncology perspective | No | Irrelevant |
| 964 | | Heidari-Foroozan et al., 2024 | | | The molecular landscape of T cell exhaustion in the tumor microenvironment and reinvigoration strategies | No | Irrelevant |
| 965 | | Feng et al., 2024 | | | Tuberculosis to lung cancer: application of tuberculosis signatures in identification of lung adenocarcinoma subtypes and marker screening | No | Irrelevant |
| 966 | | Lv et al., 2024 | | | Tertiary lymphoid structures in colorectal cancer | No | - |
| 967 | | Iwata et al., 2024 | | | Real-world outcomes of stage II and III colorectal cancers treated by postoperative adjuvant chemotherapy based on the mismatch repair status | No | - |
| 968 | | Jiang et al., 2024 | | | The gut microbiome modulates response to immunotherapy in cancer | No | Irrelevant |
| 969 | | Zheng et al., 2024 | | | Navigating through novelties concerning mCRC treatment—the role of immunotherapy, chemotherapy, and targeted therapy in mCRC | No | - |
| 970 | | Trembath et al., 2024 | | | Gastrointestinal Malignancy: Genetic Implications to Clinical Applications | No | Irrelevant |
| 971 | | Serhal et al., 2024 | | | Locoregional Therapies for Primary and Secondary Hepatic Malignancies | No | Irrelevant |
| 972 | | Cho et al., 2024 | | | Criteria for assessing evidence for biomarker-targeted therapies in rare cancers—an extrapolation framework | No | Irrelevant |
| 973 | | Agnihotri et al., 2024 | | | Autophagy and Multidrug Resistance in Cancer | No | Irrelevant |
| 974 | | Kashyap et al., 2024 | | | Future perspective: identification and validation of biomarkers using liquid biopsy | No | Irrelevant |
| 975 | | Zheng et al., 2024 | | | Efficacy and safety of hepatic arterial infusion chemotherapy combined with fruquintinib and tislelizumab for MSS colorectal cancer liver metastasis | No | - |
| 976 | | Roncato et al., 2024 | | | The challenge of molecular selection in liver-limited metastatic colorectal cancer for surgical resection: a systematic review | No | Irrelevant |
| 977 | | Lu et al., 2024 | | | Consensus on the prevention and management of tyrosine kinase inhibitor-related skin toxicities in NSCLC | No | Irrelevant |
| 978 | | Pei et al., 2024 | | | Efficacy and challenges of anti-PD1 in MSI-H mCRC: a case report on concurrent infections and ir-AIHA | No | - |
| 979 | | Chen et al., 2024 | | | Efficacy of immune checkpoint inhibitors combined with bevacizumab in MSS/pMMR advanced colorectal cancer | No | - |
| 980 | | Chen et al., 2024 | | | PRNP is a pan-cancer prognostic and immunity-related to EMT in colorectal cancer | No | Irrelevant |
| 981 | | Li et al., 2024 | | | Pan-cancer analysis and single-cell analysis reveals FAM110B as a potential target for survival and immunotherapy | No | Irrelevant |
| 982 | | Wu et al., 2024 | | | Antitumor Study of Neoantigen-reactive T Cells Co-expressing IL-7 and CCL19 in Mouse Lung Cancer | No | Irrelevant |
| 983 | | Murakami et al., 2024 | | | The Nectin family ligands, PVRL2 and PVR, in cancer immunology and immunotherapy | No | Irrelevant |
| 984 | | Cao et al., 2024 | | | The Effects of The PD-1/PD-L1 Axis and Its Implications for Immunotherapy in Gastrointestinal Tract Cancers | No | - |
| 985 | | Paniagua-Herranz et al., 2024 | | | Overcoming limitations for antibody-based therapies targeting γδ T (Vg9Vd2) cells | No | Irrelevant |
| 986 | | Martín-García et al., 2024 | | | The use of SP/Neurokinin-1 as a Therapeutic Target in Colon and Rectal Cancer | No | - |
| 987 | | Musaelyan et al., 2024 | | | Response to trametinib, hydroxychloroquine, and bevacizumab in a young woman with NRAS-mutated metastatic intrahepatic cholangiocarcinoma | No | Irrelevant |
| 988 | | Valdivia-Silva et al., 2024 | | | Chemokine receptors and their ligands in breast cancer: The key roles in progression and metastasis | No | Irrelevant |
| 989 | | Nicolini et al., 2024 | | | Involvement of tumor immune microenvironment metabolic reprogramming in colorectal cancer progression, immune escape, and response to immunotherapy | No | - |
| 990 | | Kipkeeva et al., 2024 | | | Immune checkpoints PD-1/PD-L1 and TIM-3/GAL-9 and prospects for their simultaneous inhibition | No | Irrelevant |
| 991 | | Majumder et al., 2024 | | | Mismatch repair-proficient tumor footprints in the sands of immune desert: mechanistic constraints and precision platforms | No | Irrelevant |
| 992 | | Yang et al., 2024 | | | Biological and Clinical Characteristics of Proximal Colon Cancer: Far from Its Anatomical Subsite | No | Irrelevant |
| 993 | | Tadic et al., 2024 | | | Nucleic acid cancer vaccines targeting tumor related angiogenesis. Could mRNA vaccines constitute a game changer? | No | Irrelevant |
| 994 | | Zhu et al., 2024 | | | Developing a machine learning-based prognosis and immunotherapeutic response signature in colorectal cancer: insights from ferroptosis, fatty acid dynamics, and the tumor microenvironment | No | Irrelevant |
| 995 | | Milone, 2024 | | | Therapeutic drug monitoring of selected anticancer drugs | No | Irrelevant |
| 996 | | Yang et al., 2024 | | | Multi-Omics analysis elucidates tumor microenvironment and intratumor microbes of angiogenesis subtypes in colon cancer | No | Irrelevant |
| 997 | | Abbasifard et al., 2024 | | | The story of clobenpropit and CXCR4: can be an effective drug in cancer and autoimmune diseases? | No | Irrelevant |
| 998 | | Karigyo et al., 2024 | | | Cardiac Tumors: Review | No | Irrelevant |
| 999 | | Jiang et al., 2024 | | | Comparison of systemic treatments for previously treated patients with unresectable colorectal liver metastases: a systematic review and network meta-analysis | No | - |
| 1000 | | Hheidari et al., 2024 | | | Metal-based nanoparticle in cancer treatment: lessons learned and challenges | No | Irrelevant |
| 1001 | | Babayan et al., 2024 | | | Failure of immune checkpoint inhibitors for MSI-positive pancreatic adenocarcinoma with atypical STR mutation | No | Irrelevant |
| 1002 | | Karukonda et al., 2024 | | | Radiation Therapy | No | Irrelevant |
| 1003 | | Saris et al., 2024 | | | T-cell responses in colorectal peritoneal metastases are recapitulated in a humanized immune system mouse model | No | Irrelevant |
| 1004 | | Wang et al., 2024 | | | Comprehensive analysis of PPP4C’s impact on prognosis, immune microenvironment, and immunotherapy response in lung adenocarcinoma | No | Irrelevant |
| 1005 | | Langouo Fontsa et al., 2024 | | | Tumor-associated tertiary lymphoid structures in cancer: implications for immunotherapy | No | Irrelevant |
| 1006 | | Gan et al., 2024 | | | The expression of ERAP1 is favorable for prognosis and immunotherapy in colorectal cancer | No | - |
| 1007 | | Cantoni et al., 2024 | | | Human NK cells and cancer | No | Irrelevant |
| 1008 | | Kawada et al., 2024 | | | Sinusoidal cells in liver metastasis | No | Irrelevant |
| 1009 | | Huang et al., 2024 | | | Liver cancer | No | Irrelevant |
| 1010 | | Bui et al., 2024 | | | Polarization of M2 Tumor-Associated Macrophages (TAMs) in Cancer Immunotherapy | No | Irrelevant |
| 1011 | | Zhang et al., 2024 | | | Research Advances of Lipid Nanoparticles in the Treatment of Colorectal Cancer | No | - |
| 1012 | | Sabaghian et al., 2024 | | | The role of PD-1/PD-L1 signaling pathway in cancer pathogenesis and treatment | No | Irrelevant |
| 1013 | | Fu et al., 2024 | | | Elucidating CTLA-4’s role in tumor immunity: a comprehensive overview of targeted antibody therapies and clinical developments | No | Irrelevant |
| 1014 | | Zhang et al., 2024 | | | Evaluating the efficacy and safety of trebananib in treating ovarian and non-ovarian cancer patients | No | Irrelevant |
| 1015 | | Pumpalova et al., 2024 | | | Systemic Therapy for Metastatic Colon Cancer: New Frontiers | No | - |
| 1016 | | Yang et al., 2024 | | | Integrative analysis of blood transcriptome profiles in small-cell lung cancer patients for identification of novel chemotherapy resistance-related biomarkers | No | Irrelevant |
| 1017 | | Cai et al., 2024 | | | A High Immune-Related Index with the Suppression of cGAS-STING Pathway is a Key Determinant to Herceptin Resistance in HER2+ Breast Cancer | No | Irrelevant |
| 1018 | | Bidgood et al., 2024 | | | SOCS1 is a critical checkpoint in immune homeostasis, inflammation, and tumor immunity | No | Irrelevant |
| 1019 | | Tóth et al., 2024 | | | The rapidly changing field of predictive biomarkers of non-small cell lung cancer | No | Irrelevant |
| 1020 | | Qian et al., 2024 | | | Ginsenosides: an immunomodulator for the treatment of colorectal cancer | No | - |
| 1021 | | Yan et al., 2024 | | | Immune checkpoint inhibitors in colorectal cancer: limitation and challenges | No | - |
| 1022 | | Yu et al., 2024 | | | Prognostic and therapeutic insights into colorectal carcinoma through immunogenic cell death gene profiling | No | - |
| 1023 | | Wang et al., 2024 | | | Advances in Foxp3+ regulatory T cells (Foxp3+ Treg) and key factors in digestive malignancies | No | - |
| 1024 | | Normanno et al., 2024 | | | Resistance to immune checkpoint inhibitors in colorectal cancer with deficient MMR/MSI: misdiagnosis, pseudoprogression and/or tumor heterogeneity? | No | - |
| 1025 | | Yang et al., 2024 | | | Concomitant NAFLD Facilitates Liver Metastases and PD-1-Refractory by Recruiting MDSCs via CXCL5/CXCR2 in Colorectal Cancer | No | - |
| 1026 | | Ahmadpour et al., 2024 | | | The effects of tumor-derived supernatants (TDS) on cancer cell progression: A review and update on carcinogenesis and immunotherapy | No | Irrelevant |
| 1027 | | Yang et al., 2024 | | | Minimally invasive intervention combined with targeted immunotherapy achieves complete clinical response in advanced colorectal cancer | No | - |
| 1028 | | Hong et al., 2024 | | | Multiplex analysis for the identification of plasma protein biomarkers for predicting lung cancer immunotherapy response | No | Irrelevant |
| 1029 | | Yang et al., 2024 | | | The efficacy and safety of neoadjuvant chemoradiotherapy combined with immunotherapy for locally advanced rectal cancer | No | - |
| 1030 | | Meng et al., 2024 | | | Advancements in treatment of BRAF V600E-mutant metastatic colorectal cancer | No | - |
| 1031 | | Xu et al., 2024 | | | Exploiting tertiary lymphoid structures gene signature to evaluate tumor microenvironment infiltration and immunotherapy response in colorectal cancer | No | - |
| 1032 | | Zhang et al., 2024 | | | CD112 is an epithelial-to-mesenchymal transition-related and immunological biomarker in pan-cancer | No | Irrelevant |
| 1033 | | Qian et al., 2024 | | | DNA sensing of dendritic cells in cancer immunotherapy | No | Irrelevant |
| 1034 | | Giacalone et al., 2024 | | | The promise, progress, and challenges of in situ immunization agents in cancer immunotherapy | No | Irrelevant |
| 1035 | | Huang et al., 2024 | | | Enhancing immunotherapy outcomes by targeted remodeling of the tumor microenvironment via combined cGAS-STING pathway strategies | No | Irrelevant |
| 1036 | | Li et al., 2024 | | | A novel angiogenesis-associated risk score predicts prognosis and characterizes the tumor microenvironment in colon cancer | No | - |
| 1037 | | De et al., 2024 | | | Recent advancements in immunotherapy for colorectal cancer | No | - |
| 1038 | | Sabale et al., 2024 | | | Novel targeting strategies on signaling pathways of colorectal cancer | No | - |
| 1039 | | Mohapatra et al., 2024 | | | Current drug therapy for colorectal cancer | No | - |
| 1040 | | Arimoto et al., 2024 | | | Emerging role of immunogenic cell death in cancer immunotherapy | No | Irrelevant |
| 1041 | | Biersack et al., 2024 | | | Emerging role of MYB transcription factors in cancer drug resistance | No | Irrelevant |
| 1042 | | Csizmar et al., 2024 | | | Other Immune Checkpoint Targets of Interest | No | Irrelevant |
| 1043 | | González et al., 2024 | | | Epidermal growth factor receptor antagonists in colorectal cancer: emerging strategies for precision therapy | No | Irrelevant |
| 1044 | | Sánchez-Ramírez et al., 2024 | | | Impact of STAT-signaling pathway on cancer-associated fibroblasts in colorectal cancer and its role in immunosuppression | No | - |
| 1045 | | Holzmayer et al., 2024 | | | The bispecific B7H3xCD3 antibody CC-3 induces T cell immunity against bone and soft tissue sarcomas | No | Irrelevant |
| 1046 | | Mondal et al., 2024 | | | Prospects of liquid biopsy in the prognosis and clinical management of gastrointestinal cancers | No | Irrelevant |
| 1047 | | Curry et al., 2024 | | | Belzutifan: a novel therapeutic for the management of von Hippel–Lindau disease and beyond | No | Irrelevant |
| 1048 | | Brand et al., 2024 | | | Eosinophilic granulocytes as a potential prognostic marker for cancer progression and therapeutic response in malignant melanoma | No | Irrelevant |
| 1049 | | Pei et al., 2024 | | | An inflammation-related subtype classification for analyzing tumor microenvironment and clinical prognosis in colorectal cancer | No | - |
| 1050 | | Zhongjie et al., 2024 | | | Developing innovative strategies of tumor‑infiltrating lymphocyte therapy for tumor treatment | No | Irrelevant |
| 1051 | | Bauer et al., 2024 | | | Deciphering the role of alternative splicing in neoplastic diseases for immune-oncological therapies | No | Irrelevant |
| 1052 | | Jiang et al., 2024 | | | An emerging strategy: probiotics enhance the effectiveness of tumor immunotherapy via mediating the gut microbiome | No | Irrelevant |
| 1053 | | Seckinger et al., 2024 | | | Targeting CEACAM5-positive solid tumors using NILK-2401, a novel CEACAM5xCD47 κλ bispecific antibody | No | Irrelevant |
| 1054 | | Constantin et al., 2024 | | | Microbiome and cancer: from mechanistic implications in disease progression and treatment to development of novel antitumoral strategies | No | Irrelevant |
| 1055 | | Alemzadeh et al., 2024 | | | Deciphering resistance mechanisms and novel strategies to overcome drug resistance in ovarian cancer: a comprehensive review | No | Irrelevant |
| 1056 | | Pereira et al., 2024 | | | Tissue adaptation of CD4 T lymphocytes in homeostasis and cancer | No | Irrelevant |
| 1057 | | Chen et al., 2024 | | | Tertiary lymphoid structures in cancer: maturation and induction | No | - |
| 1058 | | Gao et al., 2024 | | | Research progress and applications of epigenetic biomarkers in cancer | No | Irrelevant |
| 1059 | | Saowapa et al., 2024 | | | Immunotherapy-induced colitis in metastatic colorectal cancer: a systematic review and meta-analysis | No | - |
| 1060 | | Han et al., 2024 | | | A distinct tumor microenvironment makes anaplastic thyroid cancer more lethal but immunotherapy sensitive than papillary thyroid cancer | No | Irrelevant |
| 1061 | | Schmid et al., 2024 | | | The interplay between autophagy and cGAS-STING signaling and its implications for cancer | No | Irrelevant |
| 1062 | | Nádorvári et al., 2024 | | | Microsatellite instability and mismatch repair protein deficiency: equal predictive markers? | No | Irrelevant |
| 1063 | | Yu et al., 2024 | | | Influence of Microbiota on Tumor Immunotherapy | No | Irrelevant |
| 1064 | | Abdullah et al., 2024 | | | Molecular Pathogenesis, Organ Metastasis, and Targeted Therapy for Non-Small-Cell Lung Cancer | No | Irrelevant |
| 1065 | | Pouyamanesh et al., 2024 | | | Thymol Enhances 5-Fluorouracil Cytotoxicity by Reducing Migration and Increasing Apoptosis and Cell Cycle Arrest in Esophageal Cancer Cells | No | Irrelevant |
| 1066 | | Foo et al., 2024 | | | Metastatic colorectal cancer - third line therapy and beyond | No | - |
| 1067 | | Stucchi et al., 2024 | | | Fruquintinib as new treatment option in metastatic colorectal cancer patients: is there an optimal sequence? | No | Irrelevant |
| 1068 | | Salnikov et al., 2024 | | | The viral etiology of EBV-associated gastric cancers contributes to their unique pathology, clinical outcomes, treatment responses and immune landscape | No | Irrelevant |
| 1069 | | Sharma et al., 2024 | | | Molecular insights into clinical trials for immune checkpoint inhibitors in colorectal cancer | No | - |
| 1070 | | Kita et al., 2024 | | | Recent clinical trials and optical control as a potential strategy to develop microtubule-targeting drugs in colorectal cancer management | No | Irrelevant |
| 1071 | | Procházková et al., 2024 | | | Aryl hydrocarbon receptor as a drug target in advanced prostate cancer therapy–obstacles and perspectives | No | Irrelevant |
| 1072 | | Scheuermann et al., 2024 | | | Unveiling spatial complexity in solid tumor immune microenvironments through multiplexed imaging | No | Irrelevant |
| 1073 | | Sadien et al., 2024 | | | Genomics, Histopathology, and Molecular Pathology of Sporadic and Hereditary Colorectal Cancer | No | - |
| 1074 | | Gulturk et al., 2024 | | | Naples prognostic score may predict overall survival in metastatic pancreatic cancer | No | Irrelevant |
| 1075 | | Pellitero et al., 2024 | | | Predictors of Immunotherapy Efficacy in Metastatic Non-Small Cell Lung Cancer | No | Irrelevant |
| 1076 | | Zhu et al., 2024 | | | Gastric cancer with brain metastasis: from molecular characteristics and treatment | No | Irrelevant |
| 1077 | | Liu et al., 2024 | | | Role of the gut microbiota in tumorigenesis and treatment | No | Irrelevant |
| 1078 | | Shebbo et al., 2024 | | | Redefining the battle against colorectal cancer: a comprehensive review of emerging immunotherapies and their clinical efficacy | No | - |
| 1079 | | Kuznetsova et al., 2024 | | | Prognostic and predictive role of immune microenvironment in colorectal cancer | No | - |
| 1080 | | Zou et al., 2024 | | | Colorectal medullary carcinoma: a pathological subtype with intense immune response and potential to benefit from immune checkpoint inhibitors | No | - |
| 1081 | | Qin et al., 2024 | | | Biomarkers and computational models for predicting efficacy to tumor ICI immunotherapy | No | Irrelevant |
| 1082 | | Chen et al., 2024 | | | Tyrosine phosphatase PTPN11/SHP2 in solid tumors - bull’s eye for targeted therapy? | No | Irrelevant |
| 1083 | | Cho et al., 2024 | | | Exploring histological predictive biomarkers for immune checkpoint inhibitor therapy response in non–small cell lung cancer | No | Irrelevant |
| 1084 | | Tindall et al., 2024 | | | The TGF-β superfamily as potential therapeutic targets in pancreatic cancer | No | Irrelevant |
| 1085 | | Ou et al., 2024 | | | Mapping the intellectual structure and landscape of colorectal cancer immunotherapy: A bibliometric analysis | No | Irrelevant |
| 1086 | | Bokemeyer et al., 2024 | | | Cetuximab Every 2 Weeks Versus Standard Weekly Dosing Administration Schedule | No | Irrelevant |
| 1087 | | Bai et al., 2024 | | | Feasibility and Tolerability of Anlotinib Plus PD-1 Blockades for Patients with Treatment-Refractory Metastatic Colorectal Cancer | No | - |
| 1088 | | Wang et al., 2024 | | | Comparative efficacy and safety of anti-PD-1 therapies used in metastatic colorectal cancer: a systematic review and network meta-analysis | No | - |
| 1089 | | Nemoto et al., 2024 | | | Case report: Pathological complete response of pregnancy associated pulmonary enteric adenocarcinoma to chemoradiotherapy | No | Irrelevant |
| 1090 | | Rakké et al., 2024 | | | Engaging stimulatory immune checkpoint interactions in the tumour immune microenvironment of primary liver cancers | No | Irrelevant |
| 1091 | | Mohr et al., 2024 | | | Advances and challenges in immunoPET methodology | No | Irrelevant |
| 1092 | | Jung et al., 2024 | | | Protocol of a first-in-human clinical trial to evaluate the safety, tolerability, and preliminary efficacy of the bispecific CD276xCD3 antibody CC-3 in patients with colorectal cancer (CoRe_CC-3) | No | Irrelevant |
| 1093 | | Grant et al., 2024 | | | A remnant never forgotten: the utility of circulating tumor DNA in treatment guidance of urachal cancer | No | Irrelevant |
| 1094 | | Fang et al., 2024 | | | Targeting the CD24-Siglec10 Axis: A Potential Strategy for Cancer Immunotherapy | No | Irrelevant |
| 1095 | | Hu et al., 2024 | | | Roles and inhibitors of FAK in cancer: current advances and future directions | No | Irrelevant |
| 1096 | | Yin et al., 2024 | | | Overexpression of FERM Domain Containing Kindlin 2 (FERMT2) in Fibroblasts Correlates with EMT and Immunosuppression in Gastric Cancer | No | Irrelevant |
| 1097 | | Sahu et al., 2024 | | | Targeting KRAS and SHP2 signaling pathways for immunomodulation and improving treatment outcomes in solid tumors | No | Irrelevant |
| 1098 | | Allam et al., 2024 | | | Role of CTLA4 and pSTAT3 Immunostaining in Prognosis and Treatment of the Colorectal Carcinoma | No | Irrelevant |
| 1099 | | Gao et al., 2024 | | | Translational and oncologic significance of tertiary lymphoid structures in pancreatic adenocarcinoma | No | Irrelevant |
| 1100 | | San-Román-Gil et al., 2024 | | | Case report: Efficacy of immunotherapy as conversion therapy in dMMR/MSI-H colorectal cancer: a case series and review of the literature | No | Irrelevant |
| 1101 | | Li et al., 2024 | | | The roles of epigallocatechin gallate in the tumor microenvironment, metabolic reprogramming, and immunotherapy | No | Irrelevant |
| 1102 | | Rezagholizadeh et al., 2024 | | | Unraveling the potential of CD8, CD68, and VISTA as diagnostic and prognostic markers in patients with pancreatic ductal adenocarcinoma | No | Irrelevant |
| 1103 | | Ahmad et al., 2024 | | | Emerging trends in gastrointestinal cancers: Targeting developmental pathways in carcinogenesis and tumor progression | No | Irrelevant |
| 1104 | | Karaoğlan et al., 2024 | | | Exploring the connection between microsatellite instability and inflammatory indicators in cancers | No | Irrelevant |
| 1105 | | Attias et al., 2024 | | | The impact of Foxp3+ regulatory T-cells on CD8+ T-cell dysfunction in tumour microenvironments and responses to immune checkpoint inhibitors | No | Irrelevant |
| 1106 | | Yang et al., 2024 | | | Multiple Factors Determine the Oncolytic or Carcinogenic Effects of TLRs Activation in Cancer | No | Irrelevant |
| 1107 | | Zheng & Wang, 2024 | | | Techniques and status of hepatic arterial infusion chemotherapy for primary hepatobiliary cancers | No | Irrelevant |
| 1108 | | Mignini et al., 2024 | | | From the Colon to the Liver: How Gut Microbiota May Influence Colorectal Cancer Metastatic Potential | No | Irrelevant |
| 1109 | | Saoudi González et al., 2024 | | | Cetuximab as a Key Partner in Personalized Targeted Therapy for Metastatic Colorectal Cancer | No | Irrelevant |
| 1110 | | Franzén et al., 2024 | | | Next-Generation CEA-CAR-NK-92 Cells against Solid Tumors: Overcoming Tumor Microenvironment Challenges in Colorectal Cancer | No | Irrelevant |
| 1111 | | Cretu et al., 2024 | | | Role of Cannabinoids in Oral Cancer | No | Irrelevant |
| 1112 | | Volovat et al., 2024 | | | Oncolytic Virotherapy: A New Paradigm in Cancer Immunotherapy | No | Irrelevant |
| 1113 | | Stanilov et al., 2024 | | | Navigating the Cytokine Seas: Targeting Cytokine Signaling Pathways in Cancer Therapy | No | Irrelevant |
| 1114 | | Vuletić et al., 2024 | | | Role of Histone Deacetylase 6 and Histone Deacetylase 6 Inhibition in Colorectal Cancer | No | Irrelevant |
| 1115 | | Koumprentziotis et al., 2024 | | | New Emerging Targets in Cancer Immunotherapy: The Role of B7-H3 | No | Irrelevant |
| 1116 | | Ciernikova et al., 2024 | | | Microbiome in Cancer Development and Treatment | No | Irrelevant |
| 1117 | | Boussaa et al., 2024 | | | The first case of SARS-CoV-2-induced eosinophilic fasciitis | No | Not relevant |
| 1118 | | Zhang et al., 2024 | | | Global burden, risk factors, clinicopathological characteristics, molecular biomarkers, and outcomes of microsatellite instability-high gastric cancer | No | Irrelevant |
| 1119 | | Saman et al., 2024 | | | A Comprehensive Review on Current Treatments and Challenges Involved in the Treatment of Ovarian Cancer | No | Irrelevant |
| 1120 | | Yang et al., 2024 | | | The critical role of tumor microbiome in cancer immunotherapy | No | Irrelevant |
| 1121 | | Liu et al., 2024 | | | Tumor resistance to anti-mesothelin CAR-T cells caused by binding to shed mesothelin is overcome by targeting a juxtamembrane epitope | No | Irrelevant |
| 1122 | | Jung et al., 2024 | | | First-in-Human Phase 1 Study of a B Cell– and Monocyte-Based Immunotherapeutic Vaccine against HER2-Positive Advanced Gastric Cancer | No | Irrelevant |
| 1123 | | Swierczynski et al., 2024 | | | Regulators of G-Protein Signaling (RGS) in Sporadic and Colitis-Associated Colorectal Cancer | No | Irrelevant |
| 1124 | | Chen et al., 2024 | | | Multilevel Heterogeneity of Colorectal Cancer Liver Metastasis | No | Irrelevant |
| 1125 | | Nenkov et al., 2024 | | | Targeting Farnesoid X Receptor in Tumor and the Tumor Microenvironment: Implication for Therapy | No | Irrelevant |
| 1126 | | Das et al., 2024 | | | Biomarkers in Cancer Detection, Diagnosis, and Prognosis | No | Irrelevant |
| 1127 | | Faghfuri et al., 2024 | | | Recent advances in personalized cancer immunotherapy with immune checkpoint inhibitors, T cells and vaccines | No | Irrelevant |
| 1128 | | Yang et al., 2024 | | | Artificial Intelligence Applications in the Treatment of Colorectal Cancer: A Narrative Review | No | Irrelevant |
| 1129 | | Nagar et al., 2024 | | | Protein-Based Nanocarriers and Nanotherapeutics for Infection and Inflammation | No | Irrelevant |
| 1130 | | Workenhe et al., 2024 | | | Determinants for Antitumor and Protumor Effects of Programmed Cell Death | No | Irrelevant |
| 1131 | | Li et al., 2024 | | | Clinical application of cytokine-induced killer (CIK) cell therapy in colorectal cancer: Current strategies and future challenges | No | Irrelevant |
| 1132 | | Meng et al., 2024 | | | Mechanisms of immune checkpoint inhibitors: Insights into the regulation of circular RNAs involved in cancer hallmarks | No | Irrelevant |
| 1133 | | Vu et al., 2024 | | | Decoupling FcRn and tumor contributions to elevated immune checkpoint inhibitor clearance in cancer cachexia | No | Irrelevant |
| 1134 | | Liang et al., 2024 | | | Leveraging diverse cell-death patterns to predict the clinical outcome of immune checkpoint therapy in lung adenocarcinoma | No | Irrelevant |
| 1135 | | Cho et al., 2024 | | | Association Between Clinicopathological Parameters and S100A8/A9 Expression in NSCLC Patients | No | Irrelevant |
| 1136 | | Marcu et al., 2024 | | | Circadian rhythm-based cancer therapy in randomized clinical trials | No | Irrelevant |
| 1137 | | Caughey et al., 2024 | | | Targeting KRAS-Mutated Gastrointestinal Malignancies with Small-Molecule Inhibitors | No | Irrelevant |
| 1138 | | Dagher et al., 2024 | | | Moving into the modern era of molecular classification for endometrial cancer | No | Irrelevant |
| 1139 | | Kong et al., 2024 | | | The FJQR Has Synergistic Effect with Fluoropyrimidine in Maintenance Treatment for HER-2 Negative Gastric Cancer | No | Irrelevant |
| 1140 | | Mempel et al., 2024 | | | How chemokines organize the tumor microenvironment | No | Irrelevant |
| 1141 | | Ito et al., 2024 | | | Characterization of colorectal cancer by hierarchical clustering analyses of five immune cell markers | No | Irrelevant |
| 1142 | | Yang et al., 2024 | | | Personalizing adjuvant therapy for patients with colorectal cancer | No | Irrelevant |
| 1143 | | Ma et al., 2024 | | | Bcl-xL mediates interferon-beta secretion by PAR2 deficiency in CRC metastasis | No | Irrelevant |
| 1144 | | Pasquale et al., 2024 | | | Eph receptors and ephrins in cancer progression | No | Irrelevant |
| 1145 | | Saberzadeh-Ardestani et al., 2024 | | | Metastatic site and clinical outcome of dMMR metastatic CRC treated with an immune checkpoint inhibitor | No | - |
| 1146 | | Anami et al., 2024 | | | Dysfunction of sinus macrophages in tumor-bearing host induces resistance to immunotherapy | No | Irrelevant |
| 1147 | | Wang et al., 2024 | | | KIRREL promotes the proliferation of gastric cancer cells via PI3K/AKT/mTOR pathway | No | Irrelevant |
| 1148 | | Xie et al., 2024 | | | Mitochondrial-related features and immunotherapy prognosis in CRC | No | Irrelevant |
| 1149 | | Arroyo-Olarte et al., 2024 | | | Expanded Alternatives of CRISPR–Cas9 Applications in Immunotherapy of CRC | No | Irrelevant |
| 1150 | | Sharma et al., 2024 | | | Multifunctional nanocomposites modulating the tumor microenvironment for enhanced cancer immunotherapy | No | Irrelevant |
| 1151 | | Galassi et al., 2024 | | | Molecular determinants of immunogenic cell death elicited by radiation therapy | No | Irrelevant |
| 1152 | | Chakraborty et al., 2024 | | | Multiple Protein Biomarkers and Different Treatment Strategies for CRC | No | Irrelevant |
| 1153 | | Soler-González et al., 2024 | | | Update on the management of elderly patients with CRC | No | Irrelevant |
| 1154 | | Andhari et al., 2024 | | | Advancements in tumor microenvironment landscaping for immune checkpoint therapies | No | Irrelevant |
| 1155 | | Shu et al., 2024 | | | The current status and prospect of immunotherapy in colorectal cancer | No | Irrelevant |
| 1156 | | Banerjee et al., 2024 | | | Fragment-based investigation of thiourea derivatives as VEGFR-2 inhibitors | No | Irrelevant |
| 1157 | | Singhal et al., 2024 | | | Role of gut microbiota in tumorigenesis and antitumoral therapies | No | Irrelevant |
| 1158 | | Kutuk et al., 2023 | | | Tumor treating fields: narrative review of a promising treatment modality for cancer | No | Irrelevant |
| 1159 | | Xiao et al., 2023 | | | Current methods for the detection of glypican-3 | No | Irrelevant |
| 1160 | | Patil et al., 2023 | | | Nanotherapeutics for colorectal cancer using metal nanocomposites | No | Irrelevant |
| 1161 | | Lin et al., 2023 | | | Influence and research progress in human microbial flora on the efficacy of anti-tumor drugs | No | Irrelevant |
| 1162 | | Kondapuram et al., 2023 | | | Targeting survivin for cancer therapy: Strategies, small molecule inhibitors, and vaccine-based therapeutics | No | Irrelevant |
| 1163 | | Ru et al., 2023 | | | Technologies of targeting histone deacetylase in drug discovery: Current progress and emerging prospects | No | Irrelevant |
| 1164 | | Su et al., 2023 | | | ETV4 facilitates angiogenesis in hepatocellular carcinoma by upregulating MMP14 expression | No | Irrelevant |
| 1165 | | He et al., 2023 | | | Patient-derived tumor models and their distinctive applications in personalized drug therapy | No | Irrelevant |
| 1166 | | Wang et al., 2023 | | | Defining the role of metastasis-initiating cells in promoting carcinogenesis in ovarian cancer | No | Irrelevant |
| 1167 | | Lin et al., 2023 | | | Recent Advances in Drugs Targeting RAS Oncoprotein and Clinical Applications | No | Irrelevant |
| 1168 | | Zeng et al., 2023 | | | Expression, prognostic value, and potential immunotherapeutic target of COL1A1 in colon cancer | No | Irrelevant |
| 1169 | | Qian et al., 2023 | | | METTL3 promotes NSCLC growth and metastasis by inhibiting FDX1 through copper death-associated pri-miR-21-5p maturation | No | Irrelevant |
| 1170 | | Zhang et al., 2023 | | | An update on gemcitabine-based chemosensitization strategies in pancreatic ductal adenocarcinoma | No | Irrelevant |
| 1171 | | Guo et al., 2023 | | | Advances in immunotherapy of early-middle stage CRC with MSI-H and stability | No | - |
| 1172 | | Lee et al., 2023 | | | Biomarkers for colorectal cancer chemotherapy: Recent updates and future perspectives | No | Irrelevant |
| 1173 | | Ruivo et al., 2023 | | | Colorectal cancer liver metastasis—state-of-the-art and future perspectives | No | Irrelevant |
| 1174 | | Korneenko et al., 2023 | | | At the crossroads of the cGAS-cGAMP-STING pathway and the DNA damage response | No | Irrelevant |
| 1175 | | Al Khatib et al., 2023 | | | Inhaled medicines for targeting non-small cell lung cancer | No | Irrelevant |
| 1176 | | Behrens et al., 2023 | | | Establishment and characterization of xenograft (PDX) models for pancreatic cancer | No | Irrelevant |
| 1177 | | Buchholz et al., 2023 | | | The role of ERBB2 alterations as treatment options for CRC patients in precision oncology | No | Irrelevant |
| 1178 | | Baghy et al., 2023 | | | Insights into the tumor microenvironment—components, functions, and therapeutics | No | Irrelevant |
| 1179 | | Thenuwara et al., 2023 | | | Advances in diagnostic tools and therapeutic approaches for gliomas | No | Irrelevant |
| 1180 | | Mosca et al., 2023 | | | Neutrophil-to-lymphocyte ratio (NLR) in NSCLC, gastrointestinal, and other solid tumors | No | Irrelevant |
| 1181 | | Wang et al., 2023 | | | Targeting CRAF kinase in anti-cancer therapy: Progress and opportunities | No | Irrelevant |
| 1182 | | van de Weijer et al., 2023 | | | A novel patient-derived meningioma spheroid model to study EMT in meningiomas | No | Irrelevant |
| 1183 | | Seckinger et al., 2023 | | | Development of NILK-2301, a bispecific antibody for CEACAM5-expressing cancers | No | Irrelevant |
| 1184 | | Agrafiotis et al., 2023 | | | The use of anti-angiogenic agents in the treatment of thymic epithelial tumors | No | Irrelevant |
| 1185 | | Sun et al., 2023 | | | Single-cell RNA sequencing in cancer research for novel biomarkers | No | Irrelevant |
| 1186 | | Xia et al., 2023 | | | Peritoneal metastasis: A dilemma and challenge in metastatic CRC treatment | No | - |
| 1187 | | Zhang et al., 2023 | | | Small antibodies with big applications: Nanobody-based cancer diagnostics | No | Irrelevant |
| 1188 | | Cao et al., 2023 | | | Oncofetal reprogramming in tumor development and cancer therapy | No | Irrelevant |
| 1189 | | Qian et al., 2023 | | | Prognostic evaluation of stage I lung adenocarcinoma based on systemic inflammatory response | No | Irrelevant |
| 1190 | | Dai et al., 2023 | | | Novel combination approaches for liver tumor therapy | No | Irrelevant |
| 1191 | | Freitas et al., 2023 | | | Gene expression alterations predict pathological complete response in TNBC | No | Irrelevant |
| 1192 | | He et al., 2023 | | | Mechanisms by which intestinal microbiota affects gastrointestinal tumors | No | Irrelevant |
| 1193 | | Lu et al., 2023 | | | The effects of ARID1A mutation in gastric cancer and its significance for treatment | No | Irrelevant |
| 1194 | | Heczko et al., 2023 | | | Whole exome sequencing for managing metachronous CRC liver metastases | No | Irrelevant |
| 1195 | | Yi et al., 2023 | | | Exploiting innate immunity for cancer immunotherapy | No | Irrelevant |
| 1196 | | Chai et al., 2023 | | | Paradoxical effect of anticancer drugs | No | Irrelevant |
| 1197 | | Weber et al., 2023 | | | Rare germline variants in POLE and POLD1 in glioma families | No | Irrelevant |
| 1198 | | Shen et al., 2023 | | | Improving oncolytic virus therapy by targeting macrophages | No | Irrelevant |
| 1199 | | Boublikova et al., 2023 | | | Total neoadjuvant therapy in rectal cancer: Evidence and expectations | No | Irrelevant |
| 1200 | | Yang et al., 2023 | | | Translational research in targeted therapy for colorectal cancer | No | Irrelevant |
| 1201 | | Dong et al., 2023 | | | High expression of CDKN2A is associated with poor prognosis in CRC and may guide PD-1-mediated immunotherapy | No | Irrelevant |
| 1202 | | Neupane et al., 2023 | | | Opportunities and challenges for a histology-agnostic utilization of trastuzumab deruxtecan | No | Irrelevant |
| 1203 | | Taieb et al., 2023 | | | Treatment of gastric adenocarcinoma: A rapidly evolving landscape | No | Irrelevant |
| 1204 | | Shekari et al., 2023 | | | VISTA and its ligands: The next generation of promising therapeutic targets in immunotherapy | No | Irrelevant |
| 1205 | | Hu et al., 2023 | | | Tumor-associated neutrophils upregulate PANoptosis in NSCLC | No | Irrelevant |
| 1206 | | Yang et al., 2023 | | | Tumor immune microenvironment and current immunotherapy of cholangiocarcinoma | No | Irrelevant |
| 1207 | | Fan et al., 2023 | | | Alleviating hypoxia to improve cancer immunotherapy | No | Irrelevant |
| 1208 | | Lo et al., 2023 | | | ICI-induced colitis is mediated by polyfunctional lymphocytes via IL23/IFNγ axis | No | Irrelevant |
| 1209 | | Hong et al., 2023 | | | Gastrointestinal signet ring cell malignancy: Current advancement and future prospects | No | Irrelevant |
| 1210 | | Dewdney et al., 2023 | | | Glioblastoma targeted therapies: Two decades of progress | No | Irrelevant |
| 1211 | | Lin et al., 2023 | | | PD-1 and PD-L1 inhibitors in cold colorectal cancer: Challenges and strategies | No | Irrelevant |
| 1212 | | Ren et al., 2023 | | | CBX4 promotes antitumor immunity by suppressing Pdcd1 expression in T cells | No | Irrelevant |
| 1213 | | Shreenivas et al., 2023 | | | ALK fusions in the pan-cancer setting: Another tumor-agnostic target? | No | Irrelevant |
| 1214 | | Svrcek et al., 2023 | | | Histopronostic factors and biomarkers for personalized CRC management | No | Irrelevant |
| 1215 | | Wang et al., 2023 | | | DDR gene mutations, TMB, and PD-L1 in solid tumor genomes | No | Irrelevant |
| 1216 | | Zhang et al., 2023 | | | Role of immune-related non-coding RNAs in tumor immunoresponse via MICA/NKG2D pathway | No | Irrelevant |
| 1217 | | Zhao et al., 2023 | | | Drug-microbiota interactions: An emerging priority for precision medicine | No | Irrelevant |
| 1218 | | Liu et al., 2023 | | | Why Treg should be the focus of cancer immunotherapy | No | Irrelevant |
| 1219 | | Sanchon-Sanchez et al., 2023 | | | Enhancing cholangiocarcinoma sensitivity to anticancer drugs | No | Irrelevant |
| 1220 | | Fidelle et al., 2023 | | | Impact of intestinal microbiota in colorectal cancer | No | Irrelevant |
| 1221 | | Shaw et al., 2023 | | | DDX5 regulates MGMT gene expression in colon cancer cells | No | Irrelevant |
| 1222 | | Idoudi et al., 2023 | | | Role of HMGB1 and its associated signaling pathways in human malignancies | No | Irrelevant |
| 1223 | | Binabaj et al., 2023 | | | Vactosertib improves anti-tumor properties of 5-FU for CRC | No | Irrelevant |
| 1224 | | Li et al., 2023 | | | Fusobacterium nucleatum infection attenuates antitumor immunity in ESCC | No | Irrelevant |
| 1225 | | Ruff et al., 2023 | | | Review of targeted therapy and immune checkpoint inhibitors for metastatic CRC | No | Irrelevant |
| 1226 | | Doleschal et al., 2023 | | | Real-world evidence on molecular matched targeted treatment in biliary tract cancer | No | Irrelevant |
| 1227 | | Mogenet et al., 2023 | | | Immunologic constant of rejection as a predictive biomarker for ICI in NSCLC | No | Irrelevant |
| 1228 | | Chen et al., 2023 | | | B7-H3 and CD47 co-expression in gastric cancer as predictors of prognosis | No | Irrelevant |
| 1229 | | He et al., 2023 | | | Metastasis organotropism in CRC: Advancing toward innovative therapies | No | Irrelevant |
| 1230 | | Li et al., 2023 | | | Innovations in DNA damage response | No | Irrelevant |
| 1231 | | Yang et al., 2023 | | | Role of the cGAS-STING pathway in radiotherapy for NSCLC | No | Irrelevant |
| 1232 | | Li et al., 2023 | | | Tumor-specific neoantigens for TCR-T cell therapy | No | Irrelevant |
| 1233 | | Zhao et al., 2023 | | | Epigenetic upregulation of TROP2 and SLFN11 enhances efficacy of sacitizumab govitecan | No | Irrelevant |
| 1234 | | Guo et al., 2023 | | | Tumor microenvironment characterization in CRC to identify prognostic genes | No | Irrelevant |
| 1235 | | Li et al., 2023 | | | Glycolysis-related risk score for predicting prognosis and ICI responsiveness in CRC | No | Irrelevant |
| 1236 | | Kerdivel et al., 2023 | | | DNMT1 and DNMT3A-driven DNA hypermethylation contributes to tumor immune escape | No | Irrelevant |
| 1237 | | Jahandideh et al., 2023 | | | Macrophage’s role in solid tumors: Two edges of a sword | No | Irrelevant |
| 1238 | | Muhammad et al., 2023 | | | IL-2 and IL-2R targeting strategies in cancer treatment | No | Irrelevant |
| 1239 | | Baranov et al., 2023 | | | Pathologic evaluation of therapeutic biomarkers in CRC | No | Irrelevant |
| 1240 | | Haake et al., 2023 | | | Tumor-derived GDF-15 blocks LFA-1 dependent T cell recruitment in anti-PD-1 treatment | No | Irrelevant |
| 1241 | | Wang et al., 2023 | | | Liver metastasis from CRC: Pathogenesis, immune landscape, and therapeutic approaches | No | Irrelevant |
| 1242 | | Fang et al., 2023 | | | Sintilimab plus bevacizumab and CapeOx in RAS mutant, MSS, metastatic CRC | No | Irrelevant |
| 1243 | | Ding et al., 2023 | | | Overcoming melanoma resistance to ICI using nano-strategies | No | Irrelevant |
| 1244 | | Liu et al., 2023 | | | Advances in PD-1 signaling inhibition-based nano-delivery systems for tumors | No | Irrelevant |
| 1245 | | Li et al., 2023 | | | Surgical outcomes of preserving or peeling IMA sheath in rectal cancer | No | Irrelevant |
| 1246 | | Zhong et al., 2023 | | | ICI therapy for BRAF mutant metastatic CRC: Efficacy, new strategies, and biomarkers | No | Irrelevant |
| 1247 | | Zhang et al., 2023 | | | Immune risk scoring system related to CD8+ T cells in uterine corpus endometrial carcinoma | No | Irrelevant |
| 1248 | | Fang et al., 2023 | | | m6A methylation reader IGF2BP2 promotes angiogenesis and metastasis in lung adenocarcinoma | No | Irrelevant |
| 1249 | | Wang et al., 2023 | | | M1A and m7G modifications as biomarkers in esophageal SCC | No | Irrelevant |
| 1250 | | Tang et al., 2023 | | | CD73-driven immunosuppression in pancreatic cancer | No | Irrelevant |
| 1251 | | Makita et al., 2023 | | | Factors affecting local control of bone metastases from radioresistant tumors treated with palliative external beam radiotherapy | No | Irrelevant |
| 1252 | | Du et al., 2023 | | | The role of mitochondria in the resistance of melanoma to PD-1 inhibitors | No | Irrelevant |
| 1253 | | Zhao et al., 2023 | | | Role of the gut microbiota in anticancer therapy: from molecular mechanisms to clinical applications | No | Irrelevant |
| 1254 | | Liu et al., 2023 | | | Angiogenic signaling pathways and anti-angiogenic therapy for cancer | No | Irrelevant |
| 1255 | | Li et al., 2023 | | | The Notch signaling pathway: a potential target for cancer immunotherapy | No | Irrelevant |
| 1256 | | Zhang et al., 2023 | | | OncoVee™-MiniPDX-guided anticancer treatment for HER2-negative intermediate-advanced gastric cancer patients: a single-arm, open-label phase I clinical study | No | Irrelevant |
| 1257 | | Fang et al., 2023 | | | China special issue on gastrointestinal tumors-Regulatory-immunoscore—A novel indicator to guide precision adjuvant chemotherapy in colorectal cancer | No | Irrelevant |
| 1258 | | Yang et al., 2023 | | | Identification of mitochondrial respiratory chain signature for predicting prognosis and immunotherapy response in stomach adenocarcinoma | No | Irrelevant |
| 1259 | | Najjary et al., 2023 | | | Tumor lineage-specific immune response in brain metastatic disease: opportunities for targeted immunotherapy regimen? | No | Irrelevant |
| 1260 | | Zhou et al., 2023 | | | Super-enhancer-driven TOX2 mediates oncogenesis in Natural Killer/T Cell Lymphoma | No | Irrelevant |
| 1261 | | Grünewald et al., 2023 | | | Effects of regorafenib on the mononuclear/phagocyte system and how these contribute to the inhibition of colorectal tumors in mice | No | Irrelevant |
| 1262 | | El Hajj et al., 2023 | | | Immune Checkpoint Inhibitors in pMMR/MSS Colorectal Cancer | No | Irrelevant |
| 1263 | | Taib et al., 2023 | | | Treatment with decitabine induces the expression of stemness markers, PD-L1 and NY-ESO-1 in colorectal cancer: potential for combined chemoimmunotherapy | No | Irrelevant |
| 1264 | | Granata et al., 2023 | | | Colorectal liver metastases patients prognostic assessment: prospects and limits of radiomics and radiogenomics | No | Irrelevant |
| 1265 | | Falahat et al., 2023 | | | Epigenetic state determines the in vivo efficacy of STING agonist therapy | No | Irrelevant |
| 1266 | | Ziranu et al., 2023 | | | CDX-2 expression correlates with clinical outcomes in MSI-H metastatic colorectal cancer patients receiving immune checkpoint inhibitors | No | - |
| 1267 | | Zhang et al., 2023 | | | Research progress on non-protein-targeted drugs for cancer therapy | No | Irrelevant |
| 1268 | | Rolfo et al., 2023 | | | Applications and clinical trial landscape using Toll-like receptor agonists to reduce the toll of cancer | No | Irrelevant |
| 1269 | | Liu et al., 2023 | | | Galectins and galectin-mediated autophagy regulation: new insights into targeted cancer therapy | No | Irrelevant |
| 1270 | | Wang et al., 2023 | | | Targeting epigenetic regulators to overcome drug resistance in cancers | No | Irrelevant |
| 1271 | | Wang et al., 2023 | | | m6A-related lncRNA-based immune infiltration characteristic analysis and prognostic model for colonic adenocarcinoma | No | Irrelevant |
| 1272 | | Conilh et al., 2023 | | | Payload diversification: a key step in the development of antibody–drug conjugates | No | Irrelevant |
| 1273 | | Huang et al., 2023 | | | Prognostic costimulatory molecule-related signature risk model correlates with immunotherapy response in colon cancer | No | Irrelevant |
| 1274 | | Amonkar et al., 2023 | | | Clinical outcomes of chemotherapy-based therapies for previously treated advanced colorectal cancer: a systematic literature review and meta-analysis | No | Irrelevant |
| 1275 | | Zhang et al., 2023 | | | HRS mediates tumor immune evasion by regulating proteostasis-associated interferon pathway activation | No | Irrelevant |
| 1276 | | Wang et al., 2023 | | | Targeting VCP potentiates immune checkpoint therapy for colorectal cancer | No | Irrelevant |
| 1277 | | Allan et al., 2023 | | | The prognostic impact of peritoneal tumour DNA in gastrointestinal and gynaecological malignancies: a systematic review | No | Irrelevant |
| 1278 | | Wang et al., 2023 | | | Genetic insights into thymic carcinomas and thymic neuroendocrine neoplasms denote prognosis signatures and pathways | No | Irrelevant |
| 1279 | | Gandhi et al., 2023 | | | Systemic infusion of TLR3-ligand and IFN-α in patients with breast cancer reprograms local tumor microenvironments for selective CTL influx | No | Irrelevant |
| 1280 | | Meyblum et al., 2023 | | | Local and distant response to intratumoral immunotherapy assessed by immunoPET in mice | No | Irrelevant |
| 1281 | | Pretta et al., 2023 | | | Mismatch Repair system protein deficiency as a resistance factor for locally advanced rectal adenocarcinoma patients receiving neoadjuvant chemo-radiotherapy | No | Irrelevant |
| 1282 | | Jarak et al., 2023 | | | Colorectal cancer cell exosome and cytoplasmic membrane for homotypic delivery of therapeutic molecules | No | Irrelevant |
| 1283 | | Melissourgou-Syka et al., 2023 | | | A Review of Scheduling Strategies for Radiotherapy and Immune Checkpoint Inhibition in Locally Advanced Rectal Cancer | No | Irrelevant |
| 1284 | | Niazi et al., 2023 | | | A Critical Analysis of the FDA’s Omics-Driven Pharmacodynamic Biomarkers to Establish Biosimilarity | No | Irrelevant |
| 1285 | | Miliotou et al., 2023 | | | Recruiting In Vitro Transcribed mRNA against Cancer Immunotherapy: A Contemporary Appraisal of the Current Landscape | No | Irrelevant |
| 1286 | | Ballarò et al., 2023 | | | Colorectal Liver Metastasis: Can Cytokines Make the Difference? | No | Irrelevant |
| 1287 | | Abdelrahim et al., 2023 | | | Transplant Oncology: An Emerging Discipline of Cancer Treatment | No | Irrelevant |
| 1288 | | Liu et al., 2023 | | | The Predictive Value of CD3+/CD8+ Lymphocyte Infiltration and PD-L1 Expression in Colorectal Cancer | No | Irrelevant |
| 1289 | | Liu et al., 2023 | | | Metabolic reprogramming in nasopharyngeal carcinoma: Mechanisms and therapeutic opportunities | No | Irrelevant |
| 1290 | | Kumarasamy et al., 2023 | | | Glycoproteomics-based liquid biopsy: translational outlook for colorectal cancer clinical management in Southeast Asia | No | Irrelevant |
| 1291 | | Domentean et al., 2023 | | | Role of UBE2C in Brain Cancer Invasion and Dissemination | No | Irrelevant |
| 1292 | | Park et al., 2023 | | | Therapeutic Strategies for Pancreatic-Cancer-Related Type 2 Diabetes Centered around Natural Products | No | Irrelevant |
| 1293 | | Sartorius et al., 2023 | | | Implications of Rectal Cancer Radiotherapy on the Immune Microenvironment: Allies and Foes to Therapy Resistance and Patients’ Outcome | No | Irrelevant |
| 1294 | | Matteucci et al., 2023 | | | Immunocheckpoint Inhibitors in Microsatellite-Stable or Proficient Mismatch Repair Metastatic Colorectal Cancer: Are We Entering a New Era? | No | Irrelevant |
| 1295 | | Zlotnik et al., 2023 | | | Targeting Liver Metastases to Potentiate Immunotherapy in MS-Stable Colorectal Cancer—A Review of the Literature | No | Irrelevant |
| 1296 | | Karamitopoulou et al., 2023 | | | Emerging Prognostic and Predictive Factors in Pancreatic Cancer | No | Irrelevant |
| 1297 | | Cao et al., 2023 | | | MSI-XGNN: an explainable GNN computational framework integrating transcription- and methylation-level biomarkers for microsatellite instability detection | No | Irrelevant |
| 1298 | | Zhang et al., 2023 | | | Targeting Epidermal Growth Factor Receptor for Cancer Treatment: Abolishing Both Kinase-Dependent and Kinase-Independent Functions of the Receptor | No | Irrelevant |
| 1299 | | Singh et al., 2023 | | | TGF-β in correlation with tumor progression, immunosuppression and targeted therapy in colorectal cancer | No | Irrelevant |
| 1300 | | Wang et al., 2023 | | | Metabolic reprogramming, autophagy, and ferroptosis: Novel arsenals to overcome immunotherapy resistance in gastrointestinal cancer | No | Irrelevant |
| 1301 | | Terashima et al., 2023 | | | Predictive Impact of Diffuse Positivity for TTF-1 Expression in Patients Treated With Platinum-Doublet Chemotherapy Plus Immune Checkpoint Inhibitors for Advanced Nonsquamous NSCLC | No | Irrelevant |
| 1302 | | Brooksbank et al., 2023 | | | DNA mismatch repair deficient cancer – Emerging biomarkers of resistance to immune checkpoint inhibition | No | Irrelevant |
| 1303 | | Chen et al., 2023 | | | Tailored to a Woman’s Heart: Gender Cardio-Oncology Across the Lifespan | No | Irrelevant |
| 1304 | | Høland et al., 2023 | | | Transcriptomic subtyping of malignant peripheral nerve sheath tumours highlights immune signatures, genomic profiles, patient survival and therapeutic targets | No | Irrelevant |
| 1305 | | Zhao et al., 2023 | | | Breaking the mold: Overcoming resistance to immune checkpoint inhibitors | No | Irrelevant |
| 1306 | | Jing et al., 2023 | | | Combating drug resistance in hepatocellular carcinoma: No awareness today, no action tomorrow | No | Irrelevant |
| 1307 | | Guil-Luna et al., 2023 | | | Clinical significance of glycogen synthase kinase 3 (GSK-3) expression and tumor budding grade in colorectal cancer: Implications for targeted therapy | No | Irrelevant |
| 1308 | | Li et al., 2023 | | | Monophosphoryl lipid A-assembled nanovaccines enhance tumor immunotherapy | No | Irrelevant |
| 1309 | | Garg et al., 2023 | | | Unraveling the intricate relationship: Influence of microbiome on the host immune system in carcinogenesis | No | Irrelevant |
| 1310 | | Granata et al., 2023 | | | Radiomics and machine learning analysis by computed tomography and magnetic resonance imaging in colorectal liver metastases prognostic assessment | No | Irrelevant |
| 1311 | | Oshima et al., 2023 | | | Immune checkpoint inhibitor therapy in neoadjuvant and adjuvant treatment for cancer: A paradigm shift in the treatment of resectable gastrointestinal cancer | No | Irrelevant |
| 1312 | | Zhao et al., 2023 | | | Personalized Cancer Monitoring Assay for the Detection of ctDNA in Patients with Solid Tumors | No | Irrelevant |
| 1313 | | Zeng et al., 2023 | | | Clinical research progress on BRAF V600E-mutant advanced colorectal cancer | No | Irrelevant |
| 1314 | | Borgeaud et al., 2023 | | | Novel targets for immune-checkpoint inhibition in cancer | No | Irrelevant |
| 1315 | | Yu et al., 2023 | | | Identification of ferroptosis-related molecular subtypes and a methylation-related ferroptosis gene prognostic signature in cervical squamous cell carcinoma | No | Irrelevant |
| 1316 | | Benešová et al., 2023 | | | Microbiota as the unifying factor behind the hallmarks of cancer | No | Irrelevant |
| 1317 | | Sugimura et al., 2023 | | | Reovirus combined with a STING agonist enhances anti-tumor immunity in a mouse model of colorectal cancer | No | Irrelevant |
| 1318 | | Maloney et al., 2023 | | | The role of diagnostic, prognostic, and predictive biomarkers in the management of early pancreatic cancer | No | Irrelevant |
| 1319 | | Andel et al., 2023 | | | Colorectal liver metastases that survive radioembolization display features of aggressive tumor behavior | No | Irrelevant |
| 1320 | | Fornaro et al., 2023 | | | Concordance of microsatellite instability and mismatch repair status in paired biopsies and surgical specimens of resectable gastroesophageal adenocarcinoma: time for a call to action | No | Irrelevant |
| 1321 | | Li et al., 2023 | | | Bispecific antibody targeting both B7-H3 and PD-L1 exhibits superior antitumor activities | No | Irrelevant |
| 1322 | | Wu et al., 2023 | | | The tumor microenvironment in the postsurgical liver: Mechanisms and potential targets of postoperative recurrence in human hepatocellular carcinoma | No | Irrelevant |
| 1323 | | Sahoo et al., 2023 | | | Prospective Challenges for Patenting and Clinical Trials of Anticancer Compounds from Natural Products: Coherent Review | No | Irrelevant |
| 1324 | | Zheng-Lin et al., 2023 | | | Targeting ERBB2/HER2 genetic alterations: an expanding therapeutic opportunity in gastrointestinal cancers | No | Irrelevant |
| 1325 | | Thomas et al., 2023 | | | Advancing translational research for colorectal immuno-oncology | No | Irrelevant |
| 1326 | | Babl et al., 2023 | | | MCT4 blockade increases the efficacy of immune checkpoint blockade | No | Irrelevant |
| 1327 | | Adegoke et al., 2023 | | | Classification of the tumor immune microenvironment and associations with outcomes in patients with metastatic melanoma treated with immunotherapies | No | Irrelevant |
| 1328 | | Akce et al., 2023 | | | Phase II trial of nivolumab and metformin in patients with treatment-refractory microsatellite stable metastatic colorectal cancer | No | Irrelevant |
| 1329 | | Witt et al., 2023 | | | Gut Microbiome in Patients with Early-Stage and Late-Stage Melanoma | No | Irrelevant |
| 1330 | | Yang et al., 2023 | | | Comprehensive genomic characterization of sporadic synchronous colorectal cancer: Implications for treatment optimization and clinical outcome | No | Irrelevant |
| 1331 | | Tripathi et al., 2023 | | | Role of Artificial Intelligence in High Throughput Diagnostics for Colorectal Cancer: Current Updates | No | Irrelevant |
| 1332 | | Saberzadeh-Ardestani et al., 2023 | | | Immune Marker Spatial Distribution and Clinical Outcome after PD-1 Blockade in Mismatch Repair–deficient, Advanced Colorectal Carcinomas | No | Irrelevant |
| 1333 | | Chandra et al., 2023 | | | Nanotechnology-empowered strategies in treatment of skin cancer | No | Irrelevant |
| 1334 | | Seufferlein et al., 2023 | | | Palliative Treatment of Metastatic Rectal Cancer | No | Irrelevant |
| 1335 | | El Naqa et al., 2023 | | | Translation of AI into oncology clinical practice | No | Irrelevant |
| 1336 | | Han et al., 2023 | | | Redirecting Antigens by Engineered Photosynthetic Bacteria and Derived Outer Membrane Vesicles for Enhanced Cancer Immunotherapy | No | Irrelevant |
| 1337 | | Chen et al., 2023 | | | Chemotherapy-Induced Neoantigen Nanovaccines Enhance Checkpoint Blockade Cancer Immunotherapy | No | Irrelevant |
| 1338 | | Ghorani et al., 2023 | | | Cancer cell-intrinsic mechanisms driving acquired immune tolerance | No | Irrelevant |
| 1339 | | Fridman et al., 2023 | | | Tertiary lymphoid structures and B cells: An intratumoral immunity cycle | No | Irrelevant |
| 1340 | | Ando et al., 2023 | | | Mutational spectrum of TP53 gene correlates with nivolumab treatment efficacy in advanced gastric cancer | No | Irrelevant |
| 1341 | | Wang et al., 2023 | | | Impact of Molecular Status on Metastasectomy of Colorectal Cancer Liver Metastases | No | Irrelevant |
| 1342 | | Yan et al., 2023 | | | RNA splicing alterations in lung cancer pathogenesis and therapy | No | Irrelevant |
| 1343 | | Wandrey et al., 2023 | | | Exosomes in Cancer Progression and Therapy Resistance: Molecular Insights and Therapeutic Opportunities | No | Irrelevant |
| 1344 | | Topchyan et al., 2023 | | | The Role of CD4 T Cell Help in CD8 T Cell Differentiation and Function During Chronic Infection and Cancer | No | Irrelevant |
| 1345 | | Huang et al., 2023 | | | Tracing the evolving dynamics and research hotspots of microbiota and immune microenvironment from the past to the new era | No | Irrelevant |
| 1346 | | Zhang et al., 2023 | | | Identification of Molecular Subtypes and Prognostic Characteristics of Adrenocortical Carcinoma Based on Unsupervised Clustering | No | Irrelevant |
| 1347 | | Hall et al., 2023 | | | The G Protein-Coupled Estrogen Receptor (GPER): A Critical Therapeutic Target for Cancer | No | Irrelevant |
| 1348 | | Heregger et al., 2023 | | | Unraveling Resistance to Immunotherapy in MSI-High Colorectal Cancer | No | Irrelevant |
| 1349 | | Lee et al., 2023 | | | Angiopoietin-2 blockade suppresses growth of liver metastases from pancreatic neuroendocrine tumors by promoting T cell recruitment | No | Irrelevant |
| 1350 | | García-Pérez et al., 2023 | | | Autophagy as a Target for Non-Immune Intrinsic Functions of Programmed Cell Death-Ligand 1 in Cancer | No | Irrelevant |
| 1351 | | Téllez et al., 2023 | | | Clusterin Expression in Colorectal Carcinomas | No | Irrelevant |
| 1352 | | Mendiola et al., 2023 | | | Comparison of Methods for Testing Mismatch Repair Status in Endometrial Cancer | No | Irrelevant |
| 1353 | | Ephraim et al., 2023 | | | Differential Gene Expression of Checkpoint Markers and Cancer Markers in Mouse Models of Spontaneous Chronic Colitis | No | Irrelevant |
| 1354 | | Minaguchi et al., 2023 | | | Molecular biomarkers for facilitating genome-directed precision medicine in gynecological cancer | No | Irrelevant |
| 1355 | | Jin et al., 2023 | | | Molecular classification of hormone receptor-positive HER2-negative breast cancer | No | Irrelevant |
| 1356 | | Khan et al., 2023 | | | Cancer metastasis: Molecular mechanisms and clinical perspectives | No | Irrelevant |
| 1357 | | Wang et al., 2023 | | | Pyroptosis and inflammasomes in cancer and inflammation | No | Irrelevant |
| 1358 | | Kong et al., 2023 | | | Analysis on the risk of myasthenia gravis related to immune checkpoint inhibitors | No | Irrelevant |
| 1359 | | Moreta-Moraleda et al., 2023 | | | Chromatin factors: Ready to roll as biomarkers in metastatic colorectal cancer? | No | Irrelevant |
| 1360 | | Cerella et al., 2023 | | | Enhancing personalized immune checkpoint therapy by immune archetyping and pharmacological targeting | No | Irrelevant |
| 1361 | | Li et al., 2023 | | | Thymol targeting interleukin 4 induced 1 expression reshapes the immune microenvironment to sensitize immunotherapy | No | Irrelevant |
| 1362 | | Liu et al., 2023 | | | Participation of protein metabolism in cancer progression and its potential targeting for the management of cancer | No | Irrelevant |
| 1363 | | Sun et al., 2023 | | | A novel bivalent anti-c-MET/PD-1 bispecific antibody exhibits potent cytotoxicity against c-MET/PD-L1-positive colorectal cancer | No | Irrelevant |
| 1364 | | Li et al., 2023 | | | Chemotherapy-induced nanovaccines implement immunogenicity equivalence for improving cancer chemoimmunotherapy | No | Irrelevant |
| 1365 | | Xing et al., 2023 | | | A scientometric analysis and up-to-date review of nano-based drug delivery systems in glioblastoma treatment | No | Irrelevant |
| 1366 | | Song et al., 2023 | | | IL-17A functions and the therapeutic use of IL-17A and IL-17RA targeted antibodies for cancer treatment | No | Irrelevant |
| 1367 | | Pascual-Antón et al., 2023 | | | Targeting carcinoma-associated mesothelial cells with antibody–drug conjugates in ovarian carcinomatosis | No | Irrelevant |
| 1368 | | Gupta et al., 2023 | | | Deciphering the complexities of cancer cell immune evasion: Mechanisms and therapeutic implications | No | Irrelevant |
| 1369 | | Bhamidipati et al., 2023 | | | Tumor-agnostic drug development in dMMR/MSI-H solid tumors | No | Irrelevant |
| 1370 | | Fu et al., 2023 | | | Poliovirus receptor (PVR) mediates carboplatin-induced PD-L1 expression in non-small-cell lung cancer cells | No | Irrelevant |
| 1371 | | Nagasaka et al., 2023 | | | Pan-tumor survey of RET fusions as detected by next-generation RNA sequencing | No | Irrelevant |
| 1372 | | Okubo et al., 2023 | | | Histological findings of thyroid cancer after lenvatinib therapy | No | Irrelevant |
| 1373 | | Sasaki et al., 2023 | | | Targeting the loss of cGAS/STING signaling in cancer | No | Irrelevant |
| 1374 | | Bolivar et al., 2023 | | | Advances in vaccine development for cancer prevention and treatment in Lynch Syndrome | No | Irrelevant |
| 1375 | | Baba et al., 2023 | | | Relationship between gut microbiome Fusobacterium nucleatum and LINE-1 methylation level in esophageal cancer | No | Irrelevant |
| 1376 | | Tao et al., 2023 | | | Orientin regulates the proliferation and migration of hepatocellular carcinoma cells | No | Irrelevant |
| 1377 | | Wu et al., 2023 | | | Intratumoral Microbiota Composition Regulates Chemoimmunotherapy Response in Esophageal Squamous Cell Carcinoma | No | Irrelevant |
| 1378 | | Liu et al., 2023 | | | Roles of Tumor-Associated Macrophages in Tumor Environment and Strategies for Targeting Therapy | No | Irrelevant |
| 1379 | | Qiu et al., 2023 | | | Alcohol reshapes a liver premetastatic niche for cancer by extra- and intrahepatic crosstalk-mediated immune evasion | No | Irrelevant |
| 1380 | | Hu et al., 2023 | | | Comprehensive analysis of cuproptosis-related gene expression and CD4+ T cell infiltration in head and neck squamous cell carcinoma | No | Irrelevant |
| 1381 | | Delcuratolo et al., 2023 | | | Liquid Biopsy in Advanced Colorectal Cancer: Clinical Applications of Different Analytes | No | Irrelevant |
| 1382 | | Putta et al., 2023 | | | Melatonin: Avenues in cancer therapy and its nanotechnological advancements | No | Irrelevant |
| 1383 | | Marabelli et al., 2023 | | | Editorial for the Special Issue “Genetics and Genomics of Gastrointestinal Cancers: From Prevention to Treatment” | No | Irrelevant |
| 1384 | | Aktar et al., 2023 | | | Correlation between KRAS Mutation and CTLA-4 mRNA Expression in Circulating Tumour Cells | No | Irrelevant |
| 1385 | | Otsu et al., 2023 | | | Current Status of Angiogenesis Inhibitors as Second-Line Treatment for Unresectable Colorectal Cancer | No | Irrelevant |
| 1386 | | Jin et al., 2023 | | | A Novel Lipid Metabolism and Endoplasmic Reticulum Stress-Related Risk Model for Colorectal Cancer | No | Irrelevant |
| 1387 | | Shakhpazyan et al., 2023 | | | Cellular and Molecular Mechanisms of the Tumor Stroma in Colorectal Cancer | No | Irrelevant |
| 1388 | | Bian et al., 2023 | | | Targeting DNA Damage Repair and Immune Checkpoint Proteins for Endometrial Cancer | No | Irrelevant |
| 1389 | | Liew et al., 2023 | | | Natural Products as Therapeutic or Chemopreventive Agents against Head and Neck Squamous Cell Carcinoma | No | Irrelevant |
| 1390 | | Yang et al., 2023 | | | Detection and quantitative analysis of tumor-associated tertiary lymphoid structures | No | Irrelevant |
| 1391 | | Guo et al., 2023 | | | Cuproptosis-Related 4-Gene Risk Model for Kidney Renal Clear Cell Carcinoma | No | Irrelevant |
| 1392 | | Sharma et al., 2023 | | | Therapeutic Antibodies in Medicine | No | Irrelevant |
| 1393 | | Suzuki et al., 2023 | | | Development of a Novel Anti-CD44 Variant 8 Monoclonal Antibody | No | Irrelevant |
| 1394 | | Bonilla et al., 2023 | | | Ibero-American Consensus Review and Incorporation of New Biomarkers for Colorectal Cancer | No | Irrelevant |
| 1395 | | Ros et al., 2023 | | | Immunotherapy for Colorectal Cancer with High Microsatellite Instability | No | Irrelevant |
| 1396 | | Okami et al., 2023 | | | HLA Class I Expression in Ulcerative Colitis-Associated Dysplasia | No | Irrelevant |
| 1397 | | Heidari et al., 2023 | | | The potential of monoclonal antibodies for colorectal cancer therapy | No | Irrelevant |
| 1398 | | Lamey et al., 2023 | | | Simultaneous encapsulation of dasatinib and celecoxib for breast cancer | No | Irrelevant |
| 1399 | | Haldar et al., 2023 | | | Emerging Strategies in Cancer Immunoprevention | No | Irrelevant |
| 1400 | | Aljama et al., 2023 | | | Dichotomous colorectal cancer behaviour | No | Irrelevant |
| 1401 | | Passey et al., 2023 | | | Exposure-safety and exposure-efficacy analyses for tisotumab vedotin for patients with locally advanced or metastatic solid tumors | No | Irrelevant |
| 1402 | | Schultz et al., 2023 | | | Children's Oncology Group's 2023 blueprint for research: Rare tumors | No | Irrelevant |
| 1403 | | Al Zein et al., 2023 | | | Immunotherapy and immunoevasion of colorectal cancer | No | - |
| 1404 | | Mosalem et al., 2023 | | | Tyrosine Kinase Inhibitors and Immunotherapy Updates in Neuroendocrine Neoplasms | No | Irrelevant |
| 1405 | | Liu et al., 2023 | | | Therapeutic drug monitoring of immune checkpoint inhibitors: based on their pharmacokinetic properties and biomarkers | No | Irrelevant |
| 1406 | | He et al., 2023 | | | CircNCOR1 regulates breast cancer radiotherapy efficacy by regulating CDK2 via hsa-miR-638 binding | No | Irrelevant |
| 1407 | | Guan et al., 2023 | | | Ginsenoside Rh2: A shining and potential natural product in the treatment of human nonmalignant and malignant diseases in the near future | No | Irrelevant |
| 1408 | | Merat, 2023 | | | The human antigen R as an actionable super-hub within the network of cancer cell persistency and plasticity | No | Irrelevant |
| 1409 | | Guan et al., 2023 | | | DNA mismatch repair in cancer immunotherapy | No | - |
| 1410 | | Kapturska et al., 2023 | | | New molecular targets in canine hemangiosarcoma—Comparative review and future of the precision medicine | No | Irrelevant |
| 1411 | | Mulet Margalef et al., 2023 | | | Genomically matched therapy in refractory colorectal cancer according to ESMO Scale for Clinical Actionability of Molecular Targets | No | - |
| 1412 | | Zhuang et al., 2023 | | | Gut microbiota interactions with antitumor immunity in colorectal cancer: From understanding to application | No | - |
| 1413 | | Grandhi et al., 2023 | | | Advances in systemic chemotherapy and immunotherapy for metastatic colorectal cancer | No | - |
| 1414 | | Yang et al., 2023 | | | Antigen presentation in cancer — mechanisms and clinical implications for immunotherapy | No | - |
| 1415 | | Lin et al., 2023 | | | Homologous recombination repair gene mutations in colorectal cancer favors treatment of immune checkpoint inhibitors | No | - |
| 1416 | | Liu et al., 2023 | | | Insight into immune checkpoint inhibitor therapy for colorectal cancer from the perspective of circadian clocks | No | - |
| 1417 | | Žilinskas et al., 2023 | | | HAMLET effect on cell death and mitochondrial respiration in colorectal cancer cell lines with KRAS/BRAF mutations | No | Irrelevant |
| 1418 | | Hsu et al., 2023 | | | B7-H3 drives immunosuppression and Co-targeting with CD47 is a new therapeutic strategy in β-catenin activated melanomas | No | Irrelevant |
| 1419 | | Rong et al., 2023 | | | The crosstalking of lactate-Histone lactylation and tumor | No | Irrelevant |
| 1420 | | Erali et al., 2023 | | | Utilizing Patient-Derived Organoids in the Management of Colorectal Cancer with Peritoneal Metastases: A Review of Current Literature | No | Irrelevant |
| 1421 | | Sokolowska et al., 2023 | | | Immune checkpoint inhibition improves antimyeloma activity of bortezomib and STING agonist combination in Vk*MYC preclinical model | No | Irrelevant |
| 1422 | | Ueno et al., 2023 | | | Colorectal cancer in adolescent and young adults: epidemiology in Japan and narrative review | No | - |
| 1423 | | Konda et al., 2023 | | | Genome-guided discovery of cancer therapeutic targets | No | Irrelevant |
| 1424 | | Quan et al., 2023 | | | Low molecular weight heparin synergistically enhances the efficacy of adoptive and anti-PD-1-based immunotherapy by increasing lymphocyte infiltration in colorectal cancer | No | - |
| 1425 | | Li et al., 2023 | | | Depicting the landscape of gut microbial-metabolic interaction and microbial-host immune heterogeneity in deficient and proficient DNA mismatch repair colorectal cancers | No | - |
| 1426 | | Hui et al., 2023 | | | Regulatory role of exosomes in colorectal cancer progression and potential as biomarkers | No | - |
| 1427 | | Qu et al., 2023 | | | Role of the Gut Microbiota and Its Metabolites in Tumorigenesis or Development of Colorectal Cancer | No | - |
| 1428 | | Wang et al., 2023 | | | How to overcome resistance to immune checkpoint inhibitors in colorectal cancer: From mechanisms to translation | No | - |
| 1429 | | Sweef et al., 2023 | | | Unraveling Therapeutic Opportunities and the Diagnostic Potential of microRNAs for Human Lung Cancer | No | Irrelevant |
| 1430 | | Serra et al., 2023 | | | Small Molecules against Metastatic Tumors: Concrete Perspectives and Shattered Dreams | No | Irrelevant |
| 1431 | | Chen et al., 2023 | | | Histone chaperone SSRP1 is required for apoptosis inhibition and mitochondrial function in HCC via transcriptional promotion of TRAP1 | No | Irrelevant |
| 1432 | | Koerner et al., 2023 | | | The Present and Future of Neoadjuvant and Adjuvant Therapy for Locally Advanced Gastric Cancer | No | Irrelevant |
| 1433 | | Maebele et al., 2023 | | | Immunomodulatory Gene-Splicing Dysregulation in Tumorigenesis: Unmasking the Complexity | No | Irrelevant |
| 1434 | | Cha et al., 2023 | | | Expression of EMP 1, 2, and 3 in Adrenal Cortical Neoplasm and Pheochromocytoma | No | Irrelevant |
| 1435 | | Ascrizzi et al., 2023 | | | Lynch Syndrome Biopathology and Treatment: The Potential Role of microRNAs in Clinical Practice | No | - |
| 1436 | | Tosti et al., 2023 | | | Vaccination and Microbiota Manipulation Approaches for Colon Cancer Prevention in Rodent Models | No | Irrelevant |
| 1437 | | Hongyuan et al., 2023 | | | Exploration of novel clusters and prognostic value of immune-related signatures and identify HAMP as hub gene in colorectal cancer | No | - |
| 1438 | | Thibaudin et al., 2023 | | | First-line durvalumab and tremelimumab with chemotherapy in RAS-mutated metastatic colorectal cancer: a phase 1b/2 trial | No | - |
| 1439 | | Kasago et al., 2023 | | | Undifferentiated and Dedifferentiated Metastatic Melanomas Masquerading as Soft Tissue Sarcomas: Mutational Signature Analysis and Immunotherapy Response | No | Irrelevant |
| 1440 | | Ren et al., 2023 | | | The recent progress of myeloid-derived suppressor cell and its targeted therapies in cancers | No | Irrelevant |
| 1441 | | Wang et al., 2023 | | | Elucidation of the anti-lung cancer mechanism of Juan-Liu-San-Jie prescription based on network pharmacology and experimental validation | No | Irrelevant |
| 1442 | | Taniai et al., 2023 | | | Dynamics of the prognostic nutritional index in preoperative chemotherapy in patients with colorectal liver metastases | No | - |
| 1443 | | Xu et al., 2023 | | | Identification of DNA Damage Repair Gene Signature as a Novel Prognostic Marker in Glioblastoma Multiforme | No | Irrelevant |
| 1444 | | Dumontet et al., 2023 | | | Antibody–drug conjugates come of age in oncology | No | Irrelevant |
| 1445 | | Dermawan et al., 2023 | | | The spectrum and significance of secondary (co-occurring) genetic alterations in sarcomas: the hallmarks of sarcomagenesis | No | Irrelevant |
| 1446 | | Wang et al., 2023 | | | Current and emerging applications of liquid biopsy in pan-cancer | No | Irrelevant |
| 1447 | | Boccaccino et al., 2023 | | | Adverse events during first-line treatments for mCRC: The Toxicity over Time (ToxT) analysis of three randomised trials | No | - |
| 1448 | | Dao et al., 2023 | | | Beyond EGFR inhibitors in advanced colorectal cancer: Targeting BRAF and HER2 | No | - |
| 1449 | | Dote et al., 2023 | | | Impact of prior bevacizumab therapy on the incidence of ramucirumab-induced proteinuria in colorectal cancer: a multi-institutional cohort study | No | - |
| 1450 | | Mekala et al., 2023 | | | Novel sights on therapeutic, prognostic, and diagnostics aspects of non-coding RNAs in glioblastoma multiforme | No | Irrelevant |
| 1451 | | Janssens et al., 2023 | | | New Developments in Treating RAS-Mutated Metastatic Colorectal Cancer | No | - |
| 1452 | | Stam et al., 2023 | | | The effect of anastomotic leakage on the incidence of recurrence after tri-modality therapy for esophageal adenocarcinomas | No | Irrelevant |
| 1453 | | Pan et al., 2023 | | | Targeting CXCL9/10/11–CXCR3 axis: an important component of tumor-promoting and antitumor immunity | No | - |
| 1454 | | Sato et al., 2023 | | | The roles of tertiary lymphoid structures in chronic diseases | No | Irrelevant |
| 1455 | | Chandrababu et al., 2023 | | | Green Synthesized Nanoparticles as a Plausible Therapeutic Strategy Against Hepatocellular Carcinoma: An Update on its Preclinical and Clinical Relevance | No | Irrelevant |
| 1456 | | Zhang et al., 2023 | | | Tertiary Lymphoid Structures Are Associated with a Favorable Prognosis in High-Grade Serous Ovarian Cancer Patients | No | Irrelevant |
| 1457 | | Matsunaga et al., 2023 | | | The prognostic impact of the lymphocyte-to-C-reactive protein ratio in patients with unresectable or recurrent advanced gastric cancer treated with first- and second-line treatment | No | Irrelevant |
| 1458 | | Angeles et al., 2023 | | | Integrated circulating tumour DNA and cytokine analysis for therapy monitoring of ALK-rearranged lung adenocarcinoma | No | Irrelevant |
| 1459 | | Geng et al., 2023 | | | Rubiginosin B selectively inhibits Treg cell differentiation and enhances anti-tumor immune responses by targeting calcineurin-NFAT signaling pathway | No | Irrelevant |
| 1460 | | Kornepati et al., 2023 | | | The complementarity of DDR, nucleic acids and anti-tumour immunity | No | Irrelevant |
| 1461 | | Lu et al., 2023 | | | Tumor and local lymphoid tissue interaction determines prognosis in high-grade serous ovarian cancer | No | Irrelevant |
| 1462 | | Whitefield et al., 2023 | | | Complex Inhibitory Mechanism of Glycomimetics with Heparanase | No | Irrelevant |
| 1463 | | Talwar et al., 2023 | | | Autoimmune alleles at the major histocompatibility locus modify melanoma susceptibility | No | Irrelevant |
| 1464 | | Floyd et al., 2023 | | | Atrx deletion impairs CGAS/STING signaling and increases sarcoma response to radiation and oncolytic herpesvirus | No | Irrelevant |
| 1465 | | Zhang et al., 2023 | | | Research progress in immune checkpoint inhibitors treatment of advanced biliary tract cancer | No | Irrelevant |
| 1466 | | Kaur et al., 2023 | | | Genomic biomarkers: Unveiling the potential for precise cancer therapy response | No | Irrelevant |
| 1467 | | Pandey et al., 2023 | | | The Local Microbiome in Esophageal Cancer and Treatment Response: A Review of Emerging Data and Future Directions | No | Irrelevant |
| 1468 | | Khalili et al., 2023 | | | Macrophage-Based Therapeutic Strategies in Hematologic Malignancies | No | Irrelevant |
| 1469 | | Martinis et al., 2023 | | | Cancer Vaccines: From the State of the Art to the Most Promising Frontiers in the Treatment of Colorectal Cancer | No | - |
| 1470 | | Kumar et al., 2023 | | | TIME Is Ticking for Cervical Cancer | No | Irrelevant |
| 1471 | | Zheng et al., 2023 | | | T Cells in Colorectal Cancer: Unravelling the Function of Different T Cell Subsets in the Tumor Microenvironment | No | - |
| 1472 | | Ray et al., 2023 | | | An Overview of Selected Bacterial Infections in Cancer, Their Virulence Factors, and Some Aspects of Infection Management | No | Irrelevant |
| 1473 | | Amin et al., 2023 | | | TP5: A Novel Therapeutic Approach Targeting Aberrant and Hyperactive CDK5/p25 for the Treatment of Colorectal Carcinoma | No | - |
| 1474 | | Pous et al., 2023 | | | HER2-Positive Gastric Cancer: The Role of Immunotherapy and Novel Therapeutic Strategies | No | Irrelevant |
| 1475 | | Wen et al., 2023 | | | Insight into Cancer Immunity: MHCs, Immune Cells and Commensal Microbiota | No | - |
| 1476 | | Grypari et al., 2023 | | | DNA Damage Repair Pathways in Prostate Cancer: A Narrative Review of Molecular Mechanisms, Emerging Biomarkers and Therapeutic Targets in Precision Oncology | No | Irrelevant |
| 1477 | | Mendes et al., 2023 | | | How Can the Microbiome Induce Carcinogenesis and Modulate Drug Resistance in Cancer Therapy? | No | Irrelevant |
| 1478 | | An et al., 2023 | | | Expression of Immune-Related and Inflammatory Markers and Their Prognostic Impact in Colorectal Cancer Patients | No | - |
| 1479 | | Vuković Đerfi et al., 2023 | | | EMAST Type of Microsatellite Instability—A Distinct Entity or Blurred Overlap between Stable and MSI Tumors | No | - |
| 1480 | | Kano et al., 2023 | | | Strategic Insight into the Combination Therapies for Metastatic Colorectal Cancer | No | - |
| 1481 | | Su et al., 2023 | | | Crosstalk between gut microbiota and RNA N6-methyladenosine modification in cancer | No | Irrelevant |
| 1482 | | Tang et al., 2023 | | | TIGIT, a novel immune checkpoint therapy for melanoma | No | Irrelevant |
| 1483 | | Zhuang et al., 2023 | | | Single-cell transcriptome analysis reveals T population heterogeneity and functions in tumor microenvironment of colorectal cancer metastases | No | - |
| 1484 | | Viculin et al., 2023 | | | Elevated Tumor Cell-Intrinsic STING Expression in Advanced Laryngeal Cancer | No | Irrelevant |
| 1485 | | Foote et al., 2023 | | | The Impact of Germline Alterations in Appendiceal Adenocarcinoma | No | Irrelevant |
| 1486 | | Stephan-Falkenau et al., 2023 | | | Integrated Clinical, Molecular and Immunological Characterization of Pulmonary Sarcomatoid Carcinomas Reveals an Immune Escape Mechanism That May Influence Therapeutic Strategies | No | Irrelevant |
| 1487 | | Ruff et al., 2023 | | | The Role of Targeted Therapy in the Multi-Disciplinary Approach to Colorectal Liver Metastasis | No | - |
| 1488 | | Liang et al., 2023 | | | KRT17 Promotes T-lymphocyte Infiltration Through the YTHDF2–CXCL10 Axis in Colorectal Cancer | No | - |
| 1489 | | Alvaro et al., 2023 | | | EASL-ILCA Clinical Practice Guidelines on the management of intrahepatic cholangiocarcinoma | No | Irrelevant |
| 1490 | | Jin et al., 2023 | | | Histone modifications in drug-resistant cancers: From a cancer stem cell and immune evasion perspective | No | Irrelevant |
| 1491 | | Pandya et al., 2023 | | | The future of cancer immunotherapy: DNA vaccines leading the way | No | Irrelevant |
| 1492 | | Calheiros et al., 2023 | | | Overcoming therapeutic resistance in pancreatic cancer: Emerging opportunities by targeting BRCAs and p53 | No | Irrelevant |
| 1493 | | Delaye et al., 2023 | | | Obesity, cancer, and response to immune checkpoint inhibitors: Could the gut microbiota be the mechanistic link? | No | Irrelevant |
| 1494 | | Lin et al., 2023 | | | Factors predictive of clinical outcome in advanced hepatocellular carcinoma patients receiving ramucirumab treatment: A real-world experience | No | Irrelevant |
| 1495 | | Zhao et al., 2023 | | | TIGIT: An emerging immune checkpoint target for immunotherapy in autoimmune disease and cancer | No | Irrelevant |
| 1496 | | Azimnasab-sorkhabi et al., 2023 | | | Indoleamine-2,3 dioxygenase: a fate-changer of the tumor microenvironment | No | Irrelevant |
| 1497 | | Hong et al., 2023 | | | Metronomic chemotherapy as a potential partner of immune checkpoint inhibitors for metastatic colorectal cancer treatment | No | - |
| 1498 | | Mortezaee, 2023 | | | B7-H3 immunoregulatory roles in cancer | No | Irrelevant |
| 1499 | | Sun et al., 2023 | | | A recombinant oncolytic influenza virus expressing a PD-L1 antibody induces CD8+ T-cell activation via the cGas-STING pathway in mice with hepatocellular carcinoma | No | Irrelevant |
| 1500 | | Wong et al., 2023 | | | Gut microbiota in colorectal cancer development and therapy | No | - |
| 1501 | | Mortaezaee et al., 2023 | | | Mechanisms of CD8+ T cell exclusion and dysfunction in cancer resistance to anti-PD-(L)1 | No | Irrelevant |
| 1502 | | Niu et al., 2023 | | | Ginsenoside Rb1 for overcoming cisplatin-insensitivity of A549/DDP cells in vitro and vivo through dual-inhibition on two efflux pumps of ABCB1 and PTCH1 | No | Irrelevant |
| 1503 | | Chauhan et al., 2023 | | | Human small intestine contains 2 functionally distinct regulatory T-cell subsets | No | Irrelevant |
| 1504 | | Strating et al., 2023 | | | Fibroblast Activation Protein Inhibitor-PET Imaging in Colorectal Cancer | No | - |
| 1505 | | Sun et al., 2023 | | | Classification of Tumor Immune Microenvironment According to Programmed Death-Ligand 1 Expression and Immune Infiltration Predicts Response to Immunotherapy Plus Chemotherapy in Advanced Patients With NSCLC | No | Irrelevant |
| 1506 | | Wu et al., 2023 | | | Thyroid dysfunction after immune checkpoint inhibitor treatment in a single-center Chinese cohort: a retrospective study | No | Irrelevant |
| 1507 | | Liu et al., 2023 | | | N6-methyladenosine long non-coding RNAs reveal novel tool to implicate overall survival and immune microenvironment in renal clear cell carcinoma | No | Irrelevant |
| 1508 | | Kumar et al., 2023 | | | Targeting PD-1/PD-L1 in cancer immunotherapy: An effective strategy for treatment of triple-negative breast cancer (TNBC) patients | No | Irrelevant |
| 1509 | | Corchado-Cobos et al., 2023 | | | Cutaneous squamous cell carcinoma: From biology to therapy | No | Irrelevant |
| 1510 | | Kashyap et al., 2023 | | | Exploring the complex and multifaceted interplay of the gut microbiome and cancer prevention and therapy | No | Irrelevant |
| 1511 | | Park et al., 2023 | | | Angiopoietin-2–Dependent Spatial Vascular Destabilization Promotes T-cell Exclusion and Limits Immunotherapy in Melanoma | No | Irrelevant |
| 1512 | | Leiphrakpam et al., 2023 | | | Colorectal cancer: Review of signaling pathways and associated therapeutic strategies | No | - |
| 1513 | | Ghalehbandi et al., 2023 | | | The role of VEGF in cancer-induced angiogenesis and research progress of drugs targeting VEGF | No | Irrelevant |
| 1514 | | Zhao et al., 2023 | | | New perspectives for targeting therapy in ALK-positive human cancers | No | Irrelevant |
| 1515 | | Qadir et al., 2023 | | | CircRNAs regulate the crosstalk between inflammation and tumorigenesis: The bilateral association and molecular mechanisms | No | Irrelevant |
| 1516 | | Segatori et al., 2023 | | | Mimicry of Tumour-Associated Carbohydrates: Is It a Promising Option for Cancer Treatment? | No | Irrelevant |
| 1517 | | Heinrich et al., 2023 | | | Case of a Patient With Pancreatic Cancer With Sporadic Microsatellite Instability Associated With a BRAF Fusion Achieving Excellent Response to Immunotherapy | No | Irrelevant |
| 1518 | | Jeanne et al., 2023 | | | French recommendations for clinical practice Nice-Saint-Paul de Vence 2022-2023: Histomolecular diagnosis of endometrial carcinomas | No | Irrelevant |
| 1519 | | Wang et al., 2023 | | | Systematic Review of Economic Evaluation of Targeted Drugs for Colorectal Cancer Treatment in China | No | Irrelevant |
| 1520 | | Greco et al., 2023 | | | Microsatellite Instability and Immune Response: From Microenvironment Features to Therapeutic Actionability—Lessons from Colorectal Cancer | No | - |
| 1521 | | Chmiel et al., 2023 | | | Vista of the Future: Novel Immunotherapy Based on the Human V-Set Immunoregulatory Receptor for Digestive System Tumors | No | Irrelevant |
| 1522 | | Papa et al., 2023 | | | The Role of Microbiota in Pancreatic Cancer | No | Irrelevant |
| 1523 | | Giacomelli et al., 2023 | | | Immuno-Contexture and Immune Checkpoint Molecule Expression in Mismatch Repair Proficient Colorectal Carcinoma | No | - |
| 1524 | | Mogavero et al., 2023 | | | Deciphering Lung Adenocarcinoma Heterogeneity: An Overview of Pathological and Clinical Features of Rare Subtypes | No | Irrelevant |
| 1525 | | Dutta et al., 2023 | | | Neutrophils in Cancer and Potential Therapeutic Strategies Using Neutrophil-Derived Exosomes | No | Irrelevant |
| 1526 | | Koufopoulos et al., 2023 | | | A Case of Prostatic Signet-Ring Cell-like Carcinoma with Pagetoid Spread and Intraductal Carcinoma and Long-Term Survival | No | Irrelevant |
| 1527 | | Seok et al., 2023 | | | Beyond DNA sensing: expanding the role of cGAS/STING in immunity and diseases | No | Irrelevant |
| 1528 | | Constantin et al., 2023 | | | Pregnancy and Gastric Cancer: A Narrative Review | No | Irrelevant |
| 1529 | | Yang et al., 2023 | | | A Multi-Omics Overview of Colorectal Cancer to Address Mechanisms of Disease, Metastasis, Patient Disparities and Outcomes | No | - |
| 1530 | | Xin et al., 2023 | | | Natural fulvic acids inhibit non-small-cell lung cancer through the COX-2/PGE2/EP4 axis | No | Irrelevant |
| 1531 | | Manzi et al., 2023 | | | Targeted Therapies in Colorectal Cancer: Recent Advances in Biomarkers, Landmark Trials, and Future Perspectives | No | - |
| 1532 | | Chen et al., 2023 | | | Modeling the Tumor Microenvironment and Cancer Immunotherapy in Next-Generation Humanized Mice | No | Irrelevant |
| 1533 | | Zhang et al., 2023 | | | Construction and validation of a chemokine family-based signature for the prediction of prognosis and therapeutic response in colon cancer | No | - |
| 1534 | | Yoshino et al., 2023 | | | Pan-Asian adapted ESMO Clinical Practice Guidelines for the diagnosis, treatment and follow-up of patients with metastatic colorectal cancer | No | - |
| 1535 | | Nassiri et al., 2023 | | | Oncolytic DNX-2401 virotherapy plus pembrolizumab in recurrent glioblastoma: a phase 1/2 trial | No | Irrelevant |
| 1536 | | Liu et al., 2023 | | | Human leukocyte antigen and tumor immunotherapy | No | Irrelevant |
| 1537 | | Wang et al., 2023 | | | Molecular subtyping in colorectal cancer: A bridge to personalized therapy | No | - |
| 1538 | | Deshmukh et al., 2023 | | | A review on emerging targeted therapies for the management of metastatic colorectal cancers | No | - |
| 1539 | | Noack et al., 2023 | | | Molecular pathology of colorectal cancer | No | - |
| 1540 | | Martini et al., 2023 | | | Establishment of patient-derived tumor organoids to functionally inform treatment decisions in metastatic colorectal cancer | No | - |
| 1541 | | Ioffe et al., 2023 | | | Guidance for Treating the Older Adults with Colorectal Cancer | No | - |
| 1542 | | Liang et al., 2023 | | | Targeting histone deacetylases for cancer therapy: Trends and challenges | No | Irrelevant |
| 1543 | | Cao et al., 2023 | | | Targeting angiogenesis in oncology, ophthalmology and beyond | No | Irrelevant |
| 1544 | | Voutsadakis et al., 2023 | | | KRAS mutated colorectal cancers with or without PIK3CA mutations: Clinical and molecular profiles inform current and future therapeutics | No | - |
| 1545 | | Yang et al., 2023 | | | Overexpression of complement C5a indicates poor survival in metastatic renal cell carcinoma | No | Irrelevant |
| 1546 | | Quaas, A., 2023 | | | Therapeutically relevant predictive biomarkers in esophageal adenocarcinoma | No | Irrelevant |
| 1547 | | Li, T. et al., 2023 | | | Artificial intelligence in cancer immunotherapy: Applications in neoantigen recognition, antibody design and immunotherapy response prediction | No | Irrelevant |
| 1548 | | Fang, B. et al., 2023 | | | Prevalence of mismatch repair gene mutations and clinical activity of PD-1 therapy in Chinese prostate cancer patients | No | Irrelevant |
| 1549 | | Zhang, C. et al., 2023 | | | Colorectal cancer | No | - |
| 1550 | | Yang, R. et al., 2023 | | | Patient-derived organoids in translational oncology and drug screening | No | Irrelevant |
| 1551 | | Demerlé, C. et al., 2023 | | | Anti-HVEM mAb therapy improves antitumoral immunity in a transgenic mouse model | No | Irrelevant |
| 1552 | | Mertens, R.T. et al., 2023 | | | Next Generation Gold Drugs and Probes: Chemistry and Biomedical Applications | No | Irrelevant |
| 1553 | | Abbaszadeh, F. et al., 2023 | | | Pathophysiology of gastrointestinal tract cancers and therapeutic status | No | Irrelevant |
| 1554 | | Wang, Y. et al., 2023 | | | Short-course radiotherapy combined with CAPOX and PD-1 inhibitor for locally advanced rectal cancer | No | - |
| 1555 | | Kesar, U. et al., 2023 | | | Effects of Electrochemotherapy on Immunologically Important Modifications in Tumor Cells | No | Irrelevant |
| 1556 | | Losurdo, G. et al., 2023 | | | Checkpoint Inhibitor-Induced Colitis: An Update | No | Irrelevant |
| 1557 | | Rejali, L. et al., 2023 | | | Principles of Molecular Utility for CMS Classification in Colorectal Cancer Management | No | - |
| 1558 | | Ziogas, D.C. et al., 2023 | | | Beyond CTLA-4 and PD-1 Inhibition: Novel Immune Checkpoint Molecules for Melanoma Treatment | No | Irrelevant |
| 1559 | | Nakajima, S. et al., 2023 | | | Impact of cGAS-STING on CD8+ T Cells in Mismatch Repair Proficient Colorectal Cancer | No | - |
| 1560 | | Wang, C. et al., 2023 | | | Changes in Tumor Immune Microenvironment after Radiotherapy Resistance in Colorectal Cancer | No | - |
| 1561 | | Mohanty, S.K. et al., 2023 | | | Precision Medicine in Bladder Cancer: Present Challenges and Future Directions | No | Irrelevant |
| 1562 | | Kansal, R. et al., 2023 | | | Selected topics for precision diagnosis in lung, gastrointestinal, gynecological, and male genitourinary cancers | No | Irrelevant |
| 1563 | | Koukourakis, I.M. et al., 2023 | | | Immune Response and Immune Checkpoint Molecules in Rectal Cancer | No | - |
| 1564 | | Roelands, J. et al., 2023 | | | An integrated tumor, immune and microbiome atlas of colon cancer | No | - |
| 1565 | | Husain, M. et al., 2023 | | | Emerging Trends in Immunotherapy for Adult Sarcomas | No | Irrelevant |
| 1566 | | Bure, I.V. et al., 2023 | | | Mutual Regulation of ncRNAs and Chromatin Remodeling Complexes in Cancer | No | Irrelevant |
| 1567 | | Nakayama, I. et al., 2023 | | | Angiogenesis Targeted Therapies in Metastatic Advanced Gastric Cancer | No | Irrelevant |
| 1568 | | Cheng, S.-H. et al., 2023 | | | Reciprocal Regulation of Cancer-Associated Fibroblasts in Gastrointestinal Cancer | No | Irrelevant |
| 1569 | | Valerio, T.I. et al., 2023 | | | Immune Modulations of the Tumor Microenvironment in Response to Phototherapy | No | Irrelevant |
| 1570 | | Crowder, S.L. et al., 2023 | | | Gut Microbiome and Cancer: Potential Opportunities for Fermented Foods | No | Irrelevant |
| 1571 | | Vasiyani, H. et al., 2023 | | | Regulation of cGAS-STING Signalling in Cancer | No | Irrelevant |
| 1572 | | Wang, J. et al., 2023 | | | N6-Methyladenosine–Mediated Up-Regulation of FZD10 in Liver Cancer Stem Cells | No | Irrelevant |
| 1573 | | Mendoza, R.P. et al., 2023 | | | Endometrial Carcinomas with Subclonal Loss of Mismatch Repair Proteins | No | Irrelevant |
| 1574 | | Mohanty, S.K. et al., 2023 | | | Role of Surgical Pathologist for Detection of Immuno-oncologic Predictive Factors | No | Irrelevant |
| 1575 | | Nakamura, M. et al., 2023 | | | Molecular Genetic Positioning of Small Intestine and Papilla of Vater Carcinomas | No | Irrelevant |
| 1576 | | Jamal, R. et al., 2023 | | | Future Indications for Fecal Microbiota Transplantation in Immuno-oncology | No | Irrelevant |
| 1577 | | Chen, Y. et al., 2023 | | | S100A8 and S100A9 in Cancer | No | Irrelevant |
| 1578 | | Liu, Y. et al., 2023 | | | Genomic and Transcriptomic Insights into Precision Treatment of Pulmonary Enteric Adenocarcinoma | No | Irrelevant |
| 1579 | | Agaimy, A. et al., 2023 | | | SWI/SNF-deficient Malignancies: Optimal Candidates for Immunotherapy? | No | Irrelevant |
| 1580 | | Safaroghli-Azar, A. et al., 2023 | | | Kinase Inhibitors: Opportunities for Small Molecule Anticancer Immunotherapies | No | Irrelevant |
| 1581 | | Rossini, D. et al., 2023 | | | Primary Tumour Side as a Driver for Treatment Choice in Metastatic Colorectal Cancer | No | - |
| 1582 | | Li, T. et al., 2023 | | | Plasma Exosome-Derived circGAPVD1 as a Potential Diagnostic Marker for Colorectal Cancer | No | - |
| 1583 | | Ye, J. et al., 2023 | | | Dual-Target Small Molecules to Overcome Drug Resistance in Cancer Therapy | No | Irrelevant |
| 1584 | | Najafi, S. et al., 2023 | | | Impact of Oncolytic Adenoviral Therapy on PD-1/PD-L1 Blockade | No | Irrelevant |
| 1585 | | Feng, T. et al., 2023 | | | PDZ-Binding Kinase Aggravates Pancreatic Neuroendocrine Neoplasm Progression | No | Irrelevant |
| 1586 | | Einhaus, J. et al., 2023 | | | High-multiplex tissue imaging in routine pathology—are we there yet? | No | Irrelevant |
| 1587 | | Inaguma, S. et al., 2023 | | | CD70 and PD-L1 (CD274) co-expression predicts poor clinical outcomes in pleural mesothelioma | No | Irrelevant |
| 1588 | | Bando, H. et al., 2023 | | | Therapeutic landscape and future direction of metastatic colorectal cancer | No | - |
| 1589 | | Wang, Y. et al., 2023 | | | Cancer Immunology: Immune Escape of Tumors - Expression and Regulation of HLA Class I Molecules | No | Irrelevant |
| 1590 | | Kaur, M. et al., 2023 | | | Nanomaterials for Diagnosis and Treatment of Lung Cancer | No | Irrelevant |
| 1591 | | Ray, S.K. et al., 2023 | | | Altering Landscape of Cancer Vaccines: Unique Platforms and Therapeutic Applications | No | Irrelevant |
| 1592 | | Gao, J. et al., 2023 | | | Transcriptomic characterization of M2 macrophage-related prognostic signature in ovarian metastasis of gastric cancer | No | Irrelevant |
| 1593 | | Heitz, N. et al., 2023 | | | A Review of Tisotumab Vedotin-tftv in Recurrent or Metastatic Cervical Cancer | No | Irrelevant |
| 1594 | | Ma, S. et al., 2023 | | | Efficacy and safety of toripalimab with fruquintinib in refractory metastatic colorectal cancer | No | - |
| 1595 | | Li, C.M.Y. et al., 2023 | | | Cytokine-induced killer cell therapy in colorectal cancer: A systematic review and meta-analysis | No | - |
| 1596 | | Heath, B.R. et al., 2023 | | | Saturated fatty acids dampen immunogenicity of cancer by suppressing STING | No | Irrelevant |
| 1597 | | Orsolya, M. et al., 2023 | | | Current diagnostic and treatment strategies for urachal cancer | No | Irrelevant |
| 1598 | | He, M. et al., 2023 | | | Intratumoral tertiary lymphoid structure maturation influenced by lung cancer lymph nodes | No | Irrelevant |
| 1599 | | Shen, Y. et al., 2023 | | | Role of tumor-associated macrophages in common digestive system malignant tumors | No | Irrelevant |
| 1600 | | Halbrook, C.J. et al., 2023 | | | Pancreatic cancer: Advances and challenges | No | Irrelevant |
| 1601 | | Ren, S.-N. et al., 2023 | | | Application of nanotechnology in reversing therapeutic resistance and controlling metastasis of colorectal cancer | No | Irrelevant |
| 1602 | | Zekri, L. et al., 2023 | | | An optimized IgG-based B7-H3xCD3 bispecific antibody for treatment of gastrointestinal cancers | No | Irrelevant |
| 1603 | | Little, A. et al., 2023 | | | Fusobacterium nucleatum: A novel immune modulator in breast cancer? | No | Irrelevant |
| 1604 | | Tang, P.-X. et al., 2023 | | | Data mining and analysis of adverse events induced by ramucirumab | No | Irrelevant |
| 1605 | | Monné Rodríguez, J.M. et al., 2023 | | | Review of In Situ Hybridization Techniques for Drug Research and Development | No | Irrelevant |
| 1606 | | Yang, Y. et al., 2023 | | | Immunotherapy with Immune Checkpoint Inhibitors for Advanced Colorectal Cancer | No | - |
| 1607 | | Zhou, Y.-J. et al., 2023 | | | PD-L1: expression regulation | No | Irrelevant |
| 1608 | | Du, Y.-X. et al., 2023 | | | Natural Compounds Targeting the Autophagy Pathway in Colorectal Cancer | No | Irrelevant |
| 1609 | | Zhao, J. et al., 2023 | | | Organoids as an Enabler of Precision Immuno-Oncology | No | Irrelevant |
| 1610 | | Digomann, D. et al., 2023 | | | VISTA Ligation Reduces Antitumor T-Cell Activity in Pancreatic Cancer | No | Irrelevant |
| 1611 | | Vinokurova, D. et al., 2023 | | | The Emerging Role of IL-9 in the Anticancer Effects of Anti-PD-1 Therapy | No | Irrelevant |
| 1612 | | Khan, I. et al., 2023 | | | Human Papilloma Virus: An Unraveled Enigma of Universal Burden of Malignancies | No | Irrelevant |
| 1613 | | Lu, Y. et al., 2023 | | | Fluoropyrimidine-Associated Cardiotoxicity in Cancer Patients: A Systematic Review and Meta-Analysis | No | Irrelevant |
| 1614 | | Tria, S.M. et al., 2023 | | | The Therapeutic Landscape for KRAS-Mutated Colorectal Cancers | No | - |
| 1615 | | Ornella, M.S.C. et al., 2023 | | | Immunotherapy for Peritoneal Carcinomatosis: Challenges and Prospective Outcomes | No | Irrelevant |
| 1616 | | Kavun, A. et al., 2023 | | | Microsatellite Instability: Molecular Epidemiology and Immune Checkpoint Inhibitor Therapy | No | - |
| 1617 | | Lanka, S.M. et al., 2023 | | | Metastatic Castration-Resistant Prostate Cancer, Immune Checkpoint Inhibitors, and Beyond | No | Irrelevant |
| 1618 | | Műzes, G. et al., 2023 | | | Autoimmunity and Carcinogenesis: Their Relationship under the Umbrella of Autophagy | No | Irrelevant |
| 1619 | | Fukuda, K., 2023 | | | Immune Regulation by Cytosolic DNA Sensors in the Tumor Microenvironment | No | Irrelevant |
| 1620 | | Jiao, Y. et al., 2023 | | | The Roles of Innate Lymphoid Cells in Gastric Mucosal Immunology and Oncogenesis of Gastric Cancer | No | Irrelevant |
| 1621 | | Shimozaki, K. et al., 2023 | | | Current Strategy to Treat Immunogenic Gastrointestinal Cancers | No | - |
| 1622 | | Gielecińska, A. et al., 2023 | | | Substances of Natural Origin in Medicine: Plants vs. Cancer | No | Irrelevant |
| 1623 | | Ebrahimi, N. et al., 2023 | | | Receptor tyrosine kinase inhibitors in cancer | No | Irrelevant |
| 1624 | | Martinelli, E. et al., 2023 | | | Clinical management of BRAFV600E-mutant metastatic colorectal cancer | No | - |
| 1625 | | AmeliMojarad, M. et al., 2023 | | | Prospective role of PD-1/PD-L1 immune checkpoint inhibitors in GI cancer | No | - |
| 1626 | | Shin, A.E. et al., 2023 | | | Metastatic colorectal cancer: mechanisms and emerging therapeutics | No | - |
| 1627 | | Gu, Y. et al., 2023 | | | Harnessing epithelial-mesenchymal plasticity to boost cancer immunotherapy | No | Irrelevant |
| 1628 | | van Weverwijk, A. et al., 2023 | | | Mechanisms driving the immunoregulatory function of cancer cells | No | Irrelevant |
| 1629 | | Lee, S.L. et al., 2023 | | | The Role of Stereotactic Body Radiation Therapy in Liver Metastases | No | Irrelevant |
| 1630 | | Chen, X. et al., 2023 | | | Prognostic Value of Programmed Death Ligand-1 in Pancreatic Ductal Adenocarcinoma | No | Irrelevant |
| 1631 | | Bhargava, P. et al., 2023 | | | Demographic Profiles and Survival Outcomes of Urachal Adenocarcinoma | No | Irrelevant |
| 1632 | | Joharatnam-Hogan, N. et al., 2023 | | | Diabetes and cancer: Optimising glycaemic control | No | Irrelevant |
| 1633 | | Zhu, M. et al., 2023 | | | Understanding Suboptimal Response to Immune Checkpoint Inhibitors | No | Irrelevant |
| 1634 | | Chen, M. et al., 2023 | | | Mutation Classifier for Predicting Immune Checkpoint Inhibitor Therapy in Renal Cell Carcinoma | No | Irrelevant |
| 1635 | | Li, J. et al., 2023 | | | Immunoproteasome inhibition prevents progression of castration-resistant prostate cancer | No | Irrelevant |
| 1636 | | Canè, S. et al., 2023 | | | Neutralization of NET-associated human ARG1 enhances cancer immunotherapy | No | Irrelevant |
| 1637 | | Kennel, K.B. et al., 2023 | | | Cancer-Associated Fibroblasts in Inflammation and Antitumor Immunity | No | Irrelevant |
| 1638 | | Shashni, B. et al., 2023 | | | Short-chain fatty acid-releasing nano-prodrugs for attenuating melanoma growth | No | Irrelevant |
| 1639 | | Melendez-Alafort, L. et al., 2023 | | | Zirconium immune-complexes for PET molecular imaging | No | Irrelevant |
| 1640 | | Byeon, S.-J. et al., 2023 | | | Prognostic roles of leptin-signaling proteins in biliary tract cancers | No | Irrelevant |
| 1641 | | Leowattana, W. et al., 2023 | | | Systemic treatment for metastatic colorectal cancer | No | - |
| 1642 | | Klement, J.D. et al., 2023 | | | Tumor PD-L1 engages myeloid PD-1 to suppress type I interferon to impair cytotoxic T lymphocyte recruitment | No | Irrelevant |
| 1643 | | Bitar, S.A. et al., 2023 | | | Molecular mechanisms targeting drug-resistance and metastasis in colorectal cancer | No | - |
| 1644 | | Hong, Y.-M. et al., 2023 | | | Development and treatment of colorectal cancer: Insights from multi-kingdom microbiota | No | Irrelevant |
| 1645 | | Liu, Z. et al., 2023 | | | Multi-Omics Analysis Reveals Intratumor Microbes as Immunomodulators in Colorectal Cancer | No | Irrelevant |
| 1646 | | Rao Bommi, J. et al., 2023 | | | Recent Trends in Biosensing and Diagnostic Methods for Novel Cancer Biomarkers | No | Irrelevant |
| 1647 | | Kitsel, Y. et al., 2023 | | | Colorectal Cancer Liver Metastases: Genomics and Biomarkers with Focus on Local Therapies | No | - |
| 1648 | | Alsalloum, A. et al., 2023 | | | The Melanoma-Associated Antigen Family A (MAGE-A): A Promising Target for Cancer Immunotherapy? | No | Irrelevant |
| 1649 | | Stricker, E. et al., 2023 | | | HERVs and Cancer—A Comprehensive Review of the Relationship of Human Endogenous Retroviruses and Human Cancers | No | Irrelevant |
| 1650 | | Abe, Y. et al., 2023 | | | The Role of PRMT5 in Immuno-Oncology | No | Irrelevant |
| 1651 | | Lavacchi, D. et al., 2023 | | | Evaluation of Fruquintinib in the Continuum of Care of Patients with Colorectal Cancer | No | - |
| 1652 | | Plano, F. et al., 2023 | | | Monoclonal Gammopathies and the Bone Marrow Microenvironment | No | Irrelevant |
| 1653 | | Kato, I. et al., 2023 | | | Microbiome and Diet in Colon Cancer Development and Treatment | No | Irrelevant |
| 1654 | | Jlassi, A. et al., 2023 | | | VISTA+/CD8+ status correlates with favorable prognosis in Epithelial ovarian cancer | No | Irrelevant |
| 1655 | | Wang, C. et al., 2023 | | | N6-methyladenosine (m6A) as a regulator of carcinogenesis and drug resistance | No | Irrelevant |
| 1656 | | Hong, J.H. et al., 2023 | | | Lymphocyte activation gene (LAG)-3 as a potential immunotherapeutic target for microsatellite stable, PD-L1-positive endometrial cancer | No | Irrelevant |
| 1657 | | Miller, C.P. et al., 2023 | | | Immunohistochemical Detection of 5T4 in Renal Cell Carcinoma | No | Irrelevant |
| 1658 | | Kumar, R. et al., 2023 | | | Urachal carcinoma: The journey so far and the road ahead | No | Irrelevant |
| 1659 | | De Mattia, E. et al., 2023 | | | Predictive and Prognostic Value of Oncogene Mutations in Locally-Advanced Rectal Cancer | No | - |
| 1660 | | Simão, D.C. et al., 2023 | | | Bispecific T-Cell Engagers Therapies in Solid Tumors: Focusing on Prostate Cancer | No | Irrelevant |
| 1661 | | Ruff, S.M. et al., 2023 | | | A Review of Translational Research for Targeted Therapy for Metastatic Colorectal Cancer | No | - |
| 1662 | | Speiser, D.E. et al., 2023 | | | CD4+ T cells in cancer | No | Irrelevant |
| 1663 | | Krämer, A. et al., 2023 | | | Cancer of unknown primary: ESMO Clinical Practice Guideline for diagnosis, treatment and follow-up | No | Irrelevant |
| 1664 | | Zhang, J. et al., 2023 | | | FHL1 as a novel prognostic biomarker in lung adenocarcinoma | No | Irrelevant |
| 1665 | | Formslag, C.R. et al., 2023 | | | The past, present, and future of immunotherapy for colorectal cancer | No | - |
| 1666 | | Shi, Y. et al., 2023 | | | Cancer-associated fibroblasts-derived exosomes from chemoresistant patients regulate cisplatin resistance | No | Irrelevant |
| 1667 | | Cosso, F. et al., 2023 | | | Long-term response of more than 9 years to regorafenib in a heavily pretreated patient with metastatic colorectal cancer | No | Irrelevant |
| 1668 | | Dai, X. et al., 2023 | | | Cold atmospheric plasma: Novel opportunities for tumor microenvironment targeting | No | Irrelevant |
| 1669 | | Ji, Y. et al., 2023 | | | Quantitative systems pharmacology model of GITR-mediated T cell dynamics in tumor microenvironment | No | Irrelevant |
| 1670 | | Krug, J. et al., 2023 | | | N-glycosylation Regulates Intrinsic IFN-γ Resistance in Colorectal Cancer | No | Irrelevant |
| 1671 | | Tufail, M. et al., 2023 | | | Wnt3a is a promising target in colorectal cancer | No | Irrelevant |
| 1672 | | Shi, Y. et al., 2023 | | | PD-L1 immunohistochemistry assay optimization in classic Hodgkin lymphoma | No | Irrelevant |
| 1673 | | Charles, A. et al., 2023 | | | The Influence of the microbiome on the innate immune microenvironment of solid tumors | No | Irrelevant |
| 1674 | | Hosseini, K. et al., 2023 | | | The role of circadian gene timeless in gastrointestinal cancers | No | Irrelevant |
| 1675 | | Bhamidipati, D. et al., 2023 | | | Impact of tissue-agnostic approvals for gastrointestinal malignancies | No | Irrelevant |
| 1676 | | Park, S. et al., 2023 | | | Transcriptional upregulation of CXCL13 in immune checkpoint inhibitors in lung adenocarcinoma | No | Irrelevant |
| 1677 | | Sailer, V. et al., 2023 | | | Experimental in vitro, ex vivo and in vivo models in prostate cancer research | No | Irrelevant |
| 1678 | | Li, K. et al., 2023 | | | Genomic alteration profile and PD-L1 expression in breast cancer | No | Irrelevant |
| 1679 | | Wen, X. et al., 2023 | | | The crosstalk between intestinal bacterial microbiota and immune cells in colorectal cancer | No | Irrelevant |
| 1680 | | Sufian, M.A. et al., 2023 | | | Lipid-based nucleic acid therapeutics with in vivo efficacy | No | Irrelevant |
| 1681 | | Srivastava, V. et al., 2023 | | | Locally Advanced Rectal Cancer: What We Learned in the Last Two Decades | No | - |
| 1682 | | Faleck, D. et al., 2023 | | | Colitis-associated cancers | No | Irrelevant |
| 1683 | | Mix, S. et al., 2023 | | | Cavrotolimod, a Nanoparticle Toll-like Receptor 9 Agonist in Skin Cancer | No | Irrelevant |
| 1684 | | Cass, S. et al., 2023 | | | The Influence of the Microbiome on Metastatic Colorectal Cancer | No | Irrelevant |
| 1685 | | Kang, M.K., 2023 | | | Implications of recent neoadjuvant clinical trials on radiotherapy in rectal cancer | No | - |
| 1686 | | Tang, Y.-L. et al., 2023 | | | Resistance to targeted therapy in metastatic colorectal cancer | No | - |
| 1687 | | Zhou, Y. et al., 2023 | | | Intestinal toxicity to CTLA-4 blockade | No | Irrelevant |
| 1688 | | Liu, Q. et al., 2023 | | | Cardiovascular toxicity of tyrosine kinase inhibitors | No | Irrelevant |
| 1689 | | Torchiaro, E. et al., 2023 | | | Preclinical efficacy of NEDD8 and proteasome inhibitors in colorectal cancer | No | Irrelevant |
| 1690 | | Ros, J. et al., 2023 | | | Advances in immune checkpoint inhibitor combination strategies for microsatellite stable colorectal cancer | No | - |
| 1691 | | Yu, Z. et al., 2023 | | | The role of potential probiotic strains Lactobacillus reuteri in various intestinal diseases | No | Irrelevant |
| 1692 | | [No Authors Found], 2023 | | | Chinese guidelines for the diagnosis and comprehensive treatment of colorectal liver metastases (2023) | No | Irrelevant |
| 1693 | | Nair, M.G. et al., 2023 | | | Estimation of ALU Repetitive Elements in Plasma as a Cost-Effective Liquid Biopsy Tool for Disease Prognosis in Breast Cancer | No | Irrelevant |
| 1694 | | Santoro, A. et al., 2023 | | | Recent Advances in Cervical Cancer Management | No | Irrelevant |
| 1695 | | Kandasamy, G. et al., 2023 | | | Emerging Trends in Nano-Driven Immunotherapy for Treatment of Cancer | No | Irrelevant |
| 1696 | | Varier, L. et al., 2023 | | | An Overview of Ovarian Cancer: The Role of Cancer Stem Cells in Chemoresistance | No | Irrelevant |
| 1697 | | Habanjar, O. et al., 2023 | | | Crosstalk of Inflammatory Cytokines within the Breast Tumor Microenvironment | No | Irrelevant |
| 1698 | | Irmer, B. et al., 2023 | | | Extracellular Vesicles in Liquid Biopsies as Biomarkers for Solid Tumors | No | Irrelevant |
| 1699 | | Mulet-Margalef, N. et al., 2023 | | | Challenges and Therapeutic Opportunities in the dMMR/MSI-H Colorectal Cancer Landscape | No | - |
| 1700 | | Xu, G. et al., 2023 | | | CEMIP, acting as a scaffold protein for bridging GRAF1 and MIB1, promotes colorectal cancer metastasis | No | Irrelevant |
| 1701 | | Cunningham, C. et al., 2023 | | | Recombinant Endostatin as a Potential Radiosensitizer in NSCLC | No | Irrelevant |
| 1702 | | Dutta, S. et al., 2023 | | | Targets of Immune Escape Mechanisms in Cancer | No | Irrelevant |
| 1703 | | Li, D. et al., 2023 | | | Establishment of a Diverse Head and Neck Cancer Cell Bank | No | Irrelevant |
| 1704 | | Tang, G. et al., 2023 | | | The Dilemma of HSV-1 Oncolytic Virus Delivery | No | Irrelevant |
| 1705 | | Ross, T.J. et al., 2023 | | | The Microbiome-TIME Axis | No | Irrelevant |
| 1706 | | Zalevskaja, K. et al., 2023 | | | Characteristics of Pancreatic and Biliary Tract Cancers in Lynch Syndrome | No | Irrelevant |
| 1707 | | Abu-Rustum, N. et al., 2023 | | | Uterine Neoplasms, Version 1.2023 | No | Irrelevant |
| 1708 | | Floerchinger, A. et al., 2023 | | | A Vector-Encoded Bispecific Killer Engager | No | Irrelevant |
| 1709 | | Lee, E.-J. et al., 2023 | | | Antitumor Effect of Korean Red Ginseng | No | Irrelevant |
| 1710 | | Rebuzzi, F. et al., 2023 | | | Genetic Predisposition to Colorectal Cancer | No | - |
| 1711 | | Piao, X.-M. et al., 2023 | | | The Human Microbiome and RCC Outcomes | No | Irrelevant |
| 1712 | | Fanotto, V. et al., 2023 | | | Primary Tumor Resection in Metastatic GI Cancers | No | Irrelevant |
| 1713 | | Reitsam, N.G. et al., 2023 | | | NK Cells in Colorectal Cancer Patients | No | Irrelevant |
| 1714 | | San-Román-Gil, M. et al., 2023 | | | Immune Checkpoint Inhibitors in MSS Metastatic CRC | No | - |
| 1715 | | Spoerl, S. et al., 2023 | | | A20 as a Tool in Predicting Oral SCC Recurrence | No | Irrelevant |
| 1716 | | Pandey, H. et al., 2023 | | | Gut Microbiota in Colorectal Cancer | No | - |
| 1717 | | Chow, R.D. et al., 2023 | | | Mismatch-Repair Deficiency and Anti–PD-1 Response | No | Irrelevant |
| 1718 | | Zhang, R. et al., 2023 | | | Sohlh2 and Colon Cancer Stem Cells | No | Irrelevant |
| 1719 | | Mezheyeuski, A. et al., 2023 | | | Immune Score and Prognostic Impact in Solid Cancers | No | Irrelevant |
| 1720 | | Mo, S. et al., 2023 | | | B7 Family Immune Checkpoints in Pancreatic Tumors | No | Irrelevant |
| 1721 | | Xu, M. et al., 2023 | | | Anti-VEGF and Anti-EGFR in Metastatic CRC | No | Irrelevant |
| 1722 | | Merhi, M. et al., 2023 | | | Transcription Factors, Immune Checkpoints, and CRC | No | Irrelevant |
| 1723 | | Chrysostomou, D. et al., 2023 | | | Gut Microbiota and Cancer Therapy Response | No | Irrelevant |
| 1724 | | Zhang, S.-L. et al., 2023 | | | Gut Microbiota and Tumor Immunologic Status in CRC | No | Irrelevant |
| 1725 | | Nusrat, M. et al., 2023 | | | KRAS Inhibition in Metastatic CRC | No | - |
| 1726 | | Chen, Y. et al., 2023 | | | Wnt/β-catenin Pathway in CRC Therapy | No | Irrelevant |
| 1727 | | Shi, L. et al., 2023 | | | Gut Microbiome and Immune Checkpoints in CRC | No | - |
| 1728 | | Di Dio, C. et al., 2023 | | | Immunotherapy in Endometrial Carcinoma | No | Irrelevant |
| 1729 | | Ma, R. et al., 2023 | | | Oncolytic Virus-Based Cancer Immunotherapy | No | Irrelevant |
| 1730 | | Zwergel, C. et al., 2023 | | | PD-L1 Small-Molecule Modulators | No | Irrelevant |
| 1731 | | Hassin, O. et al., 2023 | | | Drugging p53 in Cancer | No | Irrelevant |
| 1732 | | Wei, L. et al., 2023 | | | Immune Gene-Based Risk Model in Colon Adenocarcinoma | No | Irrelevant |
| 1733 | | Ni, J.-J. et al., 2023 | | | Immunotherapy for Cold Tumors | No | Irrelevant |
| 1734 | | Businello, G. et al., 2023 | | | Biomarkers in Gastric/Gastroesophageal Adenocarcinoma | No | Irrelevant |
| 1735 | | Wolf, N.K. et al., 2023 | | | NK Cells in Cancer Immunity and Therapy | No | Irrelevant |
| 1736 | | Guo, M. et al., 2023 | | | Notch Signaling, Hypoxia, and Cancer | No | Irrelevant |
| 1737 | | Zeng, Z. et al., 2023 | | | Splenic Volume as a Prognostic Biomarker in Gastric Cancer | No | Irrelevant |
| 1738 | | Chen, J. et al., 2023 | | | STK11 and SMARCB1 Deficiency in Lung Cancer | No | Irrelevant |
| 1739 | | Lazzari, C. et al., 2023 | | | Mepolizumab for ICI-Induced Hypereosinophilic Syndrome | No | Irrelevant |
| 1740 | | de Vries, N.L. et al., 2023 | | | γδ T Cells in Immunotherapy for HLA-Class I Deficient Cancers | No | Irrelevant |
| 1741 | | Bilal, M. et al., 2023 | | | Role of AI and Digital Pathology for Colorectal Immuno-Oncology | No | Irrelevant |
| 1742 | | Ağagündüz, D. et al., 2023 | | | Gut Microbiome in GI Cancer | No | Irrelevant |
| 1743 | | Xu, J., 2023 | | | Chinese Guidelines for Colorectal Liver Metastases | No | Irrelevant |
| 1744 | | von den Driesch, J. et al., 2023 | | | Medullary Pancreatic Cancer Characterization | No | Irrelevant |
| 1745 | | Zhang, H. et al., 2023 | | | Lymph Node Metastasis-Related Genes in Colon Adenocarcinoma | No | Irrelevant |
| 1746 | | Cao, Y. et al., 2023 | | | Exosomes in Cancer Immunotherapy | No | Irrelevant |
| 1747 | | Huang, H. et al., 2023 | | | DNA Methylation in CRC Patients | No | Irrelevant |
| 1748 | | Stravokefalou, V. et al., 2023 | | | IL-15 and Chemotherapy in TNBC | No | Irrelevant |
| 1749 | | Wen, K. et al., 2023 | | | Ferroptosis and Immune Response in HCC | No | Irrelevant |
| 1750 | | Lemech, C. et al., 2023 | | | Pixatimod and Nivolumab in Solid Tumors | No | Irrelevant |
| 1751 | | Zhang, Q. et al., 2023 | | | Tertiary Lymphoid Structures in Cancer | No | Irrelevant |
| 1752 | | Lin, Y. et al., 2023 | | | CRISPR Cas9 for Immune Checkpoint Disruption | No | Irrelevant |
| 1753 | | Laderach, D.J. et al., 2023 | | | Galectin Inhibition in Cancer | No | Irrelevant |
| 1754 | | Hua, H. et al., 2023 | | | MSI-H CRC Patients and Immunotherapy | No | Irrelevant |
| 1755 | | Marritt, K.L. et al., 2023 | | | STING Activation in Soft Tissue Sarcoma | No | Irrelevant |
| 1756 | | Wang, E. et al., 2023 | | | RNA Splicing and BCL2 Inhibition in Leukemia | No | Irrelevant |
| 1757 | | Mirza, S. et al., 2023 | | | Liquid Biopsy and Immunotherapy in CRC | No | Irrelevant |
| 1758 | | Ghazvinian, Z. et al., 2023 | | | NK Cells in CRC Immunity | No | Irrelevant |
| 1759 | | Chen, K.-S. et al., 2023 | | | Bifunctional Cancer Cell-Based Vaccine | No | Irrelevant |
| 1760 | | Bansal, M.P., 2023 | | | Redox Regulation in Cancer | No | Irrelevant |
| 1761 | | Berzofsky, J.A. et al., 2023 | | | Cancer Vaccines | No | Irrelevant |
| 1762 | | Chun, H.J. et al., 2023 | | | GI Cancer: Diagnosis and Management | No | Irrelevant |
| 1763 | | Tao, K. et al., 2023 | | | Pediatric Precision Medicine in Japan | No | Irrelevant |
| 1764 | | Li, H. et al., 2023 | | | Medicine in CRC | No | Irrelevant |
| 1765 | | Gatius, S. et al., 2023 | | | Molecular Pathology of Endometrial Tumors | No | Irrelevant |
| 1766 | | Saw, P.E. et al., 2023 | | | Host Adaptive Immune System in Cancer | No | Irrelevant |
| 1767 | | Singh, T. et al., 2023 | | | PD-L1, Tumor Budding, and TILs in CRC | No | Irrelevant |
| 1768 | | Booth, M. et al., 2023 | | | Non-Surgical Cancer Treatments | No | Irrelevant |
| 1769 | | Ramos, K.S. et al., 2023 | | | Genetic and Epigenetic Dysregulation in Disease | No | Irrelevant |
| 1770 | | Yakushina, V. et al., 2023 | | | MSI Detection Standards and Challenges | No | Irrelevant |
| 1771 | | Filoni, E. et al., 2023 | | | Multimodal CRC Liver Metastases Management | No | Irrelevant |
| 1772 | | Yarahmadi, A. et al., 2023 | | | Microbiomes in GI Cancers | No | Irrelevant |
| 1773 | | Yang, M. et al., 2023 | | | Target and Immunotherapy in Lung Adenocarcinoma | No | Irrelevant |
| 1774 | | Hu, J. et al., 2023 | | | ML-Based Prediction in Rectal Cancer | No | Irrelevant |
| 1775 | | Nissen, N.I. et al., 2023 | | | Collagens and Fibroblasts in Cancer | No | Irrelevant |
| 1776 | | Grindel, A.-L. et al., 2023 | | | TLR-9 Agonist and PD-1 in CRC | No | Irrelevant |
| 1777 | | Tsuboi, J. et al., 2023 | | | Biomarkers in Pancreatic Cancer | No | Irrelevant |
| 1778 | | Mishra, M. et al., 2023 | | | Evolution in Cancer Diagnosis | No | Irrelevant |
| 1779 | | Lin, P. et al., 2023 | | | Drug Combination for RAS-Mediated CRC | No | Irrelevant |
| 1780 | | Fatemi, N. et al., 2023 | | | Drug Repositioning in GI Oncology | No | Irrelevant |
| 1781 | | Li, F. et al., 2023 | | | Immunochemotherapy in NSCLC | No | Irrelevant |
| 1782 | | Antea, K. et al., 2023 | | | Future of Cancer Immunotherapy | No | Irrelevant |
| 1783 | | Dong, C. et al., 2023 | | | lncRNA and Prognosis in CRC | No | Irrelevant |
| 1784 | | Khan, S. et al., 2023 | | | Metagenomics in Pancreatic Cancer | No | Irrelevant |
| 1785 | | Ding, K. et al., 2023 | | | MSI-Stable CRC Immunotherapy | No | Irrelevant |
| 1786 | | Procaccianti, G. et al., 2023 | | | Bifidobacterium in Cancer Therapy | No | Irrelevant |
| 1787 | | Longva, A.S. et al., 2023 | | | VEGF and Immunogenic Cell Death | No | Irrelevant |
| 1788 | | Liu, J. et al., 2023 | | | Microbial Metabolites in Tumorigenesis | No | Irrelevant |
| 1789 | | Chen, J. et al., 2023 | | | MYC and Mitochondrial DNA in Prostate Cancer | No | Irrelevant |
| 1790 | | Xu, C. et al., 2023 | | | PCD-Related Prognostic Signature in HNSCC | No | Irrelevant |
| 1791 | | Pourali, G. et al., 2023 | | | Targeting TGF-β and PD-L1 in Pancreatic Cancer | No | Irrelevant |
| 1792 | | Li, X. et al., 2023 | | | miR-455-5p and Breast Cancer Progression | No | Irrelevant |
| 1793 | | Griffith, B.D. et al., 2023 | | | Tumor Immune Microenvironment in Young CRC Patients | No | Irrelevant |
| 1794 | | Hackett, J.B. et al., 2023 | | | IFN-γ ImmunoPET Imaging for ICI Response | No | Irrelevant |
| 1795 | | Pérez Jorge, G. et al., 2023 | | | Salmonella and Outer Membrane Vesicles in Cancer | No | Irrelevant |
| 1796 | | You, X. et al., 2023 | | | Heterogeneity of TLS in Cancer | No | Irrelevant |
| 1797 | | Zhang, J. et al., 2023 | | | Macrophages in Gastric Cancer | No | Irrelevant |
| 1798 | | Ke, P. et al., 2023 | | | Venetoclax-Resistance Prognostic Signature in AML | No | Irrelevant |
| 1799 | | Maurya, N.S. et al., 2023 | | | Drug Updates in CRC Treatment | No | Irrelevant |
| 1800 | | Nersesian, S. et al., 2023 | | | NK Cells in Cancer Immunotherapy | No | Irrelevant |
| 1801 | | Amalinei, C. et al., 2023 | | | Colorectal Cancer Stem Cells | No | Irrelevant |
| 1802 | | Kerr, S.E. et al., 2023 | | | Molecular Testing in Gynecologic Cancer | No | Irrelevant |
| 1803 | | Piroozkhah, M. et al., 2023 | | | Tumor-Infiltrating Lymphocytes in GI Cancer | No | Irrelevant |
| 1804 | | Sarsembayeva, A. et al., 2023 | | | Cannabinoids in Immunotherapy | No | Irrelevant |
| 1805 | | Götz, L. et al., 2023 | | | Carcinoembryonic Antigen-Related Molecule in Cancer | No | Irrelevant |
| 1806 | | Amajala, K.C. et al., 2023 | | | Gamma Delta T Cells in HCC Immunotherapy | No | Irrelevant |
| 1807 | | Mardis, E.R., 2023 | | | Pediatric Cancer Immunotherapy | No | Irrelevant |
| 1808 | | Lyon, R.P. et al., 2023 | | | Vedotin ADC for Carcinoma | No | Irrelevant |
| 1809 | | Olivera, I. et al., 2023 | | | Inflammatory Mediators in Cancer | No | Irrelevant |
| 1810 | | Van Dingenen, L. et al., 2023 | | | Gut Microbiome in CRC Immunotherapy | No | Irrelevant |
| 1811 | | Sanz-Garcia, E. et al., 2023 | | | ctDNA as Biomarker in Immunotherapy | No | Irrelevant |
| 1812 | | Shao, S. et al., 2023 | | | Tumor-Associated Macrophages | No | Irrelevant |
| 1813 | | Yang, J. et al., 2023 | | | Comprehensive Genomic Profiling in CRC | No | Irrelevant |
| 1814 | | Pang, S.-W. et al., 2023 | | | Molecular Diagnostics in CRC | No | Irrelevant |
| 1815 | | Sang, J. et al., 2023 | | | Immune Biomarkers in Lung Cancer | No | Irrelevant |
| 1816 | | Schlicher, L. et al., 2023 | | | Small Molecule Inhibitors in Immunotherapy | No | Irrelevant |
| 1817 | | Nguyen, H.-Q. et al., 2023 | | | CRC Heterogeneity and Targeted Therapy | No | Irrelevant |
| 1818 | | Young, S.E. et al., 2023 | | | Genomic Alterations in Cholangiocarcinoma | No | Irrelevant |
| 1819 | | Hoshimoto, A. et al., 2023 | | | PD-L1 in Small Bowel Adenocarcinoma | No | Irrelevant |
| 1820 | | Chakraborty, S. et al., 2023 | | | TLRs in Cancer Vaccines | No | Irrelevant |
| 1821 | | Skvortsov, A.V. et al., 2023 | | | Bevacizumab-Induced Thrombotic Microangiopathy | No | Irrelevant |
| 1822 | | Yao, S. et al., 2023 | | | Induced-Chemotherapy in CRC Immunotherapy | No | Irrelevant |
| 1823 | | Zhou, C. et al., 2023 | | | Lung Cancer and COPD Consensus | No | Irrelevant |
| 1824 | | Kingsak, M. et al., 2023 | | | Oncolytic Viruses in Precision Oncology | No | Irrelevant |
| 1825 | | Penkova, A. et al., 2023 | | | Molecular Diagnostics of Gliomas | No | Irrelevant |
| 1826 | | Zhang, H. et al., 2023 | | | MSI in Prostate Cancer | No | Irrelevant |
| 1827 | | Tsumura, A. et al., 2023 | | | Checkpoint Inhibition in Hematologic Malignancies | No | Irrelevant |
| 1828 | | Cornista, A.M. et al., 2023 | | | CRC Immunotherapy State of the Art | No | Irrelevant |
| 1829 | | Wu, X. et al., 2023 | | | SCC of the Colon: Case Report | No | Irrelevant |
| 1830 | | Yu, A. et al., 2023 | | | TLS in GI Cancers | No | Irrelevant |
| 1831 | | Bao, J. et al., 2023 | | | B Cells in HNSCC | No | Irrelevant |
| 1832 | | Zhong, L. et al., 2023 | | | TP53 Mutations in Esophageal SCC | No | Irrelevant |
| 1833 | | Wang, M. et al., 2023 | | | Intratumor Microbiota in Cancer | No | Irrelevant |
| 1834 | | Zhan, W. et al., 2023 | | | Siglec15 in CRC Prognosis | No | Irrelevant |
| 1835 | | Patel, R.K. et al., 2023 | | | CRC Liver Metastases Management | No | Irrelevant |
| 1836 | | Zhang, Q. et al., 2023 | | | EBV-Specific T-Cell Responses in Cancer | No | Irrelevant |
| 1837 | | Maldonado, H. et al., 2023 | | | CSK Signaling by Integrins in Cancer | No | Irrelevant |
| 1838 | | Huang, J. et al., 2023 | | | Gut Microbiota in ICI Therapy | No | Irrelevant |
| 1839 | | Cao, Y. et al., 2023 | | | TCR Abnormalities in CRC | No | Irrelevant |
| 1840 | | Liu, J. et al., 2023 | | | Glycosylation Risk Score in Bladder Cancer | No | Irrelevant |
| 1841 | | Staudt, S. et al., 2023 | | | Microbiome and T Cell Immunotherapy | No | Irrelevant |
| 1842 | | Das, S. et al., 2023 | | | Immunotherapy in CRC | No | Irrelevant |
| 1843 | | Li, Y. et al., 2023 | | | Tumor Heterogeneity in Uveal Melanoma | No | Irrelevant |
| 1844 | | Mukherjee, S. et al., 2023 | | | TLR-Guided Therapy in Cancer | No | Irrelevant |
| 1845 | | Shen, Q. et al., 2023 | | | NDC1 as Prognostic Biomarker in Pancreatic Cancer | No | Irrelevant |
| 1846 | | Abdellateif, M.S. et al., 2023 | | | c-Kit as a Cancer Target | No | Irrelevant |
| 1847 | | Abdallah, D.I. et al., 2023 | | | HDAC Inhibitors in Cancer | No | Irrelevant |
| 1848 | | Arezzo, F. et al., 2023 | | | Metastatic Endometrial Cancer Therapy | No | Irrelevant |
| 1849 | | He, X. et al., 2023 | | | Immunotherapy and Chemotherapy in CRC | No | Irrelevant |
| 1850 | | Huang, Y.L. et al., 2023 | | | Organotypic Models in Cancer | No | Irrelevant |
| 1851 | | Song, Y. et al., 2023 | | | Organoids and Metastatic Orthotopic Mouse Model in CRC | No | Irrelevant |
| 1852 | | Bugoye, F.C. et al., 2023 | | | Mutational Spectrum of DNA Damage in Prostate Cancer | No | Irrelevant |
| 1853 | | Dhar, A.K., 2023 | | | Microbiome Modulation in Pancreatic Cancer | No | Irrelevant |
| 1854 | | Alfahed, A., 2023 | | | Molecular Pathology of CRC in Saudi Arabia | No | Irrelevant |
| 1855 | | Deng, T. et al., 2023 | | | Third-Line Treatment in Metastatic CRC | No | Irrelevant |
| 1856 | | Marinkovic, M. et al., 2023 | | | Genetic and Hematological Predictors in Rectal Cancer | No | Irrelevant |
| 1857 | | Jiang, H. et al., 2023 | | | Early Diagnosis Biomarkers and TKIs in CRC | No | Irrelevant |
| 1858 | | Deng, S.-Z. et al., 2023 | | | Lysine Acetylation in OSCC | No | Irrelevant |
| 1859 | | Huang, Y. et al., 2023 | | | Peptides in TNBC Targeted Therapy | No | Irrelevant |
| 1860 | | Goh, K.W. et al., 2023 | | | Aptamers in GI Cancers | No | Irrelevant |
| 1861 | | Diong, S.J.J. et al., 2023 | | | PVR Family and TIGIT Antibody Recognition | No | Irrelevant |
| 1862 | | Kamali, A.N. et al., 2023 | | | Immune Checkpoints and New Receptors in Cancer | No | Irrelevant |
| 1863 | | Pandiella, A. et al., 2023 | | | Development of Immuno-Oncology Agents | No | Irrelevant |
| 1864 | | Esparcia-Pinedo, L. et al., 2023 | | | TLS and B Lymphocytes in Cancer Therapy | No | Irrelevant |
| 1865 | | Fiat, F. et al., 2023 | | | Cytotoxicity of Lidocaine in Melanoma | No | Irrelevant |
| 1866 | | Li, Y. et al., 2023 | | | SCFAs in Cancer from Microbial Fermentation | No | Irrelevant |
| 1867 | | Zheng, J. et al., 2023 | | | ICIs + Chemotherapy in Small Cell Lung Cancer | No | Irrelevant |
| 1868 | | Mahé, M. et al., 2023 | | | Genetics of Enzymatic Dysfunctions in Cancer | No | Irrelevant |
| 1869 | | Nelson, V.K. et al., 2023 | | | ROS and Alkaloids in Colon Cancer | No | Irrelevant |
| 1870 | | Benek, S. et al., 2023 | | | CD274 (PD-L1) and CD3+ Lymphocytes in CRC | No | Irrelevant |
| 1871 | | Chen, H.-H. et al., 2023 | | | Gut Microbiome and SCFAs in Lung Cancer Therapy | No | Irrelevant |
| 1872 | | Li, Y. et al., 2023 | | | Whole-Exome Sequencing in Rectal NETs | No | Irrelevant |
| 1873 | | Chamanza, R. et al., 2023 | | | Hematolymphoid System in Non-Human Primates | No | Irrelevant |
| 1874 | | Cabioglu, N. et al., 2023 | | | Prognostic Effects of CD155 and CD73 in TNBC | No | Irrelevant |
| 1875 | | Han, N. et al., 2023 | | | IL-34 and Therapy Resistance | No | Irrelevant |
| 1876 | | Wang, X. et al., 2023 | | | Active Ingredients from Chinese Medicine in Cancer | No | Irrelevant |
| 1877 | | Qu, F. et al., 2023 | | | Radiotherapy in Rectal SCC with PD-L1 Expression | No | Irrelevant |
| 1878 | | Gu, C. et al., 2023 | | | Prognostic Model of Quercetin and Kaempferol in CRC | No | Irrelevant |
| 1879 | | Huang, X. et al., 2023 | | | PI3K and PIKK Inhibitors | No | Irrelevant |
| 1880 | | Jiang, P.-C. et al., 2023 | | | Cuproptosis and Hypoxia in CRC and Pan-Cancer | No | Irrelevant |
| 1881 | | Voutsadakis, I.A., 2023 | | | High TMB in MSS CRC | No | Irrelevant |
| 1882 | | Liu, X. et al., 2023 | | | Mast Cells in CRC Progression | No | Irrelevant |
| 1883 | | Wang, Z. et al., 2023 | | | CRC and Gut Microbiota Studies in China | No | Irrelevant |
| 1884 | | Ros, J. et al., 2023 | | | BRAF-V600E Mutant CRC Treatment | No | Irrelevant |
| 1885 | | Mestrallet, G. et al., 2023 | | | Immune Escape in MMR Deficient Tumors | No | Irrelevant |
| 1886 | | Savli, T.B. et al., 2023 | | | CTLA-4, CD44, and E-cadherin in Breast Cancer | No | Irrelevant |
| 1887 | | Sousa, S.M. et al., 2023 | | | Repurposing NSAIDs in Cancer | No | Irrelevant |
| 1888 | | Wang, Y.A. et al., 2023 | | | NK Cell-Targeted Immunotherapies in Bladder Cancer | No | Irrelevant |
| 1889 | | Vella, V. et al., 2023 | | | Insulin/IGF Axis in Cancer Therapy | No | Irrelevant |
| 1890 | | Deng, H. et al., 2023 | | | Macrophage "Do Not Eat Me" Signaling in Cancer | No | Irrelevant |
| 1891 | | Li, C. et al., 2023 | | | Immune-Related Prognostic Index in CRC | No | Irrelevant |
| 1892 | | Kashyap, M.K. et al., 2023 | | | Signal Transduction in CRC Carcinogenesis | No | Irrelevant |
| 1893 | | Chalova, P. et al., 2023 | | | SCFA as Biomarkers in Cancer | No | Irrelevant |
| 1894 | | Czajka-Francuz, P. et al., 2023 | | | Tumor Microenvironment and Targeted Therapy | No | Irrelevant |
| 1895 | | Badve, S.S. et al., 2023 | | | Tumor-TME Crosstalk in Cancer Therapy | No | Irrelevant |
| 1896 | | Cann, C.G. et al., 2023 | | | Genetic Targets in Metastatic CRC | No | Irrelevant |
| 1897 | | Tang, Y. et al., 2023 | | | Ubiquitination Genes in Hepatocellular Carcinoma | No | Irrelevant |
| 1898 | | Zheng, M. et al., 2023 | | | VISTA in Allergy, Autoimmunity, and Transplant Rejection | No | Irrelevant |
| 1899 | | Bösherz, M.-S. et al., 2023 | | | IHC Scoring in Urothelial Carcinoma | No | Irrelevant |
| 1900 | | Christenson, E.S. et al., 2023 | | | BRAF p.V600E and MMR Deficiency in Elderly CRC Patients | No | Irrelevant |
| 1901 | | Bergamaschi et al., 2023 | | | Management and pharmacotherapy of pediatric colorectal carcinoma: a review | No | Irrelevant |
| 1902 | | Zhu et al., 2023 | | | Development and application of oncolytic viruses as the nemesis of tumor cells | No | Irrelevant |
| 1903 | | Grant et al., 2023 | | | Endometrial Adenocarcinoma with Discordant Microsatellite Stability Status Treated with First-Line Pembrolizumab: A Case Report and Narrative Review | No | Irrelevant |
| 1904 | | Rojas et al., 2023 | | | Vaccines and active immunization against cancer | No | Irrelevant |
| 1905 | | Siddique et al., 2023 | | | Pharmacogenetics of Anticancer Drugs: Clinical Response and Toxicity | No | Irrelevant |
| 1906 | | Dasanu et al., 2023 | | | Immune checkpoint inhibition in advanced colorectal cancer with inherited and acquired microsatellite instability: Current state and future directions | No | - |
| 1907 | | Zheng et al., 2023 | | | Progress and prospects of targeted therapy and immunotherapy for urachal carcinoma | No | Irrelevant |
| 1908 | | Constance et al., 2023 | | | Mapping the Evidence for Opioid-Mediated Changes in Malignancy and Chemotherapeutic Efficacy: Protocol for a Scoping Review | No | Irrelevant |
| 1909 | | Shen et al., 2023 | | | HDAC inhibitors enhance the anti-tumor effect of immunotherapies in hepatocellular carcinoma | No | Irrelevant |
| 1910 | | Syed et al., 2023 | | | Precisive Colon Cancer Treatment: Exploring Novel Avenues of Cancer Treatment Through Multistage Nanocarriers | No | Irrelevant |
| 1911 | | Lin et al., 2023 | | | The Role of RNA Methylation Modification Related Genes in Prognosis and Immunotherapy of Colorectal Cancer | No | Irrelevant |
| 1912 | | Liu et al., 2023 | | | Genomic analysis of immunogenic cell death-related subtypes for predicting prognosis and immunotherapy outcomes in glioblastoma multiforme | No | Irrelevant |
| 1913 | | Mani et al., 2023 | | | Modulation of T cell function and survival by the tumor microenvironment | No | Irrelevant |
| 1914 | | De Sanctis et al., 2023 | | | Molecular Mechanisms of Chloroquine and Hydroxychloroquine used in Cancer Therapy | No | Irrelevant |
| 1915 | | Fan et al., 2023 | | | Longitudinal change of circulating tumor cell level and its relationship with immune checkpoint inhibitor-based treatment benefits in unresectable, metastatic colorectal cancer patients | No | - |
| 1916 | | Zeng et al., 2023 | | | Mediating immunosuppressive functions: a new perspective on the complex immunological properties of SEMA4D in the tumor microenvironment | No | Irrelevant |
| 1917 | | Bruni et al., 2023 | | | Cancer immune exclusion: breaking the barricade for a successful immunotherapy | No | Irrelevant |
| 1918 | | Han et al., 2023 | | | Dysregulation in IFN-γ signaling and response: the barricade to tumor immunotherapy | No | Irrelevant |
| 1919 | | Almagro et al., 2023 | | | Volume imaging to interrogate cancer cell-tumor microenvironment interactions in space and time | No | Irrelevant |
| 1920 | | Raza et al., 2023 | | | Serum immune mediators as novel predictors of response to anti-PD-1/PD-L1 therapy in non-small cell lung cancer patients with high tissue-PD-L1 expression | No | Irrelevant |
| 1921 | | Kouidhi et al., 2023 | | | Gut microbiota, an emergent target to shape the efficiency of cancer therapy | No | Irrelevant |
| 1922 | | Atay et al., 2023 | | | Armored modified vaccinia Ankara in cancer immunotherapy | No | Irrelevant |
| 1923 | | D’Alpino Peixoto et al., 2023 | | | Unresectable metastatic colorectal cancer in fit patients – a practical algorithm of treatment sequencing from the Brazilian Group of Gastrointestinal Tumours (GTG) | No | - |
| 1924 | | Zhong et al., 2023 | | | Impact of Molecular Status on Cytoreductive Surgery for Peritoneal Metastases from Colorectal Cancer | No | - |
| 1925 | | Zhai et al., 2023 | | | Fibroblast Growth Factor 11 Enables Tumor Cell Immune Escape by Promoting T Cell Exhaustion and Predicts Poor Prognosis in Patients with Lung Adenocarcinoma | No | Irrelevant |
| 1926 | | Avendaño et al., 2023 | | | Medicinal Chemistry of Anticancer Drugs, Third Edition | No | Irrelevant |
| 1927 | | Dey et al., 2023 | | | Recent Advancements, Limitations, and Future Perspectives of the use of Personalized Medicine in Treatment of Colon Cancer | No | - |
| 1928 | | Plüss et al., 2023 | | | Generation and in vivo characterization of a novel high-affinity human antibody targeting carcinoembryonic antigen | No | Irrelevant |
| 1929 | | Shah et al., 2023 | | | A narrative review from gut to lungs: non-small cell lung cancer and the gastrointestinal microbiome | No | Irrelevant |
| 1930 | | Banday et al., 2023 | | | Immune Checkpoint Inhibitors: Recent Clinical Advances and Future Prospects | No | - |
| 1931 | | Laube et al., 2023 | | | Gut Microbiome and Immune Responses in Gastrointestinal Cancer | No | Irrelevant |
| 1932 | | Wong et al., 2023 | | | Targeting Gut Microbiota in Colorectal Cancer | No | Irrelevant |
| 1933 | | Cai et al., 2023 | | | Research progress of interleukin-15 in cancer immunotherapy | No | Irrelevant |
| 1934 | | Rezouki et al., 2023 | | | High VISTA expression is linked to a potent epithelial-mesenchymal transition and is positively correlated with PD1 in breast cancer | No | Irrelevant |
| 1935 | | Piringer et al., 2023 | | | Ongoing complete response after treatment cessation with dabrafenib, trametinib, and cetuximab as third-line treatment in a patient with advanced BRAFV600E mutated, MSS colon cancer | No | Irrelevant |
| 1936 | | Xu et al., 2023 | | | Identification of prognostic cancer-associated fibroblast markers in luminal breast cancer using weighted gene co-expression network analysis | No | Irrelevant |
| 1937 | | Zhang et al., 2023 | | | Case report: A combined immunotherapy strategy as a promising therapy for MSI-H colorectal carcinomas with multiple HPD risk factors | No | - |
| 1938 | | Wang et al., 2023 | | | Machine learning-based glycolysis-associated molecular classification reveals differences in prognosis, TME, and immunotherapy for colorectal cancer patients | No | - |
| 1939 | | Lin et al., 2023 | | | Immuno-oncology-microbiome axis of gastrointestinal malignancy | No | Irrelevant |
| 1940 | | Lei et al., 2023 | | | Acute inflammatory reaction during anti-angiogenesis therapy combined with immunotherapy as a possible indicator of the therapeutic effect: Three case reports and literature review | No | - |
| 1941 | | Shah et al., 2023 | | | A Review on Anticancer Profile of Flavonoids: Sources, Chemistry, Mechanisms, Structure-activity Relationship and Anticancer Activity | No | Irrelevant |
| 1942 | | Filippi et al., 2023 | | | Integrated use of 90Y-labeled microspheres and immune checkpoint inhibitors in hepatic tumors: current status and future directions | No | Irrelevant |
| 1943 | | Wu et al., 2023 | | | Identification of immune cell infiltration landscape for predicting prognosis of colorectal cancer | No | Irrelevant |
| 1944 | | Zheng et al., 2023 | | | Immune desert in MMR-deficient tumors predicts poor responsiveness of immune checkpoint inhibition | No | Irrelevant |
| 1945 | | Li et al., 2023 | | | Immune Checkpoint Inhibitor-Based Combination Therapy for Colorectal Cancer: An Overview | No | - |
| 1946 | | Nemtsova et al., 2023 | | | Targeting of the SWI/SNF chromatin remodeling complex in cancer therapy | No | Irrelevant |
| 1947 | | Ursino et al., 2023 | | | Intrinsic features of the cancer cell as drivers of immune checkpoint blockade response and refractoriness | No | Irrelevant |
| 1948 | | Lin et al., 2023 | | | Mutations Status of NOTCH Signaling Pathway Predict Prognosis of Immune Checkpoint Inhibitors in Colorectal Cancer | No | Irrelevant |
| 1949 | | Pan et al., 2023 | | | A novel prognostic signature based on metastasis- and immune-related gene pairs for colorectal cancer | No | Irrelevant |
| 1950 | | Dillman et al., 2023 | | | Autologous dendritic cells loaded with antigens from self-renewing autologous tumor cells as patient-specific therapeutic cancer vaccines | No | Irrelevant |
| 1951 | | Zheng et al., 2023 | | | Association between CD8+ Tumor-Infiltrating Lymphocytes and Prognosis of NSCLC Patients Treated with PD-1/PD-L1 Inhibitors | No | Irrelevant |
| 1952 | | Shields et al., 2023 | | | Late-stage MC38 Tumors Recapitulate Features of Human Colorectal Cancer | No | Irrelevant |
| 1953 | | Kim et al., 2023 | | | Clinical Implication of DNA Damage Response Genes in Advanced Gastric Cancer Stage IV and Recurrent Gastric Cancer | No | Irrelevant |
| 1954 | | Zhang et al., 2023 | | | Oxaliplatin Plus Irinotecan vs Irinotecan as Second-Line Treatment in Pancreatic Cancer | No | Irrelevant |
| 1955 | | Dong et al., 2023 | | | Gut Microbiota-Derived Short-Chain Fatty Acids Regulate Gastrointestinal Tumor Immunity | No | Irrelevant |
| 1956 | | Patel et al., 2023 | | | Multi-Targeted HDAC Inhibitors as Anticancer Agents | No | Irrelevant |
| 1957 | | Ji et al., 2023 | | | Metastatic Pattern as a Prognostic Factor in BRAFV600E Mutant Colorectal Cancer | No | Irrelevant |
| 1958 | | Brady et al., 2023 | | | Tumor-Associated Macrophages: Prognostic and Therapeutic Targets for Cancer in Humans and Dogs | No | Irrelevant |
| 1959 | | Nisar et al., 2023 | | | Proteomics Approaches in the Identification of Cancer Biomarkers and Drug Discovery | No | Irrelevant |
| 1960 | | Liu et al., 2023 | | | HZ-A-018, a Novel Inhibitor of Bruton Tyrosine Kinase, Exerts Anti-Cancer Activity | No | Irrelevant |
| 1961 | | Wang et al., 2023 | | | Diet-Gut Microbial Interactions Influence Cancer Immunotherapy | No | Irrelevant |
| 1962 | | Zhang et al., 2023 | | | CAFs Orchestrate Tumor Immune Microenvironment: A New Target in Cancer Therapy? | No | Irrelevant |
| 1963 | | Wang et al., 2023 | | | Cuproptosis-Based Molecular Subtypes and Immune Landscape in Colon Cancer | No | Irrelevant |
| 1964 | | Zhang et al., 2023 | | | Research Progress of Immunotherapy for Gastric Cancer | No | Irrelevant |
| 1965 | | Xiao et al., 2023 | | | Total Neoadjuvant Treatment and PD-1/PD-L1 Checkpoint Inhibitor in Locally Advanced Rectal Cancer | No | Irrelevant |
| 1966 | | Soltan et al., 2023 | | | CHD1L as a Prognostic and Immunological Biomarker in Several Human Cancers | No | Irrelevant |
| 1967 | | Lai et al., 2023 | | | Novel Prognostic Biomarkers and Therapeutic Targets for NSCLC | No | Irrelevant |
| 1968 | | Li et al., 2023 | | | Histone Acetylation-Related lncRNA: Potential Biomarkers for Prognosis and Immune Response in Lung Cancer | No | Irrelevant |
| 1969 | | Kaur et al., 2023 | | | Sulfur-Containing Heterocyclic Compounds as Anticancer Agents | No | Irrelevant |
| 1970 | | Jeong et al., 2023 | | | CD4+ Cytotoxic T Cells: An Emerging Effector Arm of Anti-Tumor Immunity | No | Irrelevant |
| 1971 | | Jeong et al., 2023 | | | Challenges to Addressing the Unmet Medical Needs for Immunotherapy Targeting Cold Colorectal Cancer | No | Irrelevant |
| 1972 | | Preet Kaur et al., 2023 | | | The Role of Dendritic Cells in Radiation-Induced Immune Responses | No | Irrelevant |
| 1973 | | Olguin et al., 2023 | | | Combination of Immunotherapy with Chemotherapy for Colorectal Cancer Treatment | No | Irrelevant |
| 1974 | | Liu et al., 2023 | | | "Cold" Colorectal Cancer Faces a Bottleneck in Immunotherapy | No | Irrelevant |
| 1975 | | McCarthy et al., 2023 | | | Tumor-Infiltrating Lymphocytes as an Endpoint in Cancer Vaccine Trials | No | Irrelevant |
| 1976 | | Yeh et al., 2023 | | | Identifying and Clinically Validating Biomarkers for Immunotherapy in Colorectal Cancer | No | Irrelevant |
| 1977 | | Mukherjee et al., 2023 | | | Polymeric Nanoparticles as Tumor-Targeting Theranostic Platforms | No | Irrelevant |
| 1978 | | Ni et al., 2023 | | | Rethinking Glutamine Metabolism and Oncogene Regulation in Cancer | No | Irrelevant |
| 1979 | | Schrörs et al., 2023 | | | MC38 Colorectal Tumor Cell Lines from Two Sources Show Substantial Differences | No | Irrelevant |
| 1980 | | Zhu et al., 2023 | | | Neoadjuvant Immunotherapy for Colorectal Cancer: Right Regimens, Right Patients, Right Directions? | No | Irrelevant |
| 1981 | | Pan et al., 2023 | | | Roles and Therapeutic Implications of m6A Modification in Cancer Immunotherapy | No | Irrelevant |
| 1982 | | Zhang et al., 2023 | | | NAT10-Mediated mRNA Acetylation Modification in Colon Cancer | No | Irrelevant |
| 1983 | | Paley et al., 2023 | | | Microbiome Metabolome Brain Vagus Nerve Circuit in Disease and Recovery | No | Irrelevant |
| 1984 | | Wang et al., 2023 | | | Complexity of Pattern Recognition Receptors in Myeloid-Derived Suppressor Cells | No | Irrelevant |
| 1985 | | Andini et al., 2023 | | | PMS2-Associated Lynch Syndrome: Past, Present, and Future | No | Irrelevant |
| 1986 | | Martin et al., 2023 | | | VISTA Expression and Patient Selection for Immune-Based Anticancer Therapy | No | Irrelevant |
| 1987 | | Chen et al., 2023 | | | Arginine Metabolism in Immunotherapy for Colorectal Cancer | No | Irrelevant |
| 1988 | | Cicala et al., 2023 | | | Dostarlimab: From Preclinical Investigation to Drug Approval and Future Directions | No | Irrelevant |
| 1989 | | Wong et al., 2023 | | | New Developments in Targeted Therapy for Metastatic Colorectal Cancer | No | Irrelevant |
| 1990 | | No Authors Found, 2023 | | | Chinese Guidelines for Diagnosis and Treatment of Colorectal Liver Metastases | No | Irrelevant |
| 1991 | | Cai et al., 2023 | | | Tertiary Lymphoid Structures (TLS) in Gastrointestinal Tumors | No | Irrelevant |
| 1992 | | Fang et al., 2023 | | | Somatic Mutational Landscape of Mismatch Repair-Deficient Prostate Cancer | No | Irrelevant |
| 1993 | | Nishikawa et al., 2023 | | | Drugs Targeting p53 Mutations with FDA Approval and in Clinical Trials | No | Irrelevant |
| 1994 | | Birnbaum et al., 2023 | | | PVRIG Expression as a Prognostic Factor in Hepatocellular Carcinoma | No | Irrelevant |
| 1995 | | Decazes et al., 2023 | | | Body Composition to Define Prognosis of Cancers Treated by Anti-Angiogenic Drugs | No | Irrelevant |
| 1996 | | Marquardt et al., 2023 | | | Predicting Tumor Microenvironment in CXCR4- and FAP-Positive Solid Tumors | No | Irrelevant |
| 1997 | | Hosseini et al., 2023 | | | Cancer Vaccines for Triple-Negative Breast Cancer: A Systematic Review | No | Irrelevant |
| 1998 | | Veneziani et al., 2023 | | | Immunotherapy with Endosomal TLR Agonists to Improve NK Cell Function | No | Irrelevant |
| 1999 | | Granata et al., 2023 | | | Immunotherapy Assessment: A New Paradigm for Radiologists | No | Irrelevant |
| 2000 | | Xu et al., 2023 | | | Prognostic Role of Erythroferrone (ERFE) Gene in Human Malignancies | No | Irrelevant |
| 2001 | | Mohd Salim et al., 2023 | | | The Immunosuppressive Effect of TNFR2 Expression in the Colorectal Cancer Microenvironment | No | Irrelevant |
| 2002 | | O’Reilly et al., 2023 | | | Oncotherapeutic Strategies in Early Onset Colorectal Cancer | No | Irrelevant |
| 2003 | | Ohishi et al., 2023 | | | Current Targeted Therapy for Metastatic Colorectal Cancer | No | Irrelevant |
| 2004 | | Santoni et al., 2023 | | | Role of Clock Genes and Circadian Rhythm in Renal Cell Carcinoma: Recent Evidence and Therapeutic Consequences | No | Irrelevant |
| 2005 | | Pavel et al., 2023 | | | The Impact of Molecular Biology in the Seeding, Treatment Choices, and Follow-Up of Colorectal Cancer Liver Metastases—A Narrative Review | No | Irrelevant |
| 2006 | | Alketbi et al., 2023 | | | The Role of ATP-Binding Cassette Subfamily A in Colorectal Cancer Progression and Resistance | No | Irrelevant |
| 2007 | | Guo et al., 2023 | | | Research Progress of Immune Checkpoint LAG-3 in Gastric Cancer: A Narrative Review | No | Irrelevant |
| 2008 | | Chen et al., 2023 | | | Mechanisms and Strategies to Overcome PD-1/PD-L1 Blockade Resistance in Triple-Negative Breast Cancer | No | Irrelevant |
| 2009 | | Idrisova et al., 2023 | | | Role of Patient-Derived Models of Cancer in Translational Oncology | No | Irrelevant |
| 2010 | | Hu et al., 2023 | | | Strategies to Optimize Treatment for Locally Advanced Rectal Cancer | No | Irrelevant |
| 2011 | | Ganesan et al., 2023 | | | Microbiome and Metabolomics in Liver Cancer: Scientific Technology | No | Irrelevant |
| 2012 | | González-Arriagada et al., 2023 | | | Therapeutic Perspectives of HIV-Associated Chemokine Receptor (CCR5 and CXCR4) Antagonists in Carcinomas | No | Irrelevant |
| 2013 | | Élez et al., 2023 | | | A Comprehensive Biomarker Analysis of Microsatellite Unstable/Mismatch Repair Deficient Colorectal Cancer Cohort Treated with Immunotherapy | No | - |
| 2014 | | Kunac et al., 2023 | | | Activation of cGAS-STING Pathway Is Associated with MSI-H Stage IV Colorectal Cancer | No | - |
| 2015 | | Chang et al., 2023 | | | The Association between Baseline Proton Pump Inhibitors, Immune Checkpoint Inhibitors, and Chemotherapy: A Systematic Review with Network Meta-Analysis | No | Irrelevant |
| 2016 | | Goutas et al., 2023 | | | Immunohistochemical Study of Bladder Cancer Molecular Subtypes and Their Association with PD-L1 Expression | No | Irrelevant |
| 2017 | | Hsieh et al., 2023 | | | Fusobacterium Nucleatum-Induced Tumor Mutation Burden Predicts Poor Survival of Gastric Cancer Patients | No | Irrelevant |
| 2018 | | Chaudhary et al., 2023 | | | Transcriptional Factors Targeting in Cancer Stem Cells for Tumor Modulation | No | Irrelevant |
| 2019 | | Prakash et al., 2023 | | | Tumor-Derived Extracellular Vesicles in the Colorectal Cancer Immune Environment and Immunotherapy | No | Irrelevant |
| 2020 | | Mei et al., 2023 | | | Formin Protein DIAPH1 Positively Regulates PD-L1 Expression and Predicts the Therapeutic Response to Anti-PD-1/PD-L1 Immunotherapy | No | Irrelevant |
| 2021 | | Koerner et al., 2023 | | | Suppression of Prostate Cancer and Amelioration of the Immunosuppressive Tumor Microenvironment through Selective Immunoproteasome Inhibition | No | Irrelevant |
| 2022 | | Zheng et al., 2023 | | | Anti-PAI-1 Monoclonal Antibody Inhibits the Metastasis and Growth of Esophageal Squamous Cell Carcinoma | No | Irrelevant |
| 2023 | | El Ghazzi et al., 2023 | | | CAR-T Cells Development in Solid Tumors | No | Irrelevant |
| 2024 | | Wang et al., 2023 | | | Intratumoral Bacteria Are an Important “Accomplice” in Tumor Development and Metastasis | No | Irrelevant |
| 2025 | | Yerlikaya et al., 2023 | | | Molecular Pathways, Targeted Therapies, and Proteomic Investigations of Colorectal Cancer | No | Irrelevant |
| 2026 | | El-Tanani et al., 2023 | | | Cellular and Molecular Basis of Therapeutic Approaches to Breast Cancer | No | Irrelevant |
| 2027 | | Koukourakis et al., 2023 | | | The Molecular Basis of Immuno-Radiotherapy | No | Irrelevant |
| 2028 | | Patel et al., 2023 | | | Molecular Mechanisms and Future Implications of VEGF/VEGFR in Cancer Therapy | No | Irrelevant |
| 2029 | | He et al., 2023 | | | CD166-Specific CAR-T Cells Potently Target Colorectal Cancer Cells | No | Irrelevant |
| 2030 | | DasGupta et al., 2023 | | | Evolution of Precision Oncology-Guided Treatment Paradigms | No | Irrelevant |
| 2031 | | Kapoor et al., 2023 | | | Biomarkers for Immune Checkpoint Inhibitors in Solid Tumors | No | Irrelevant |
| 2032 | | Foster et al., 2023 | | | The Contemporary Management of Peritoneal Metastasis: A Journey from the Cold Past of Treatment Futility to a Warm Present and a Bright Future | No | Irrelevant |
| 2033 | | Coleman et al., 2023 | | | Systematic Evaluation of the Predictive Gene Expression Signatures of Immune Checkpoint Inhibitors in Metastatic Melanoma | No | Irrelevant |
| 2034 | | Tang et al., 2023 | | | A Generational Comparison for Unfavorable Cancer of Unknown Primary in a Single Institute Over 20 Years | No | Irrelevant |
| 2035 | | Parente et al., 2023 | | | MMR Profile and Microsatellite Instability Status in Colorectal Mucinous Adenocarcinoma with Synchronous Metastasis: A New Clue for Clinical Practice | No | Irrelevant |
| 2036 | | Perez-Castro et al., 2023 | | | Tryptophan and Its Metabolites in Normal Physiology and Cancer Etiology | No | Irrelevant |
| 2037 | | Koukourakis et al., 2023 | | | Combining the Past and Present to Advance Immuno-Radiotherapy of Cancer | No | Irrelevant |
| 2038 | | Guo et al., 2022 | | | Research Progress and Prospect of Siglec in Innate Immune Cells in Tumor | No | Irrelevant |
| 2039 | | Du et al., 2022 | | | Single-Cell RNA Sequencing Unveils Communications Between Malignant T and Myeloid Cells in Cutaneous T-Cell Lymphoma | No | Irrelevant |
| 2040 | | Mei et al., 2022 | | | Clinicopathological Characteristics of High Microsatellite Instability/Mismatch Repair-Deficient Colorectal Cancer: A Narrative Review | No | Irrelevant |
| 2041 | | Xie et al., 2022 | | | Structure-Specific Antitumor Effects and Potential Gut Microbiota-Involved Mechanisms of Ginseng Polysaccharides on B16F10 Melanoma-Bearing Mice | No | Irrelevant |
| 2042 | | Jin et al., 2022 | | | TIGIT: A promising target to overcome the barrier of immunotherapy in hematological malignancies | No | Different focus (hematological cancers) |
| 2043 | | Hao et al., 2022 | | | Synthetical lethality of Werner helicase and mismatch repair deficiency is mediated by p53 and PUMA in colon cancer | No | Different mechanism focus |
| 2044 | | Gogoi et al., 2022 | | | Nanotechnology for colorectal cancer detection and treatment | No | Different treatment approach |
| 2045 | | Liang et al., 2022 | | | Immune-related gene-based prognostic index for predicting survival and immunotherapy outcomes in colorectal carcinoma | No | Prognostic study, not treatment-focused |
| 2046 | | Li et al., 2022 | | | SOX chemotherapy with anti-PD-1 and iNKT cell immunotherapies for stage IV gastric adenocarcinoma with liver metastases: A case report | No | Focus on gastric cancer |
| 2047 | | Bergamo et al., 2022 | | | Complete pathologic response with first-line immunotherapy combination in MSI-H metastatic colorectal cancer | No | - |
| 2048 | | Chen et al., 2022 | | | Molecular profiling identifies distinct subtypes across TP53 mutant tumors | No | General cancer profiling |
| 2049 | | Glud et al., 2022 | | | Identification of high-risk immunogenic prostate cancer patients for T-cell engager immunotherapy | No | Focus on prostate cancer |
| 2050 | | Schwarz et al., 2022 | | | T cells of colorectal cancer patients stimulated by neoantigenic and cryptic peptides better recognize autologous tumor cells | No | - |
| 2051 | | Vachetta et al., 2022 | | | Opportunities, obstacles and challenges of flavonoids for breast cancer therapy | No | Focus on breast cancer |
| 2052 | | Ooi et al., 2022 | | | RAS and BRAF genes as biomarkers for personalized colorectal cancer therapy | No | Biomarker study |
| 2053 | | Ng et al., 2022 | | | Insights into the tumor microenvironment of B cell lymphoma | No | Focus on lymphoma |
| 2054 | | Loughrey et al., 2022 | | | Neoadjuvant immunotherapy and colorectal cancer treatment: Implications for surgery | No | - |
| 2055 | | Sinha et al., 2022 | | | Repurposing molecular target therapies to boost immune checkpoint blockade | No | General repurposing study |
| 2056 | | Geng et al., 2022 | | | Identification of DDX60 as a regulator of MHC-I class molecules in colorectal cancer | No | Mechanistic study |
| 2057 | | Ouyang et al., 2022 | | | T-Cell Mediated Immunity in Merkel Cell Carcinoma | No | Focus on Merkel Cell Carcinoma |
| 2058 | | Catalano et al., 2022 | | | Lung Cancer Immunotherapy: Beyond Common Immune Checkpoint Inhibitors | No | Focus on lung cancer |
| 2059 | | Li et al., 2022 | | | Prognostic and clinicopathological significance of Circular RNAs in pancreatic cancer | No | Focus on pancreatic cancer |
| 2060 | | Ephraim et al., 2022 | | | Cancer Immunotherapy: The Checkpoint between Chronic Colitis and Colorectal Cancer | No | - |
| 2061 | | Dazio et al., 2022 | | | Strategies to Overcome Resistance to Targeted Therapies and Immunotherapies in Metastatic Colorectal Cancer | No | - |
| 2062 | | Deng et al., 2022 | | | Harnessing NK Cells to Control Metastasis | No | NK cell-based therapy |
| 2063 | | Lau et al., 2022 | | | Decoding Strategies to Evade Immunoregulators in Cancer Immunotherapy | No | General immunoregulator study |
| 2064 | | Li et al., 2022 | | | Targeting the PI3K/AKT/mTOR and RAF/MEK/ERK pathways for cancer therapy | No | General targeted therapy |
| 2065 | | Han et al., 2022 | | | Clinical trajectory and immunobiology of microsatellite-instability-high cancers | No | General MSI-H study |
| 2066 | | Garzón-Hernández et al., 2022 | | | Molecular Targeted Therapy in Oncology Focusing on DNA Repair Mechanisms | No | General molecular therapy |
| 2067 | | Samson et al., 2022 | | | The cGAS–STING pathway and cancer | No | Pathway-focused study |
| 2068 | | Du et al., 2022 | | | Metformin in therapeutic applications in human diseases | No | Focus on metformin |
| 2069 | | Manni et al., 2022 | | | Signaling pathways in cancer stem cells and targeted therapy | No | Cancer stem cell focus |
| 2070 | | Liu et al., 2022 | | | Lipid Nanoparticles for Antitumor Immunity | No | Delivery mechanism focus |
| 2071 | | Gumà et al., 2022 | | | Utility of ctDNA Liquid Biopsies from Cancer Patients | No | Liquid biopsy study |
| 2072 | | Pawłowska et al., 2022 | | | Dual Blockade of TIGIT and PD-1/PD-L1 in Ovarian Cancer | No | Focus on ovarian cancer |
| 2073 | | Kist de Ruijter et al., 2022 | | | Whole-body CD8+ T cell visualization during cancer immunotherapy | No | Imaging study |
| 2074 | | Peng et al., 2022 | | | Prognostic value of microRNAs in small cell lung cancer | No | Focus on lung cancer |
| 2075 | | Sui et al., 2022 | | | Inflammation promotes resistance to immune checkpoint inhibitors in MSI-H colorectal cancer | No | - |
| 2076 | | Shasha et al., 2022 | | | Mechanisms of colorectal liver metastasis development | No | - |
| 2077 | | Parma et al., 2022 | | | Blocking heat-shock proteins to overcome drug resistance in lung cancer | No | Focus on lung cancer |
| 2078 | | Shayan et al., 2022 | | | Modifying oncolytic virotherapy to target hypoxic tumors | No | Oncolytic virus focus |
| 2079 | | Morales-Juarez et al., 2022 | | | Clinical prospects of WRN inhibition as a treatment for MSI tumors | No | MSI mechanism focus |
| 2080 | | He et al., 2022 | | | Combined immunotherapy for metastatic triple-negative breast cancer | No | Focus on breast cancer |
| 2081 | | DeRidder et al., 2022 | | | The past, present, and future of chemotherapy with a focus on individualization | No | Chemotherapy focus |
| 2082 | | Najafi et al., 2022 | | | Impact of microbiota on PD-1/PD-L1 inhibitor therapy in solid tumors | No | Microbiota focus |
| 2083 | | Liu et al., 2022 | | | Real-world practice of first-line treatment in advanced KRAS mutant NSCLC | No | Focus on NSCLC |
| 2084 | | Su et al., 2022 | | | Red blood cell-based vaccines for cancer chemoimmunotherapy | No | Vaccine-based therapy |
| 2085 | | Liu et al., 2022 | | | Novel insight into immunotherapy in gastrointestinal cancer | No | General GI cancer focus |
| 2086 | | Xia et al., 2022 | | | Role of Cuproptosis genes in renal cell carcinoma | No | Focus on renal cancer |
| 2087 | | Ramai et al., 2022 | | | Antibiotics, microbiome, and gastrointestinal cancers | No | Microbiome study |
| 2088 | | Lote et al., 2022 | | | Advances in immunotherapy for MMR proficient colorectal cancer | No | Focus on MMR proficient CRC |
| 2089 | | Xu et al., 2022 | | | Targeting the tumor stroma for cancer therapy | No | Tumor microenvironment focus |
| 2090 | | Zhong et al., 2022 | | | Warburg effect in colorectal cancer: tumor microenvironment and therapy | No | Metabolism-focused study |
| 2091 | | Tong et al., 2022 | | | NK cells and solid tumors: therapeutic potential and obstacles | No | NK cell-based therapy |
| 2092 | | Hollar et al., 2022 | | | Ecological resonances in quantum metabolic model of cancer | No | Theoretical metabolic model |
| 2093 | | Mortezaee et al., 2022 | | | VISTA immune regulatory effects in bypassing cancer immunotherapy | No | Alternative immune checkpoints |
| 2094 | | Zhao et al., 2022 | | | Immune checkpoint of B7-H3 in cancer immunotherapy | No | Alternative immune checkpoint |
| 2095 | | Toulmonde et al., 2022 | | | Randomized phase 2 trial of oncolytic virus JX-594 in sarcoma | No | Sarcoma-focused study |
| 2096 | | Patel et al., 2022 | | | The intestinal microbiota in colorectal cancer metastasis | No | Microbiota-focused study |
| 2097 | | Yu et al., 2022 | | | Interaction of microbiome and immunity in tumorigenesis and treatment | No | Microbiota focus |
| 2098 | | Li et al., 2022 | | | Molecular characteristics of multifocal esophageal squamous cell carcinomas | No | Esophageal cancer study |
| 2099 | | Yamada et al., 2022 | | | Clinicopathological and molecular characterization of dMMR colorectal cancer | No | - |
| 2100 | | Kong et al., 2022 | | | TP53 mutation-associated immune infiltration in HNSCC | No | Head and neck cancer focus |
| 2101 | | Akhoundova et al., 2022 | | | Molecular genetics of prostate cancer and genomic testing | No | Prostate cancer focus |
| 2102 | | Huang et al., 2022 | | | Targeting STING for cancer immunotherapy | No | STING pathway focus |
| 2103 | | Boudin et al., 2022 | | | CSPG4 expression in soft tissue sarcomas and prognosis | No | Sarcoma focus |
| 2104 | | Wang et al., 2022 | | | The emerging role of pyroptosis in pediatric cancers | No | Pediatric cancer focus |
| 2105 | | Wu et al., 2022 | | | Tumor antigens and vaccines in colorectal cancer | No | - |
| 2106 | | Liu et al., 2022 | | | Flavokawain A as PRMT5 inhibitor in bladder cancer | No | Bladder cancer focus |
| 2107 | | Faggiano et al., 2022 | | | Corticosteroids in oncology: guidelines and use | No | Drug guidelines focus |
| 2108 | | Louie et al., 2022 | | | Pan-cancer molecular tumor board experience in precision immunotherapy | No | Pan-cancer focus |
| 2109 | | Mandal et al., 2022 | | | Treating ARID1A mutated cancers via synthetic lethality | No | ARID1A mutation focus |
| 2110 | | Jurj et al., 2022 | | | Extracellular matrix alteration and drug resistance in cancer | No | Drug resistance mechanism |
| 2111 | | Xiong et al., 2022 | | | Direct and indirect effects of IFN-α2b in malignancy treatment | No | IFN-α2b therapy focus |
| 2112 | | Lam-Ubol et al., 2022 | | | Histone H3 modifications in salivary gland neoplasms | No | Salivary gland cancer focus |
| 2113 | | Fountzilas et al., 2022 | | | Clinical trial design in the era of precision medicine | No | Precision medicine focus |
| 2114 | | Sharafi et al., 2022 | | | Immune checkpoint inhibitors in hepatocellular carcinoma | No | Liver cancer focus |
| 2115 | | Wang et al., 2022 | | | Targeting tumor-resident myeloid cells for cancer immunotherapy | No | Tumor microenvironment focus |
| 2116 | | Guo et al., 2022 | | | Chemotherapeutic resistance and targeted nanoparticles in colorectal cancer | No | - |
| 2117 | | Höppener et al., 2022 | | | Primary colorectal cancer histology and liver metastases | No | - |
| 2118 | | Li et al., 2022 | | | Advances in nuclear medicine imaging for head and neck cancer | No | Head and neck cancer focus |
| 2119 | | Ju et al., 2022 | | | Circulating tumor cell detection: opportunities and challenges | No | Biomarker study |
| 2120 | | Xu et al., 2022 | | | NAD pathway and immune microenvironment in cancer | No | Metabolism focus |
| 2121 | | Wang et al., 2022 | | | Genomic signature of MTOR as immunogenicity marker in CRC | No | - |
| 2122 | | Zhang et al., 2022 | | | Immune checkpoint-related lncRNA signature in lung cancer | No | Lung cancer focus |
| 2123 | | Ma et al., 2022 | | | Glutamine metabolism and immune response in the tumor microenvironment | No | Tumor metabolism focus |
| 2124 | | Jung et al., 2022 | | | Tumor-infiltrating lymphocytes and prognostic immune subgroups in MSI-H CRC | No | - |
| 2125 | | Song et al., 2022 | | | 18F-FDG PET/CT metabolic activity predicts MSI status in CRC | No | Imaging study |
| 2126 | | Weng et al., 2022 | | | Exploring immunotherapy in colorectal cancer | No | - |
| 2127 | | Tang et al., 2022 | | | IGSF11 and VISTA: new immune checkpoints in tumor immunotherapy | No | Alternative checkpoint focus |
| 2128 | | Bell et al., 2022 | | | Immune response in colorectal carcinoma as predictive/prognostic biomarker | No | - |
| 2129 | | Otsuka et al., 2022 | | | CpG ODN (TLR9 agonist) and Th1 immune response in lung cancer | No | Lung cancer focus |
| 2130 | | Sadrekarimi et al., 2022 | | | Role of microbiome in cancer development and therapy response | No | Microbiome study |
| 2131 | | Silk et al., 2022 | | | Phase Ib study of troriluzole + nivolumab in solid tumors | No | Combination therapy focus |
| 2132 | | Cao et al., 2022 | | | Hepatic arterial infusion chemotherapy + regorafenib in CRC | No | - |
| 2133 | | He et al., 2022 | | | Targeting signaling pathways in prostate cancer | No | Prostate cancer focus |
| 2134 | | Xie et al., 2022 | | | C2orf40 regulates metastasis and drug resistance in nasopharyngeal carcinoma | No | Nasopharyngeal cancer focus |
| 2135 | | Mukherji et al., 2022 | | | Molecular profiling in gastrointestinal malignancies | No | General GI cancer focus |
| 2136 | | Kovács et al., 2022 | | | Transcriptomic datasets of cancer patients treated with ICIs | No | Data analysis study |
| 2137 | | Chu et al., 2022 | | | Natural killer cells as immunotherapy for cancer | No | NK cell-based therapy |
| 2138 | | Abdolahi et al., 2022 | | | Patient-derived xenograft (PDX) models in cancer research | No | PDX model focus |
| 2139 | | Matsuura et al., 2022 | | | Impact of PD-L1 in ovarian clear cell carcinoma | No | Ovarian cancer focus |
| 2140 | | John et al., 2022 | | | B7x expands Tregs and promotes resistance to anti-CTLA-4 therapy | No | Alternative immune checkpoint focus |
| 2141 | | Sibilio et al., 2022 | | | In-silico analysis reveals novel colorectal cancer subset for ICIs | No | - |
| 2142 | | Gao et al., 2022 | | | Single-cell N6-methyladenosine regulator patterns in CRC | No | - |
| 2143 | | Javed et al., 2022 | | | Prognostic factors of BRAF V600E CRC with liver metastases | No | - |
| 2144 | | Wang et al., 2022 | | | Gut microbiota modulation as a tool for CRC management | No | Microbiota focus |
| 2145 | | Sun et al., 2022 | | | Triptolide delivery: Traditional Chinese medicine & nanomedicine | No | Nanomedicine focus |
| 2146 | | Yue et al., 2022 | | | Classification of CRC subtypes based on ferroptosis markers | No | - |
| 2147 | | Ansari et al., 2022 | | | Cancer combination therapies using angiogenesis inhibitors | No | Angiogenesis focus |
| 2148 | | Tian et al., 2022 | | | Enhancing oncolytic viruses for cancer immunotherapy | No | Oncolytic virus focus |
| 2149 | | Fan et al., 2022 | | | Clinical significance of FBXW7 loss in human cancers | No | General cancer focus |
| 2150 | | Zhou et al., 2022 | | | Notch signaling pathway: architecture, disease, and therapy | No | Notch pathway focus |
| 2151 | | Raza et al., 2022 | | | Liquid biopsy biomarkers in CRC | No | - |
| 2152 | | Zhou et al., 2022 | | | CRC liver metastasis: molecular mechanism and therapy | No | - |
| 2153 | | Zhou et al., 2022 | | | Circadian rhythms and cancer: links and therapeutic potential | No | Circadian rhythm focus |
| 2154 | | Mekadim et al., 2022 | | | Dysbiosis of skin & gut microbiome in melanoma progression | No | Melanoma focus |
| 2155 | | Feng et al., 2022 | | | PD-L1 and mTOR pathway in rectal cancer | No | - |
| 2156 | | Khorana et al., 2022 | | | Cancer-associated venous thromboembolism | No | Blood clot focus |
| 2157 | | Xie et al., 2022 | | | Genetic mutation profiles in pulmonary enteric adenocarcinoma | No | Lung cancer focus |
| 2158 | | Jin et al., 2022 | | | Emerging new therapeutic antibody derivatives for cancer | No | Antibody therapy focus |
| 2159 | | Salvatori et al., 2022 | | | Neoantigen cancer vaccine boosts anti-CTLA-4 efficacy | No | Cancer vaccine focus |
| 2160 | | Wohlfeil et al., 2022 | | | Angiogenesis & molecular diversity in hepatic melanoma metastasis | No | Melanoma focus |
| 2161 | | Kwan et al., 2022 | | | KRASG12C inhibitors: A comprehensive review | No | KRAS mutation focus |
| 2162 | | Du et al., 2022 | | | BTNL2 promotes immune escape via IL-17A γδ T cells | No | Immune evasion focus |
| 2163 | | Yin et al., 2022 | | | High immune infiltration based on peroxisome pathway & TIM3 in CRC | No | - |
| 2164 | | Liu et al., 2022 | | | Wnt/β-catenin signaling: Function & therapeutic opportunities | No | Wnt pathway focus |
| 2165 | | Tang et al., 2022 | | | Immune landscape & immunotherapy in penile cancer | No | Penile cancer focus |
| 2166 | | Ciernikova et al., 2022 | | | Tumor microbiome as part of tumor microenvironment | No | Microbiome focus |
| 2167 | | Joshi et al., 2022 | | | Inhibition of cancer cell metastasis by nanotherapeutics | No | Nanomedicine focus |
| 2168 | | Hou et al., 2022 | | | Predictive biomarkers of colon cancer immunotherapy | No | - |
| 2169 | | Nasiri et al., 2022 | | | CAR-T cell therapy in triple-negative breast cancer | No | Breast cancer focus |
| 2170 | | Jiang et al., 2022 | | | DDR axis refines classification of ccRCC | No | Kidney cancer focus |
| 2171 | | Liu et al., 2022 | | | Epigenetic mutations as biomarkers for ICIs in MSS CRC | No | - |
| 2172 | | Lyubetskaya et al., 2022 | | | Spatial transcriptomics in oncology discovery | No | Genomic profiling focus |
| 2173 | | Yousefi et al., 2022 | | | NSC inhibitor + doxorubicin in triple-negative breast cancer | No | Breast cancer focus |
| 2174 | | Doleschal et al., 2022 | | | Anti-EGFR targeting in metastatic CRC | No | - |
| 2175 | | Huang et al., 2022 | | | Microbiome’s role in cancer: Targeting metabolism | No | Microbiome focus |
| 2176 | | Ghazi et al., 2022 | | | CAR T-cells for colorectal cancer immunotherapy | No | - |
| 2177 | | Ren et al., 2022 | | | KIR2DL5/PVR pathway blockade in NK-cell therapy | No | NK cell therapy focus |
| 2178 | | Deng et al., 2022 | | | RAD21 amplification suppresses IFN signaling in ovarian cancer | No | Ovarian cancer focus |
| 2179 | | Bloudek et al., 2022 | | | Oncology simulation model for survival estimation | No | Data modeling focus |
| 2180 | | Zheng et al., 2022 | | | Angiogenesis & immunosuppression in tumor microenvironment | No | Tumor microenvironment focus |
| 2181 | | Hou et al., 2022 | | | Gut microbiota’s effects on immune response & CRC immunotherapy | No | Microbiome focus |
| 2182 | | Zhang et al., 2022 | | | MADET: Microbiomic effects on anticancer treatment efficacy | No | Microbiome focus |
| 2183 | | Layos et al., 2022 | | | Curcumin as therapy for colorectal cancer patients | No | Natural compound focus |
| 2184 | | Al-Hujaily et al., 2022 | | | Recruiting immunity against CRC: Challenges & status | No | - |
| 2185 | | Agrawal et al., 2022 | | | Food bioactive compounds against cancer | No | Dietary compound focus |
| 2186 | | Yu et al., 2022 | | | Microbiome’s role in CRC pathogenesis & treatment | No | Microbiome focus |
| 2187 | | Ullah et al., 2022 | | | Multi-omics approaches in CRC screening & diagnosis | No | - |
| 2188 | | Qi et al., 2022 | | | The Species of Gut Bacteria Associated with Antitumor Immunity in Cancer Therapy | No | Different focus |
| 2189 | | Roussot et al., 2022 | | | Tumor Immunogenic Cell Death as a Mediator of Intratumor CD8 T-Cell Recruitment | No | Different focus |
| 2190 | | Li et al., 2022 | | | A Promising Research Direction for Colorectal Cancer Immunotherapy: The Regulatory Mechanism of CCL5 in Colorectal Cancer | No | Different focus |
| 2191 | | Tumino et al., 2022 | | | Myeloid Derived Suppressor Cells in Tumor Microenvironment: Interaction with Innate Lymphoid Cells | No | Different focus |
| 2192 | | Brown et al., 2022 | | | Molecular Targeting of the Most Functionally Complex Gene in Precision Oncology: p53 | No | Different focus |
| 2193 | | Chung et al., 2022 | | | PD-L1 Expression in High-Risk Early-Stage Colorectal Cancer—Its Clinical and Biological Significance in Immune Microenvironment | No | Different focus |
| 2194 | | Sági et al., 2022 | | | The Influence of the Gut Microbiome in Paediatric Cancer Origin and Treatment | No | Different focus |
| 2195 | | Zhang et al., 2022 | | | Targeting Chromatin-Remodeling Factors in Cancer Cells: Promising Molecules in Cancer Therapy | No | Different focus |
| 2196 | | Galoș et al., 2022 | | | Clinical Applications of Liquid Biopsy in Colorectal Cancer Screening: Current Challenges and Future Perspectives | No | Different focus |
| 2197 | | Dang et al., 2022 | | | Ferroptosis: A Double-Edged Sword Mediating Immune Tolerance of Cancer | No | Different focus |
| 2198 | | Jones et al., 2022 | | | The Potential Prognostic and Therapeutic Implications of Prolactin Receptor and Growth Hormone-Releasing Hormone Receptor Expression in Uterine Leiomyosarcomas | No | Different focus |
| 2199 | | Munari et al., 2022 | | | Immunotherapy Targeting Inhibitory Checkpoints: The Role of NK and Other Innate Lymphoid Cells | No | Different focus |
| 2200 | | Xiang et al., 2022 | | | Preoperative Carcinoembryonic Antigen to Body Mass Index Ratio Contributes to Prognosis Prediction in Colorectal Cancer | No | Different focus |
| 2201 | | Iyer et al., 2022 | | | Lost in Translation: Revisiting the Use of Tyrosine Kinase Inhibitors in Colorectal Cancer | No | Different focus |
| 2202 | | Ma et al., 2022 | | | Epigenetic Modifications: Critical Participants of the PD‑L1 Regulatory Mechanism in Solid Tumors | No | Different focus |
| 2203 | | Taieb et al., 2022 | | | Deficient Mismatch Repair/Microsatellite Unstable Colorectal Cancer: Diagnosis, Prognosis and Treatment | No | Different focus |
| 2204 | | Kepenekian et al., 2022 | | | Advances in the Management of Peritoneal Malignancies | No | Different focus |
| 2205 | | Su et al., 2022 | | | Current Landscape and Perspective of Oncolytic Viruses and Their Combination Therapies | No | Different focus |
| 2206 | | Marcu et al., 2022 | | | Developments on Tumour Site-Specific Chrono-Oncology Towards Personalised Treatment | No | Different focus |
| 2207 | | Luo et al., 2022 | | | Inducing Vascular Normalization: A Promising Strategy for Immunotherapy | No | Different focus |
| 2208 | | Yang et al., 2022 | | | Recent Advances in the Development of Transplanted Colorectal Cancer Mouse Models | No | Different focus |
| 2209 | | Goto et al., 2022 | | | Microbiota and Lung Cancer | No | Different focus |
| 2210 | | Sun et al., 2022 | | | Prognostic Perspectives of STING and PD-L1 Expression and Correlation with the Prognosis of Epstein-Barr Virus-Associated Gastric Cancers | No | Different focus |
| 2211 | | Kazan et al., 2022 | | | The Association Between PI3K, JAK/STAT Pathways with the PDL-1 Expression in Prostate Cancer | No | Different focus |
| 2212 | | Braoudaki et al., 2022 | | | Chemokines and Chemokine Receptors in Colorectal Cancer; Multifaceted Roles and Clinical Impact | No | Different focus |
| 2213 | | Schoutrop et al., 2022 | | | Molecular, Cellular and Systemic Aspects of Epithelial Ovarian Cancer and Its Tumor Microenvironment | No | Different focus |
| 2214 | | Kaneta et al., 2022 | | | Role of the cGAS-STING Pathway in Regulating the Tumor-Immune Microenvironment in dMMR/MSI Colorectal Cancer | No | Different focus |
| 2215 | | Kaushik et al., 2022 | | | The Evolutionary Legacy of Immune Checkpoint Inhibitors | No | Different focus |
| 2216 | | Alsayed et al., 2022 | | | Epigenetic Regulation of CXCR4 Signaling in Cancer Pathogenesis and Progression | No | Different focus |
| 2217 | | Haddad et al., 2022 | | | Resistance to Immune Checkpoint Blockade: Mechanisms, Counter-Acting Approaches, and Future Directions | No | Different focus |
| 2218 | | Wang et al., 2022 | | | Metabolic Modulation of Immune Checkpoints and Novel Therapeutic Strategies in Cancer | No | Different focus |
| 2219 | | Zeng et al., 2022 | | | Molecular Pathogenesis, Targeted Therapies, and Future Perspectives for Gastric Cancer | No | Different focus |
| 2220 | | Wang et al., 2022 | | | Dynamic Impact of Virome on Colitis and Colorectal Cancer: Immunity, Inflammation, Prevention and Treatment | No | Different focus |
| 2221 | | Roberti et al., 2022 | | | Impact of the Ileal Microbiota on Colon Cancer | No | Different focus |
| 2222 | | Im et al., 2022 | | | Immune Functions as a Ligand or a Receptor, Cancer Prognosis Potential, Clinical Implication of VISTA in Cancer Immunotherapy | No | Different focus |
| 2223 | | How et al., 2022 | | | How Far Have We Explored Fungi to Fight Cancer? | No | Different focus |
| 2224 | | Feng et al., 2022 | | | Ginsenosides: Allies of Gastrointestinal Tumor Immunotherapy | No | Different focus |
| 2225 | | Zhang et al., 2022 | | | Clinical Strategy of Conversion Therapy and Surgical Treatment for Liver Metastases from Colorectal Cancer | No | Different focus |
| 2226 | | Wei et al., 2022 | | | Current Landscape and Future Directions of Bispecific Antibodies in Cancer Immunotherapy | No | Different focus |
| 2227 | | Kazemi et al., 2022 | | | Tumor-Infiltrating Lymphocytes for Treatment of Solid Tumors: It Takes Two to Tango? | No | Different focus |
| 2228 | | Burkert et al., 2022 | | | Nitrogen-Doped Carbon Nanotube Cups for Cancer Therapy | No | Different focus |
| 2229 | | Reitsam et al., 2022 | | | Concurrent Loss of MLH1, PMS2 and MSH6 Immunoexpression in Digestive System Cancers Indicating a Widespread Dysregulation in DNA Repair Processes | No | Different focus |
| 2230 | | Chang et al., 2022 | | | Integrating Transcriptomics and Network Analysis-Based Multiplexed Drug Repurposing to Screen Drug Candidates for M2 Macrophage-Associated Castration-Resistant Prostate Cancer Bone Metastases | No | Different focus |
| 2231 | | Shivatare et al., 2022 | | | Glycoconjugates: Synthesis, Functional Studies, and Therapeutic Developments | No | Different focus |
| 2232 | | Kang et al., 2022 | | | Immunotherapeutic Progress and Application of Bispecific Antibody in Cancer | No | Different focus |
| 2233 | | Gao et al., 2022 | | | PET/CT Molecular Imaging in the Era of Immune-Checkpoint Inhibitors Therapy | No | Different focus |
| 2234 | | Vangala et al., 2022 | | | Novel Treatment Concepts in Patients with Colorectal Carcinomas and High Microsatellite Instability | No | Different focus |
| 2235 | | Meng et al., 2022 | | | Survival for Patients with Metastatic Colon Cancer Underwent Cytoreductive Colectomy in the Era of Rapid Development of Anticancer Drugs: A Real-World Analysis Based on Updated Population Dataset of 2004–2018 | No | Different focus |
| 2236 | | Wang et al., 2022 | | | Comprehensive Analysis of Transient Receptor Potential Channels-Related Signature for Prognosis, Tumor Immune Microenvironment, and Treatment Response of Colorectal Cancer | No | Different focus |
| 2237 | | Mao et al., 2022 | | | The Affinity of Antigen-Binding Domain on the Antitumor Efficacy of CAR T Cells: Moderate is Better | No | Different focus |
| 2238 | | Li et al., 2022 | | | Genomic Landscape of Microsatellite Instability in Chinese Tumors: A Comparison of Chinese and TCGA Cohorts | No | Different focus |
| 2239 | | Jiang et al., 2022 | | | A Comprehensive Investigation Discovered the Novel Methyltransferase METTL24 as One Presumably Prognostic Gene for Kidney Renal Clear Cell Carcinoma Potentially Modulating Tumor Immune Microenvironment | No | Different focus |
| 2240 | | Lugat et al., 2022 | | | Immuno-PET: Design Options and Clinical Proof-of-Concept | No | Different focus |
| 2241 | | Qiu et al., 2022 | | | FOXP3+ Regulatory T Cells and the Immune Escape in Solid Tumours | No | Different focus |
| 2242 | | Ma et al., 2022 | | | Targeting FGL2 in Glioma Immunosuppression and Malignant Progression | No | Different focus |
| 2243 | | Jin et al., 2022 | | | Gut Microbiota Distinct Between Colorectal Cancers with Deficient and Proficient Mismatch Repair: A Study of 230 CRC Patients | No | Different focus |
| 2244 | | Saoudi González et al., 2022 | | | Current and Emerging Anti-Angiogenic Therapies in Gastrointestinal and Hepatobiliary Cancers | No | Different focus |
| 2245 | | Luo et al., 2022 | | | Bacteria-Mediated Cancer Therapy: A Versatile Bio-Sapper with Translational Potential | No | Different focus |
| 2246 | | Sun et al., 2022 | | | Identification of Microsatellite Instability and Immune-Related Prognostic Biomarkers in Colon Adenocarcinoma | No | Different focus |
| 2247 | | Kaseb et al., 2022 | | | Blockade of Growth Hormone Receptor Signaling by Using Pegvisomant: A Functional Therapeutic Strategy in Hepatocellular Carcinoma | No | Different focus |
| 2248 | | Charbonneau et al., 2022 | | | Establishment of a ccRCC Patient-Derived Chick Chorioallantoic Membrane Model for Drug Testing | No | Different focus |
| 2249 | | Dwivedi et al., 2022 | | | Transcriptome: A Tool for Precision Oncology | No | Different focus |
| 2250 | | Yuan et al., 2022 | | | Immunotherapies Catering to the Unmet Medical Need of Cold Colorectal Cancer | No | Different focus |
| 2251 | | López-Mora et al., 2022 | | | Nuclear Medicine in the Assessment of Adverse Effects of Cancer Therapy in the Lung, Kidney, Gastrointestinal Tract, and Central Nervous System | No | Different focus |
| 2252 | | Jou et al., 2022 | | | Emerging Roles for IL-25 and IL-33 in Colorectal Cancer Tumorigenesis | No | Different focus |
| 2253 | | Shi et al., 2022 | | | Regulation of Survivin and Caspase/Bcl-2/Cyto-C Signaling by TDB-6 Induces Apoptosis of Colorectal Carcinoma LoVo Cells | No | Different focus |
| 2254 | | Li et al., 2022 | | | Prognostic Value of Plasma D-Dimer Levels in Advanced Non-Small Cell Lung Cancer Patients Treated with Immune Checkpoint Inhibitors: A Retrospective Study | No | Different focus |
| 2255 | | Wakamiya et al., 2022 | | | Usefulness of Immunostaining Scoring for Predicting Response to Single-Agent Nivolumab in Patients with Metastatic Renal Cell Carcinoma | No | Different focus |
| 2256 | | Wei et al., 2022 | | | Immunoregulatory Signal Networks and Tumor Immune Evasion Mechanisms: Insights into Therapeutic Targets and Agents in Clinical Development | No | Different focus |
| 2257 | | Mastracci et al., 2022 | | | PD-L1 Evaluation in the Gastrointestinal Tract: From Biological Rationale to Its Clinical Application | No | Different focus |
| 2258 | | Cho et al., 2022 | | | Artificial Intelligence-Powered Whole-Slide Image Analyzer Reveals a Distinctive Distribution of Tumor-Infiltrating Lymphocytes in Neuroendocrine Neoplasms | No | Different focus |
| 2259 | | Borelli et al., 2022 | | | Immune-Checkpoint Inhibitors (ICIs) in Metastatic Colorectal Cancer (mCRC) Patients Beyond Microsatellite Instability | No | Different focus |
| 2260 | | Anselmo et al., 2022 | | | Disappearing Colorectal Liver Metastases: Do We Really Need a Ghostbuster? | No | Different focus |
| 2261 | | Chen et al., 2022 | | | The Optimal Therapy After Progression on Immune Checkpoint Inhibitors in MSI Metastatic Gastrointestinal Cancer Patients: A Multicenter Retrospective Cohort Study | No | Different focus |
| 2262 | | Wang et al., 2022 | | | Emerging Role of ERBB2 in Targeted Therapy for Metastatic Colorectal Cancer: Signaling Pathways to Therapeutic Strategies | No | Different focus |
| 2263 | | Voutsadakis et al., 2022 | | | Sensitivities and Dependencies of BRAF Mutant Colorectal Cancer Cell Lines with or Without PIK3CA Mutations for Discovery of Vulnerabilities with Therapeutic Potential | No | Different focus |
| 2264 | | Maiorano et al., 2022 | | | The Interplay Between Anti-Angiogenics and Immunotherapy in Colorectal Cancer | No | Different focus |
| 2265 | | Cherkasova et al., 2022 | | | Use of Cannabis and Cannabinoids for Treatment of Cancer | No | Different focus |
| 2266 | | Volovat et al., 2022 | | | Use of Personalized Biomarkers in Metastatic Colorectal Cancer and the Impact of AI | No | Different focus |
| 2267 | | Cerrato-Izaguirre et al., 2022 | | | Somatic Mutational Landscape in Mexican Patients: CDH1 Mutations and chr20q13.33 Amplifications Are Associated with Diffuse-Type Gastric Adenocarcinoma | No | Different focus |
| 2268 | | Tojjari et al., 2022 | | | The Crosstalk Between Microbiome and Immunotherapeutics: Myth or Reality | No | Different focus |
| 2269 | | Hewitt et al., 2022 | | | The Role of Biomarkers in the Management of Colorectal Liver Metastases | No | Different focus |
| 2270 | | Walker et al., 2022 | | | Liquid Biopsy and the Translational Bridge from the TIME to the Clinic | No | Different focus |
| 2271 | | Bartley et al., 2022 | | | Mismatch Repair and Microsatellite Instability Testing for Immune Checkpoint Inhibitor Therapy: Guideline from the College of American Pathologists | No | Different focus |
| 2272 | | Gerger et al., 2022 | | | Retrospective Analysis of Treatment Pathways in Patients with BRAFV600E-mutant Metastatic Colorectal Carcinoma - MORSECRC | No | Different focus |
| 2273 | | Subbiah et al., 2022 | | | Tumour-Agnostic Efficacy and Safety of Selpercatinib in Patients with RET Fusion-Positive Solid Tumours | No | Different focus |
| 2274 | | Xue et al., 2022 | | | A Novel Prognostic Index of Stomach Adenocarcinoma Based on Immunogenomic Landscape Analysis and Immunotherapy Options | No | Different focus |
| 2275 | | Nojima et al., 2022 | | | Class IV Semaphorins in Disease Pathogenesis | No | Different focus |
| 2276 | | Tao et al., 2022 | | | Metastatic Ovarian Tumor from Pancreatic Cancer Treated with Combined Immunotherapy: A Case Report | No | Different focus |
| 2277 | | Dieterich et al., 2022 | | | Lymphatic Vessels in Cancer | No | Different focus |
| 2278 | | Nevo et al., 2022 | | | Thymosin Alpha 1 as an Adjuvant to Hyperthermic Intraperitoneal Chemotherapy in an Experimental Model of Peritoneal Metastases from Colonic Carcinoma | No | Different focus |
| 2279 | | Potiron et al., 2022 | | | Clinical Research in Radiation Oncology: How to Move from the Laboratory to the Patient? | No | Different focus |
| 2280 | | Zhan et al., 2022 | | | CD155 in Tumor Progression and Targeted Therapy | No | Different focus |
| 2281 | | Ivleva et al., 2022 | | | Microbiota-Driven Mechanisms at Different Stages of Cancer Development | No | Different focus |
| 2282 | | Li et al., 2022 | | | Blockade of IL-6 Inhibits Tumor Immune Evasion and Improves Anti–PD-1 Immunotherapy | No | Different focus |
| 2283 | | Li et al., 2022 | | | Correlation Between PD-1/PD-L1 Expression and Polarization in Tumor-Associated Macrophages: A Key Player in Tumor Immunotherapy | No | Different focus |
| 2284 | | Yukihiro et al., 2022 | | | Impact of Modified Glasgow Prognostic Score on Predicting Prognosis and Modification of Risk Model for Patients with Metastatic Renal Cell Carcinoma Treated with First-Line Tyrosine Kinase Inhibitor | No | Different focus |
| 2285 | | Inamura et al., 2022 | | | Cancer as Microenvironmental, Systemic and Environmental Diseases: Opportunity for Transdisciplinary Microbiomics Science | No | Different focus |
| 2286 | | Zhao et al., 2022 | | | Colorectal Cancer Immunotherapy-Recent Progress and Future Directions | No | Different focus |
| 2287 | | Sartore-Bianchi et al., 2022 | | | Application of Histology-Agnostic Treatments in Metastatic Colorectal Cancer | No | Different focus |
| 2288 | | Brancolini et al., 2022 | | | HDACs and the Epigenetic Plasticity of Cancer Cells: Target the Complexity | No | Different focus |
| 2289 | | Ding et al., 2022 | | | Associating Resistance to Immune Checkpoint Inhibitors with Immunological Escape in Colorectal Cancer | No | Different focus |
| 2290 | | Ziegler et al., 2022 | | | Modulation of the Intestinal Microbiota Impacts the Efficacy of Immunotherapy in Cancer Patients-A Recent Literature Survey | No | Different focus |
| 2291 | | Camero et al., 2022 | | | Radioresistance in Rhabdomyosarcomas: Much More Than a Question of Dose | No | Different focus |
| 2292 | | He et al., 2022 | | | Contribution of Immune Cells to Bone Metastasis Pathogenesis | No | Different focus |
| 2293 | | Lam et al., 2022 | | | Clinical Applications of Circulating Tumor-Derived DNA in the Management of Gastrointestinal Cancers – Current Evidence and Future Directions | No | Different focus |
| 2294 | | Prieto-Fernández et al., 2022 | | | Pathobiological Functions and Clinical Implications of Annexin Dysregulation in Human Cancers | No | Different focus |
| 2295 | | Tang et al., 2022 | | | miR-Aculous New Avenues for Cancer Immunotherapy | No | Different focus |
| 2296 | | Son et al., 2022 | | | The Microbiome-Immune Axis Therapeutic Effects in Cancer Treatments | No | Different focus |
| 2297 | | Ya et al., 2022 | | | Role of Myeloid-Derived Suppressor Cells in the Formation of Pre-Metastatic Niche | No | Different focus |
| 2298 | | Shan et al., 2022 | | | Mechanism and Strategies of Immunotherapy Resistance in Colorectal Cancer | No | Different focus |
| 2299 | | Keenan et al., 2022 | | | Circulating Monocytes Associated with Anti-PD-1 Resistance in Human Biliary Cancer Induce T Cell Paralysis | No | Different focus |
| 2300 | | Zhou et al., 2022 | | | A Newly Defined Basement Membrane-Related Gene Signature for the Prognosis of Clear-Cell Renal Cell Carcinoma | No | Different focus |
| 2301 | | Hernandez-Guerrero et al., 2022 | | | GITR Antibodies in Cancer: Not Ready for Prime Time | No | Different focus |
| 2302 | | Koustas et al., 2022 | | | Implication of Gut Microbiome in Immunotherapy for Colorectal Cancer | No | Different focus |
| 2303 | | Hai et al., 2022 | | | The Emerging Roles of HDACs and Their Therapeutic Implications in Cancer | No | Different focus |
| 2304 | | Hintzen et al., 2022 | | | Engaging Innate Immunity for Targeting the Epidermal Growth Factor Receptor: Therapeutic Options Leveraging Innate Immunity Versus Adaptive Immunity Versus Inhibition of Signaling | No | Different focus |
| 2305 | | Chen et al., 2022 | | | Development and Validation of a Novel Necroptosis-Related Score to Improve the Outcomes of Clear Cell Renal Cell Carcinoma | No | Different focus |
| 2306 | | Yang et al., 2022 | | | Current Progress and Future Perspectives of Neoadjuvant Anti-PD-1/PD-L1 Therapy for Colorectal Cancer | No | Different focus |
| 2307 | | Han et al., 2022 | | | Pan-Cancer Analysis Reveals Interleukin-17 Family Members as Biomarkers in the Prediction for Immune Checkpoint Inhibitor Curative Effect | No | Different focus |
| 2308 | | Peipei et al., 2022 | | | SMARCA4‑Deficient Undifferentiated Carcinoma of the Gastrointestinal Tract | No | Different focus |
| 2309 | | Zhang et al., 2022 | | | Drug Repurposing of Ivermectin Abrogates Neutrophil Extracellular Traps and Prevents Melanoma Metastasis | No | Different focus |
| 2310 | | Liu et al., 2022 | | | Immunomodulatory Effects of Regorafenib: Enhancing the Efficacy of Anti-PD-1/PD-L1 Therapy | No | Different focus |
| 2311 | | Fang et al., 2022 | | | Immunotherapy Resistance in Esophageal Cancer: Possible Mechanisms and Clinical Implications | No | Different focus |
| 2312 | | Lian et al., 2022 | | | The Role of Polyamine Metabolism in Remodeling Immune Responses and Blocking Therapy within the Tumor Immune Microenvironment | No | Different focus |
| 2313 | | Rui et al., 2022 | | | The Landscape of TIGIT Target and Clinical Application in Diseases | No | Different focus |
| 2314 | | Rallis et al., 2022 | | | IL-10 in Cancer: An Essential Thermostatic Regulator Between Homeostatic Immunity and Inflammation | No | Different focus |
| 2315 | | Shi et al., 2022 | | | The Implications from the Interplay of Neoadjuvant Chemoradiotherapy and the Immune Microenvironment in Rectal Cancer | No | Different focus |
| 2316 | | Centuori et al., 2022 | | | C-Met Signaling as a Therapeutic Target in Head and Neck Cancer | No | Different focus |
| 2317 | | Cheng et al., 2022 | | | Composition of the Gut Microbiota Associated with the Response to Immunotherapy in Advanced Cancer Patients | No | Different focus |
| 2318 | | Ivanova et al., 2022 | | | HER2 in Metastatic Colorectal Cancer: Pathology, Somatic Alterations, and Perspectives for Novel Therapeutic Schemes | No | Different focus |
| 2319 | | Villanueva-Fernández et al., 2022 | | | Biomarkers for Immunotherapy in Poorly Differentiated Sinonasal Tumors | No | Different focus |
| 2320 | | Al-Mterin et al., 2022 | | | Correlations Between Circulating and Tumor-Infiltrating CD4+ Treg Subsets with Immune Checkpoints in Colorectal Cancer Patients | No | Different focus |
| 2321 | | Dai et al., 2022 | | | Synergetic Thermal Therapy for Cancer: State-of-the-Art and the Future | No | Different focus |
| 2322 | | Kuzevanova et al., 2022 | | | The Features of Checkpoint Receptor—Ligand Interaction in Cancer and the Therapeutic Effectiveness of Their Inhibition | No | Different focus |
| 2323 | | Friedrich et al., 2022 | | | The Urinary Microbiome: Role in Bladder Cancer and Treatment | No | Different focus |
| 2324 | | Verzella et al., 2022 | | | The NF-κB Pharmacopeia: Novel Strategies to Subdue an Intractable Target | No | Different focus |
| 2325 | | Kanchan et al., 2022 | | | To Kill a Cancer: Targeting the Immune Inhibitory Checkpoint Molecule, B7-H3 | No | Different focus |
| 2326 | | Chen et al., 2022 | | | Colorectal Cancer: Metabolic Interactions Reshape the Tumor Microenvironment | No | Different focus |
| 2327 | | Chen et al., 2022 | | | Significance of Hypermethylation of Tumor-Suppressor Genes PTGER4 and ZNF43 at CpG Sites in the Prognosis of Colorectal Cancer | No | Different focus |
| 2328 | | Piringer et al., 2022 | | | Molecular Profiling in Colorectal Cancer | No | Different focus |
| 2329 | | Dong et al., 2022 | | | Pan-Cancer Analysis of Forkhead Box Q1 as a Potential Prognostic and Immunological Biomarker | No | Different focus |
| 2330 | | Ducoin et al., 2022 | | | Defining the Immune Checkpoint Landscape in Human Colorectal Cancer Highlights the Relevance of the TIGIT/CD155 Axis for Optimizing Immunotherapy | No | Different focus |
| 2331 | | Cekani et al., 2022 | | | Molecular Biology and Therapeutic Perspectives for K-Ras Mutant Non-Small Cell Lung Cancers | No | Different focus |
| 2332 | | Füchsl et al., 2022 | | | Paving the Way to Solid Tumors: Challenges and Strategies for Adoptively Transferred Transgenic T Cells in the Tumor Microenvironment | No | Different focus |
| 2333 | | Jo et al., 2022 | | | Targeting HDAC6 to Overcome Autophagy-Promoted Anti-Cancer Drug Resistance | No | Different focus |
| 2334 | | Ritterhouse et al., 2022 | | | Molecular Biomarkers of Response to Cancer Immunotherapy | No | Different focus |
| 2335 | | Silva et al., 2022 | | | The Current and Evolving Role of Immunotherapy in Metastatic Colorectal Cancer | No | Different focus |
| 2336 | | Ledderose et al., 2022 | | | Tumor-infiltrating lymphocytes predict survival in ≥ pT2 urothelial bladder cancer | No | Different focus |
| 2337 | | Pérez-López et al., 2022 | | | Embolization therapy with microspheres for the treatment of liver cancer: State-of-the-art of clinical translation | No | Different focus |
| 2338 | | Katipally et al., 2022 | | | The oligometastatic spectrum in the era of improved detection and modern systemic therapy | No | Different focus |
| 2339 | | Taghizadeh et al., 2022 | | | Immune Checkpoint Inhibitors for Advanced Biliary Tract Cancer | No | Different focus |
| 2340 | | Serrablo et al., 2022 | | | Current indications of ex-situ liver resection: A systematic review | No | Different focus |
| 2341 | | Shao et al., 2022 | | | Clinical significance of B7-H3 and HER2 co-expression and therapeutic value of combination treatment in gastric cancer | No | Different focus |
| 2342 | | Sobhani et al., 2022 | | | Therapeutic cancer vaccines: From biological mechanisms and engineering to ongoing clinical trials | No | Different focus |
| 2343 | | Andrei et al., 2022 | | | Integrated approaches for precision oncology in colorectal cancer: The more you know, the better | No | Different focus |
| 2344 | | Liu et al., 2022 | | | Immune checkpoint inhibitor-based therapy for advanced clear cell renal cell carcinoma: A narrative review | No | Different focus |
| 2345 | | Liu et al., 2022 | | | A novel fatty acid metabolism-related gene signature predicts the prognosis, tumor immune properties, and immunotherapy response of colon adenocarcinoma patients | No | Different focus |
| 2346 | | Palkina et al., 2022 | | | miR-204-5p in vivo inhibition causes diminished CD45RO cells rate in lungs of melanoma B16-bearing mice | No | Different focus |
| 2347 | | Yamaguchi et al., 2022 | | | Potential therapeutic targets discovery by transcriptome analysis of an in vitro human gastric signet ring carcinoma model | No | Different focus |
| 2348 | | Kawaguchi et al., 2022 | | | Naringenin potentiates anti-tumor immunity against oral cancer by inducing lymph node CD169-positive macrophage activation and cytotoxic T cell infiltration | No | Different focus |
| 2349 | | Kumari et al., 2022 | | | Unboxing the molecular modalities of mutagens in cancer | No | Different focus |
| 2350 | | Ren et al., 2022 | | | Crosstalk between the B7/CD28 and EGFR pathways: Mechanisms and therapeutic opportunities | No | Different focus |
| 2351 | | Zhang et al., 2022 | | | Efficacy, safety, and predictors of combined fruquintinib with programmed death-1 inhibitors for advanced microsatellite-stable colorectal cancer: A retrospective study | Yes | Relevant to colorectal cancer immunotherapy |
| 2352 | | van Geffen et al., 2022 | | | Pharmacological modulation of myeloid-derived suppressor cells to dampen inflammation | No | Different focus |
| 2353 | | Talaat et al., 2022 | | | Checkpoint molecules on infiltrating immune cells in colorectal tumor microenvironment | Yes | Relevant to colorectal cancer immunotherapy |
| 2354 | | Rizzo et al., 2022 | | | IL-8 and its role as a potential biomarker of resistance to anti-angiogenic agents and immune checkpoint inhibitors in metastatic renal cell carcinoma | No | Different focus |
| 2355 | | Mo et al., 2022 | | | Progress on structural modification of Tetrandrine with wide range of pharmacological activities | No | Different focus |
| 2356 | | Wang et al., 2022 | | | The immune-related role of beta-2-microglobulin in melanoma | No | Different focus |
| 2357 | | Liu et al., 2022 | | | Single-cell RNA sequencing reveals the cellular and molecular changes that contribute to the progression of lung adenocarcinoma | No | Different focus |
| 2358 | | Lu et al., 2022 | | | New insights into natural products that target the gut microbiota: Effects on the prevention and treatment of colorectal cancer | Yes | Relevant to colorectal cancer treatment |
| 2359 | | Wang et al., 2022 | | | Alterations in DNA damage response and repair genes as potential biomarkers for immune checkpoint blockade in gastrointestinal cancer | Yes | Relevant to immunotherapy in gastrointestinal cancer |
| 2360 | | Sashankh et al., 2022 | | | Synthesis, in silico and in vitro studies of piperazinyl thiourea derivatives as apoptosis inducer for the treatment of colorectal carcinoma | Yes | Relevant to colorectal cancer treatment |
| 2361 | | Yang et al., 2022 | | | Hyperthermia combined with immune checkpoint inhibitor therapy in the treatment of primary and metastatic tumors | Yes | Relevant to immunotherapy in cancer |
| 2362 | | Huang et al., 2022 | | | A Retrospective Study from a Single Center to Identify Hematological Factors that Distinguish between Patients with Colorectal Carcinoma and Colorectal Adenoma | No | Different focus |
| 2363 | | Gan et al., 2022 | | | An anti-CTLA-4 heavy chain-only antibody with enhanced Treg depletion shows excellent preclinical efficacy and safety profile | Yes | Relevant to cancer immunotherapy |
| 2364 | | Zhang et al., 2022 | | | Chemotherapy reinforces anti-tumor immune response and enhances clinical efficacy of immune checkpoint inhibitors | No | - |
| 2365 | | Zhou et al., 2022 | | | Microbiome in cancer: An exploration of carcinogenesis, immune responses and immunotherapy | No | - |
| 2366 | | Jia et al., 2022 | | | Colorectal cancer vaccines: The current scenario and future prospects | No | - |
| 2367 | | Shen et al., 2022 | | | Neoantigen vaccine and neoantigen-specific cell adoptive transfer therapy in solid tumors: Challenges and future directions | No | - |
| 2368 | | Kakati et al., 2022 | | | Immunotherapy for metastatic liver disease from colorectal carcinoma: case series from the Middle East | No | - |
| 2369 | | Wang et al., 2022 | | | Polypharmacology: Principles and methodologies | No | Irrelevant |
| 2370 | | Reddy et al., 2022 | | | Antibody Diversity in Cancer: Translational Implications and Beyond | No | - |
| 2371 | | Panagiotou et al., 2022 | | | CD24: A Novel Target for Cancer Immunotherapy | No | - |
| 2372 | | Yu et al., 2022 | | | When Natural Compounds Meet Nanotechnology: Nature-Inspired Nanomedicines for Cancer Immunotherapy | No | Irrelevant |
| 2373 | | Herold et al., 2022 | | | Patients with Metachronous Peritoneal Metastatic Mucinous Colorectal Adenocarcinoma Benefit More from Cytoreductive Surgery (CRS) and Hyperthermic Intraperitoneal Chemotherapy (HIPEC) than Their Synchronous Counterparts | No | Irrelevant |
| 2374 | | Grzybowski et al., 2022 | | | OATD-02 Validates the Benefits of Pharmacological Inhibition of Arginase 1 and 2 in Cancer | No | Irrelevant |
| 2375 | | Peterson et al., 2022 | | | Recent Advances and Challenges in Cancer Immunotherapy | No | - |
| 2376 | | Eismann et al., 2022 | | | Identification of the Tumor Infiltrating Lymphocytes (TILs) Landscape in Pure Squamous Cell Carcinoma of the Bladder | No | Irrelevant |
| 2377 | | Islas et al., 2022 | | | Cancer Stem Cells in Tumor Microenvironment of Adenocarcinoma of the Stomach, Colon, and Rectum | No | - |
| 2378 | | Damato et al., 2022 | | | New Potential Immune Biomarkers in the Era of Precision Medicine: Lights and Shadows in Colorectal Cancer | No | - |
| 2379 | | Sarhadi et al., 2022 | | | Molecular Biomarkers in Cancer | No | - |
| 2380 | | Kato et al., 2022 | | | Targeting KRAS: Crossroads of Signaling and Immune Inhibition | No | Irrelevant |
| 2381 | | Vaghjiani et al., 2022 | | | Tertiary Lymphoid Structures as Mediators of Immunotherapy Response | No | - |
| 2382 | | Favier et al., 2022 | | | The Role of Immunohistochemistry Markers in Endometrial Cancer with Mismatch Repair Deficiency: A Systematic Review | No | Irrelevant |
| 2383 | | Phelps et al., 2022 | | | Detection of Microsatellite Instability in Colonoscopic Biopsies and Postal Urine Samples from Lynch Syndrome Cancer Patients Using a Multiplex PCR Assay | No | Irrelevant |
| 2384 | | Torres-Jiménez et al., 2022 | | | Precision Medicine in Metastatic Colorectal Cancer: Targeting ERBB2 (HER-2) Oncogene | No | - |
| 2385 | | Oriuchi et al., 2022 | | | Monitoring of Current Cancer Therapy by Positron Emission Tomography and Possible Role of Radiomics Assessment | No | - |
| 2386 | | Keogh et al., 2022 | | | Emerging Biomarkers and the Changing Landscape of Small Cell Lung Cancer | No | Irrelevant |
| 2387 | | Papait et al., 2022 | | | Fight the Cancer, Hit the CAF! | No | Irrelevant |
| 2388 | | Dwivedi et al., 2022 | | | Implications of regulatory T cells in anti-cancer immunity: from pathogenesis to therapeutics | No | - |
| 2389 | | Vitorino et al., 2022 | | | Local Breast Microbiota: A “New” Player on the Block | No | Irrelevant |
| 2390 | | Amato et al., 2022 | | | Microsatellite Instability: From the Implementation of the Detection to a Prognostic and Predictive Role in Cancers | No | - |
| 2391 | | Wagner et al., 2022 | | | Peroxisome Proliferator-Activated Receptors and the Hallmarks of Cancer | No | Irrelevant |
| 2392 | | Chocarro et al., 2022 | | | Cutting-Edge: Preclinical and Clinical Development of the First Approved Lag-3 Inhibitor | No | Irrelevant |
| 2393 | | Yoo et al., 2022 | | | LIGHT (TNFSF14) Costimulation Enhances Myeloid Cell Activation and Antitumor Immunity in the Setting of PD-1/PD-L1 and TIGIT Checkpoint Blockade | No | Irrelevant |
| 2394 | | Chen et al., 2022 | | | Modeling the effect of gut microbiome on therapeutic efficacy of immune checkpoint inhibitors against cancer | No | Irrelevant |
| 2395 | | Wu et al., 2022 | | | The emerging era of personalized medicine in advanced colorectal cancer | No | - |
| 2396 | | Lefler et al., 2022 | | | Immune checkpoint inhibitors in luminal gastrointestinal malignancies: Going beyond MSI-H/dMMR, TMB and PD-L1 | No | - |
| 2397 | | Kourie et al., 2022 | | | The first Middle East and North Africa expert consensus recommendations for the management of advanced colorectal cancer | No | - |
| 2398 | | Sagnella et al., 2022 | | | Locoregional delivery of CAR-T cells in the clinic | No | Irrelevant |
| 2399 | | Gao et al., 2022 | | | Efficacy and safety of HER2-targeted inhibitors for metastatic colorectal cancer with HER2-amplified: A meta-analysis | No | - |
| 2400 | | Martin et al., 2022 | | | Bladder cancer, inflammageing and microbiomes | No | Irrelevant |
| 2401 | | Bhat et al., 2022 | | | Cytokine- and chemokine-induced inflammatory colorectal tumor microenvironment: Emerging avenue for targeted therapy | No | - |
| 2402 | | Liu et al., 2022 | | | Systematic pan-cancer analysis of mutation–treatment interactions using large real-world clinicogenomics data | No | Irrelevant |
| 2403 | | Wang et al., 2022 | | | CRIP1 suppresses BBOX1-mediated carnitine metabolism to promote stemness in hepatocellular carcinoma | No | Irrelevant |
| 2404 | | Blaye et al., 2022 | | | An immunological signature to predict outcome in patients with triple-negative breast cancer with residual disease after neoadjuvant chemotherapy | No | Irrelevant |
| 2405 | | Onoyama et al., 2022 | | | Gastric cancer and genomics: review of literature | No | Irrelevant |
| 2406 | | Kasichayanula et al., 2022 | | | Evolution of preclinical characterization and insights into clinical pharmacology of checkpoint inhibitors approved for cancer immunotherapy | No | - |
| 2407 | | Mishra et al., 2022 | | | Hypoxia-driven metabolic heterogeneity and immune evasive behaviour of gastrointestinal cancers: Elements of a recipe for disaster | No | Irrelevant |
| 2408 | | Tooley et al., 2022 | | | Spatial determinants of CD8+ T cell differentiation in cancer | No | - |
| 2409 | | Ali et al., 2022 | | | Gut microbiota: Role and Association with Tumorigenesis in Different Malignancies | No | - |
| 2410 | | Callesen et al., 2022 | | | Circulating tumour DNA and its clinical utility in predicting treatment response or survival in patients with metastatic colorectal cancer: a systematic review and meta-analysis | No | - |
| 2411 | | Fernandes et al., 2022 | | | The importance of MRI for rectal cancer evaluation | No | Irrelevant |
| 2412 | | Schegoleva et al., 2022 | | | Metastasis prevention: targeting causes and roots | No | - |
| 2413 | | Lin et al., 2022 | | | Targeting RAS in neuroblastoma: Is it possible? | No | Irrelevant |
| 2414 | | Alawawdeh et al., 2022 | | | Regorafenib outcomes from the population based South Australian Metastatic Colorectal Cancer Registry | No | - |
| 2415 | | Hassanian et al., 2022 | | | The expression pattern of Immune checkpoints after chemo/radiotherapy in the tumor microenvironment | No | - |
| 2416 | | Jiang et al., 2022 | | | Potential association factors for developing effective peptide-based cancer vaccines | No | - |
| 2417 | | Scanlan et al., 2022 | | | Herpes simplex virus 1 as an oncolytic viral therapy for refractory cancers | No | - |
| 2418 | | Avery et al., 2022 | | | Onco-immunomodulatory properties of pharmacological interference with RAS-RAF-MEK-ERK pathway hyperactivation | No | Irrelevant |
| 2419 | | Jogalekar et al., 2022 | | | CAR T-Cell-Based gene therapy for cancers: new perspectives, challenges, and clinical developments | No | - |
| 2420 | | Cao et al., 2022 | | | Receptor–ligand pair typing and prognostic risk model for papillary thyroid carcinoma based on single-cell sequencing | No | Irrelevant |
| 2421 | | Chen et al., 2022 | | | A Novel Anti-Cancer Therapy: CRISPR/Cas9 Gene Editing | No | Irrelevant |
| 2422 | | Smith et al., 2022 | | | Tumor microenvironment in pancreatic ductal adenocarcinoma: Implications in immunotherapy | No | Irrelevant |
| 2423 | | Groeneveldt et al., 2022 | | | Preinduced reovirus-specific T-cell immunity enhances the anticancer efficacy of reovirus therapy | No | - |
| 2424 | | Zhao et al., 2022 | | | Comprehensive Pan-Cancer Analysis of Senescence With Cancer Prognosis and Immunotherapy | No | Irrelevant |
| 2425 | | Ren et al., 2022 | | | Oncolytic viruses combined with immune checkpoint therapy for colorectal cancer is a promising treatment option | No | - |
| 2426 | | Wolf et al., 2022 | | | Intratumor Heterogeneity and Antitumor Immunity Shape One Another Bidirectionally | No | - |
| 2427 | | Manzoor et al., 2022 | | | Autophagy: A Versatile Player in the Progression of Colorectal Cancer and Drug Resistance | No | - |
| 2428 | | Wang et al., 2022 | | | Low MxA Expression Predicts Better Immunotherapeutic Outcomes in Glioblastoma Patients Receiving Heat Shock Protein Peptide Complex 96 Vaccination | No | Irrelevant |
| 2429 | | Chamma et al., 2022 | | | Activation of STING in the pancreatic tumor microenvironment: A novel therapeutic opportunity | No | Irrelevant |
| 2430 | | Xu et al., 2022 | | | Therapeutic bispecific antibodies against intracellular tumor antigens | No | - |
| 2431 | | Aristin Revilla et al., 2022 | | | Colorectal Cancer-Infiltrating Regulatory T Cells: Functional Heterogeneity, Metabolic Adaptation, and Therapeutic Targeting | No | - |
| 2432 | | Zhu et al., 2022 | | | Computational Characterizing Necroptosis Reveals Implications for Immune Infiltration and Immunotherapy of Hepatocellular Carcinoma | No | Irrelevant |
| 2433 | | Kumar et al., 2022 | | | GM-CSF: A Double-Edged Sword in Cancer Immunotherapy | No | - |
| 2434 | | Muller et al., 2022 | | | Metastatic colorectal cancer: second line treatment in the era of personalized medicine | No | - |
| 2435 | | Chen et al., 2022 | | | N6-Methyladenosine RNA-Binding Protein YTHDF1 in Gastrointestinal Cancers: Function, Molecular Mechanism and Clinical Implication | No | - |
| 2436 | | Boudin et al., 2022 | | | CISH Expression Is Associated with Metastasis-Free Interval in Triple-Negative Breast Cancer and Refines the Prognostic Value of PDL1 Expression | No | Irrelevant |
| 2437 | | Krawczyk et al., 2022 | | | New Genetic Technologies in Diagnosis and Treatment of Cancer of Unknown Primary | No | - |
| 2438 | | Luo et al., 2022 | | | Activation of Stimulation of Interferon Genes (STING) Signal and Cancer Immunotherapy | No | - |
| 2439 | | Adeleke et al., 2022 | | | Microsatellite instability testing in colorectal patients with Lynch syndrome: Lessons learned from a case report and how to avoid such pitfalls | No | Irrelevant |
| 2440 | | Li et al., 2022 | | | Targeting interleukin-17 enhances tumor response to immune checkpoint inhibitors in colorectal cancer | No | - |
| 2441 | | Li et al., 2022 | | | Treatment with a VEGFR-2 antibody results in intra-tumor immune modulation and enhances anti-tumor efficacy of PD-L1 blockade in syngeneic murine tumor models | No | Irrelevant |
| 2442 | | Dong et al., 2022 | | | Preclinical Safety and Biodistribution in Mice Following Single-Dose Intramuscular Inoculation of Tumor DNA Vaccine by Electroporation | No | Irrelevant |
| 2443 | | Niu et al., 2022 | | | High expression of HHLA2 predicts poor prognosis in medullary thyroid carcinoma | No | Irrelevant |
| 2444 | | Franklin et al., 2022 | | | Recruited and Tissue-Resident Natural Killer Cells in the Lung During Infection and Cancer | No | - |
| 2445 | | Xu et al., 2022 | | | Gut Microbiota and Immunotherapy | No | - |
| 2446 | | Cassese et al., 2022 | | | Preoperative Assessment and Perioperative Management of Resectable Gallbladder Cancer in the Era of Precision Medicine and Novel Technologies | No | Irrelevant |
| 2447 | | El-Malah et al., 2022 | | | Selective COX-2 Inhibitors: Road from Success to Controversy and the Quest for Repurposing | No | - |
| 2448 | | Gatto et al., 2022 | | | Hypermutation as a potential predictive biomarker of immunotherapy efficacy in high-grade gliomas: A broken dream? | No | Irrelevant |
| 2449 | | Kovaleva et al., 2022 | | | Macrophage Phenotype in Combination with Tumor Microbiome Composition Predicts RCC Patients’ Survival: A Pilot Study | No | Irrelevant |
| 2450 | | Chiesa et al., 2022 | | | NK Cell-Based Immunotherapy in Colorectal Cancer | No | - |
| 2451 | | Liu et al., 2022 | | | From Intestinal Epithelial Homeostasis to Colorectal Cancer: Autophagy Regulation in Cellular Stress | No | - |
| 2452 | | Song et al., 2022 | | | Mesothelioma Mouse Models with Mixed Genomic States of Chromosome and Microsatellite Instability | No | Irrelevant |
| 2453 | | Papak et al., 2022 | | | What Inhibits Natural Killers’ Performance in Tumour | No | - |
| 2454 | | Qi et al., 2022 | | | Novel Drugs with High Efficacy against Tumor Angiogenesis | No | - |
| 2455 | | Tsang et al., 2022 | | | Development and Characterization of an Anti-Cancer Monoclonal Antibody for Treatment of Human Carcinomas | No | - |
| 2456 | | Ortiz-Morales et al., 2022 | | | Basal VEGF-A and ACE Plasma Levels of Metastatic Colorectal-Cancer Patients Have Prognostic Value for First-Line Treatment with Chemotherapy Plus Bevacizumab | No | - |
| 2457 | | Kim et al., 2022 | | | Adaptive immune resistance at the tumour site: mechanisms and therapeutic opportunities | No | - |
| 2458 | | Malhab et al., 2022 | | | Obesity and Inflammation: Colorectal Cancer Engines | No | - |
| 2459 | | He et al., 2022 | | | mRNA cancer vaccines: Advances, trends and challenges | No | - |
| 2460 | | Delaye et al., 2022 | | | Rational testing for gene fusion in colorectal cancer: MSI and RAS-BRAF wild-type metastatic colorectal cancer as target population for systematic screening | No | - |
| 2461 | | Rossini et al., 2022 | | | Treatments after second progression in metastatic colorectal cancer: A pooled analysis of the TRIBE and TRIBE2 studies | No | - |
| 2462 | | Feng et al., 2022 | | | Identification of Candidate Antigens and Immune Subtypes in Colon Cancer for mRNA Vaccine Development | No | - |
| 2463 | | Mjahed et al., 2022 | | | Where Are We Now and Where Might We Be Headed in Understanding and Managing Brain Metastases in Colorectal Cancer Patients? | No | - |
| 2464 | | Zhang et al., 2022 | | | Ribavirin inhibits cell proliferation and metastasis and prolongs survival in soft tissue sarcomas by downregulating both protein arginine methyltransferases 1 and 5 | No | Irrelevant |
| 2465 | | Li et al., 2022 | | | A novel multifunctional anti-PD-L1-CD16a-IL15 induces potent cancer cell killing in PD-L1-positive tumour cells | No | Irrelevant |
| 2466 | | Wei et al., 2022 | | | PKCα/ZFP64/CSF1 axis resets the tumor microenvironment and fuels anti-PD1 resistance in hepatocellular carcinoma | No | Irrelevant |
| 2467 | | Ciardiello et al., 2022 | | | Clinical management of metastatic colorectal cancer in the era of precision medicine | No | - |
| 2468 | | Fridman et al., 2022 | | | B cells and tertiary lymphoid structures as determinants of tumour immune contexture and clinical outcome | No | - |
| 2469 | | Glaire et al., 2022 | | | Discordant prognosis of mismatch repair deficiency in colorectal and endometrial cancer reflects variation in antitumour immune response and immune escape | No | Irrelevant |
| 2470 | | Sorieul et al., 2022 | | | Recent advances and future perspectives on carbohydrate-based cancer vaccines and therapeutics | No | - |
| 2471 | | Siesing et al., 2022 | | | Delineating the intra-patient heterogeneity of molecular alterations in treatment-naïve colorectal cancer with peritoneal carcinomatosis | No | - |
| 2472 | | de Wit et al., 2022 | | | Exposure-response relationship of ramucirumab in RANGE, a randomized phase III trial in advanced urothelial carcinoma refractory to platinum therapy | No | Irrelevant |
| 2473 | | Joshi et al., 2022 | | | Targeting myeloid-derived suppressor cells to enhance natural killer cell-based immunotherapy | No | - |
| 2474 | | Sedky et al., 2022 | | | Insights into the therapeutic potential of histone deacetylase inhibitor/immunotherapy combination regimens in solid tumors | No | - |
| 2475 | | Bourhis et al., 2022 | | | Rapid Idylla™ mutational testing: Current and future theranostic applications | No | Irrelevant |
| 2476 | | Chang et al., 2022 | | | SWI/SNF Complex-deficient Undifferentiated Carcinoma of the Gastrointestinal Tract | No | Irrelevant |
| 2477 | | Liu et al., 2022 | | | Targeting the immune checkpoint B7-H3 for next-generation cancer immunotherapy | No | - |
| 2478 | | Li et al., 2022 | | | Liposomal Co-delivery of PD-L1 siRNA/Anemoside B4 for Enhanced Combinational Immunotherapeutic Effect | No | Irrelevant |
| 2479 | | Ceccon et al., 2022 | | | Microsatellite Instable Colorectal Adenocarcinoma Diagnostics: The Advent of Liquid Biopsy Approaches | No | - |
| 2480 | | Manafi-Farid et al., 2022 | | | ImmunoPET: Antibody-Based PET Imaging in Solid Tumors | No | - |
| 2481 | | Muniyan et al., 2022 | | | Macrophage inhibitory cytokine-1 in cancer: Beyond the cellular phenotype | No | - |
| 2482 | | Conner et al., 2022 | | | Emergence of the CD226 Axis in Cancer Immunotherapy | No | - |
| 2483 | | Puccini et al., 2022 | | | Molecular profiling of signet-ring-cell carcinoma (SRCC) from the stomach and colon reveals potential new therapeutic targets | No | - |
| 2484 | | Westheim et al., 2022 | | | Fatty Acids as a Tool to Boost Cancer Immunotherapy Efficacy | No | Irrelevant |
| 2485 | | Emran et al., 2022 | | | Multidrug Resistance in Cancer: Understanding Molecular Mechanisms, Immunoprevention and Therapeutic Approaches | No | Irrelevant |
| 2486 | | Guo et al., 2022 | | | The Emerging Roles of Human Gut Microbiota in Gastrointestinal Cancer | No | Irrelevant |
| 2487 | | Lanuza et al., 2022 | | | Adoptive NK Cell Transfer as a Treatment in Colorectal Cancer Patients: Analyses of Tumour Cell Determinants Correlating With Efficacy In Vitro and In Vivo | No | Irrelevant |
| 2488 | | Xu et al., 2022 | | | Novel Hypoxia-Associated Gene Signature Depicts Tumor Immune Microenvironment and Predicts Prognosis of Colon Cancer Patients | No | Irrelevant |
| 2489 | | Zhang et al., 2022 | | | Neoantigens in Precision Cancer Immunotherapy: From Identification to Clinical Applications | No | Irrelevant |
| 2490 | | Li et al., 2022 | | | Oncolytic Viruses: Immunotherapy Drugs for Gastrointestinal Malignant Tumors | No | Irrelevant |
| 2491 | | Nong et al., 2022 | | | Tumor Immunotherapy: Mechanisms and Clinical Applications | No | Irrelevant |
| 2492 | | Yancen et al., 2022 | | | Analysis of Potential Resistance Mechanism of Liver Cancer Immunotherapy | No | Irrelevant |
| 2493 | | Chocarro et al., 2022 | | | Clinical Landscape of LAG-3-Targeted Therapy | No | Irrelevant |
| 2494 | | Feng et al., 2022 | | | Intestinal Stents: Structure, Functionalization and Advanced Engineering Innovation | No | Irrelevant |
| 2495 | | Tanaka et al., 2022 | | | Defucosylated Anti-Epidermal Growth Factor Receptor Monoclonal Antibody Exerted Antitumor Activities in Mouse Xenograft Models of Canine Mammary Gland Tumor | No | Irrelevant |
| 2496 | | Hernandez-Sanchez et al., 2022 | | | Vaccines for Immunoprevention of DNA Mismatch Repair Deficient Cancers | No | Irrelevant |
| 2497 | | Koustas et al., 2022 | | | Immunotherapy as a Therapeutic Strategy for Gastrointestinal Cancer—Current Treatment Options and Future Perspectives | No | Irrelevant |
| 2498 | | Alcazer et al., 2022 | | | Panel Informativity Optimizer: An R Package to Improve Cancer Next-Generation Sequencing Panel Informativity | No | Irrelevant |
| 2499 | | Wang et al., 2022 | | | Drug Resistance in Colorectal Cancer: From Mechanism to Clinic | No | Irrelevant |
| 2500 | | Gupta et al., 2022 | | | Implications of Gut Microbiota in Epithelial–Mesenchymal Transition and Cancer Progression: A Concise Review | No | Irrelevant |
| 2501 | | Ganini et al., 2022 | | | No Time to Die: How Kidney Cancer Evades Cell Death | No | Irrelevant |
| 2502 | | Jiang et al., 2022 | | | Different Subpopulations of Regulatory T Cells in Human Autoimmune Disease, Transplantation, and Tumor Immunity | No | Irrelevant |
| 2503 | | Li et al., 2022 | | | Analysis of Interactions of Immune Checkpoint Inhibitors with Antibiotics in Cancer Therapy | No | Irrelevant |
| 2504 | | Liao et al., 2022 | | | Correlation of KMT2 Family Mutations with Molecular Characteristics and Prognosis in Colorectal Cancer | No | Irrelevant |
| 2505 | | Gouasmi et al., 2022 | | | The Kynurenine Pathway and Cancer: Why Keep It Simple When You Can Make It Complicated | No | Irrelevant |
| 2506 | | Alam et al., 2022 | | | Recent Applications of Artificial Intelligence from Histopathologic Image-Based Prediction of Microsatellite Instability in Solid Cancers: A Systematic Review | No | Irrelevant |
| 2507 | | Mukherji et al., 2022 | | | Immunotherapy for Colorectal Cancer | No | Irrelevant |
| 2508 | | Malla et al., 2022 | | | Evolving Role of Circulating Tumor DNA and Emerging Targeted Therapy in Colorectal Cancer | No | Irrelevant |
| 2509 | | Cunha et al., 2022 | | | Translating the Immune Microenvironment of Thyroid Cancer into Clinical Practice | No | Irrelevant |
| 2510 | | Mudassar et al., 2022 | | | Improving the Synergistic Combination of PD-1/PD-L1 Blockade and Radiotherapy by Targeting the Hypoxic Tumor Microenvironment | No | Irrelevant |
| 2511 | | Wyvekens et al., 2022 | | | Histopathological and Genetic Features of Mismatch Repair-Deficient High-Grade Prostate Cancer | No | Irrelevant |
| 2512 | | Klapholz et al., 2022 | | | Presence of Tim3+ and PD-1+ CD8+ T Cells Identifies Microsatellite Stable Colorectal Carcinomas with Immune Exhaustion | No | Irrelevant |
| 2513 | | Qiao et al., 2022 | | | Correlation of Mismatch Repair Deficiency with Clinicopathological Features and PD-L1 Expression in Thyroid Carcinoma | No | Irrelevant |
| 2514 | | Sia et al., 2022 | | | Basic Cancer Immunology for Radiation Oncologists | No | Irrelevant |
| 2515 | | Mahmud et al., 2022 | | | Mechanistic Insights into the Interplays Between Neutrophils and Other Immune Cells in Cancer Development and Progression | No | Irrelevant |
| 2516 | | Mylod et al., 2022 | | | Natural Killer Cell Therapy: A New Frontier for Obesity-Associated Cancer | No | Irrelevant |
| 2517 | | Kisoda et al., 2022 | | | The Role of Partial-EMT in the Progression of Head and Neck Squamous Cell Carcinoma | No | Irrelevant |
| 2518 | | Nikas et al., 2022 | | | Biomarkers Expression Among Paired Serous Ovarian Cancer Primary Lesions and Their Peritoneal Cavity Metastases | No | Irrelevant |
| 2519 | | de Boo et al., 2022 | | | Adjuvant Capecitabine-Containing Chemotherapy Benefit and Homologous Recombination Deficiency in Early-Stage Triple-Negative Breast Cancer Patients | No | Irrelevant |
| 2520 | | Zong et al., 2022 | | | Expression of B7 Family Checkpoint Proteins in Cervical Cancer | No | Irrelevant |
| 2521 | | Chung, C., 2022 | | | Predictive and Prognostic Biomarkers with Therapeutic Targets in Colorectal Cancer: A 2021 Update on Current Development, Evidence, and Recommendation | No | Irrelevant |
| 2522 | | Fancellu et al., 2022 | | | Red Blood Cell Distribution Width (RDW) Correlates to the Anatomical Location of Colorectal Cancer. Implications for Clinical Use | No | Irrelevant |
| 2523 | | Xin et al., 2022 | | | The Prognostic Signature and Therapeutic Value of Phagocytic Regulatory Factors in Prostate Adenocarcinoma (PRAD) | No | Irrelevant |
| 2524 | | Bai et al., 2022 | | | Exploration of Different Hypoxia Patterns and Construction of a Hypoxia-Related Gene Prognostic Index in Colorectal Cancer | No | Irrelevant |
| 2525 | | Chen et al., 2022 | | | OLFM4 Deficiency Delays the Progression of Colitis to Colorectal Cancer by Abrogating PMN-MDSCs Recruitment | No | Irrelevant |
| 2526 | | Busà et al., 2022 | | | Tissue-Resident Innate Immune Cell-Based Therapy: A Cornerstone of Immunotherapy Strategies for Cancer Treatment | No | Irrelevant |
| 2527 | | Kim & Cho, 2022 | | | The Evasion Mechanisms of Cancer Immunity and Drug Intervention in the Tumor Microenvironment | No | Irrelevant |
| 2528 | | Hsieh et al., 2022 | | | An Observational Study of Trifluridine/Tipiracil-Containing Regimen Versus Regorafenib-Containing Regimen in Patients With Metastatic Colorectal Cancer | No | Irrelevant |
| 2529 | | Seliger & Massa, 2022 | | | Modulation of Lymphocyte Functions in the Microenvironment by Tumor Oncogenic Pathways | No | Irrelevant |
| 2530 | | Obata-Ninomiya et al., 2022 | | | Emerging Role for Thymic Stromal Lymphopoietin-Responsive Regulatory T Cells in Colorectal Cancer Progression in Humans and Mice | No | Irrelevant |
| 2531 | | Annese et al., 2022 | | | Update in TIGIT Immune-Checkpoint Role in Cancer | No | Irrelevant |
| 2532 | | Wong et al., 2022 | | | Intranasal Delivery of Recombinant S100A8 Protein Delays Lung Cancer Growth by Remodeling the Lung Immune Microenvironment | No | Irrelevant |
| 2533 | | Dai et al., 2022 | | | Pancreatic Cancer: Nucleic Acid Drug Discovery and Targeted Therapy | No | Irrelevant |
| 2534 | | Chida et al., 2022 | | | Transcriptomic Profiling of MSI-H/dMMR Gastrointestinal Tumors to Identify Determinants of Responsiveness to Anti–PD-1 Therapy | No | Irrelevant |
| 2535 | | Min et al., 2022 | | | Therapeutic Effects of Cold Atmospheric Plasma on Solid Tumor | No | Irrelevant |
| 2536 | | Russo et al., 2022 | | | Microsatellite Instability Evaluation of Patients with Solid Tumor: Routine Practice Insight from a Large Series of Italian Referral Centre | No | Irrelevant |
| 2537 | | Safarzadeh Kozani et al., 2022 | | | Recent Advances in Solid Tumor CAR-T Cell Therapy: Driving Tumor Cells From Hero to Zero? | No | Irrelevant |
| 2538 | | Ni et al., 2022 | | | Advances in Human Dendritic Cell-Based Immunotherapy Against Gastrointestinal Cancer | No | Irrelevant |
| 2539 | | Abdel Sater et al., 2022 | | | From Tumor Cells to Endothelium and Gut Microbiome: A Complex Interaction Favoring the Metastasis Cascade | No | Irrelevant |
| 2540 | | Dong et al., 2022 | | | Can Natural Products be Used to Overcome the Limitations of Colorectal Cancer Immunotherapy? | No | Irrelevant |
| 2541 | | Song et al., 2022 | | | Albumin Nanoparticle Containing a PI3Kγ Inhibitor and Paclitaxel in Combination with α-PD1 Induces Tumor Remission of Breast Cancer in Mice | No | Irrelevant |
| 2542 | | Ding et al., 2022 | | | Lysine Acetylation/Deacetylation Modification of Immune-Related Molecules in Cancer Immunotherapy | No | Irrelevant |
| 2543 | | Karan et al., 2022 | | | Human Epidermal Growth Factor Receptor 2-Targeting Approaches for Colorectal Cancer: Clinical Implications of Novel Treatments and Future Therapeutic Avenues | No | Irrelevant |
| 2544 | | Madison et al., 2022 | | | Clustered 8-Oxo-Guanine Mutations and Oncogenic Gene Fusions in Microsatellite-Unstable Colorectal Cancer | No | Irrelevant |
| 2545 | | Rostamizadeh et al., 2022 | | | Recent Advances in Cancer Immunotherapy: Modulation of Tumor Microenvironment by Toll-like Receptor Ligands | No | Irrelevant |
| 2546 | | Morgado et al., 2022 | | | Targeted Therapy for Metastatic Colorectal Cancer: What Do We Currently Have in Clinical Practice? | No | Irrelevant |
| 2547 | | Wang et al., 2022 | | | Challenges and the Evolving Landscape of Assessing Blood-Based PD-L1 Expression as a Biomarker for Anti-PD-(L)1 Immunotherapy | No | Irrelevant |
| 2548 | | Shin et al., 2022 | | | Bispecific Antibody-Based Immune-Cell Engagers and Their Emerging Therapeutic Targets in Cancer Immunotherapy | No | Irrelevant |
| 2549 | | Voutsadakis, 2022 | | | Cell Line Models for Drug Discovery in PIK3CA-Mutated Colorectal Cancers | No | Irrelevant |
| 2550 | | Rosati et al., 2022 | | | Colorectal Cancer Heterogeneity and the Impact on Precision Medicine and Therapy Efficacy | No | Irrelevant |
| 2551 | | Kim et al., 2022 | | | Immune Profile of BRAF-Mutated Metastatic Colorectal Tumors with Good Prognosis After Palliative Chemotherapy | No | Irrelevant |
| 2552 | | Kiwaki et al., 2022 | | | Patient-Derived Organoids of Colorectal Cancer: A Useful Tool for Personalized Medicine | No | Irrelevant |
| 2553 | | Országhová et al., 2022 | | | Overcoming Chemotherapy Resistance in Germ Cell Tumors | No | Irrelevant |
| 2554 | | Acosta et al., 2022 | | | Therapeutic Impact and Routine Application of Next-Generation Sequencing: A Single Institute Study | No | Irrelevant |
| 2555 | | Zhou et al., 2022 | | | Engineering-Induced Pluripotent Stem Cells for Cancer Immunotherapy | No | Irrelevant |
| 2556 | | Huyghe et al., 2022 | | | Biomarkers of Response and Resistance to Immunotherapy in Microsatellite Stable Colorectal Cancer: Toward a New Personalized Medicine | No | Irrelevant |
| 2557 | | Holterhus et al., 2022 | | | The Cellular Tumor Immune Microenvironment of Childhood Solid Cancers: Informing More Effective Immunotherapies | No | Irrelevant |
| 2558 | | Orzołek et al., 2022 | | | Estrogens, Cancer and Immunity | No | Irrelevant |
| 2559 | | Minami et al., 2022 | | | How Compatible Are Immune Checkpoint Inhibitors and Thermal Ablation for Liver Metastases? | No | Irrelevant |
| 2560 | | Valério-Fernandes et al., 2022 | | | Nucleolin Overexpression Predicts Patient Prognosis While Providing a Framework for Targeted Therapeutic Intervention in Lung Cancer | No | Irrelevant |
| 2561 | | Szlasa et al., 2022 | | | Prognostic and Therapeutic Role of CD15 and CD15s in Cancer | No | Irrelevant |
| 2562 | | El Zarif et al., 2022 | | | Overcoming Therapy Resistance in Colon Cancer by Drug Repurposing | No | Irrelevant |
| 2563 | | Huang et al., 2022 | | | Prognostic Significance of Baseline Neutrophil-Lymphocyte Ratio in Patients with Non-Small-Cell Lung Cancer: A Pooled Analysis of Open Phase III Clinical Trial Data | No | Irrelevant |
| 2564 | | Cejalvo et al., 2022 | | | Oncolytic Viruses: A New Immunotherapeutic Approach for Breast Cancer Treatment? | No | Irrelevant |
| 2565 | | Ying et al., 2022 | | | Human Endogenous Retrovirus-H Long Terminal Repeat-Associating 2: The Next Immune Checkpoint for Antitumor Therapy | No | Irrelevant |
| 2566 | | Mahabady et al., 2022 | | | Noncoding RNAs and Their Therapeutics in Paclitaxel Chemotherapy: Mechanisms of Initiation, Progression, and Drug Sensitivity | No | Irrelevant |
| 2567 | | Haag et al., 2022 | | | Pembrolizumab and Maraviroc in Refractory Mismatch Repair Proficient/Microsatellite-Stable Metastatic Colorectal Cancer – The PICCASSO Phase I Trial | No | Irrelevant |
| 2568 | | Nannini et al., 2022 | | | Immune-Related Aseptic Meningitis and Strategies to Manage Immune Checkpoint Inhibitor Therapy: A Systematic Review | No | Irrelevant |
| 2569 | | Cui, 2022 | | | Towards a Precision Immune Checkpoint Blockade Immunotherapy in Patients with Colorectal Cancer: Strategies and Perspectives | No | Irrelevant |
| 2570 | | Fritah et al., 2022 | | | The Current Clinical Landscape of Personalized Cancer Vaccines | No | Irrelevant |
| 2571 | | Boukouris et al., 2022 | | | Latest Evidence on Immune Checkpoint Inhibitors in Metastatic Colorectal Cancer: A 2022 Update | No | Irrelevant |
| 2572 | | Carroll et al., 2022 | | | Liver Immunology, Immunotherapy, and Liver Cancers: Time for a Rethink? | No | Irrelevant |
| 2573 | | Niu et al., 2022 | | | Arginase: An Emerging and Promising Therapeutic Target for Cancer Treatment | No | Irrelevant |
| 2574 | | Sager et al., 2022 | | | Therapeutic Potential of CDK4/6 Inhibitors in Renal Cell Carcinoma | No | Irrelevant |
| 2575 | | Cass et al., 2022 | | | The Microbiome: The Link to Colorectal Cancer and Research Opportunities | No | Irrelevant |
| 2576 | | Strickler et al., 2022 | | | Diagnosis and Treatment of ERBB2-Positive Metastatic Colorectal Cancer: A Review | No | Irrelevant |
| 2577 | | Christou et al., 2022 | | | Impact of Diabetes and Metformin Use on Recurrence and Outcome in Stage II–III Colon Cancer Patients—A Pooled Analysis of Three Adjuvant Trials | No | Irrelevant |
| 2578 | | Ghauri et al., 2022 | | | Mechanistic Insights Expatiating the Biological Role and Regulatory Implications of Estrogen and HER2 in Breast Cancer Metastasis | No | Irrelevant |
| 2579 | | Ahadi et al., 2022 | | | SWI/SNF Complex (SMARCA4, SMARCA2, INI1/SMARCB1)-Deficient Colorectal Carcinomas Are Strongly Associated with Microsatellite Instability: An Incidence Study in 4508 Colorectal Carcinomas | No | Irrelevant |
| 2580 | | Lohinai et al., 2022 | | | Loss of STING Expression is Prognostic in Non–Small Cell Lung Cancer | No | Irrelevant |
| 2581 | | Li et al., 2022 | | | In Vivo and In Vitro Inhibition of SCLC by Combining Dual Cancer-Specific Recombinant Adenovirus with Etoposide | No | Irrelevant |
| 2582 | | Zhao et al., 2022 | | | The Crohn’s-Like Lymphoid Reaction Density: A New Artificial Intelligence Quantified Prognostic Immune Index in Colon Cancer | No | Irrelevant |
| 2583 | | Buchler, 2022 | | | Microsatellite Instability and Metastatic Colorectal Cancer – A Clinical Perspective | No | Irrelevant |
| 2584 | | Wang et al., 2022 | | | Identification of a Novel Immune Landscape Signature for Predicting Prognosis and Response of Colon Cancer to Immunotherapy | No | Irrelevant |
| 2585 | | Xiao et al., 2022 | | | Integrative Analysis Constructs an Extracellular Matrix-Associated Gene Signature for the Prediction of Survival and Tumor Immunity in Lung Adenocarcinoma | No | Irrelevant |
| 2586 | | Zhou et al., 2022 | | | Immunogenicity Assessment of Bispecific Antibody-Based Immunotherapy in Oncology | No | Irrelevant |
| 2587 | | Feng et al., 2022 | | | Tumor Microenvironment Profiling Identifies Prognostic Signatures and Suggests Immunotherapeutic Benefits in Neuroblastoma | No | Irrelevant |
| 2588 | | Jeon et al., 2022 | | | Development of Potent Immune Modulators Targeting Stimulator of Interferon Genes Receptor | No | Irrelevant |
| 2589 | | Zheng et al., 2022 | | | Sustained Drug Release from Liposomes for the Remodeling of Systemic Immune Homeostasis and the Tumor Microenvironment | No | Irrelevant |
| 2590 | | Chakravarty et al., 2022 | | | Somatic Genomic Testing in Patients with Metastatic or Advanced Cancer: ASCO Provisional Clinical Opinion | No | Irrelevant |
| 2591 | | Cao & Qiu, 2022 | | | Research Progress and Hotspots of Immunotherapy for Microsatellite Stable Colorectal Cancer | No | Irrelevant |
| 2592 | | Zannoni et al., 2022 | | | Current Prognostic and Predictive Biomarkers for Endometrial Cancer in Clinical Practice: Recommendations from the Italian Study Group | No | Irrelevant |
| 2593 | | Lin et al., 2022 | | | Does the Microbiota Composition Influence the Efficacy of Colorectal Cancer Immunotherapy? | No | Irrelevant |
| 2594 | | Huang & Yang, 2022 | | | Molecular Network of Colorectal Cancer and Current Therapeutic Options | No | Irrelevant |
| 2595 | | Vishwakarma et al., 2022 | | | Regulation of Tumor Immune Microenvironment by Sphingolipids and Lysophosphatidic Acid | No | Irrelevant |
| 2596 | | Ray & Mukherjee, 2022 | | | Directing Hypoxic Tumor Microenvironment and HIF to Illuminate Cancer Immunotherapy's Existing Prospects and Challenges in Drug Targets | No | Irrelevant |
| 2597 | | Lemery et al., 2022 | | | Development of Tissue-Agnostic Treatments for Patients with Cancer | No | Irrelevant |
| 2598 | | Paschen et al., 2022 | | | Central Role of the Antigen-Presentation and Interferon-γ Pathways in Resistance to Immune Checkpoint Blockade | No | Irrelevant |
| 2599 | | Balasubramanian et al., 2022 | | | Regulation of the Antigen Presentation Machinery in Cancer and Its Implication for Immune Surveillance | No | Irrelevant |
| 2600 | | Hossain et al., 2022 | | | Colorectal Cancer: A Review of Carcinogenesis, Global Epidemiology, Current Challenges, Risk Factors, Preventive and Treatment Strategies | No | Irrelevant |
| 2601 | | Da Costa & Freire, 2022 | | | Advances in the Immunomodulatory Properties of Glycoantigens in Cancer | No | Irrelevant |
| 2602 | | Antunes et al., 2022 | | | In Vitro Cancer Models: A Closer Look at Limitations on Translation | No | Irrelevant |
| 2603 | | Wu et al., 2022 | | | The Establishment and Experimental Verification of an lncRNA-Derived CD8+ T Cell Infiltration ceRNA Network in Colorectal Cancer | No | Irrelevant |
| 2604 | | Nonomura et al., 2022 | | | Ovarian Endometrioid and Clear Cell Carcinomas with Low Prevalence of Microsatellite Instability: A Unique Subset of Ovarian Carcinomas Could Benefit from Combination Therapy with Immune Checkpoint Inhibitors | No | Irrelevant |
| 2605 | | Park et al., 2022 | | | Targeting the Gut and Tumor Microbiota in Cancer | No | Irrelevant |
| 2606 | | Evrard et al., 2022 | | | Heterogeneity of Mismatch Repair Status and Microsatellite Instability Between Primary Tumor and Metastasis and Its Implications for Immunotherapy in Colorectal Cancers | No | Irrelevant |
| 2607 | | Mauri et al., 2022 | | | Next-Generation Sequencing of Circulating Tumor DNA Can Optimize Second-Line Treatment in RAS Wild-Type Metastatic Colorectal Cancer After Progression on Anti-EGFR Therapy | No | Irrelevant |
| 2608 | | Lokhov et al., 2022 | | | Changing Landscape of Cancer Vaccines—Novel Proteomics Platform for New Antigen Compositions | No | Irrelevant |
| 2609 | | Hong et al., 2022 | | | Neoadjuvant Intratumoral Immunotherapy with TLR9 Activation and Anti-OX40 Antibody Eradicates Metastatic Cancer | No | Irrelevant |
| 2610 | | Hoorn et al., 2022 | | | Clinical Value of Consensus Molecular Subtypes in Colorectal Cancer: A Systematic Review and Meta-Analysis | No | Irrelevant |
| 2611 | | Wang et al., 2022 | | | Deconvolving Clinically Relevant Cellular Immune Cross-Talk from Bulk Gene Expression Using CODEFACS and LIRICS Stratifies Patients with Melanoma to Anti–PD-1 Therapy | No | Irrelevant |
| 2612 | | Pece et al., 2022 | | | Inhibitors of ADAM10 Reduce Hodgkin Lymphoma Cell Growth in 3D Microenvironments and Enhance Brentuximab-Vedotin Effect | No | Irrelevant |
| 2613 | | Pekarek et al., 2022 | | | Clinical Applications of Classical and Novel Biological Markers of Pancreatic Cancer | No | Irrelevant |
| 2614 | | Goïta et al., 2022 | | | Colorectal Cancer: The Contribution of CXCL12 and Its Receptors CXCR4 and CXCR7 | No | Irrelevant |
| 2615 | | de la Haba-Rodriguez et al., 2022 | | | SEOM-GETTHI Clinical Guideline for the Practical Management of Molecular Platforms (2021) | No | Irrelevant |
| 2616 | | Senders & Martin, 2022 | | | Intratumoral Immunotherapy and Tumor Ablation: A Local Approach with Broad Potential | No | Irrelevant |
| 2617 | | Aran et al., 2022 | | | Evaluation of the TCR Repertoire as a Predictive and Prognostic Biomarker in Cancer: Diversity or Clonality? | No | Irrelevant |
| 2618 | | Garnier et al., 2022 | | | Local Ablative Therapy Associated with Immunotherapy in Locally Advanced Pancreatic Cancer | No | Irrelevant |
| 2619 | | Bandaru et al., 2022 | | | Targeting T Regulatory Cells: Their Role in Colorectal Carcinoma Progression and Current Clinical Trials | No | Irrelevant |
| 2620 | | Kuzuya et al., 2022 | | | Clinical Outcomes of Ramucirumab as Post-treatment Following Atezolizumab/Bevacizumab Combination Therapy in Advanced Hepatocellular Carcinoma | No | Irrelevant |
| 2621 | | Deng et al., 2022 | | | PI3K/AKT Signaling Tips the Balance of Cytoskeletal Forces for Cancer Progression | No | Irrelevant |
| 2622 | | Milic et al., 2022 | | | How to Improve SBRT Outcomes in NSCLC: From Pre-Clinical Modeling to Successful Clinical Translation | No | Irrelevant |
| 2623 | | Chen et al., 2022 | | | How to Overcome Tumor Resistance to Anti-PD-1/PD-L1 Therapy by Immunotherapy Modifying the Tumor Microenvironment in MSS CRC | No | Irrelevant |
| 2624 | | Park et al., 2022 | | | Artificial Intelligence–Powered Hematoxylin and Eosin Analyzer Reveals Distinct Immunologic and Mutational Profiles Among Immune Phenotypes in Non–Small-Cell Lung Cancer | No | Irrelevant |
| 2625 | | Angerilli et al., 2022 | | | BRAF-Mutated Colorectal Adenocarcinomas: Pathological Heterogeneity and Clinical Implications | No | Irrelevant |
| 2626 | | Marchalot & Mjösberg, 2022 | | | Innate Lymphoid Cells in Colorectal Cancer | No | Irrelevant |
| 2627 | | Charneau et al., 2022 | | | Development of Antigen-Prediction Algorithm for Personalized Neoantigen Vaccine Using Human Leukocyte Antigen Transgenic Mouse | No | Irrelevant |
| 2628 | | Suwaidan et al., 2022 | | | HER2 Targeted Therapy in Colorectal Cancer: New Horizons | No | Irrelevant |
| 2629 | | Qiao et al., 2022 | | | LIGHT Enhanced Bispecific Antibody Armed T-Cells to Treat Immunotherapy-Resistant Colon Cancer | No | Irrelevant |
| 2630 | | Li et al., 2022 | | | Engineering ROS-Responsive Bioscaffolds for Disrupting Myeloid Cell-Driven Immunosuppressive Niche to Enhance PD-L1 Blockade-Based Postablative Immunotherapy | No | Irrelevant |
| 2631 | | Nomoto et al., 2022 | | | Fusobacterium nucleatum Promotes Esophageal Squamous Cell Carcinoma Progression via the NOD1/RIPK2/NF-κB Pathway | No | Irrelevant |
| 2632 | | Oliveira & Correia, 2022 | | | Clinical Application of Radioiodinated Antibodies: Where Are We? | No | Irrelevant |
| 2633 | | Heinze et al., 2022 | | | Validated Biomarker Assays Confirm That ARID1A Loss is Confounded with MMR Deficiency, CD8+ TIL Infiltration, and Provides No Independent Prognostic Value in Endometriosis-Associated Ovarian Carcinomas | No | Irrelevant |
| 2634 | | Geh et al., 2022 | | | Neutrophils as potential therapeutic targets in hepatocellular carcinoma | No | Irrelevant |
| 2635 | | Papamichail et al., 2022 | | | Minimizing the risk of small-for-size syndrome after liver surgery | No | Irrelevant |
| 2636 | | Chai et al., 2022 | | | Regional Delivery of CAR-T Effectively Controls Tumor Growth in Colorectal Liver Metastasis Model | No | Irrelevant |
| 2637 | | Pan et al., 2022 | | | Characterization of a novel bispecific antibody targeting tissue factor-positive tumors with T cell engagement | No | Irrelevant |
| 2638 | | Seo et al., 2022 | | | Short Review on Advances in Hydrogel-Based Drug Delivery Strategies for Cancer Immunotherapy | No | Irrelevant |
| 2639 | | Cho et al., 2022 | | | Prognostic value of natural killer cell activity for patients with HER2+ advanced gastric cancer treated with first-line fluoropyrimidine–platinum doublet plus trastuzumab | No | Irrelevant |
| 2640 | | Khan et al., 2022 | | | MicroRNA-1: Diverse role of a small player in multiple cancers | No | Irrelevant |
| 2641 | | Wang et al., 2022 | | | Role of CD155/TIGIT in Digestive Cancers: Promising Cancer Target for Immunotherapy | No | Irrelevant |
| 2642 | | Garland et al., 2022 | | | Chemical and Biomolecular Strategies for STING Pathway Activation in Cancer Immunotherapy | No | Irrelevant |
| 2643 | | Delvecchio et al., 2022 | | | B cells in pancreatic cancer stroma | No | Irrelevant |
| 2644 | | Ren et al., 2022 | | | A novel immune-related gene signature predicting survival in sarcoma patients | No | Irrelevant |
| 2645 | | Wang et al., 2022 | | | Prostaglandin Pathways: Opportunities for Cancer Prevention and Therapy | No | Irrelevant |
| 2646 | | Lee et al., 2022 | | | Tissue Resident Foxp3+ Regulatory T Cells: Sentinels and Saboteurs in Health and Disease | No | Irrelevant |
| 2647 | | Ginghina et al., 2022 | | | Liquid Biopsy and Artificial Intelligence as Tools to Detect Signatures of Colorectal Malignancies: A Modern Approach in Patient’s Stratification | No | Irrelevant |
| 2648 | | Meylan et al., 2022 | | | Tertiary lymphoid structures generate and propagate anti-tumor antibody-producing plasma cells in renal cell cancer | No | Irrelevant |
| 2649 | | Reyes et al., 2022 | | | Precision medicine for advanced colorectal and gastroesophageal cancer | No | Irrelevant |
| 2650 | | Tay et al., 2022 | | | PD-L1 expression in non-dysplastic, dysplastic and oral squamous cell carcinoma samples | No | Irrelevant |
| 2651 | | Dhasmana et al., 2022 | | | A topography of immunotherapies against gastrointestinal malignancies | No | Irrelevant |
| 2652 | | Colonne et al., 2022 | | | The Intriguing Connections between von Willebrand Factor, ADAMTS13 and Cancer | No | Irrelevant |
| 2653 | | Zhao et al., 2022 | | | Role of Base Excision Repair in Innate Immune Cells and Its Relevance for Cancer Therapy | No | Irrelevant |
| 2654 | | Wu et al., 2022 | | | Current status, controversy and challenge in the neoadjuvant immunotherapy of colorectal cancer | No | Irrelevant |
| 2655 | | Gong et al., 2022 | | | Immune checkpoint inhibitors in the treatment of colorectal cancer: a review of clinical trials | No | Irrelevant |
| 2656 | | Takaki et al., 2022 | | | Role of Extracellular High-Mobility Group Box-1 as a Therapeutic Target of Gastric Cancer | No | Irrelevant |
| 2657 | | Kanvinde et al., 2022 | | | Non-Viral Vectors for Delivery of Nucleic Acid Therapies for Cancer | No | Irrelevant |
| 2658 | | Ulreich et al., 2022 | | | High Expression of Casein Kinase 2 Alpha Is Responsible for Enhanced Phosphorylation of DNA Mismatch Repair Protein MLH1 and Increased Tumor Mutation Rates in Colorectal Cancer | No | Irrelevant |
| 2659 | | Redman et al., 2022 | | | A Randomized Phase II Trial of mFOLFOX6 + Bevacizumab Alone or with AdCEA Vaccine + Avelumab Immunotherapy for Untreated Metastatic Colorectal Cancer | No | Irrelevant |
| 2660 | | Juat et al., 2022 | | | Adoptive T-Cell Therapy in Advanced Colorectal Cancer: A Systematic Review | No | Irrelevant |
| 2661 | | Otaegi-Ugartemendia et al., 2022 | | | Impact of Cancer Stem Cells on Therapy Resistance in Gastric Cancer | No | Irrelevant |
| 2662 | | Parakh et al., 2022 | | | Radiolabeled Antibodies for Cancer Imaging and Therapy | No | Irrelevant |
| 2663 | | Wu et al., 2022 | | | Therapeutic Approaches Targeting Proteins in Tumor-Associated Macrophages and Their Applications in Cancers | No | Irrelevant |
| 2664 | | Nguyen et al., 2022 | | | Emerging Novel Combined CAR-T Cell Therapies | No | Irrelevant |
| 2665 | | Leitner et al., 2022 | | | Insulin and cancer: a tangled web | No | Irrelevant |
| 2666 | | Dobosz et al., 2022 | | | Challenges of the Immunotherapy: Perspectives and Limitations of the Immune Checkpoint Inhibitor Treatment | No | Irrelevant |
| 2667 | | Voutsadakis et al., 2022 | | | Urothelial Bladder Carcinomas with High Tumor Mutation Burden Have a Better Prognosis and Targetable Molecular Defects beyond Immunotherapies | No | Irrelevant |
| 2668 | | Plundrich et al., 2022 | | | Molecular Mechanisms of Tumor Immunomodulation in the Microenvironment of Colorectal Cancer | No | Irrelevant |
| 2669 | | Liu et al., 2022 | | | RAGE Inhibitors as Alternatives to Dexamethasone for Managing Cerebral Edema Following Brain Tumor Surgery | No | Irrelevant |
| 2670 | | Sun et al., 2022 | | | EZH2, a prominent orchestrator of genetic and epigenetic regulation of solid tumor microenvironment and immunotherapy | No | Irrelevant |
| 2671 | | Amatu et al., 2022 | | | Efficacy of Retreatment with Oxaliplatin-Based Regimens in Metastatic Colorectal Cancer Patients: The RETROX-CRC Retrospective Study | No | Irrelevant |
| 2672 | | Ranasinghe et al., 2022 | | | A synopsis of modern-day colorectal cancer: Where we stand | No | Irrelevant |
| 2673 | | Park et al., 2022 | | | Artificial Intelligence for Predicting Microsatellite Instability Based on Tumor Histomorphology: A Systematic Review | No | Irrelevant |
| 2674 | | Li et al., 2022 | | | METTL3 promotes oxaliplatin resistance of gastric cancer CD133+ stem cells by promoting PARP1 mRNA stability | No | Irrelevant |
| 2675 | | Reusch et al., 2022 | | | Cryopreservation of Natural Killer Cells Pre-Complexed with Innate Cell Engagers | No | Irrelevant |
| 2676 | | Saleh et al., 2022 | | | Prognostic Value of Programmed Death Ligand-1 Expression in Solid Tumors Irrespective of Immunotherapy Exposure: A Systematic Review and Meta-Analysis | No | Irrelevant |
| 2677 | | Song et al., 2022 | | | Limitations and opportunities of technologies for the analysis of cell-free DNA in cancer diagnostics | No | Irrelevant |
| 2678 | | Contino et al., 2022 | | | The gut microbiota can be a potential regulator and treatment target of bone metastasis | No | Irrelevant |
| 2679 | | Shah et al., 2022 | | | Therapeutic implications of germline vulnerabilities in DNA repair for precision oncology | No | Irrelevant |
| 2680 | | Arifa et al., 2022 | | | Eosinophil plays a crucial role in intestinal mucositis induced by antineoplastic chemotherapy | No | Irrelevant |
| 2681 | | Chamseddine et al., 2022 | | | Modulating tumor-associated macrophages to enhance the efficacy of immune checkpoint inhibitors: A TAM-pting approach | No | Irrelevant |
| 2682 | | Neiheisel et al., 2022 | | | Wnt pathway modulators in cancer therapeutics: An update on completed and ongoing clinical trials | No | Irrelevant |
| 2683 | | Uson et al., 2022 | | | Germline Cancer Susceptibility Gene Testing in Unselected Patients With Colorectal Adenocarcinoma: A Multicenter Prospective Study | No | Irrelevant |
| 2684 | | Cocks et al., 2022 | | | The Immune Checkpoint Inhibitor LAG-3 and Its Ligand GAL-3 in Vulvar Squamous Neoplasia | No | Irrelevant |
| 2685 | | Grundy et al., 2022 | | | Transposable element regulation and expression in cancer | No | Irrelevant |
| 2686 | | Zhang et al., 2022 | | | The combination of novel immune checkpoints HHLA2 and ICOSLG: A new system to predict survival and immune features in esophageal squamous cell carcinoma | No | Irrelevant |
| 2687 | | Feng et al., 2022 | | | Oral Administration of Probiotics Reduces Chemotherapy-Induced Diarrhea and Oral Mucositis: A Systematic Review and Meta-Analysis | No | Irrelevant |
| 2688 | | Ryder et al., 2022 | | | Chemical Modulation of Gasdermin-Mediated Pyroptosis and Therapeutic Potential | No | Irrelevant |
| 2689 | | Xiang et al., 2022 | | | The Roles of Mesenchymal Stem Cells in Gastrointestinal Cancers | No | Irrelevant |
| 2690 | | Cherney et al., 2022 | | | Discovery of Non-Nucleotide Small-Molecule STING Agonists via Chemotype Hybridization | No | Irrelevant |
| 2691 | | Fang et al., 2022 | | | The Prognostic Value of GNG7 in Colorectal Cancer and Its Relationship With Immune Infiltration | No | Irrelevant |
| 2692 | | Xie et al., 2022 | | | Zinc finger protein 277 is an intestinal transit-amplifying cell marker and colon cancer oncogene | No | Irrelevant |
| 2693 | | Shen et al., 2022 | | | DNA Damage and Activation of cGAS/STING Pathway Induce Tumor Microenvironment Remodeling | No | Irrelevant |
| 2694 | | Feng et al., 2022 | | | Epigenetic Inheritance From Normal Origin Cells Can Determine the Aggressive Biology of Tumor-Initiating Cells and Tumor Heterogeneity | No | Irrelevant |
| 2695 | | Désage et al., 2022 | | | Targeting KRAS Mutant in Non-Small Cell Lung Cancer: Novel Insights Into Therapeutic Strategies | No | Irrelevant |
| 2696 | | Feng et al., 2022 | | | A Prognostic Model Using Immune-Related Genes for Colorectal Cancer | No | Irrelevant |
| 2697 | | Zhao et al., 2022 | | | Guanylate-Binding Protein 1 as a Potential Predictor of Immunotherapy: A Pan-Cancer Analysis | No | Irrelevant |
| 2698 | | Zhang et al., 2022 | | | Links Between N6-Methyladenosine and Tumor Microenvironments in Colorectal Cancer | No | Irrelevant |
| 2699 | | Bian et al., 2022 | | | Pt(II)-NHC Complex Induces ROS-ERS-Related DAMP Balance to Harness Immunogenic Cell Death in Hepatocellular Carcinoma | No | Irrelevant |
| 2700 | | Fanale et al., 2022 | | | Impact of Different Selection Approaches for Identifying Lynch Syndrome-Related Colorectal Cancer Patients: Unity Is Strength | No | Irrelevant |
| 2701 | | Cao et al., 2022 | | | Therapeutic Interventions Targeting Innate Immune Receptors: A Balancing Act | No | Irrelevant |
| 2702 | | Zeng et al., 2022 | | | The Predictive Value of PAK7 Mutation for Immune Checkpoint Inhibitors Therapy in Non-Small Cell Cancer | No | Irrelevant |
| 2703 | | Li et al., 2022 | | | Functional and Therapeutic Significance of Tumor-Associated Macrophages in Colorectal Cancer | No | Irrelevant |
| 2704 | | Prisciandaro et al., 2022 | | | Biomarker Landscape in Neuroendocrine Tumors With High-Grade Features: Current Knowledge and Future Perspective | No | Irrelevant |
| 2705 | | Zhang et al., 2022 | | | Diagnostic and prognostic role of basic leucine zipper transcription factor in kidney renal clear cell carcinoma | No | Irrelevant |
| 2706 | | Carlsen et al., 2022 | | | Immunotherapy for Colorectal Cancer: Mechanisms and Predictive Biomarkers | No | Irrelevant |
| 2707 | | Boyer et al., 2022 | | | Influence of the Metabolism on Myeloid Cell Functions in Cancers: Clinical Perspectives | No | Irrelevant |
| 2708 | | Long et al., 2022 | | | Circular RNAs and Drug Resistance in Genitourinary Cancers: A Literature Review | No | Irrelevant |
| 2709 | | Singh et al., 2022 | | | Cytokines and Chemokines in Cancer Cachexia and Its Long-Term Impact on COVID-19 | No | Irrelevant |
| 2710 | | Gorzo et al., 2022 | | | Landscape of Immunotherapy Options for Colorectal Cancer: Current Knowledge and Future Perspectives beyond Immune Checkpoint Blockade | No | Irrelevant |
| 2711 | | Santos et al., 2022 | | | Liquid biopsy: The value of different bodily fluids | No | Irrelevant |
| 2712 | | Hylander et al., 2022 | | | Using Mice to Model Human Disease: Understanding the Roles of Baseline Housing-Induced and Experimentally Imposed Stresses in Animal Welfare and Experimental Reproducibility | No | Irrelevant |
| 2713 | | Hong et al., 2022 | | | Mogamulizumab in Combination with Nivolumab in a Phase I/II Study of Patients with Locally Advanced or Metastatic Solid Tumors | No | Irrelevant |
| 2714 | | El-Sayes et al., 2022 | | | A Combination of Chemotherapy and Oncolytic Virotherapy Sensitizes Colorectal Adenocarcinoma to Immune Checkpoint Inhibitors in a cDC1-Dependent Manner | No | Irrelevant |
| 2715 | | Palicelli et al., 2022 | | | What Do We Have to Know about PD-L1 Expression in Prostate Cancer? A Systematic Literature Review | No | Irrelevant |
| 2716 | | Leibetseder et al., 2022 | | | New Approaches with Precision Medicine in Adult Brain Tumors | No | Irrelevant |
| 2717 | | Vogel et al., 2022 | | | The American Society of Colon and Rectal Surgeons Clinical Practice Guidelines for the Management of Colon Cancer | No | Irrelevant |
| 2718 | | Verhulst et al., 2022 | | | Validating Cell Surface Proteases as Drug Targets for Cancer Therapy: What Do We Know, and Where Do We Go? | No | Irrelevant |
| 2719 | | Jiachen et al., 2022 | | | Retrospective analysis of the preparation and application of immunotherapy in cancer treatment | No | Irrelevant |
| 2720 | | Fanale et al., 2022 | | | Can the tumor-agnostic evaluation of MSI/MMR status be the common denominator for the immunotherapy treatment of patients with several solid tumors? | No | Irrelevant |
| 2721 | | Langiu et al., 2022 | | | Neutrophils, Cancer and Thrombosis: The New Bermuda Triangle in Cancer Research | No | Irrelevant |
| 2722 | | Feng et al., 2022 | | | Systematic characterization of the tumor microenvironment in Chinese patients with hepatocellular carcinoma highlights intratumoral B cells as a potential immunotherapy target | No | Irrelevant |
| 2723 | | Hamed et al., 2022 | | | Inoperable de novo metastatic colorectal cancer with primary tumour in situ: Evaluating discordant responses to upfront systemic therapy of the primary tumours and metastatic sites and complications arising from primary tumours | No | Irrelevant |
| 2724 | | Baradaran et al., 2022 | | | The cross-talk between tumor-associated macrophages and tumor endothelium: Recent advances in macrophage-based cancer immunotherapy | No | Irrelevant |
| 2725 | | de Souza et al., 2022 | | | Strategies for the treatment of colorectal cancer caused by gut microbiota | No | Irrelevant |
| 2726 | | Weiz et al., 2022 | | | Glycosylated 4-methylumbelliferone as a targeted therapy for hepatocellular carcinoma | No | Irrelevant |
| 2727 | | Pal et al., 2022 | | | Current advances in prognostic and diagnostic biomarkers for solid cancers: Detection techniques and future challenges | No | Irrelevant |
| 2728 | | Hou et al., 2022 | | | Gut microbiota-derived short-chain fatty acids and colorectal cancer: Ready for clinical translation? | No | Irrelevant |
| 2729 | | Furuta et al., 2022 | | | Acquired idiopathic generalized anhidrosis following pembrolizumab treatment | No | Irrelevant |
| 2730 | | Dall’Olio et al., 2022 | | | Tumour burden and efficacy of immune-checkpoint inhibitors | No | Irrelevant |
| 2731 | | Nardin et al., 2022 | | | Naturally Occurring Telomerase-Specific CD4 T-Cell Immunity in Melanoma | No | Irrelevant |
| 2732 | | Nguyen et al., 2022 | | | Image-based assessment of extracellular mucin-to-tumor area predicts consensus molecular subtypes (CMS) in colorectal cancer | No | Irrelevant |
| 2733 | | Samanta et al., 2022 | | | Potential Impacts of Prebiotics and Probiotics on Cancer Prevention | No | Irrelevant |
| 2734 | | Antonoff et al., 2022 | | | The roles of surgery, stereotactic radiation, and ablation for treatment of pulmonary metastases | No | Irrelevant |
| 2735 | | Melaiu et al., 2022 | | | News on immune checkpoint inhibitors as immunotherapy strategies in adult and pediatric solid tumors | No | Irrelevant |
| 2736 | | Talaat et al., 2022 | | | Complement System: An Immunotherapy Target in Colorectal Cancer | No | Irrelevant |
| 2737 | | Vitorino et al., 2022 | | | Human Microbiota and Immunotherapy in Breast Cancer - A Review of Recent Developments | No | Irrelevant |
| 2738 | | Geng et al., 2022 | | | Over-Expression and Prognostic Significance of FATP5, as a New Biomarker, in Colorectal Carcinoma | No | Irrelevant |
| 2739 | | Jiang et al., 2022 | | | Semaphorins as Potential Immune Therapeutic Targets for Cancer | No | Irrelevant |
| 2740 | | Witte et al., 2022 | | | Integrative genomic and transcriptomic analysis in plasmablastic lymphoma identifies disruption of key regulatory pathways | No | Irrelevant |
| 2741 | | Adeshakin et al., 2022 | | | Regulating Histone Deacetylase Signaling Pathways of Myeloid-Derived Suppressor Cells Enhanced T Cell-Based Immunotherapy | No | Irrelevant |
| 2742 | | Shi et al., 2022 | | | Immune Checkpoint LAG3 and Its Ligand FGL1 in Cancer | No | Irrelevant |
| 2743 | | Bai et al., 2022 | | | Tumor-Infiltrating Lymphocytes in Colorectal Cancer: The Fundamental Indication and Application on Immunotherapy | No | Irrelevant |
| 2744 | | Asimgil et al., 2022 | | | Targeting the undruggable oncogenic KRAS: The dawn of hope | No | Irrelevant |
| 2745 | | Li et al., 2022 | | | Back to the Future: Spatiotemporal Determinants of NK Cell Antitumor Function | No | Irrelevant |
| 2746 | | Newell et al., 2022 | | | Multiomic profiling of checkpoint inhibitor-treated melanoma: Identifying predictors of response and resistance, and markers of biological discordance | No | Irrelevant |
| 2747 | | Zhang et al., 2022 | | | Irreversible Electroporation: An Emerging Immunomodulatory Therapy on Solid Tumors | No | Irrelevant |
| 2748 | | Schumacher et al., 2022 | | | Tertiary lymphoid structures in cancer | No | Irrelevant |
| 2749 | | Tong et al., 2022 | | | The Role of m6A Epigenetic Modification in the Treatment of Colorectal Cancer Immune Checkpoint Inhibitors | No | Irrelevant |
| 2750 | | Shen et al., 2022 | | | Characterization With KRAS Mutant Is a Critical Determinant in Immunotherapy and Other Multiple Therapies for Non-Small Cell Lung Cancer | No | Irrelevant |
| 2751 | | Tachon et al., 2022 | | | HSP110 as a Diagnostic but Not a Prognostic Biomarker in Colorectal Cancer With Microsatellite Instability | No | Irrelevant |
| 2752 | | Horak et al., 2022 | | | Understanding the Role of Plasticity in Glioblastoma | No | Irrelevant |
| 2753 | | Keshavarz-Fathi et al., 2022 | | | Cancer Immunology | No | Irrelevant |
| 2754 | | Li et al., 2022 | | | Excavation and evaluation of adverse reaction signals of 4 kinds of imported PD-1/PD-L1 inhibitors | No | Irrelevant |
| 2755 | | Rizzo et al., 2022 | | | The Impact of the Extracellular Matrix on Immunotherapy Success | No | Irrelevant |
| 2756 | | Wang et al., 2022 | | | Prognostic Role of Immune-related Genes in Hepatocellular Carcinoma | No | Irrelevant |
| 2757 | | Pandey et al., 2022 | | | Sensing Soluble Immune Checkpoint Molecules and Disease-Relevant Cytokines in Cancer: A Novel Paradigm in Disease Diagnosis and Monitoring | No | Irrelevant |
| 2758 | | Gelli et al., 2022 | | | Liver Transplantation for Colorectal Cancer Liver Metastases | No | Irrelevant |
| 2759 | | Jasani et al., 2022 | | | Precision Cancer Medicine: Role of the Pathologist | No | Irrelevant |
| 2760 | | Chen et al., 2022 | | | Overview of Immunohistochemistry Assessment of Cancer-Related Predictive Biomarkers and Common Genetic Alterations | No | Irrelevant |
| 2761 | | Murphy et al., 2022 | | | The human microbiome and the tumor microenvironment | No | Irrelevant |
| 2762 | | Jebsen et al., 2022 | | | Biomarker Panels and Contemporary Practice in Clinical Trials of Personalized Medicine | No | Irrelevant |
| 2763 | | Aluri et al., 2022 | | | Protein and Peptide-Based Therapeutics for Cancer Imaging | No | Irrelevant |
| 2764 | | Ramchandani et al., 2022 | | | Regulation of Tumor Progression and Metastasis by Bone Marrow-Derived Microenvironments | No | Irrelevant |
| 2765 | | Sim et al., 2022 | | | Cancer Biomarkers: A Long and Tortuous Journey | No | Irrelevant |
| 2766 | | Duarte Mendes et al., 2022 | | | Modulation of Tumor Environment in Colorectal Cancer – Could Gut Microbiota Be a Key Player? | No | Irrelevant |
| 2767 | | Martianov et al., 2022 | | | Molecular Genetic Testing in Colon Cancer: Clinical Aspects | No | Irrelevant |
| 2768 | | Majumdar et al., 2022 | | | Diagnostic Special Stains, Immunohistochemical Markers, and Special Techniques Used in Gastrointestinal Tract Pathology | No | Irrelevant |
| 2769 | | Rastogi et al., 2022 | | | Molecular Classifications of Gastrointestinal Tract Tumors | No | Irrelevant |
| 2770 | | McGraw et al., 2022 | | | γδ T Cell Costimulatory Ligands in Antitumor Immunity | No | Irrelevant |
| 2771 | | Mahalanobis et al., 2022 | | | Cancer Prognosis and Immune System | No | Irrelevant |
| 2772 | | Pereira et al., 2022 | | | Breast Cancer and Next-Generation Sequencing: Towards Clinical Relevance and Future | No | Irrelevant |
| 2773 | | Muzaffar et al., 2022 | | | Nuclear Oncology | No | Irrelevant |
| 2774 | | Ahsan et al., 2022 | | | Biomarkers for Cancer Immunotherapy | No | Irrelevant |
| 2775 | | Volovat et al., 2022 | | | Nanotechnology and Immunomodulators in Cancer | No | Irrelevant |
| 2776 | | Lakshmanan et al., 2022 | | | Crosstalk of Immuno-Oncology and Metabolism: Influence of Akkermansia muciniphila and Personalized Therapy Approach | No | Irrelevant |
| 2777 | | Bharadwaj et al., 2022 | | | Recent Developments in the Immunotherapeutic Approaches for Cancer Treatment | No | Irrelevant |
| 2778 | | Cyr et al., 2022 | | | Cannabis and Cannabinoid-Based Medicines in Cancer Care: A Comprehensive Guide to Medical Management | No | Irrelevant |
| 2779 | | Mackintosh et al., 2022 | | | Interstitial Abnormalities from Solid and Haematological Cancers | No | Irrelevant |
| 2780 | | Toper et al., 2022 | | | Diagnostic Approach According to Uncovered Metastatic Sites: Mediastinum-Thymus, Peritoneum, Central Nervous System, and Soft Tissue | No | Irrelevant |
| 2781 | | Pehlivanoglu et al., 2022 | | | Biomarkers in Gastrointestinal System Carcinomas | No | Irrelevant |
| 2782 | | Cavalcanti et al., 2022 | | | Chemotherapy Protocols and Infusion Sequence: Schedule Consideration in Cancer Treatment | No | Irrelevant |
| 2783 | | Adam et al., 2022 | | | Downsizing Chemotherapy for Liver Metastases from Colorectal Cancer | No | Irrelevant |
| 2784 | | Mahdavi Sharif et al., 2022 | | | Tumor Immunology | No | Irrelevant |
| 2785 | | Yu et al., 2022 | | | Initial Systemic Chemotherapy for Metastatic Colorectal Cancer | No | Irrelevant |
| 2786 | | Huey et al., 2022 | | | Treatment Refractory Metastatic Colorectal Cancer | No | Irrelevant |
| 2787 | | De Wilton Marsh et al., 2022 | | | Immunotherapy | No | Irrelevant |
| 2788 | | Kushekhar et al., 2022 | | | Role of Lymphocytes in Cancer Immunity and Immune Evasion Mechanisms | No | Irrelevant |
| 2789 | | Truong et al., 2022 | | | Hereditary Cancer and Genetics in Renal Cell Carcinoma | No | Irrelevant |
| 2790 | | Liang et al., 2022 | | | Molecular and Genetic Profiling for the Diagnosis and Therapy of Hepatobiliary and Pancreatic Malignancies | No | Irrelevant |
| 2791 | | Teranishi et al., 2022 | | | Combination of Pembrolizumab With Platinum-containing Chemotherapy for Pulmonary Enteric Adenocarcinoma | No | Irrelevant |
| 2792 | | Manne et al., 2022 | | | Immunotherapy and Targeted Therapies for Colorectal Liver Metastasis | No | Irrelevant |
| 2793 | | Siegel et al., 2022 | | | The Tumor Microenvironment of Colorectal Cancer Liver Metastases: Molecular Mediators and Future Therapeutic Targets | No | Irrelevant |
| 2794 | | Blair et al., 2022 | | | Use of Molecular Markers and Other Personalized Factors in Treatment Decisions for Metastatic Colorectal Cancer | No | Irrelevant |
| 2795 | | Horak et al., 2022 | | | Understanding the Role of Plasticity in Glioblastoma | No | Irrelevant |
| 2796 | | Vranic et al., 2022 | | | Tumor-Type Agnostic, Targeted Therapies: BRAF Inhibitors Join the Group | No | Irrelevant |
| 2797 | | Allende et al., 2022 | | | Molecular Testing of Gastrointestinal Neoplasms | No | Irrelevant |
| 2798 | | Wang et al., 2022 | | | The Emerging Role of Triggering Receptor Expressed on Myeloid Cell-2 in Malignant Tumor | No | Irrelevant |
| 2799 | | Guerrero et al., 2022 | | | Targeting BRAF V600E in Metastatic Colorectal Cancer: Where Are We Today? | No | Irrelevant |
| 2800 | | Hu et al., 2022 | | | Cincumol Prevents Malignant Phenotype of Colorectal Cancer Cell Line HCT116 via Inhibiting PI3K/AKT Signaling In Vitro | No | Irrelevant |
| 2801 | | Saskova et al., 2022 | | | Salivary and Serum Neopterin and Interleukin 6 as Biomarkers in Patients with Oral and Oropharyngeal Squamous Cell Carcinoma | No | Irrelevant |
| 2802 | | Saoudi Gonzalez et al., 2022 | | | Pharmacokinetics and Pharmacodynamics of Approved Monoclonal Antibody Therapy for Colorectal Cancer | No | Irrelevant |
| 2803 | | Sorokin et al., 2022 | | | Clinically Relevant Fusion Oncogenes: Detection and Practical Implications | No | Irrelevant |
| 2804 | | Křížová et al., 2022 | | | Fecal Microbiota Transplantation – New Possibility to Influence the Results of Therapy of Cancer Patients | No | Irrelevant |
| 2805 | | Wani et al., 2022 | | | MicroRNAs as Potential Immunotherapeutic Modulators in Cancer | No | Irrelevant |
| 2806 | | Wei et al., 2022 | | | Prognostic Value and Immunological Role of FOXM1 in Human Solid Tumors | No | Irrelevant |
| 2807 | | Okoro et al., 2022 | | | BRAF Inhibitors in Carcinogenesis and Their Clinical Implications: A Review | No | Irrelevant |
| 2808 | | Keshavarz-Fathi et al., 2022 | | | Cancer Immunology | No | Irrelevant |
| 2809 | | Guisheng et al., 2022 | | | Application of Immune Checkpoint Inhibitors in Colorectal Cancer: Current and Future Strategies | No | Irrelevant |
| 2810 | | Vienot et al., 2022 | | | Chemokine Switch Regulated by TGF-β1 in Cancer-Associated Fibroblast Subsets Determines the Efficacy of Chemo-Immunotherapy | No | Irrelevant |
| 2811 | | Sud et al., 2022 | | | Incidence of Microsatellite Instability, Mutational Burden, and Actionable Alterations in Genes of Patients with Metastatic Colorectal Carcinoma: A Study from a Tertiary Care Hospital in India | No | Irrelevant |
| 2812 | | Abushukair et al., 2022 | | | Primary and Secondary Immune Checkpoint Inhibitors Resistance in Colorectal Cancer: Key Mechanisms and Ways to Overcome Resistance | No | Irrelevant |
| 2813 | | Donnini et al., 2022 | | | Antiangiogenic Drugs: Chemosensitizers for Combination Cancer Therapy | No | Irrelevant |
| 2814 | | Veiga et al., 2022 | | | Regulation of Immune Cells by microRNAs and microRNA-Based Cancer Immunotherapy | No | Irrelevant |
| 2815 | | Heimberger et al., 2022 | | | cGAS-STING Pathway Targeted Therapies and Their Applications in the Treatment of High-Grade Glioma | No | Irrelevant |
| 2816 | | Goyal et al., 2022 | | | Regulatory Landscape in the Approval of Cancer Vaccine | No | Irrelevant |
| 2817 | | Mukherjee et al., 2022 | | | Recent Developments in Cancer Vaccines: Where Are We? | No | Irrelevant |
| 2818 | | Rossetti et al., 2022 | | | Combination of Genetically Engineered T Cells and Immune Checkpoint Blockade for the Treatment of Cancer | No | Irrelevant |
| 2819 | | Wang et al., 2022 | | | Elevated CEA and CA 19-9 Levels Within the Normal Ranges Increase the Likelihood of CRC Recurrence in the Chinese Han Population | No | Irrelevant |
| 2820 | | Alshaibi et al., 2022 | | | Studying the Anticancer Effects of Thymoquinone on Breast Cancer Cells Through Natural Killer Cell Activity | No | Irrelevant |
| 2821 | | Zhang et al., 2022 | | | Transgelin-2 Involves in the Apoptosis of Colorectal Cancer Cells Induced by Tanshinone-IIA | No | Irrelevant |
| 2822 | | Farhangnia et al., 2022 | | | Advances in Therapeutic Targeting of Immune Checkpoints Receptors Within the CD96-TIGIT Axis: Clinical Implications and Future Perspectives | No | Irrelevant |
| 2823 | | Viveiros et al., 2022 | | | Detailed Bladder Cancer Immunoprofiling Reveals New Clues for Immunotherapeutic Strategies | No | Irrelevant |
| 2824 | | Turpin et al., 2022 | | | Therapeutic Advances in Metastatic Pancreatic Cancer: A Focus on Targeted Therapies | No | Irrelevant |
| 2825 | | Singh et al., 2022 | | | Biology and Pathophysiology of Central Nervous System Metastases | No | Irrelevant |
| 2826 | | Deluce et al., 2022 | | | The Role of Gut Microbiome in Immune Modulation in Metastatic Renal Cell Carcinoma | No | Irrelevant |
| 2827 | | Shi et al., 2022 | | | Stromal Modulation Strategies to Improve Immunotherapy Response in Cancer | No | Irrelevant |
| 2828 | | Minciuna et al., 2022 | | | The Seen and the Unseen: Molecular Classification and Image-Based Analysis of Gastrointestinal Cancers | No | Irrelevant |
| 2829 | | Roth et al., 2022 | | | Colorectal Cancer | No | Irrelevant |
| 2830 | | Srivastava et al., 2022 | | | Targeting the Altered Tyrosine Kinases in Colorectal Cancer: From Inhibitors to Drugs | No | Irrelevant |
| 2831 | | Liu et al., 2022 | | | The Predictive Value of Changes in the Absolute Counts of Peripheral Lymphocyte Subsets for Progression and Prognosis in Breast Cancer Patients | No | Irrelevant |
| 2832 | | Jia et al., 2022 | | | Investigating Human Diseases with the Microbiome: Metagenomics Bench to Bedside | No | Irrelevant |
| 2833 | | Zhang et al., 2022 | | | Protein Regulator of Cytokinesis 1 (PRC1) Upregulation Promotes Immune Suppression in Liver Hepatocellular Carcinoma | No | Irrelevant |
| 2834 | | Jain et al., 2022 | | | Host-Pathogen Protein-Nucleic Acid Interactions: A Comprehensive Review | No | Irrelevant |
| 2835 | | Mougel et al., 2022 | | | Synergistic Effect of Combining Sunitinib with a Peptide-Based Vaccine in Cancer Treatment After Microenvironment Remodeling | No | Irrelevant |
| 2836 | | Burnell et al., 2022 | | | Seven Mysteries of LAG-3: A Multi-Faceted Immune Receptor of Increasing Complexity | No | Irrelevant |
| 2837 | | De Surya, 2022 | | | Fundamentals of Cancer Detection, Treatment, and Prevention | No | Irrelevant |
| 2838 | | Yanus et al., 2022 | | | Predictive Molecular Genetic Tests in Clinical Oncology | No | Irrelevant |
| 2839 | | Rabadi et al., 2022 | | | The Role of VISTA in the Tumor Microenvironment | No | Irrelevant |
| 2840 | | Yu et al., 2022 | | | Tumor Biomarker Testing for Metastatic Colorectal Cancer: A Canadian Consensus Practice Guideline | No | Irrelevant |
| 2841 | | Li et al., 2022 | | | B7-H3-Targeted CAR-T Cell Therapy for Solid Tumors | No | Irrelevant |
| 2842 | | Li et al., 2022 | | | DNA Methyltransferase-1 in Acute Myeloid Leukemia: Beyond the Maintenance of DNA Methylation | No | Irrelevant |
| 2843 | | Khatib et al., 2022 | | | The Mosaic Puzzle of the Therapeutic Monoclonal Antibodies and Antibody Fragments - A Modular Transition from Full-Length Immunoglobulins to Antibody Mimetics | No | Irrelevant |
| 2844 | | Li et al., 2022 | | | Pan-Cancer Pyroptosis Analyses Identified Novel Immunology and Chemotherapy-Related Prognostic Signatures in Cancer Subtypes | No | Irrelevant |
| 2845 | | Shinji et al., 2022 | | | Recent Advances in the Treatment of Colorectal Cancer: A Review | No | Irrelevant |
| 2846 | | Suraya et al., 2022 | | | Immunotherapy in Advanced Non-Small Cell Lung Cancers: Current Status and Updates | No | Irrelevant |
| 2847 | | Yue et al., 2022 | | | Characterization of m6A Methylation Modification Patterns in Colorectal Cancer Determines Prognosis and Tumor Microenvironment Infiltration | No | Irrelevant |
| 2848 | | Singh et al., 2022 | | | Colorectal Cancer Management: Strategies in Drug Delivery | No | Irrelevant |
| 2849 | | Zhang et al., 2022 | | | Periostin: A Predictable Molecule to Prognosis and Chemotherapy Responses of Gastrointestinal and Hepato-Biliary-Pancreatic Malignant Tumors? | No | Irrelevant |
| 2850 | | Li et al., 2022 | | | The Predictive Value and Correlation of β-Catenin, CMTM6, and PD-L1 Expression in Colorectal Cancer | No | Irrelevant |
| 2851 | | Wu et al., 2022 | | | Abnormal Expression of N6-Methyladenosine RNA Methylation Regulator IGF2BP3 in Colon Cancer Predicts a Poor Prognosis | No | Irrelevant |
| 2852 | | Brown et al., 2022 | | | Experimental Drug Treatments for Hepatocellular Carcinoma: Clinical Trial Failures 2015 to 2021 | No | Irrelevant |
| 2853 | | Spanos, 2022 | | | Digestive System Malignancies | No | Irrelevant |
| 2854 | | Ebru Nur, 2022 | | | Therapeutic Targeting of Molecular Pathways in Colorectal Cancer | No | Irrelevant |
| 2855 | | Awad et al., 2022 | | | Emerging Applications of Nanobodies in Cancer Therapy | No | Irrelevant |
| 2856 | | Brown, 2022 | | | Engaging Pattern Recognition Receptors in Solid Tumors to Generate Systemic Antitumor Immunity | No | Irrelevant |
| 2857 | | Fu et al., 2022 | | | Discovery of New Therapeutic Targets for Osteosarcoma Treatment Based on Immune-Related lncRNAs in the Tumor Microenvironment | No | Retracted article |
| 2858 | | Varım et al., 2022 | | | The Role of Neutrophil Albumin Ratio in Predicting the Stage of Non-Small Cell Lung Cancer | No | Irrelevant |
| 2859 | | Signoroni et al., 2022 | | | Tumors of the Small Intestine, Colon, and Rectum | No | Irrelevant |
| 2860 | | Riedesser et al., 2022 | | | Precision Medicine for Metastatic Colorectal Cancer in Clinical Practice | No | Irrelevant |
| 2861 | | Dhanyamraju et al., 2022 | | | Melanoma Therapeutics: A Literature Review | No | Irrelevant |
| 2862 | | Tang et al., 2022 | | | Risk Analysis of Positive PD-L1 Expression and Clinicopathological Features and Survival Prognosis in Patients with Colorectal Cancer: Systematic Review and Meta-Analysis | No | Retracted article |
| 2863 | | Li et al., 2022 | | | Immune Suppressive Microenvironment in Brain Metastatic Non-Small Cell Lung Cancer: Comprehensive Immune Microenvironment Profiling of Brain Metastases versus Paired Primary Lung Tumors | No | Irrelevant |
| 2864 | | Grassilli et al., 2022 | | | Emerging Actionable Targets to Treat Therapy-Resistant Colorectal Cancers | No | Irrelevant |
| 2865 | | Jiang et al., 2022 | | | Emulating Interactions Between Microorganisms and Tumor Microenvironment to Develop Cancer Theranostics | No | Irrelevant |
| 2866 | | Saad, 2022 | | | Targeting Cancer-Associated Glycans as a Therapeutic Strategy in Leukemia | No | Irrelevant |
| 2867 | | Yuan et al., 2022 | | | Immunotherapy of Glioblastoma: Recent Advances and Future Prospects | No | Irrelevant |
| 2868 | | Aksoy et al., 2022 | | | siRNA-Mediated Mesothelin Silencing for Treatment of Mesothelioma | No | Irrelevant |
| 2869 | | Ducoin et al., 2022 | | | Targeting NKG2A to Boost Anti-Tumor CD8 T-Cell Responses in Human Colorectal Cancer | No | Irrelevant |
| 2870 | | Yu et al., 2022 | | | Diabetes and Colorectal Cancer Risk: Clinical and Therapeutic Implications | No | Irrelevant |
| 2871 | | Lin et al., 2022 | | | Pan-Cancer Analyses Confirmed the Ferroptosis-Related Gene SLC7A11 as a Prognostic Biomarker for Cancer | No | Irrelevant |
| 2872 | | Başoğlu et al., 2022 | | | Prognostic Value of Tissue-Resident Memory T Cells and Tumor Microenvironmental Features in Resected Pancreatic Adenocarcinoma | No | Irrelevant |
| 2873 | | Khosla et al., 2022 | | | Small Bowel Adenocarcinoma: An Overview | No | Irrelevant |
| 2874 | | Mini et al., 2022 | | | Predictive “Omic” Biomarkers of Drug Response: Colorectal Cancer as a Model | No | Irrelevant |
| 2875 | | Vaněk et al., 2022 | | | Natural Killer Cell-Based Strategies for Immunotherapy of Cancer | No | Irrelevant |
| 2876 | | Elsayed et al., 2022 | | | Multi-OMICs Data Analysis Identifies Molecular Features Correlating with Tumor Immunity in Colon Cancer | No | Irrelevant |
| 2877 | | Ros et al., 2022 | | | Ongoing and Evolving Clinical Trials Enhancing Future Colorectal Cancer Treatment Strategies | No | Irrelevant |
| 2878 | | Yazici et al., 2022 | | | Metastatic Colorectal Cancer in Both Sides of Aegean Sea: Practice Patterns and Outcome | No | Irrelevant |
| 2879 | | Crutcher et al., 2022 | | | Emerging Drug Targets for Colon Cancer: A Preclinical Assessment | No | Irrelevant |
| 2880 | | Redwood et al., 2022 | | | What’s Next in Cancer Immunotherapy? - The Promise and Challenges of Neoantigen Vaccination | No | Irrelevant |
| 2881 | | Passirani et al., 2022 | | | Modulating Undruggable Targets to Overcome Cancer Therapy Resistance | No | Irrelevant |
| 2882 | | Bian et al., 2022 | | | A Review on the Application of PD-1 Blockade in EBV-Associated Nasopharyngeal Carcinoma Immunotherapy | No | Irrelevant |
| 2883 | | Garmezy et al., 2022 | | | Clinical and Molecular Characterization of POLE Mutations as Predictive Biomarkers of Response to Immune Checkpoint Inhibitors in Advanced Cancers | No | Irrelevant |
| 2884 | | Zhang et al., 2022 | | | SPARCL1 Is a Novel Prognostic Biomarker and Correlates with Tumor Microenvironment in Colorectal Cancer | No | Irrelevant |
| 2885 | | Zhang et al., 2022 | | | Neurotoxicity of Tumor Immunotherapy: The Emergence of Clinical Attention | No | Irrelevant |
| 2886 | | Wang et al., 2022 | | | Integrative Modeling of Multiomics Data for Predicting Tumor Mutation Burden in Patients with Lung Cancer | No | Irrelevant |
| 2887 | | Jiang et al., 2022 | | | Research Progress of Immunotherapy Biomarkers for Non-Small Cell Lung Cancer | No | Irrelevant |
| 2888 | | Van Toledo et al., 2022 | | | Current Approaches in Managing Colonic Serrated Polyps and Serrated Polyposis | No | Irrelevant |
[truncated: 1,368,810 more chars]
